# Clinical efficacy and safety of sodium-glucose cotransporter protein-2 (SGLT-2) inhibitor, glucagon-like peptide-1 (GLP-1) receptor agonist, and Finerenone in type 2 diabetes mellitus with non-dialysis chronic kidney disease: a network meta-analysis of randomized clinical trials

**DOI:** 10.3389/fphar.2025.1517272

**Published:** 2025-03-27

**Authors:** Jingyi Guo, Maoying Wei, Wenhua Zhang, Yijia Jiang, Aijing Li, Churan Wang, Dan Yin, Anning Sun, Yanbing Gong

**Affiliations:** ^1^ Dongzhimen Hospital, Beijing University of Chinese Medicine, Beijing, China; ^2^ Beijing University of Chinese Medicine, Beijing, China

**Keywords:** SGLT-2 inhibitors, GLP-1 receptor agonists, Finerenone, type 2 diabetes mellitus, chronic kidney disease, network meta-analysis

## Abstract

**Objective:**

To investigate the safety and clinical efficacy of sodium-glucose cotransporter protein-2 (SGLT-2) inhibitors, glucagon-like peptide-1 (GLP-1) receptor agonists and Finerenone in treating patients with type 2 diabetes mellitus (T2DM) combined with non-dialysis chronic kidney disease (CKD).

**Methods:**

Cochrane Library, PubMed, EMBASE, Web of Science, CNKI, CQVIP database, and WanFang from their inception up to November 2023 were searched to compare the efficacy and safety of SGLT-2 inhibitors, GLP-1 RA receptor agonists and Finerenone in the treatment of T2DM patients with non-dialysis CKD. To assess the methodological quality and risk of bias in the included studies, we utilized the Cochrane Risk of Bias Assessment tool (RoB 2.0). The confidence of evidence was examined using Confidence in Network Meta-Analysis (CINeMA). Traditional meta-analysis of variables was conducted using Stata 17.0 software with a random-effects model. We assessed publication bias using funnel plots and explored potential sources of heterogeneity through subgroup analysis.

**Results:**

A total of 39 studies (99,599 patients) were included. Compared to Placebo (PBO), SGLT-2 inhibitors demonstrated superior efficacy in reducing glycosylated hemoglobin (HbA1c) (MD = −0.33; 95%CI: from −0.52 to −0.15), systolic blood pressure (SBP) (MD from −5.52 to −1.50; 95%CI from −8.80 to −0.23), body weight (MD from −3.81 to −1.29; 95%CI from −6.34 to −0.84) and diastolic blood pressure (DBP) (MD = −1.86; 95%CI: −3.18, −40.54). The efficacy of Liraglutide in reducing Low-Density Lipoprotein Cholesterol (LDL-C) surpassed that of other agents (MD from −1.58 to −1.41; 95%CI from −2.05 to −0.81). Finerenone significantly reduced SBP (MD = −1.65; 95%CI: −2.48, −0.81) compared to PBO. According to the SUCRA based relative ranking of treatments, Empagliflozin was the most effective in reducing HbA1c and DBP. Semaglutide was the least harmful to estimated glomerular filtration rate. Liraglutide was the most effective in reducing LDL-C. Bexagliflozin, Canagliflozin were the most effective in reducing SBP and body weight. Finerenone had the lowest incidence of urinary tract infection, Hypoglycemia was the lowest in the Luseogliflozin group. Ertugliflozin was the least likely to cause acute kidney injury. Canagliflozin had the lowest probability of any adverse event.

**Conclusion:**

The safety of these drugs has been confirmed, except for some special drugs. SGLT-2 inhibitors had a preferential glucose-lowering and weight-loss function, GLP-1 receptor agonists had a preferential lowering of LDL-C and blood glucose, and Finereone significantly reduced SBP compared with PBO. Systematic Review Registration: PROSPERO, CRD42024571544.

## 1 Introduction

Diabetes is a global public health concern, and with the recent surge in diabetes patients, it is projected to impact 784 million individuals by 2045, posing a significant threat to human wellbeing. Diabetic kidney disease is a prominent microvascular complication of diabetes mellitus, with an estimated 40% of individuals with diabetes projected to develop chronic kidney disease (CKD) during their lifetime, potentially necessitating the need for renal replacement therapy (Afkarian et al., 2016; Scilletta et al., 2023; Zoja et al., 2020). Hence, it is imperative to prevent further progression of kidney disease in the management of type 2 diabetes mellitus (T2DM) patients with CKD.

The SGLT-2 inhibitors represent a novel class of oral hypoglycemic medications. GLP-1 receptor agonists reduce Glycosylated hemoglobin (HbA1c) by stimulating insulin secretion and reducing glucagon secretion, while also decreasing appetite through delayed gastric emptying (Filippatos et al., 2013). Several randomized controlled trials (RCTs) have demonstrated the effectiveness and safety of these two drugs (Perkovic et al., 2019; Mann et al., 2020). Finerenone, a nonsteroidal selective mineralocorticoid receptor antagonist, has shown in large RCTs to slow down CKD progression and improve cardiovascular outcomes (Pitt et al., 2021; Filippatos et al., 2021; Bakris G. L. et al., 2020). It was approved by the Food and Drug Administration (FDA) in July 2021 for treating T2DM in CKD patients. Although GLP-1 receptor agonists have been found to lower blood pressure and body weight while improving cardiovascular outcomes, there is still no clear conclusion when compared to SGLT-2 inhibitors and Finerenone for treating T2DM in non-dialysis CKD (Sun et al., 2015; Shah et al., 2014).

There is currently a lack of comprehensive evaluation of the efficacy and safety of several drugs for treating T2DM combined with CKD. Network meta-analysis (NMA) combines direct and indirect evidence to compare multiple treatments and assess their interrelationship. Our study focused on non-dialysis CKD patients (eGFR >15 mL/min/1.73 m^2^) as these drugs are not recommended for patients with low eGFR (Chinese Diabetes Society, 2025; American Diabetes Association Professional Practice Committee, 2023). Therefore, we conducted an NMA of RCTs to assess the clinical efficacy and safety of SGLT-2 inhibitors, GLP-1 receptor agonists, and Finereone in non-dialysis CKD patients with T2DM.

## 2 Methods

The reporting of this NMA follows the Preferred Reporting Items for Systematic Reviews and Meta-analyses (PRISMA) reporting guideline, and the PRISMA extension statement for Reporting of Systematic Reviews Incorporating Network Meta-analysis of healthcare interventions (PRISMA-NMA) (Moher et al., 2009; Hutton et al., 2015). The study is registered with PROSPERO, number CRD42024571544.

### 2.1 Literature review

Two investigators (J. Guo and Y. Jiang) independently searched and identified relevant studies from various databases, including Cochrane Library, PubMed, EMBASE, Web of Science, CNKI, CQVIP database, and WanFang data, from inception to November 2023. To ensure comprehensive retrieval, a combination of subject words and free words was used. Key search terms included “SGLT-2 inhibitor”, “GLP-1 receptor agonist”, “Finerenone”, “Type 2 diabetes mellitus”, “chronic kidney disease”, *etc.* The detailed search strategies for each database are described in Supplementary Appendix 2.

### 2.2 Study selection

This trial included double-blind RCTs comparing SGLT-2 inhibitor, GLP-1 receptor agonist, and Finerenone or directly with placebo in adults with T2DM and non-dialysis CKD. Studies using other control drugs, studies with repeated publications and incomplete data, studies with eGFR<15 mL/min/1.73 m^2^, studies published in languages other than Chinese or English, and studies using the drug within 3 months before screening were excluded.

### 2.3 Outcomes

The clinical outcomes assessed changes in Glycosylated hemoglobin (HbA1c), estimate glomerular filtration rate (eGFR), low-density lipoprotein cholesterol (LDL-C), systolic blood pressure (SBP), diastolic blood pressure (DBP), and body weight from baseline. Safety endpoints included any adverse events (any AE), urinary tract infections (UTI), Hypoglycemia and Acute Kidney Injury (AKI).

### 2.4 Data extraction

The search results were screened by two blinded independent researchers (J. Guo and Y. Jiang) according to the inclusion and exclusion criteria, and the abstracts of the remaining literatures were reviewed using EndNote20. Two researchers used standard data extraction tables for information extraction, judgment, and literature extraction information including: study author, publication year, intervention measures, outcomes, etc. In case of disagreement, a third researcher (W. Zhang) assisted in making a judgment.

### 2.5 Risk of bias assessment

The risk of bias assessment was conducted by 2 researchers (J. Guo and Y. Jiang) using the Cochrane Risk of Bias Assessment tool (RoB 2.0) (Sterne et al., 2019). Each study was classified as having low risk, some concerns or high risk of bias.

### 2.6 Statistical analysis

For outcome indicators, odds ratio (OR) was used for bicategorical variables, mean difference (MD) was used for continuous variables, and 95% confidence interval (95% CI) was used to represent statistical results. The findings were considered statistically significant if the 95%CI did not include the null value (0 for MD and 1 for OR). For each result were calculated using a random effects model. Bilateral P-values <0.05 were considered statistically significant. We evaluated the between-study heterogeneity using the I^2^ statistic and its associated p-values. Specifically, I^2^ values of 25%, 50%, and 75% were indicative of low, moderate, and high levels of statistical heterogeneity, respectively. Subsequently, subgroup analyses were conducted to investigate potential sources of this heterogeneity. STATA 17.0 was used for statistical analysis, evidence network and surface under the cumulative raking curve (SUCRA). We evaluated publication bias of articles using funnel plots and Egger’s test. We examined the confidence of evidence using the CINeMA (Salanti et al., 2014) web application, which grades the confidence of the results as high, moderate, low, and very low.

## 3 Result

### 3.1 Characteristics of the included studies

**FIGURE 1:**
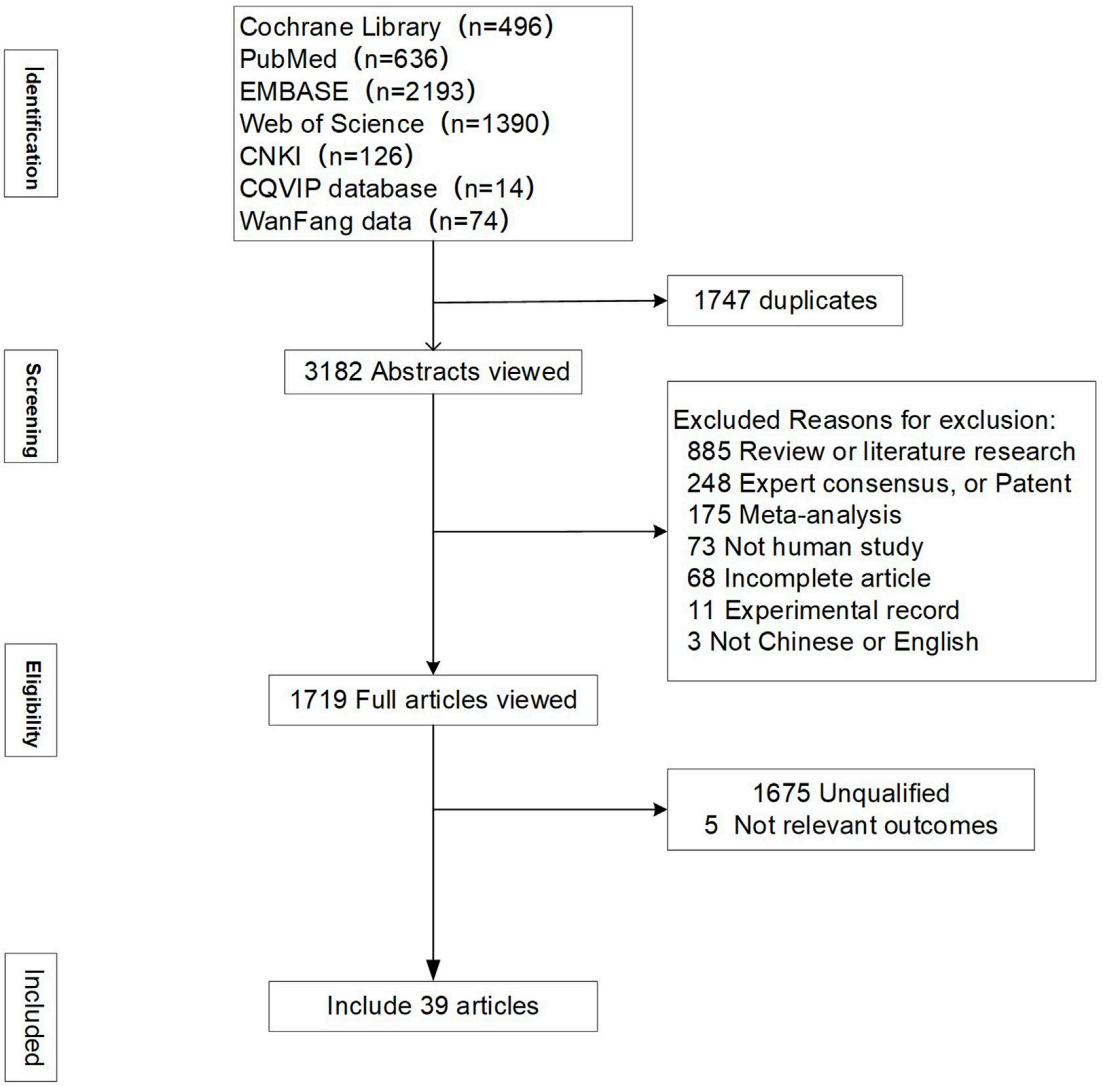
Process for identifying studies eligible for the meta-analysis.

**FIGURE 2:**
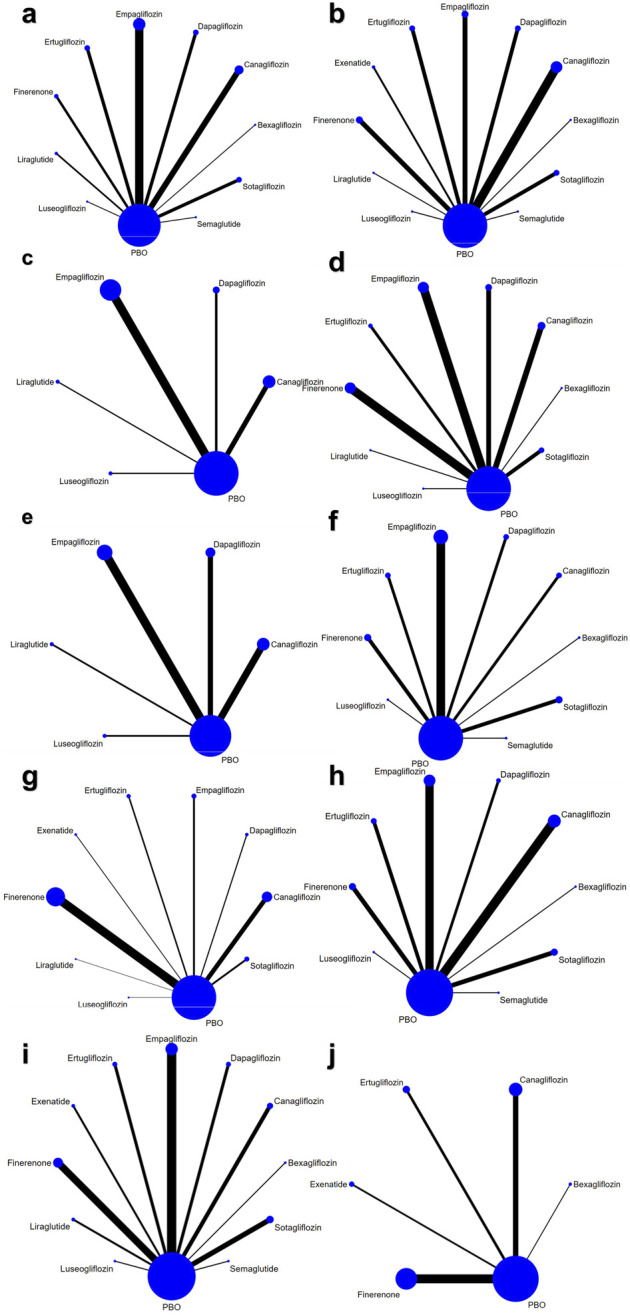
Network of eligible treatment comparisons for **(a)** HbA1c, **(b)** eGFR **(c)** LDL-C, **(d)** SBP, **(e)** DBP, **(f)** Body Weight, **(g)** any AE, **(h)** UTI, **(i)** Hypoglycemia, **(j)** AKI.

A total of 4,929 articles were retrieved. After removing duplicates, 3,182 remained. Following the title and abstract review, 1719 were selected for full text reading. Ultimately, 39 studies involving 99,599 patients were included: 23 used SGLT-2 inhibitor, 4 used GLP-1 receptor agonist and 12 used Finerenone (Figure 1). Baseline characteristics were comparable between groups. The characteristics of the included studies are shown in Table 1. Figure 2, and Supplementary Appendix 3 shows the network. (Barnett et al., 2014; Haneda et al., 2016; Curovic et al., 2022; Yale et al., 2013; Chen, 2016; Yamout et al., 2014; Pollock et al., 2019; Cherney et al., 2016; Kohan et al., 2014; Wada et al., 2022a; Wada et al., 2022b; Takashima et al., 2018; Dagogo-Jack et al., 2021; Fioretto et al., 2018; Cherney et al., 2021; Allegretti et al., 2019; Cherney et al., 2023; Sivalingam et al., 2024; George et al., 2018; Gao, 2022; Wanner et al., 2018; Sarafidis et al., 2023; Cristian et al., 2020; Bakris et al., 2020; Bakris et al., 2015; Koya et al., 2023; Agarwal et al., 2022a; Agarwal et al., 2023; Mahaffey et al., 2019; Bhatt et al., 2021; Agarwal, et al., 2022b; Zhang et al., 2023; Rosas et al., 2023; Perakakis et al., 2024; Tuttle et al., 2022; Pitt et al., 2021; Bakris et al., 2020; Filippatos et al., 2021; Perkovic et al., 2019).

**TABLE 1:** Basic characteristics of the included literature.

Study ID	Stage of CKD	Sample size			Age, mean, y (SD)			Treatment			Outcomes
		PBO group	Treatment group 1	Treatment group 2	PBO group	Treatment group 1	Treatment group 2	PBO group	Treatment group 1	Treatment group 2	
Yale et al. (2013)	CKD III	90	90	89	68.2 (8.4)	69.5 (8.2)	67.9 (8.2)	PBO	Cana	Cana	①②③④⑤⑥⑦⑩
Chen (2016)	CKD II CKD III	32	32					PBO	Lira		①②③④⑤⑧
Gao (2022)	Mild/moderate CKD	26	26		60.25 (1.52)	60.32 (1.66)		PBO	Lira		①②⑧⑩
Barnett et al. (2014)	CKD II	95	98	97	62.6 (8.1)	63.2 (8.5)	62.0 (8.4)	PBO	Empa	Empa	①②③④⑤⑥⑦⑧⑩
	CKD III	187	187		65.1 (8.2)	64.6 (8.9)	64.6 (8.9)	PBO	Empa		①②③④⑤⑥⑦⑧⑩
	CKD IV	37	37		62.9 (11.9)	65.4 (10.2)	65.4 (10.2)	PBO	Empa		①②③④⑤⑥⑦⑧⑩
Rosas et al. (2023)	CKD	1,034	1,065		64.2 (9.7)			PBO	Fine		⑩
Yamout et al. (2014)	CKD IIIA	266	216	239	66.3 (7.5)	66.2 (8.0)	66.3 (6.9)	PBO	Cana	Cana	①②③④⑥⑦⑩
	CKD IIIB	116	122	126	68.4 (7.6)	69.5 (7.9)	68.1 (8.1)	PBO	Cana	Cana	①②③④⑥⑦⑩
Cherney et al. (2016)	CKD	248	388		60.4 (10.2)	58.4 (10.5)		PBO	Empa		①②④⑤⑥⑦⑧⑩
	CKD	87	128		60.5 (10.3)	59.3 (9.9)		PBO	Empa		①②④⑤⑥⑦⑧⑩
Cherney et al. (2023)	CKD III	260	263	264	69.3 (8.1)	69.6 (7.5)	69.5 (8.2)	PBO	Sota	Sota	①②④⑥⑦⑧⑩
Curovic et al. (2022)	CKD II CKD IIIA	15	17		62.3 (7.8)	63.7 (9.0)		PBO	Dapa		①②③④⑤⑥
Takashima et al. (2018)	CKD II CKD IIIA	20	20		65.4 (10.4)	64.7 (9.8)		PBO	Dapa		①②④⑤
Sivalingam et al. (2024)	CKD	30	30		69.4 (9.1)	70.5 (6.8)		PBO	Sema		①②⑥⑦⑧
Pollock et al. (2019)	CKD II CKD III	148	145		64.7 (8.5)	64.7 (8.6)		PBO	Dapa		①②③④⑦⑧⑩
Koya et al. (2023)	CKD	2,831	2,827		63 (10)			PBO	Fine		④⑥⑧⑨⑩
Cristian et al. (2020)	CKD II	1,043	772		56.9 (9.5)	56.9 (9.3)		PBO	Exen		②⑦⑨⑩
	CKD III	207	182		62.2 (9.0)	62.5 (9.0)		PBO	Exen		②⑧⑨⑩
Fioretto et al. (2018)	CKD IIIA	161	160		66.2	65.3		PBO	Dapa		①②④⑥⑦⑧⑩
Kohan et al. (2014)	CKD III	84	83	85	67 (8.6)	66 (8.9)	68 (7.7)	PBO	Dapa	Dapa	①②④⑤⑥⑦⑧⑩
Allegretti et al. (2019)	CKD III	155	157		69.9 (8.29)	69.3 (8.36)		PBO	Bexa		①②④⑥⑦⑧⑨
Haneda et al. (2016)	CKD	50	95		68.4 (8.9)	67.9 (8.9)		PBO	Luse		①②③④⑤⑥⑦⑧⑩
Tuttle et al. (2022)	CKD IIIA	519	1,003		67.1 (8.1)	67.1 (7.5)		PBO	Empa		⑦⑧
	CKD IIIB	277	445		67.9 (8.2)	67.7 (8.7)		PBO	Empa		⑦⑧
	CKD IV	52	71		63.7 (10.7)	68.8 (9.1)		PBO	Empa		⑦⑧
Sarafidis et al. (2023)	CKD IV	450	440		67 (9)	67 (9)		PBO	Fine		②④⑨⑩
Mahaffey et al. (2019)	CKD	1,092	1,089		61.7 (9.4)	61.1 (9.7)		PBO	Cana		⑦⑧⑨⑩
	CKD	1,107	1,113		64.6 (8.9)	64.6 (8.2)		PBO	Cana		⑦⑧⑨⑩
Wada et al. (2022a)	CKD	154	154		62.4 (11.1)	62.5 (10.5)		PBO	Cana		①②④⑤⑦⑧⑩
Bakris et al. (2020a)	CKD IV	90	84		66 (9)	64 (10)		PBO	Cana		②⑨⑩
George et al. (2018)	CKD III	154	158	155	67.5 (8.9)	66.7 (8.3)	67.5 (8.5)	PBO	Ertu	Ertu	①②⑦⑩
Dagogo-Jack et al. (2021)	CKD III	598	618	560	68.0 (7.5)	68.3 (7.7)	68.2 (7.5)	PBO	Ertu	Ertu	①②④⑥⑦⑧⑨⑩
Cherney et al. (2021)	CKD IV	93	92	92	68.0 (8.3)	66.8 (10)	67.3 (9.6)	PBO	Sota	Sota	①②④⑥⑦⑧⑩
Bhatt et al. (2021)	CKD	5,292	5,292					PBO	Sota		⑦⑧⑩
Pitt et al. (2021)	CKD	3,666	3,686		64.1 (10)	64.1 (9.7)		PBO	Fine		①④⑥⑦⑧⑨⑩
Bakris et al. (2020b)	CKD	2,841	2,833		65.7 (9.2)	65.4 (8.9)		PBO	Fine		①②④⑥⑦⑧⑨⑩
Filippatos et al. (2021)	CKD	1,302	1,303		67.1 (8.4)	66 (8.2)		PBO	Fine		②④⑦⑧⑨⑩
	CKD	1,539	1,530		64.5 (9.6)	64.4 (9.4)		PBO	Fine		②④⑦⑧⑨⑩
Agarwal et al. (2023)	CKD II CKD III	27	92		60.8 (8.4)			PBO	Fine		④⑩
Bakris et al., (2015)	CKD II CKD III	94	727		63.26 (8.68)			PBO	Fine		②⑩
Wanner et al. (2018)	CKD I ∼ IV	752	1,498		66 (8.5)	66.2 (8)		PBO	Empa		①③④⑥
Perkovic et al. (2019)	CKD II CKD III	2,199	2,202		63.2 (9.2)	62.9 (9.2)		PBO	Cana		①②④⑤⑦⑧⑨⑩
Agarwal et al. (2022a)	CKD	2,328	2,291		65.4 (9.3)	65.2 (9.0)		PBO	Fine		⑨⑩
Wada et al. (2022b)	CKD II CKD III	303	301		60.9 (9.1)	60.6 (9.1)		PBO	Cana		①②④⑤⑦⑨⑩
		1,894	1,899		63.5 (9.2)	63.2 (9.1)		PBO	Cana		①②④⑤⑦⑨⑩
Zhang et al. (2023)	CKD	184	188		60.68 (10.13)	59.85 (10.16)		PBO	Fine		⑨⑩
Agarwal et al. (2022b)	CKD	6,507	6,519		64.8 (9.7)	64.7 (9.4)		PBO	Fine		④⑦⑧⑨⑩
Perakakis et al. (2024)	CKD	5,297	5,340		64.31 (9.56)	64.29 (9.27)		PBO	Fine		⑩
		1,025	1,067		61.71 (10.08)	61.94 (9.76)		PBO	Fine		⑩
		139	152		72.40 (8.02)	70.89 (7.06)		PBO	Fine		⑩

Abbreviations: ①Change in HbA1c from baseline; ②Change in eGFR from baseline; ③Change in LDL-C from baseline; ④Change in SBP from baseline; ⑤Change in DBP from baseline; ⑥Change in body weight from baseline; ⑦UTI; ⑧Hypoglycemia; ⑨AKI; ⑩any AE. NI, No Information. PBO: PBO group. CKD: chronic kidney disease. Cana: Canagliflozin. Lira: Liraglutide. Empa: Empagliflozin. Fine: Finerenone. Sota: Sotagliflozin. Dapa: Dapagliflozin. Sema: Semaglutide. Exen: Exenatide Bexa: Bexagliflozin. Luse: Luseogliflozin. Ertu: Ertugliflozin.

### 3.2 Risk of bias

**FIGURE 3:**
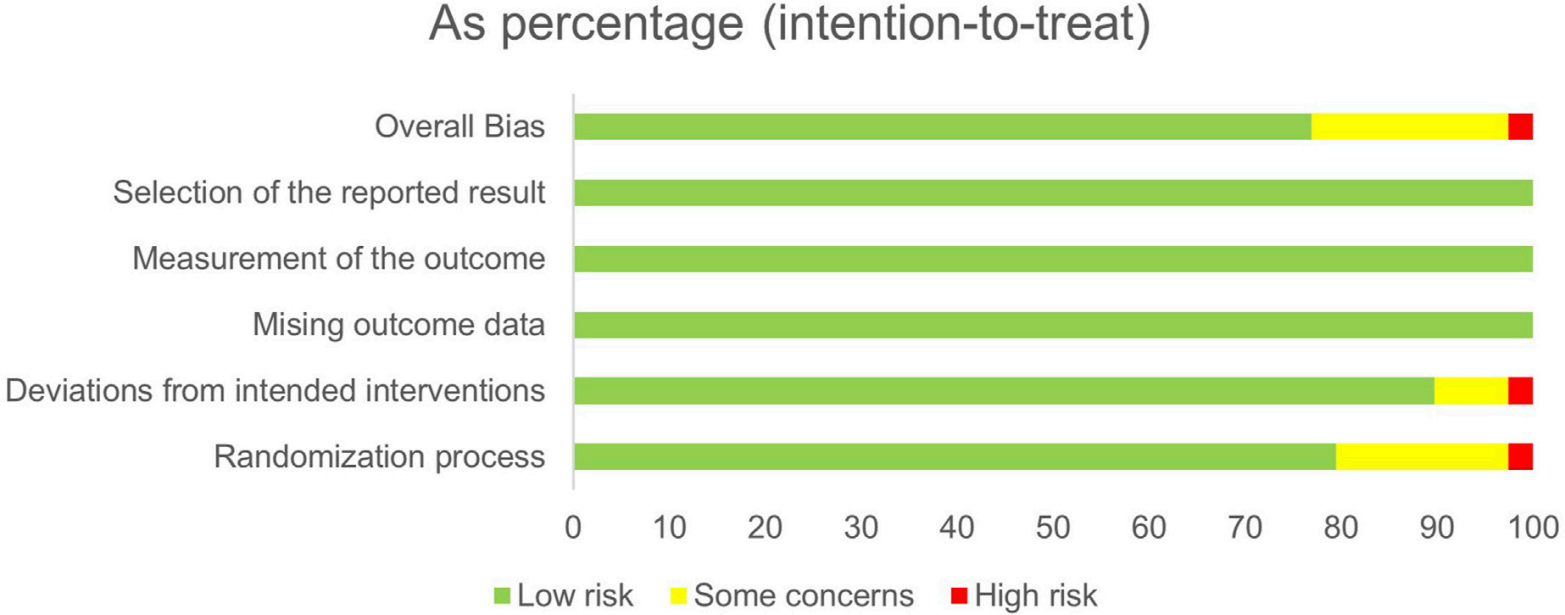
Risks of bias.

The RoB 2.0 was employed to evaluate the risk of bias in the 39 included studies, among which one study was classified as “high risk”, eight studies were categorized as having “some concerns”, and the remaining thirty studies were deemed to have a “low risk”. The distribution of each category is presented in Figure 3, while the quality assessment for each individual study can be found in Supplementary Appendix 4.

### 3.3 Evaluation of statistical inconsistency

The consistency test results show that the network has good consistency (P > 0.05), and the loop-specific method did not suggest any inconsistency between closed loops. In addition, the node-segmentation approach did not suggest statistical inconsistency for any outcome (Supplementary Appendix 5).

### 3.4 Outcomes

#### 3.4.1 Glycosylated hemoglobin (HbA1c)

**FIGURE 4:**
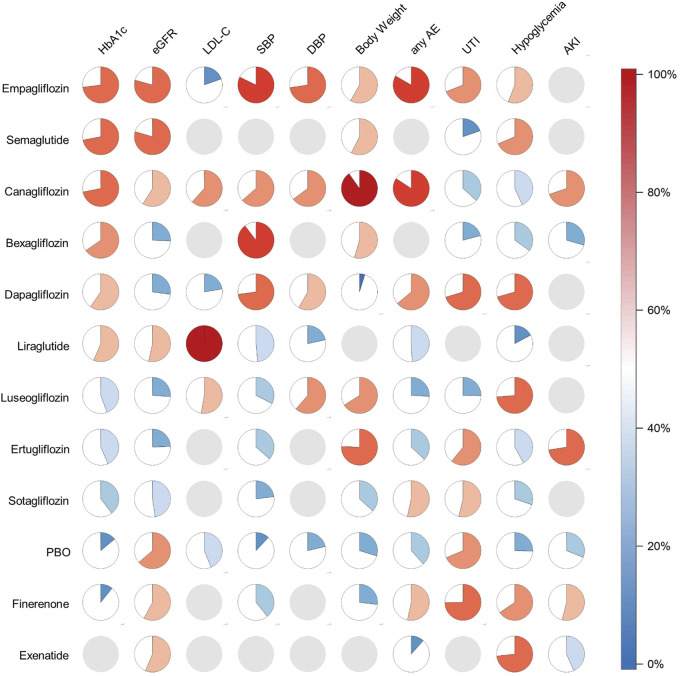
Pie charts of SUCRA value. Filling proportion and color: SUCRA value. Gray pie: not measured.

A total of 24 trials (37,252 patients) evaluated HbA1c (Yale et al., 2013; Chen, 2016; Gao, 2022; Barnett et al., 2014; Yamout et al., 2014; Cherney et al., 2016; Cherney et al., 2023; Curovic et al., 2022; Takashima et al., 2018; Sivalingam et al., 2024; Pollock et al., 2019; Fioretto et al., 2018; Kohan et al., 2014; Allegretti et al., 2019; Haneda et al., 2016; Wada et al., 2022a; George et al., 2018; Dagogo-Jack et al., 2021; Cherney et al., 2021; Pitt et al., 2021; Bakris George et al., 2020; Wanner et al., 2018; Perkovic et al., 2019; Wada et al., 2022b). Our NMA showed that Empagliflozin (MD = −0.33; 95%CI: −0.45, −0.22) and Canagliflozin (MD = −0.33; 95%CI: −0.52, −0.15) significantly reduced HbA1c compared to PBO group. The results of pairwise comparison showed that Empagliflozin (MD = −0.38; 95%CI: −0.62, −0.14) and Canagliflozin (MD = −0.38; 95%CI: −0.65, −0.10) were better than Finerenone (Supplementary Appendix 6a). Results from SUCRA showed that Empagliflozin was the most effective medicine for lowering HbA1c (SUCRA 73%), followed by Semaglutide (SUCRA 72%) and Canagliflozin (SUCRA 71.8%). Ertuglilozin was ranked 8th (SUCRA 43.4%) and placebo 10th (SUCRA 14.0%) (Figure 4; Supplementary Appendix 7b).

#### 3.4.2 Estimate glomerular filtration rate (eGFR)

27 articles involving 32,360 participants assessed eGFR changes (Yale et al., 2013; Chen, 2016; Gao, 2022; Barnett et al., 2014; Yamout et al., 2014; Cherney et al., 2016; Cherney et al., 2023; Curovic et al., 2022; Takashima et al., 2018; Sivalingam et al., 2024; Pollock et al., 2019; Cristian et al., 2020; Fioretto et al., 2018; Kohan et al., 2014; Allegretti et al., 2019; Haneda et al., 2016; Sarafidis et al., 2023; Wada et al., 2022a; Bakris G. L. et al., 2020; George et al., 2018; Dagogo-Jack et al., 2021; Cherney et al., 2021; Bakris et al., 2020; Filippatos et al., 2021; Bakris et al., 2015; Perkovic et al., 2019; Wada et al., 2022a). There was no significant difference in pairwise comparison between drugs compared with PBO group (Supplementary Appendix 6b in the Supplement). Ranked according to the efficacy of SUCRA against all medicines, Semaglutide had the least damage to kidney function (SUCRA 79.5%), followed by Empagliflozin (SUCRA 79.3%). Dapagliflozin, Luseogliflozin, Bexagliflozin and Ertugliflozin (SUCRA from 24.4% to 27.2%) had the greatest impact on renal function (Figure 4; Supplementary Appendix 8b).

#### 3.4.3 Low-density lipoprotein cholesterol (LDL-C)

Eight studies involving 4,876 participants assessed LDL-C changes (Yale et al., 2013; Chen, 2016; Barnett et al., 2014; Yamout et al., 2014; Curovic et al., 2022; Pollock et al., 2019; Haneda et al., 2016; Wanner et al., 2018). Liraglutide was superior to Canagliflozin (MD = −1.45; 95%CI: −1.87, −1.04), Luseoglifozin (MD = −1.41; 95%CI: −2.01, −0.81), PBO (MD = −1.50; 95%CI: −1.89, −1.11), Dapagliflozin (MD = −1.58; 95%CI: −2.05, −1.10), and Empagliflozin (MD = −1.56; 95%CI: −1.96, −1.15) (Supplementary Appendix 6c) and was also the best LDL-C-lowering agent (SUCRA 100%) (Figure 4; Supplementary Appendix 9b).

#### 3.4.4 Blood pressure (BP)

26 articles (56,627 participants) reported the changes in SBP (Yale et al., 2013; Chen, 2016; Barnett et al., 2014; Yamout et al., 2014; Cherney et al., 2016; Cherney et al., 2023; Curovic et al., 2022; Takashima et al., 2018; Filippatos et al., 2021; Agarwal et al., 2023; Wanner et al., 2018; Perkovic et al., 2019; Wada et al., 2022b; Agarwal et al., 2022a; Pollock et al., 2019; Koya et al., 2023; Fioretto et al., 2018; Kohan et al., 2014; Allegretti et al., 2019; Haneda et al., 2016; Sarafidis et al., 2023; Wada et al., 2022b; Dagogo-Jack et al., 2021; Cherney et al., 2021; Pitt et al., 2021; Bakris et al., 2020). Compared to the PBO group, Bexagliflozin (MD = −5.52; 95%CI: −8.80, −2.24), Empagliflozin (MD = −4.33; 95%CI: −5.13, −3.53), Dapagliflozin (MD = −3.79; 95%CI: −5.91, −1.66), Canagliflozin (MD = −3.16; 95%CI: −4.56, −1.75), Finerenone (MD = −1.65; 95%CI: −2.48, −0.81) and Ertugliflozin (MD = −1.50; 95%CI: −2.78, −0.23) significantly reduced SBP. Bexagliflozin and Empagliflozin were significantly superior to Finerenone (MD = −3.87; 95%CI: −7.26, −0.49) (MD = −2.69; 95%CI: −3.78, −1.59), Ertugliflozin (MD = −4.01, 95%CI: −7.53, −0.50) (MD = −2.83; 95%CI: −4.33, −1.32) and Sotagliflozin (MD = −4.98; 95%CI: −9.10, −0.87) (MD = −3.80; 95%CI: −6.41, −1.19) (Supplementary Appendix 6d). We analysed 11 articles with 11,497 participants about DBP (Yale et al., 2013; Chen, 2016; Barnett et al., 2014; Cherney et al., 2016; Curovic et al., 2022; Takashima et al., 2018; Kohan et al., 2014; Haneda et al., 2016; Wada et al., 2022a; Perkovic et al., 2019; Wada et al., 2022b). Compared to PBO group, Empagliflozin (MD = −1.86; 95%CI: −3.18, −40.54) significantly reduced DBP (Supplementary Appendix 6e). The SUCRA results showed that Bexagliflozin (SUCRA 89.6%) and Empagliflozin (SUCRA 82.2%) ranked first and second in reducing SBP. Empagliflozin had the best effect on DBP reduction (SUCRA 72.6%). PBO was last in reducing both SBP and DBP (SUCRA 12.1% and 21.4%) (Figure 4; Supplementary Appendix 10b, 11b).

#### 3.4.5 Body weight

A total of 17 trials (27,839 participants) evaluated body weight (Wada et al., 2022a; Barnett et al., 2014; Yamout et al., 2014; Cherney et al., 2016; Cherney et al., 2023; Curovic et al., 2022; Sivalingam et al., 2024; Koya et al., 2023; Fioretto et al., 2018; Kohan et al., 2014; Allegretti et al., 2019; Haneda et al., 2016; Dagogo-Jack et al., 2021; Cherney et al., 2021; Pitt et al., 2021; Bakris et al., 2020; Wanner et al., 2018). Compared to PBO group, Canagliflozin (MD = −3.81, 95%CI: −6.34, −1.27), Ertugliflozin (MD = −2.36, 95%CI: −3.87, −0.84) and Empagliflozin (MD = −1.29, 95%CI: −1.42, −1.16) significantly reduced body weight. The therapeutic effect of Canagliflozin was significantly better than that of Sotagliflozin (MD = −3.55, 95%CI: −6.75, −0.34), Finerenone (MD = −3.94, 95%CI: −6.73, −1.16), and Dapagliflozin (MD = −6.44, 95%CI: −10.52, −2.37). The therapeutic efficacy of Ertugliflozin (MD = −2.50; 95%CI: −4.40, −0.59) (MD = −4.99; 95%CI: −8.53, −1.46) and Empagliflozin (MD = −1.43; 95%CI: −2.58, −0.28) (MD = −3.93; 95%CI: −7.12, −0.74) was significantly superior to that of Finerenone and Dapagliflozin (Supplementary Appendix 6f). When analyzed in combination with SUCRA, Canagliflozin (SUCRA 90.2%) had the best treatment effect. The least effective for weight loss was Dapagliflozin (SUCRA 4.8%) (Figure 4; Supplementary Appendix 12b).

#### 3.4.6 Adverse events

We analyzed any AE, UTI, Hypoglycemia and AKI.

A total of 31 articles involving 92,867 subjects were included to evaluate the occurrence of any AE (Yale et al., 2013; Gao, 2022; Barnett et al., 2014; Rosas et al., 2023; Yamout et al., 2014; Cherney et al., 2016; Pollock et al., 2019; Koya et al., 2023; Cristian et al., 2020; Fioretto et al., 2018; Kohan et al., 2014; Haneda et al., 2016; Sarafidis et al., 2023; Mahaffey et al., 2019; Wada et al., 2022a; Bakris et al., 2020; George et al., 2018; Dagogo-Jack et al., 2021; Cherney et al., 2021; Bhatt et al., 2021; Pitt et al., 2021; Bakris et al., 2020; Filippatos et al., 2021; Agarwal et al., 2023; Bakris et al., 2015; Perkovic et al., 2019; Agarwal, et al., 2022b; Wada et al., 2022a; Zhang et al., 2023; Agarwal et al., 2022a; Perakakis et al., 2024). Compared to PBO group, Exenatide (OR = 0.79; 95%CI: 0.66, 0.95) showed greater risk. Canagliflozin was safer than PBO group, Sotagliflozin, Finerenone and Exenatide (OR from 1.16 to 1.51; 95%CI from 1.04 to 1.83). Compared with Exenatide, Sotagliflozin, Finerenone and Empagliflozin showed better safety (OR from 0.77 to 1.54; 95%CI from 0.63 to 2.09) (Supplementary Appendix 6g). The SUCRA analysis indicated that Canagliflozin was probably the drug with the best safety profile (SUCRA 84.2%), followed by Empagliflozin (SUCRA 83.3%). Exenatide had the worst safety profile (SUCRA 11.6%) (Figure 4; Supplementary Appendix 13b).

A total of 25 trials (67,632 participants) evaluated UTI (Yale et al., 2013; Barnett et al., 2014; Yamout et al., 2014; Cherney et al., 2016; Cherney et al., 2023; Sivalingam et al., 2024; Pollock et al., 2019; Cristian et al., 2020; Fioretto et al., 2018; Kohan et al., 2014; Allegretti et al., 2019; Haneda et al., 2016; Tuttle et al., 2022; Mahaffey et al., 2019; Wada et al., 2022b; George et al., 2018; Dagogo-Jack et al., 2021; Bhatt et al., 2021; Pitt et al., 2021; Bakris et al., 2020; Filippatos et al., 2021; Perkovic et al., 2019; Wada et al., 2022a; Agarwal et al., 2022b; Cherney et al., 2021) (Supplementary Appendix 6h in the Supplement). In terms of the occurrence of UTI, we found that Canagliflozin seemed to exhibit a worse safety profile compared with PBO group (OR = 0.89; 95%CI: 0.80, 0.99). There were no significant differences between other drugs in pairwise comparisons. Finerenone was the safest in the occurrence of UTI (SUCRA 74.8%), followed by Dapagliflozin and Empagliflozin (SUCRA 70.3% and 69.0%). We also found that Semaglutide (SUCRA 19.7%) was probably the most likely treatment to cause UTI to occur (Figure 4; Supplementary Appendix 14b).

The occurrence of Hypoglycemia was assessed in 24 studies involving 65,498 participants (Chen, 2016; Gao, 2022; Barnett et al., 2014; Cherney et al., 2016; Cherney et al., 2023; Sivalingam et al., 2024; Pollock et al., 2019; Koya et al., 2023; Cristian et al., 2020; Fioretto et al., 2018; Kohan et al., 2014; Allegretti et al., 2019; Haneda et al., 2016; Tuttle et al., 2022; Mahaffey et al., 2019; Wada et al., 2022b; Dagogo-Jack et al., 2021; Cherney et al., 2021; Bhatt et al., 2021; Pitt et al., 2021; Bakris et al., 2020; Filippatos et al., 2021; Perkovic et al., 2019; Agarwal et al., 2022a). (Supplementary Appendix 6i). Finerenone (OR = 1.18; 95%CI: 1.07, 1.31), Empagliflozin (OR = 1.13; 95%CI: 1.01, 1.27) were better than PBO group in reducing the incidence of Hypoglycemia. There were no significant difference between other drugs. In our analysis, Finerenone (SUCRA 65.5%) had a better safety than Empagliflozin (SUCRA 55.8%). Liraglutide (SUCRA 17.2%) may be a risk factor for Hypoglycemia. The safety of Luseogliflozin (SUCRA 73.7%) was probably optimal (Figure 4; Supplementary Appendix 15b).

60,930 participants involved in 15 articles were assessed for the occurrence of AKI (Koya et al., 2023; Cristian et al., 2020; Allegretti et al., 2019; Sarafidis et al., 2023; Mahaffey et al., 2019; Bakris et al., 2020; Dagogo-Jack et al., 2021; Pitt et al., 2021; Bakris et al., 2020; Filippatos et al., 2021; Perkovic et al., 2019; Agarwal et al., 2022b; Wada et al., 2022a; Zhang et al., 2023; Agarwal et al., 2022a). There were no significant difference in pairwise comparison between drugs compared with PBO group (Supplementary Appendix 6j in the Supplement). Combined with SUCRA analysis, Ertugliflozin was the medicines with the fewest AKI (SUCRA 72.5%), significantly better than Canagliflozin (SUCRA 69.7%), which ranked second. Bexagliflozin had the highest likelihood of AKI (SUCRA 29.4%) (Figure 4; Supplementary Appendix 16b).

### 3.5 Additional analyses

Assessment of outcome measures showed that 100% of the evidence was rated as low or very low (Supplementary Appendix 20). The funnel plot and Egger’s test indicated publication bias for eGFR (P = 0.007) (Supplementary Appendix 17). Heterogeneity was observed in HbA1c, body weight, and eGFR. Subgroup analyses suggested that study country, medication timing, numbers of participants and drug category may contribute to this heterogeneity (Supplementary Appendix 21).

## 4 Discussion

### 4.1 Principal findings

SGLT-2 inhibitors, GLP-1 receptor agonists, and Finereone are recommended as preferred pharmacotherapies for T2DM patients with CKD; however, the guidelines do not specify a clear hierarchy of preference among these options (Chinese Diabetes Society, 2025; American Diabetes Association Professional Practice Committee, 2023). Given the widespread clinical application of these drugs, we assert that comparing their clinical efficacy holds significant importance. Therefore, in the absence of direct comparative evidence, we conducted an indirect comparison to determine the clinical efficacy and safety of SGLT-2 inhibitors, GLP-1 receptor agonists and Finerenone in patients with T2DM and non-dialysis CKD. The study has identified several significant findings. The drug Semaglutide (a GLP-1 receptor agonist) appears to be the optimal choice among all drug classes for patients with high HbA1c and poor eGFR, when compared to the other two drugs. The efficacy of SGLT-2 inhibitors and GLP-1 receptor agonists in reducing body weight and HbA1c surpasses that of Finerenone. Canagliflozin, Empagliflozin (SGLT-2 inhibitors), and Semaglutide are considered the most suitable medications for patients with high body weight and elevated HbA1c levels, specially Canagliflozin. Specifically, Empagliflozin, Dapagliflozin and Canagliflozin (SGLT-2 inhibitors) demonstrate remarkable effectiveness in managing high SBP. The use of Empagliflozin and Canagliflozin may be considered for patients presenting with high DBP as the primary accompanying symptom. Liraglutide (a GLP-1 receptor agonist) may be prioritized for patients with elevated LDL-C levels. Although there is no obvious advantage in reducing the indicators and related risk factors in patients with T2DM with non-dialysis CKD, Finerenone has less damage to eGFR, and has a convincing reduction in the occurrence of Hypoglycemia events, and the incidence of adverse events is low, so it can still be used as the first choice for some patients.

The efficacy and safety of these drugs have been extensively deliberated. In an NMA comprising 816 randomized controlled trials with 471,038 participants, SGLT-2 inhibitors, GLP-1 receptor agonists, and Finerenone exhibited favorable outcomes in terms of reducing all-cause mortality and enhancing cardiorenal results among patients diagnosed with T2DM (Shi et al., 2023). 3 studies confirmed that SGLT-2 inhibitors have a positive effect on cardio-renal outcomes in T2DM patients with CKD compared with GLP-1 receptor agonists or Finerenone (Yamada et al., 2021; Nguyen et al., 2023; Apperloo et al., 2024). However, these studies either focused solely on T2DM patients or had a limited number of RCTs and study indicators, leaving the impact of SGLT-2 inhibitors, GLP-1 receptor agonists, and Finerenone on non-dialysis CKD patients with T2DM unclear. Therefore, we designed an NMA to evaluate the effects of several drugs on various clinically accessible indicators.

Benefits of most SGLT-2 inhibitors include renal safety, reduction in HbA1c, blood pressure, and body weight. Hyperglycemia is linked to an increase in eGFR as compensation (Thomas, 2014). Hypertension and overweight increase the risk of T2DM. Therefore, the optimal antidiabetic drug should not only have good glucose-lowering ability, but also be beneficial for body weight, blood pressure, and renal function (Leehey et al., 2015; Garvey, 2022; American Diabetes Association, 2020). The renal protective mechanism of SGLT-2 inhibitors may be dominated by direct effects on renal vessels. Empagliflozin decreases proximal tubular sodium reabsorption, increases distal sodium delivery to the macula densa, and activates glomerular feedback. This process reduces hyperfiltration and regulates vascularization (Wanner, 2017). In addition, SGLT-2 inhibitors can directly act on the kidney, reduce renal fibrosis by inhibiting oxidative stress in the kidney, (Woods et al., 2019), attenuate the increase of angiotensinogen, and reduce NLRP3 inflammasome activity (Yaribeygi et al., 2019), these mechanisms may explain how SGLT-2 inhibitors reduce blood pressure. The short-term use of SGLT-2 inhibitors may temporarily decrease eGFR, but it will gradually recover, indicating a long-term protective effect on eGFR (Barnett et al., 2014). This may be due to reduced uric acid, inhibited inflammatory response, and decreased vascular stiffness (Heerspink et al., 2016; Chilton et al., 2015). Although previous studies have suggested that angiotensin converting enzyme inhibitors (ACEI) or angiotensin II receptor blocker (ARB) are the preferred antihypertensive drugs for T2DM patients with CKD, they do not significantly reduce blood glucose levels and their effects remain limited (Bakris et al., 2000). The potential of using SGLT-2 inhibitors as the first choice after ACEI and ARB drugs, considering its high cardio-renal safety and hypoglycemic function, needs further discussion.

Unexpectedly, we found Canagliflozin, an SGLT-2 inhibitor agent, to be the most effective for weight reduction (SUCRA = 90.2%), superior to GLP-1 receptor agonists. The reason for this may be due to different principles of action. The hormone GLP-1, produced in cells lining the intestines, slows down digestion and reduces food intake by inhibiting neural activity in the brain (Drucker, 2016). SGLT-2 inhibitors can also lead to weight loss depending on the dosage, as excess glucose is eliminated from the body (Brown et al., 2019). At the same time, it can also accelerate fat burning by lipolysis and fatty acid oxidation (Vallon et al., 2017). Therefore, it can be hypothesized that the progressive increase in blood glucose levels and concurrent decrease in body weight may account for the superior weight reduction observed with SGLT-2 inhibitors compared to GLP-1 receptor agonists.

GLP-1 receptor agonists, especially Semaglutide and Liraglutide, showed the greatest advantage in reducing LDL-C (SUCRA = 100%) and HbA1c (SUCRA = 72.0% and 56.4%), and had a higher safety profile against eGFR (SUCRA = 79.5% and 53.4%). The management of LDL-C is crucial in preventing cardiovascular events caused by atherosclerotic plaque formation (Giglio et al., 2021). The existing NMA highlights the remarkable cardiovascular benefits of GLP-1 receptor agonists (Zhang Y. et al., 2022). However, our study suggests that these advantages may be attributed to specific pathways that lower LDL-C and reduce lipid deposition in the cardiovascular system. Nevertheless, further testing is required to confirm these pathways.

Finerenone is a novel non-steroidal mineralocorticoid receptor antagonist (MRA), and its safety in cardiorenal prognosis has been proved (Agarwal et al., 2022b; Zhang M-Z. et al., 2022), this may be due to the reduction of proteinuria and tissue inflammation and fibrosis (Wish and Pablo, 2022). Our study confirmed Finerenone’s safety and highlighted its significant advantage in reducing SBP, while showing no notable effect on blood glucose reduction. The combination of Finerenone with SGLT-2 inhibitors or GLP-1 receptor agonists have been suggested to enhance anti-inflammatory, anti-oxidative stress, and endothelial protection effects (Lv et al., 2023). At the same time, SGLT-2 inhibitors inhibit sodium reabsorption, while Finerenone promotes sodium retention (Lv et al., 2023; Kidokoro et al., 2019). Whether the pathways of SGLT-2 inhibitors and Finerenone partially coincide, lead to water and salt metabolism disorders, and increase additional adverse reactions still needs more clinical trials to prove its safety.

Certain limitations should be noted when interpreting our study. The results of our study indicate a higher risk of urinary tract infections in patients treated with GLP-1 receptor agonists compared to those receiving SGLT-2 inhibitors therapy, contradicting previous research findings (Tanrıverdi et al., 2023; Drucker, 2016). The effect of GLP-1 receptor agonists on UTI occurrence was only reported in one literature, which included a small number of patients and specified clear inclusion criteria as “T2DM and albuminuria” (Sivalingam et al., 2024). The presence of proteinuria may influence UTI incidence. The eGFR safety profile of most GLP-1 receptor agonists are good, except for Exenatide. From April 2005 to August 2008, 78 cases of renal disease caused by Exenatide were reported to the FDA. Gastrointestinal adverse reactions are the most common side effects of GLP-1 receptor agonists, which can cause significant fluid loss and pre-renal acute failure (Bzowyckyj, 2020). At the same time, GLP-1 receptor agonists can simultaneously enhance renal sodium efficacy, leading to renal hypoperfusion and AKI development (Skov et al., 2013). Interestingly, our study does not support a higher likelihood of Exenatide causing eGFR damage (SUCRA = 56.4%), possibly due to its ability to reduce renal pathological material deposition. Animal experiments have demonstrated that Exenatide reduces inflammatory and apoptotic cell infiltration in the glomerulus of mice, as well as lipid content (Park et al., 2007). Activation of the GLP-1 receptor stimulates adenylyl cyclase, leading to increased cAMP (a key mediator of GLP-1-induced insulin secretion) production (Fujita et al., 2014). In conclusion, Exenatide remains a controversial drug and should be minimized in patients with severe renal insufficiency.

### 4.2 Strengths and limitations

The advantages of this network meta-analysis are as follows: first, we included a large number of literatures with a large sample size and reliable data; secondly, on top of the existing analysis, we provided additional evidence to specifically analyze the advantages and disadvantages of different SGLT-2 inhibitors and GLP-1 receptor agonists and Finerenone in the treatment of T2DM patients with non-dialysis CKD to facilitate clinical decision making by physicians. Last but not least, the ADA recommends that individuals with T2DM and CKD should use SGLT-2 inhibitors or GLP-1 receptor agonists as their first choice, but it does not specify which medicine to use for patients with different clinical priorities (Committee, 2024). Our study identified the efficacy of both drugs in improving HbA1c and eGFR, while also providing more favorable evidence regarding the benefits and limitations of various drugs in other indicators such as LDL-C, SBP, DBP, as well as the safety profile of Finerenone.

Limitations of our NMA are largely driven by the available evidence. Firstly, it is acknowledged that the heterogeneity and inherent bias within the literature are objective realities, which may potentially compromise the accuracy of research outcomes. For instance, several included trials lacked baseline data, and variations existed in population characteristics, duration of pharmacological treatment, and follow-up periods. This may have led to some bias. Third, the dose of the study drug and the level of detail in our study were not considered. Finally, the literatures included in this paper provide only indirect comparisons between drugs, lacking direct comparison evidence, which affects the credibility of the results. More detailed studies are needed to supplement this aspect in the future.

## 5 Conclusion

In conclusion, this network meta-analysis provides compelling evidence regarding the effects of SGLT-2 inhibitors, GLP-1 receptor agonists, and Finerenone on patients with T2DM complicated by non-dialysis CKD. Based on robust evidence from indirect comparisons, the safety profile of Finerenone in patients with T2DM and non-dialysis. CKD outweighs its limited efficacy in improving HbA1c levels, while demonstrating an added advantage of reducing SBP. The GLP-1 receptor agonists are beneficial for T2DM patients with non-dialysis CKD, effectively reducing HbA1c, LDL-C, and body weight without significantly impacting renal function, expect Exenatide. The most recommended treatment for patients with T2DM and non-dialysis CKD, along with high levels of HbA1c, SBP, and DBP, may be Empagliflozin and Canagliflozin, as they have a lesser impact on eGFR. Dapagliflozin demonstrated lower efficacy in reducing HbA1c compared to the aforementioned medications; however, its effect on body weight reduction was less significant, and it had a lower likelihood of causing Hypoglycemia. Except for Ertugliflozin, Luseogliflozin, and Sotagliflozin, other SGLT-2 inhibitors medications demonstrated a superior impact on reducing SBP compared to Finerenone; however, they also entailed an elevated risk of UTI. Our NMA enriches the existing body of research by providing substantial evidence to assess the benefits and risks associated with various drugs. In summary, it is imperative to consider the potential adverse effects of these treatments when formulating personalized treatment plans for individual patients.

## Data Availability

The original contributions presented in the study are included in the article/Supplementary Material, further inquiries can be directed to the corresponding author.
