# Supplementary material for: Clinical efficacy and safety of sodium-glucose cotransporter protein-2 (SGLT-2) inhibitor, glucagon-like peptide-1 (GLP-1) receptor agonist, and Finerenone in type 2 diabetes mellitus with non-dialysis chronic kidney disease: a network meta-analysis of randomized clinical trials
Source: Front Pharmacol. 2025 Mar 27;16:1517272. doi: 10.3389/fphar.2025.1517272 (PMC11983143; doi:10.3389/fphar.2025.1517272)
Supplement: Supplementary file 2 [file DataSheet1.docx]

**Clinical efficacy and safety of SGLT-2i, GLP-1 RA, and Finerenone in type 2 diabetes mellitus with non-dialysis chronic kidney disease: A network meta-analysis of randomized clinical trials**

**Supplementary Material**

**Table of contents Page**

Appendix 1 Protocol 2

Appendix 2 Search strategy 7

Appendix 3 Network Plots 10

Appendix 4 Risk of bias in included studies 20

Appendix 5 Assessments of Inconsistencies 104

Appendix 6 League Table of each outcome 110

Appendix 7 HbA1c Outcome: Results 118

Appendix 8 eGFR Outcome: Results 120

Appendix 9 LDL-C Outcome: Results 122

Appendix 10 SBP Outcome: Results 124

Appendix 11 DBP Outcome: Results 126

Appendix 12 Body Weight Outcome: Results 128

Appendix 13 Any AE Outcome: Results 130

Appendix 14 UTI Outcome: Results 132

Appendix 15 Hypoglycemia Outcome: Results 134

Appendix 16 AKI Outcome: Results 136

Appendix 17 Funnel plots of each outcome indicator and Egger’s test 138

Appendix 18 Included studies 149

Appendix 19 References 155

Appendix 20 Grading the evidence for outcome of the network meta-analysis using CINeMA 164

Appendix 21 Subgroup Analysis 201

**Appendix 1 Protocol**

**Clinical efficacy and safety of SGLT-2i, GLP-1 RA, and Finerenone in type 2 diabetes mellitus with non-dialysis chronic kidney disease: A network meta-analysis of randomized clinical trials**

**Jingyi Guo, Maoying Wei, Wenhua Zhang, Yijia Jiang, Aijing Li, Churan Wang, Dan Yin, Anning Sun, Yanbing Gong*.**

**Correspondence to**

Yanbing Gong

gyb_1226@163.com

**Abstract**

**Objective:** To investigate the safety and clinical efficacy of sodium-glucose cotransporter protein-2 (SGLT-2) inhibitors, glucagon-like peptide-1 (GLP-1) receptor agonists and Finerenone in treating patients with type 2 diabetes mellitus (T2DM) combined with non-dialysis chronic kidney disease (CKD).

**Methods:** Cochrane Library, PubMed, EMBASE, Web of Science, CNKI, CQVIP database, and WanFang from their inception up to November 2023 were searched to compare the efficacy and safety of SGLT-2i, GLP-1 RA and Finerenone in the treatment of T2DM patients with non-dialysate CKD. To assess the methodological quality and risk of bias in the included studies, we utilized the Cochrane Risk of Bias Assessment tool (RoB 2.0). The confidence of evidence was examined using Confidence in Network Meta-Analysis (CINeMA). Traditional meta-analysis of variables was conducted using Stata 17.0 software with a random-effects model. We assessed publication bias using funnel plots and explored potential sources of heterogeneity through subgroup analysis.

**Results:** A total of 39 studies (99599 patients) were included. Compared to Placebo (PBO), SGLT-2 inhibitors demonstrated superior efficacy in reducing glycosylated hemoglobin (HbA1c) (MD = −0.33; 95%CI: from −0.52 to −0.15), systolic blood pressure (SBP) (MD from −5.52 to −1.50; 95%CI from −8.80 to −0.23), body weight (MD from −3.81 to −1.29; 95%CI from −6.34 to −1.42) and diastolic blood pressure (DBP) (MD = −1.86; 95%CI: −3.18, −40.54). The efficacy of Liraglutide in reducing Low-Density Lipoprotein Cholesterol (LDL-C) surpasses that of other agents (MD from −1.58 to −1.41; 95%CI from −2.01 to −0.81). Finerenone significantly reduce SBP (MD = −1.65; 95%CI: −2.48, −1.04) compare to PBO. According to the SUCRA based relative ranking of treatments, Empagliflozin was most effective in reducing HbA1c and DBP. Semaglutide was least harmful to estimated glomerular filtration rate. Liraglutide was the most effective in reducing LDL-C. Bexagliflozin, Canagliflozin were most effective in reducing SBP and body weight. Finerenone had the lowest incidence of urinary tract infection, Hypoglycemia was lowest in the Luseogliflozin group. Ertugliflozin was least likely to cause acute kidney injury. Canagliflozin had the lowest probability of any adverse event.

**Conclusion:** The safety of these drugs has been confirmed, except for some special drugs. SGLT-2 inhibitors have a preferential glucose-lowering and weight-loss function, GLP-1 receptor agonists have a preferential lowering of LDL-C and blood glucose, and Finereone significantly reduces SBP compared with PBO.

**Systematic Review Registration:** PROSPERO, CRD42024571544.

**KEYWORDS:** SGLT-2i. GLP-1 RA, Finerenone, type 2 diabetes mellitus, chronic kidney disease, network meta-analysis.

**1 Introduction**

Diabetes is a global public health concern, and with the recent surge in diabetes patients, it is projected to impact 784 million individuals by 2045, posing a significant threat to human well-being. Diabetic kidney disease is a prominent microvascular complication of diabetes mellitus, with an estimated 40% of individuals with diabetes projected to develop chronic kidney disease (CKD) during their lifetime, potentially necessitating the need for renal replacement therapy (Afkarian et al., 2016; Scilletta et al., 2023; Zoja et al., 2020). Hence, it is imperative to prevent further progression of kidney disease in the management of type 2 diabetes (T2DM) patients with CKD.

The SGLT-2 inhibitors represent a novel class of oral hypoglycemic medications. GLP-1 receptor agonists reduce Glycosylated hemoglobin (HbA1c) by stimulating insulin secretion and reducing glucagon secretion, while also decreasing appetite through delayed gastric emptying (T. D. Filippatos and Elisaf, 2013). Several randomized controlled trials (RCTs) have demonstrated the effectiveness and safety of these two drugs (Perkovic et al., 2019; Mann et al., 2020). Finerenone, a nonsteroidal selective mineralocorticoid receptor antagonist, has shown in large RCTs to slow down CKD progression and improve cardiovascular outcomes (Pitt et al., 2021; G. Filippatos et al., 2021; G. L. Bakris et al., 2020). It was approved by the Food and Drug Administration (FDA) in July 2021 for treating T2DM in CKD patients. Although GLP-1 receptor agonists1 have been found to lower blood pressure and body weight while improving cardiovascular outcomes, there is still no clear conclusion when compared to SGLT-2 inhibitors and Finerenone for treating T2DM in non-dialysis CKD (Sun et al., 2015; Shah and Vella, 2014).

There is currently a lack of comprehensive evaluation of the efficacy and safety of several drugs for treating T2DM combined with CKD. Network meta-analysis (NMA) combines direct and indirect evidence to compare multiple treatments and assess their interrelationship. Our study focused on non-dialytic CKD patients (eGFR>15mL/min/1.73m2) as these drugs are not recommended for patients with low eGFR (Chinese Diabetes Society 2025; Committee 2023). Therefore, we conducted an NMA of RCTs to assess the clinical efficacy and safety of SGLT-2 inhibitors, GLP-1 receptor agonists, and Finereone in non-dialysis CKD patients with T2DM.

**Method**

We will conduct a systematic review and network meta-analysis.

**Eligibility criteria**

This trial included double-blind RCTs comparing SGLT-2i, GLP-1 RA, and Finerenone or directly with placebo in adults with T2DM and non-dialysis CKD. Studies using other control drugs, studies with repeated publications and incomplete data, studies with eGFR<15mL/min/1.73m^2^, studies published in languages other than Chinese or English, and studies using the drug within 3 months before screening were excluded.

**Data sources and searches**

We will search seven large electronic databases (Cochrane Library, PubMed, EMBASE, Web of Science, CNKI, CQVIP database, and WanFang data) using predefined keywords to identify SGLT-2i, GLP-1 RA, Finerenone or placebo-controlled randomized trials in patients with T2DM and non-dialysis CKD. The searches will be limited to human studies conducted in English or Chinese languages. Two independent investigators will review the titles and abstracts of all citations identified during the search.

**Study selection**

Full-text articles will retrieve for the included abstracts and will subsequently screen for eligibility (according to the aforementioned inclusion criteria) by two independent investigators (G and J). Disagreements at this level will resolve by consensus and a third reviewer if needed (Z).

**Data extraction**

Two researchers used standard data extraction tables for information extraction, judgment, and literature extraction information including: study author, publication year, intervention measures, outcomes etc. In case of disagreement, a third researcher (Z) assisted in making a judgment.

**Outcomes**

We will begin by focusing on the patient-important outcomes listed below.

- Glycosylated hemoglobin (HbA1c)
- estimate glomerular filtration rate (eGFR)
- low-density lipoprotein cholesterol (LDL-C)
- systolic blood pressure (SBP)
- diastolic blood pressure (DBP)
- body weight.

We will consider any Adverse Events, Urinary Tract Infections, Hypoglycemia and Acute Kidney Injury as safety outcomes.

- any Adverse Events (any AE)
- Urinary Tract Infections (UTI)
- Hypoglycemia
- Acute Kidney Injury (AKI)

**Risk of Bias Assessment**

The risk of bias assessment was conducted by 2 researchers (G and J) using the Cochrane Risk of Bias Assessment tool (RoB 2.0) (Sterne et al., 2019). Each study was classified as having low, some concerns or high risk of bias.

**Statistical analysis**

For outcome indicators, odds ratio (OR) was used for bicategorical variables, mean difference (MD) was used for continuous variables, and 95% confidence interval (95% CI) was used to represent statistical results. The findings were considered statistically significant if the 95%CI did not include the null value (0 for MD and 1 for OR). For each result were calculated using a random effects model. Bilateral P-values<0.05 were considered statistically significant. We evaluated the between-study heterogeneity using the I2 statistic and its associated p-values. Specifically, I2 values of 25%, 50%, and 75% were indicative of low, moderate, and high levels of statistical heterogeneity, respectively. Subsequently, subgroup analyses were conducted to investigate potential sources of this heterogeneity. STATA 17.0 was used for statistical analysis, evidence network and surface under the cumulative raking curve (SUCRA). We evaluated publication bias of articles using funnel plots and Egger's test. We examined the confidence of evidence using the CINeMA (Salanti et al. 2014) web application, which grades the confidence of the results as high, moderate, low, and very low.

**Ethical issues**

Ethical approval and patient consent are not required since this is a network meta-analysis based on published studies.

**Publication**

The papers will be published in a traditional format for systematic reviews and network meta-analyses.

**Acknowledgments**

The authors thank Dongzhimen Hospital and Beijing University of Chinese Medicine for its support of this work and the reviewers for allowing the authors to improve the manuscript.

Appendix 2. Search strategy

PUBMED

((((((((((("Sodium-Glucose Transporter 2"[Mesh]) OR ((SLC5A2 Protein[Title/Abstract]) OR (SGLT2 Protein[Title/Abstract]))) OR (("Canagliflozin"[Mesh]) OR (((Invokana[Title/Abstract]) OR (Canagliflozin Hemihydrate[Title/Abstract])) OR (Canagliflozin, Anhydrous[Title/Abstract])))) OR (("empagliflozin" [Supplementary Concept]) OR (Jardiance[Title/Abstract]))) OR (("dapagliflozin" [Supplementary Concept]) OR ((Farxiga[Title/Abstract]) OR (Forxiga[Title/Abstract])))) OR (Sotagliflozin[Title/Abstract])) OR (("ertugliflozin" [Supplementary Concept]) OR (Steglatro[Title/Abstract]))) OR (("Glucagon-Like Peptide 1"[Mesh]) OR (((Peptide-1 Receptor, Glucagon-Like[Title/Abstract]) OR (GLP1R Protein[Title/Abstract])) OR (GLP 1 Receptor[Title/Abstract])))) OR (("Liraglutide"[Mesh]) OR ((Victoza[Title/Abstract]) OR (Saxenda[Title/Abstract])))) OR (("dulaglutide" [Supplementary Concept]) OR (Trulicity[Title/Abstract]))) OR (("finerenone" [Supplementary Concept]) OR (kerendia[Title/Abstract]))) AND ((("Diabetes Mellitus, Type 2"[Mesh]) OR (((((((((((((Diabetes Mellitus, Noninsulin Dependent[Title/Abstract]) OR (Ketosis-Resistant Diabetes Mellitus[Title/Abstract])) OR (Non-Insulin-Dependent Diabetes Mellitus[Title/Abstract])) OR (Noninsulin Dependent Diabetes Mellitus[Title/Abstract])) OR (Stable Diabetes Mellitus[Title/Abstract])) OR (Diabetes Mellitus, Type II[Title/Abstract])) OR (NIDDM[Title/Abstract])) OR (Maturity Onset Diabetes Mellitus[Title/Abstract])) OR (MODY[Title/Abstract])) OR (Slow-Onset Diabetes Mellitus[Title/Abstract])) OR (Maturity Onset Diabetes[Title/Abstract])) OR (Type 2 Diabetes[Title/Abstract])) OR (Adult-Onset Diabetes Mellitus[Title/Abstract]))) AND (("Renal Insufficiency, Chronic"[Mesh]) OR (((Chronic Renal Insufficiencies[Title/Abstract]) OR (Chronic Kidney Disease[Title/Abstract])) OR (Chronic Renal Disease[Title/Abstract]))))

**EMBASE**

#1 AND #4

#1='Sodium-Glucose Transporter 2':ab,ti OR 'SGLT2 Protein':ab,ti OR 'SLC5A2 Protein':ab,ti OR 'Canagliflozin':ab,ti OR 'Invokana':ab,ti OR 'Canagliflozin Hemihydrate':ab,ti OR 'Canagliflozin, Anhydrous':ab,ti OR 'Empagliflozin':ab,ti OR 'Jardiance':ab,ti OR 'Dapagliflozin':ab,ti OR 'Farxiga':ab,ti OR 'Forxiga':ab,ti OR 'Sotagliflozin':ab,ti OR 'Ertugliflozin':ab,ti OR 'Steglatro':ab,ti OR '[Glucagon-Like Peptide 1](https://www.ncbi.nlm.nih.gov/mesh/68052216)':ab,ti OR 'Peptide-1 Receptor, Glucagon-Like':ab,ti OR 'GLP1R Protein':ab,ti OR 'GLP 1 Receptor':ab,ti OR 'Liraglutide':ab,ti OR 'Victoza':ab,ti OR 'Saxenda':ab,ti OR 'Dulaglutide':ab,ti OR 'Trulicity':ab,ti OR 'Finerenone':ab,ti OR 'kerendia':ab,ti

#2='Diabetes Mellitus, Type 2':ab,ti OR 'Diabetes Mellitus, Noninsulin Dependent':ab,ti OR 'Ketosis-Resistant Diabetes Mellitus':ab,ti OR 'Non-Insulin-Dependent Diabetes Mellitus':ab,ti OR 'Noninsulin Dependent Diabetes Mellitus':ab,ti OR 'Stable Diabetes Mellitus':ab,ti OR 'Diabetes Mellitus, Type II':ab,ti OR 'NIDDM':ab,ti OR 'Maturity Onset Diabetes Mellitus':ab,ti OR 'MODY':ab,ti OR 'Slow-Onset Diabetes Mellitus':ab,ti OR 'Maturity Onset Diabetes':ab,ti OR 'Type 2 Diabetes':ab,ti OR 'Adult-Onset Diabetes Mellitus':ab,ti

#3='Renal Insufficiency, Chronic':ab,ti OR 'Chronic Renal Insufficiencies':ab,ti OR 'Chronic Kidney Disease':ab,ti OR 'Chronic Renal Disease':ab,ti

#4=#2 AND #3

**Web of Science**

TS=(Diabetes Mellitus, Type 2 OR Diabetes Mellitus, Noninsulin Dependent OR Ketosis-Resistant Diabetes Mellitus OR Non-Insulin-Dependent Diabetes Mellitus OR Noninsulin Dependent Diabetes Mellitus OR Stable Diabetes Mellitus OR Diabetes Mellitus, Type II OR NIDDM OR Maturity Onset Diabetes Mellitus OR MODY OR Slow-Onset Diabetes Mellitus OR Maturity Onset Diabetes OR Type 2 Diabetes OR Adult-Onset Diabetes Mellitus) AND TS=(Renal Insufficiency, Chronic OR Chronic Renal Insufficiencies OR Chronic Kidney Disease OR Chronic Renal Disease) AND TS=(Sodium-Glucose Transporter 2 OR SLC5A2 Protein OR SGLT2 Protein OR Canagliflozin OR Invokana OR Canagliflozin Hemihydrate OR Canagliflozin, Anhydrous OR Empagliflozin OR Jardiance OR Dapagliflozin OR Farxiga OR Forxiga OR Sotagliflozin OR Ertugliflozin OR Steglatro OR Glucagon-Like Peptide 1 OR Peptide-1 Receptor, Glucagon-Like OR GLP1R Protein OR GLP 1 Receptor OR Liraglutide OR Victoza OR Saxenda OR Dulaglutide OR Trulicity OR Finerenone OR kerendia)

**Cochrane**

#1 AND #4

#1=(Sodium-Glucose Transporter 2):ab,ti,kw OR (SLC5A2 Protein):ab,ti,kw OR (SGLT2 Protein):ab,ti,kw OR (Canagliflozin):ab,ti,kw OR (Invokana):ab,ti,kw OR (Canagliflozin Hemihydrate):ab,ti,kw OR (Canagliflozin, Anhydrous):ab,ti,kw OR (Empagliflozin):ab,ti,kw OR (Jardiance):ab,ti,kw OR (Dapagliflozin):ab,ti,kw OR (Farxiga):ab,ti,kw OR (Forxiga):ab,ti,kw OR (Sotagliflozin):ab,ti,kw OR (Ertugliflozin):ab,ti,kw OR (Steglatro):ab,ti,kw OR ([Glucagon-Like Peptide 1](https://www.ncbi.nlm.nih.gov/mesh/68052216)):ab,ti,kw OR (Peptide-1 Receptor, Glucagon-Like):ab,ti,kw OR (GLP1R Protein):ab,ti,kw OR (GLP 1 Receptor):ab,ti,kw OR (GLP 1):ab,ti,kw OR (Liraglutide):ab,ti,kw OR (Victoza):ab,ti,kw OR (Saxenda):ab,ti,kw OR (Dulaglutide):ab,ti,kw OR (Trulicity):ab,ti,kw OR (Finerenone):ab,ti,kw OR (kerendia):ab,ti,kw

#2=(Diabetes Mellitus, Type 2):ab,ti,kw OR (Diabetes Mellitus, Noninsulin Dependent):ab,ti,kw OR (Ketosis-Resistant Diabetes Mellitus):ab,ti,kw OR (Non-Insulin-Dependent Diabetes Mellitus):ab,ti,kw OR (Noninsulin Dependent Diabetes Mellitus):ab,ti,kw OR (Stable Diabetes Mellitus):ab,ti,kw OR (Diabetes Mellitus, Type II):ab,ti,kw OR (NIDDM):ab,ti,kw OR (Maturity Onset Diabetes Mellitus):ab,ti,kw OR (MODY):ab,ti,kw OR (Slow-Onset Diabetes Mellitus):ab,ti,kw OR (Maturity Onset Diabetes):ab,ti,kw OR (Type 2 Diabetes):ab,ti,kw OR (Adult-Onset Diabetes Mellitus):ab,ti,kw OR (T2DM):ab,ti,kw

#3=(Renal Insufficiency, Chronic):ab,ti,kw OR (Chronic Renal Insufficiencies):ab,ti,kw OR (Chronic Kidney Disease):ab,ti,kw OR (Chronic Renal Disease):ab,ti,kw OR (CKD):ab,ti,kw

#4=#2 AND #3

CNKI

SU%=('钠-葡萄糖共转运蛋白-2抑制剂' + 'SGLT2' + 'SGLT2i' + '卡格列净' + '坎格列嗪' + '恩格列净' + '索格列净' + '艾托格列净' + '胰高血糖素样肽-1受体激动剂' + 'GLP-1' + '利拉鲁肽' + '杜拉糖肽' + '非奈利酮') AND SU%=('2型糖尿病' + '非胰岛素依赖型糖尿病' + 'T2DM' AND SU%='慢性肾功能不全' + '慢性肾功能衰竭' + '慢性肾脏病' + '慢性肾病' + 'CKD')

**CQVIP database**

((U=艾托格列净 OR U=胰高血糖素样肽-1受体激动剂 OR U=GLP-1 OR U=利拉鲁肽 OR U=杜拉糖肽 OR U=菲耐力酮) OR (U=钠-葡萄糖共转运蛋白-2抑制剂 OR M=SGLT-2 OR U=卡格列净 OR U=坎格列嗪 OR U=恩格列净 OR U=达格列净 OR U=索格列净)) AND (U=慢性肾功能不全 OR U=慢性肾脏病 OR U=慢性肾病 OR U=慢性肾功能衰竭 OR U=CKD) AND (U=2型糖尿病 OR U=T2DM OR U=非胰岛素依赖型糖尿病)

WanFang database

(钠-葡萄糖共转运蛋白-2抑制剂+SGLT2+SGLT2i+卡格列净+坎格列嗪+恩格列净+达格列净+索格列净+艾托格列净) or 主题:(胰高血糖素样肽-1受体激动剂+GLP-1+利拉鲁肽+杜拉糖肽) or 主题:(非奈利酮) and 主题:(2型糖尿病+T2DM+非胰岛素依赖型糖尿病) and 主题:(慢性肾功能不全+慢性肾脏病+慢性肾病+慢性肾功能衰竭+CKD)

**Appendix 3 Network Plots**

**a. HbA1c Outcome: Network Plots.**

**
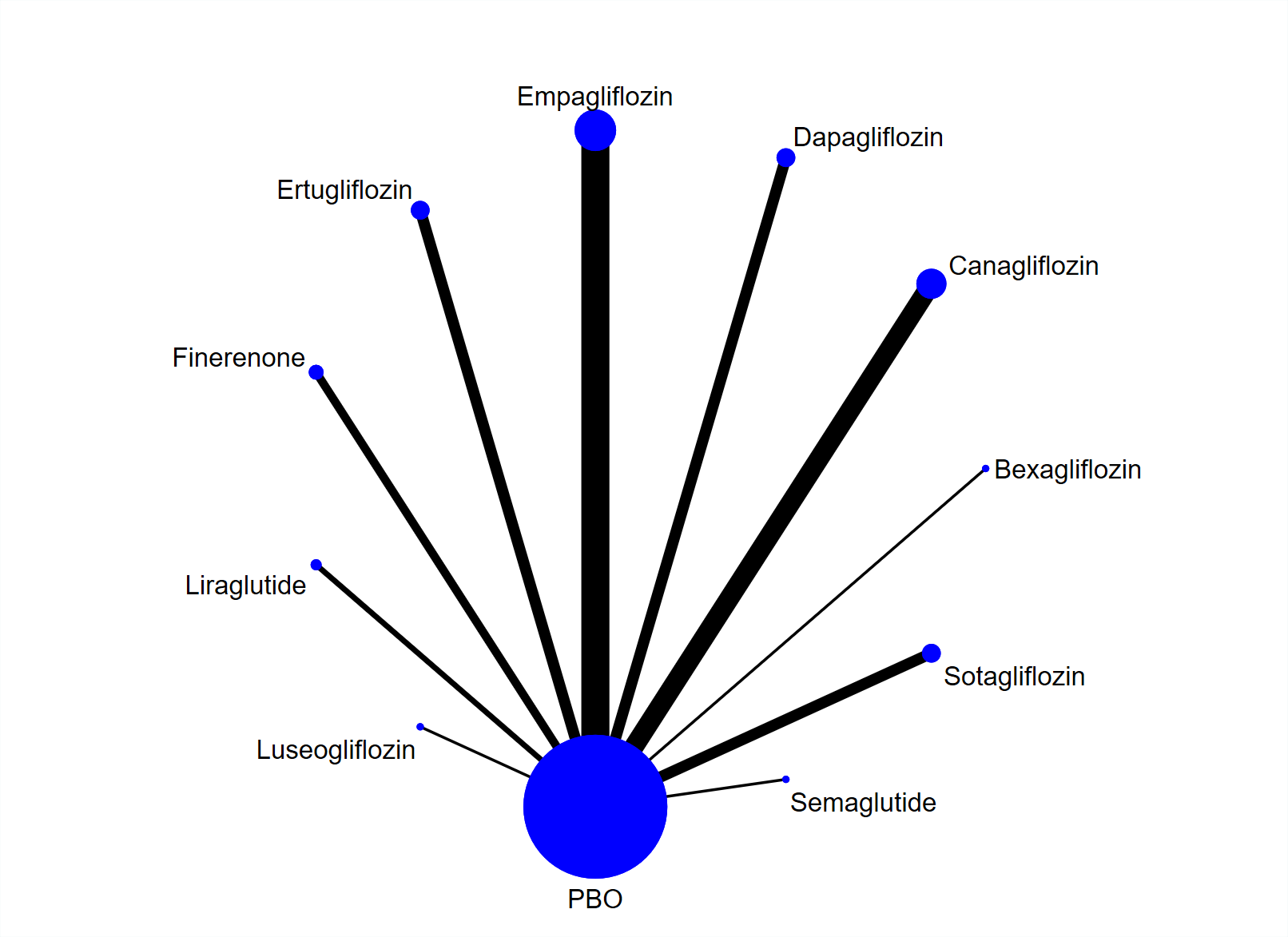
**

The width of the lines is directly proportional to the number of trials comparing each pair of treatments, while the size of each circle is directly proportional to the number of randomized participants (sample size).

**b. eGFR Outcome: Network Plots.**

**
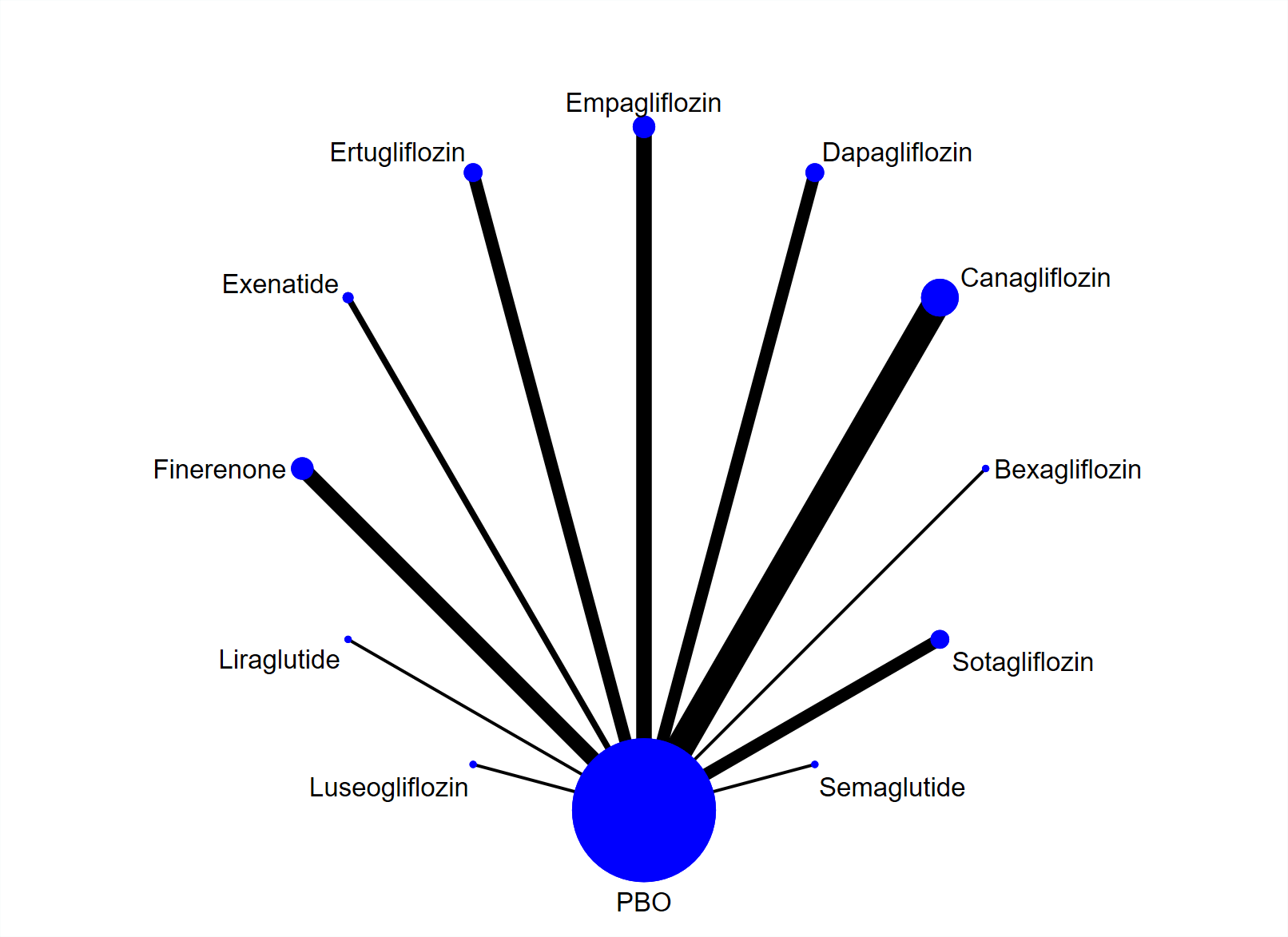
**

The width of the lines is directly proportional to the number of trials comparing each pair of treatments, while the size of each circle is directly proportional to the number of randomized participants (sample size).

**c. LDL-C Outcome: Network Plots.**

**
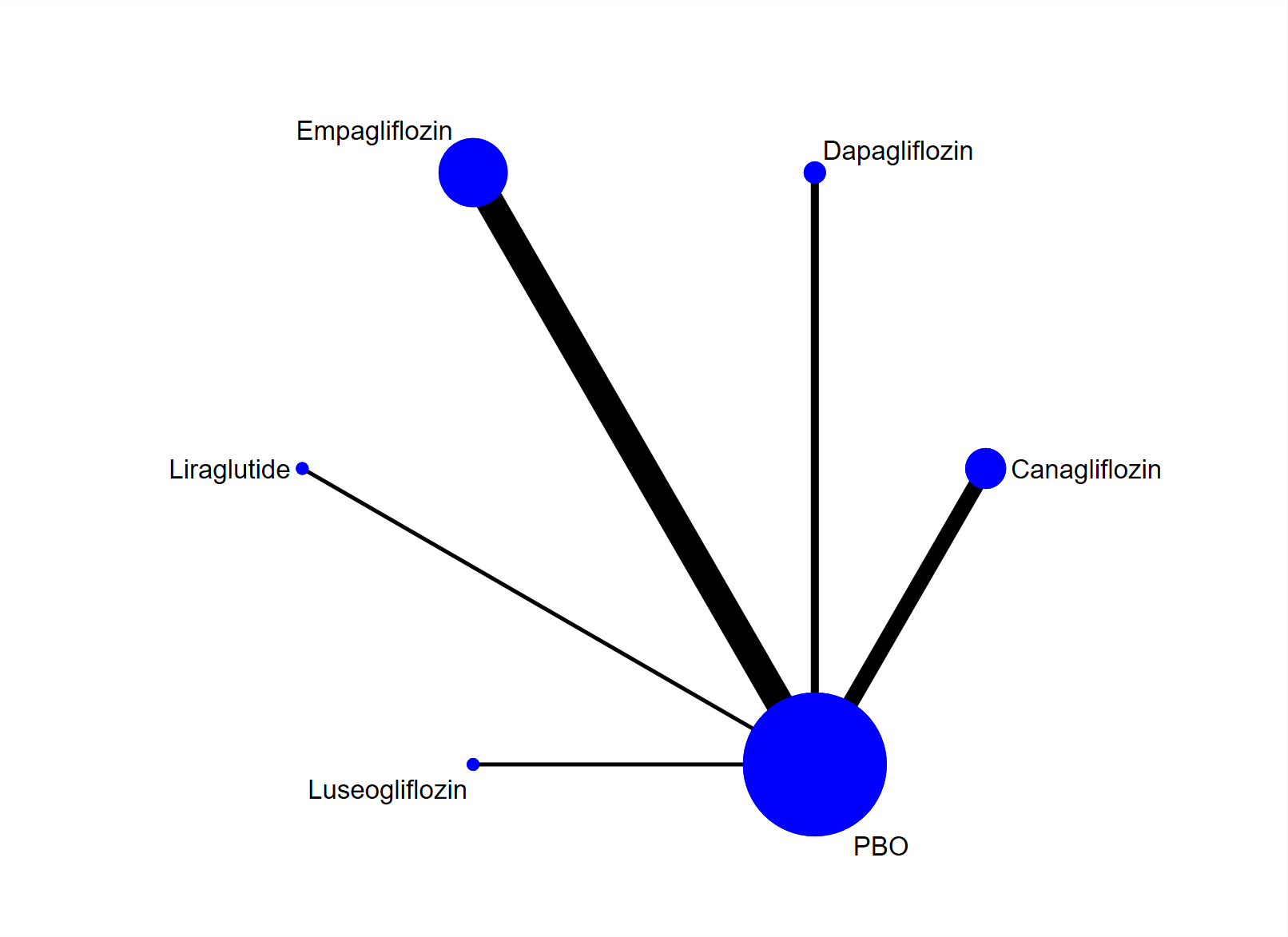
**

The width of the lines is directly proportional to the number of trials comparing each pair of treatments, while the size of each circle is directly proportional to the number of randomized participants (sample size).

**d. SBP Outcome: Network Plots.**

**
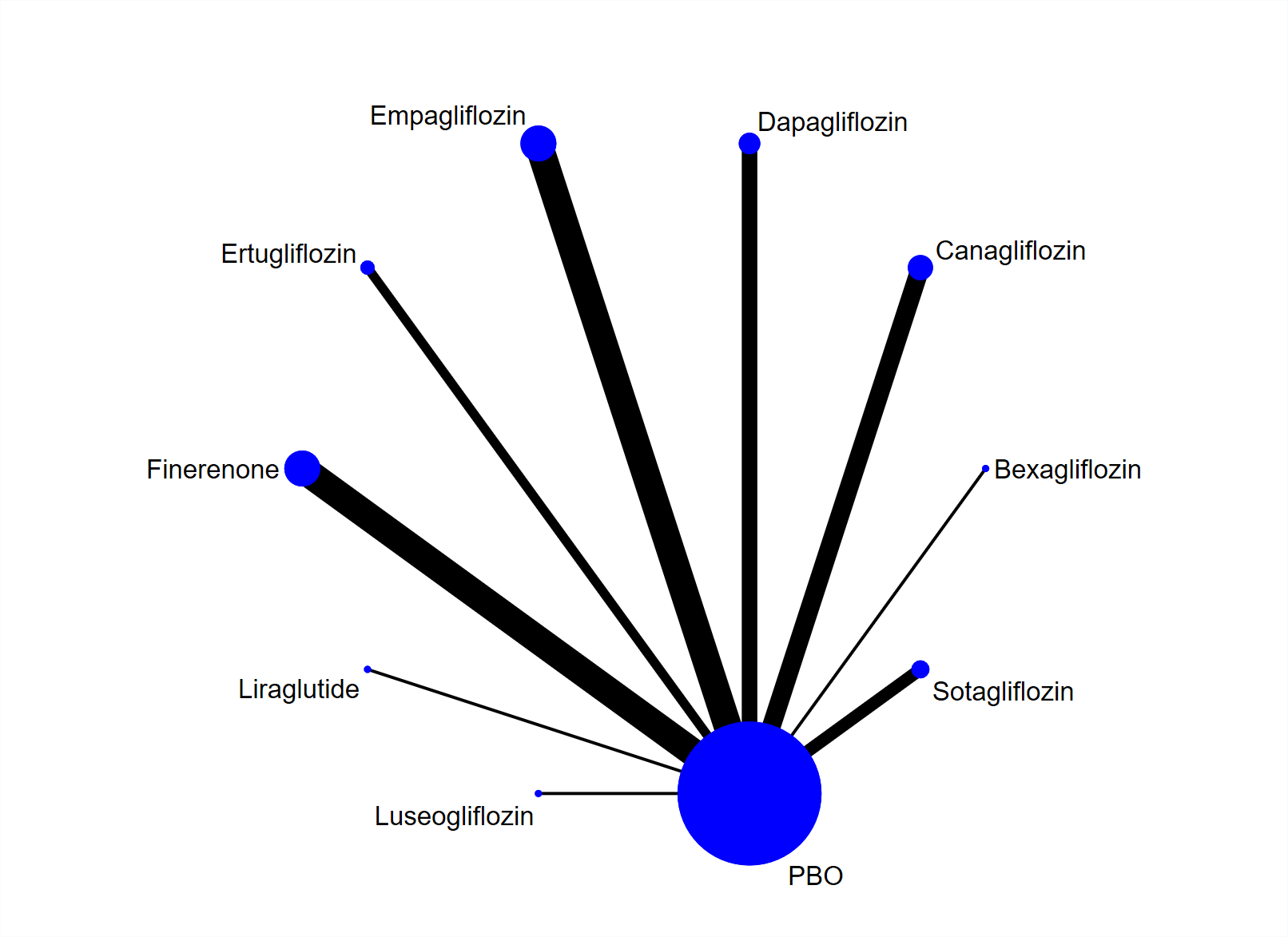
**

The width of the lines is directly proportional to the number of trials comparing each pair of treatments, while the size of each circle is directly proportional to the number of randomized participants (sample size).

**e. DBP Outcome: Network Plots.**

**
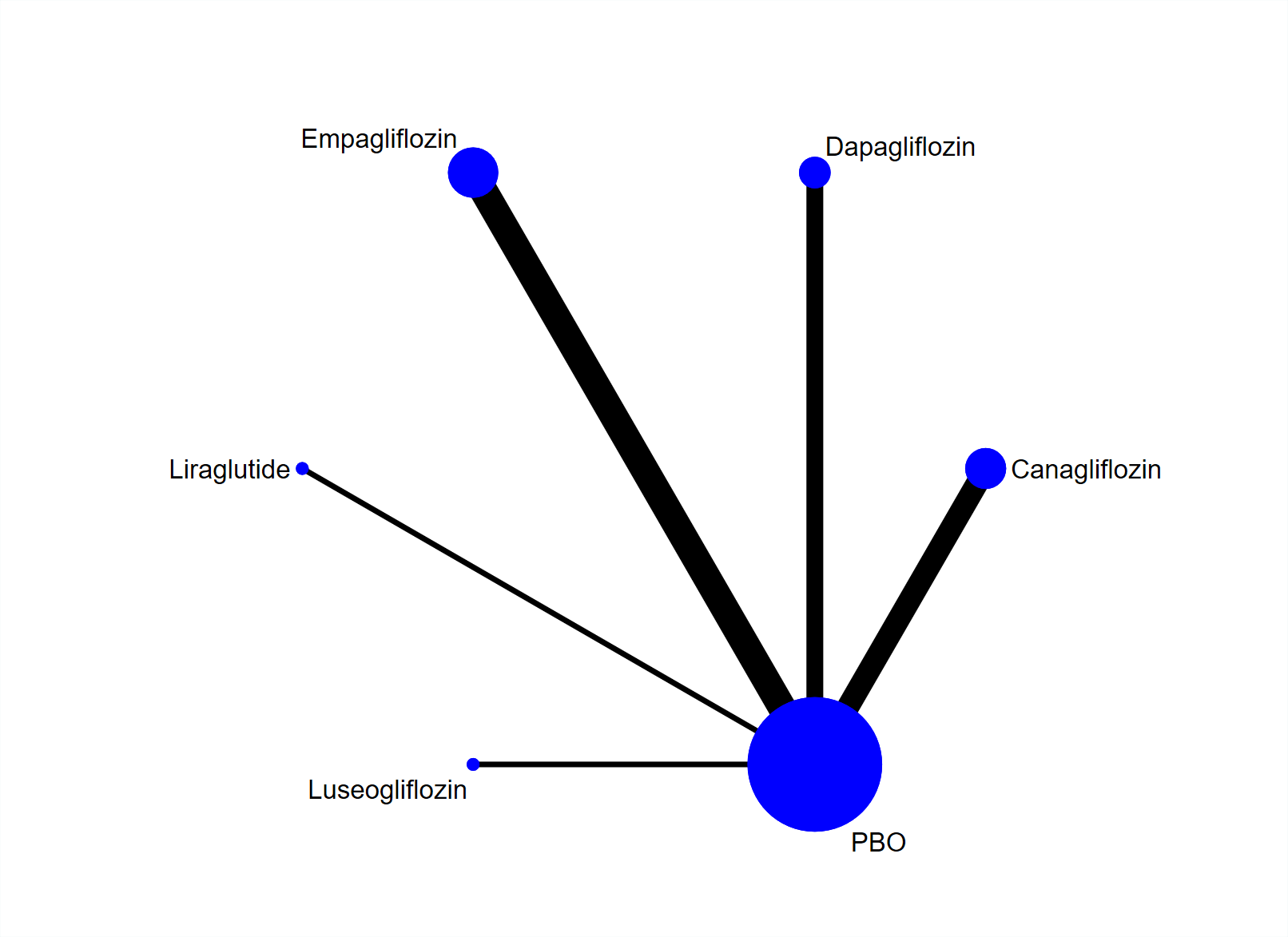
**

The width of the lines is directly proportional to the number of trials comparing each pair of treatments, while the size of each circle is directly proportional to the number of randomized participants (sample size).

**f. Body Weight Outcome: Network Plots.**

**
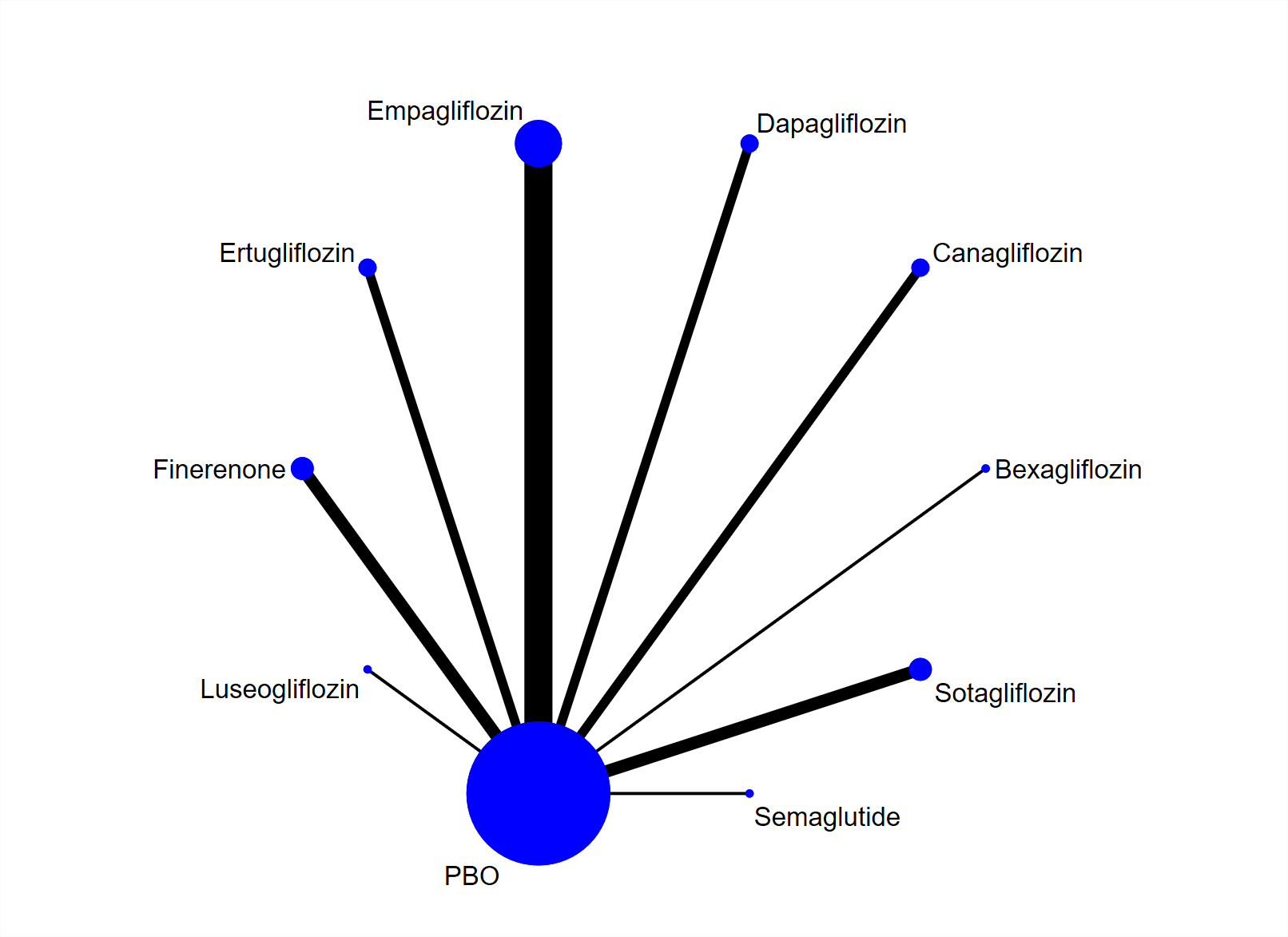
**

The width of the lines is directly proportional to the number of trials comparing each pair of treatments, while the size of each circle is directly proportional to the number of randomized participants (sample size).

**g. any AE Outcome: Network Plots.**

**
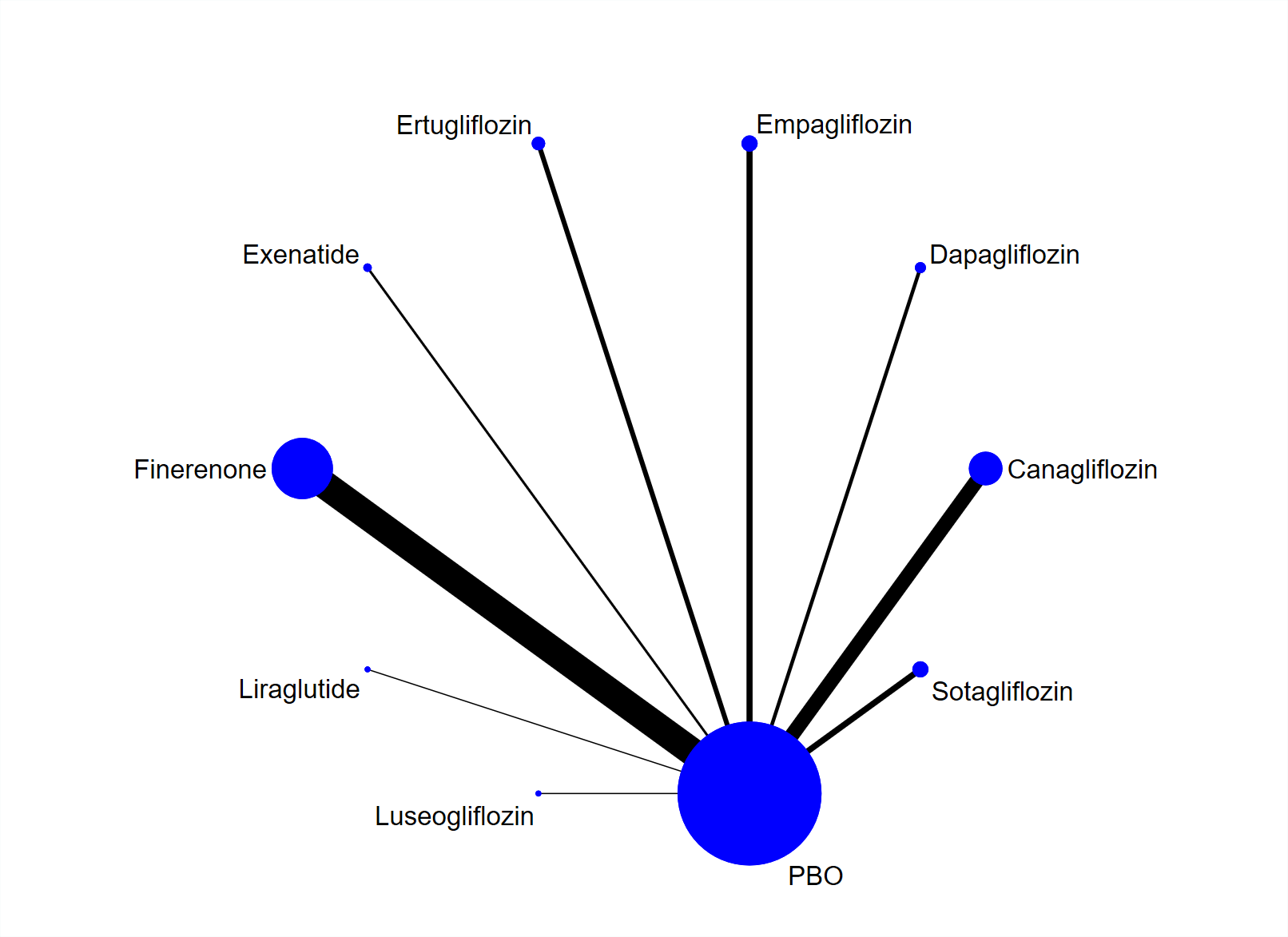
**

The width of the lines is directly proportional to the number of trials comparing each pair of treatments, while the size of each circle is directly proportional to the number of randomized participants (sample size).

**h. UTI Outcome: Network Plots.**

**
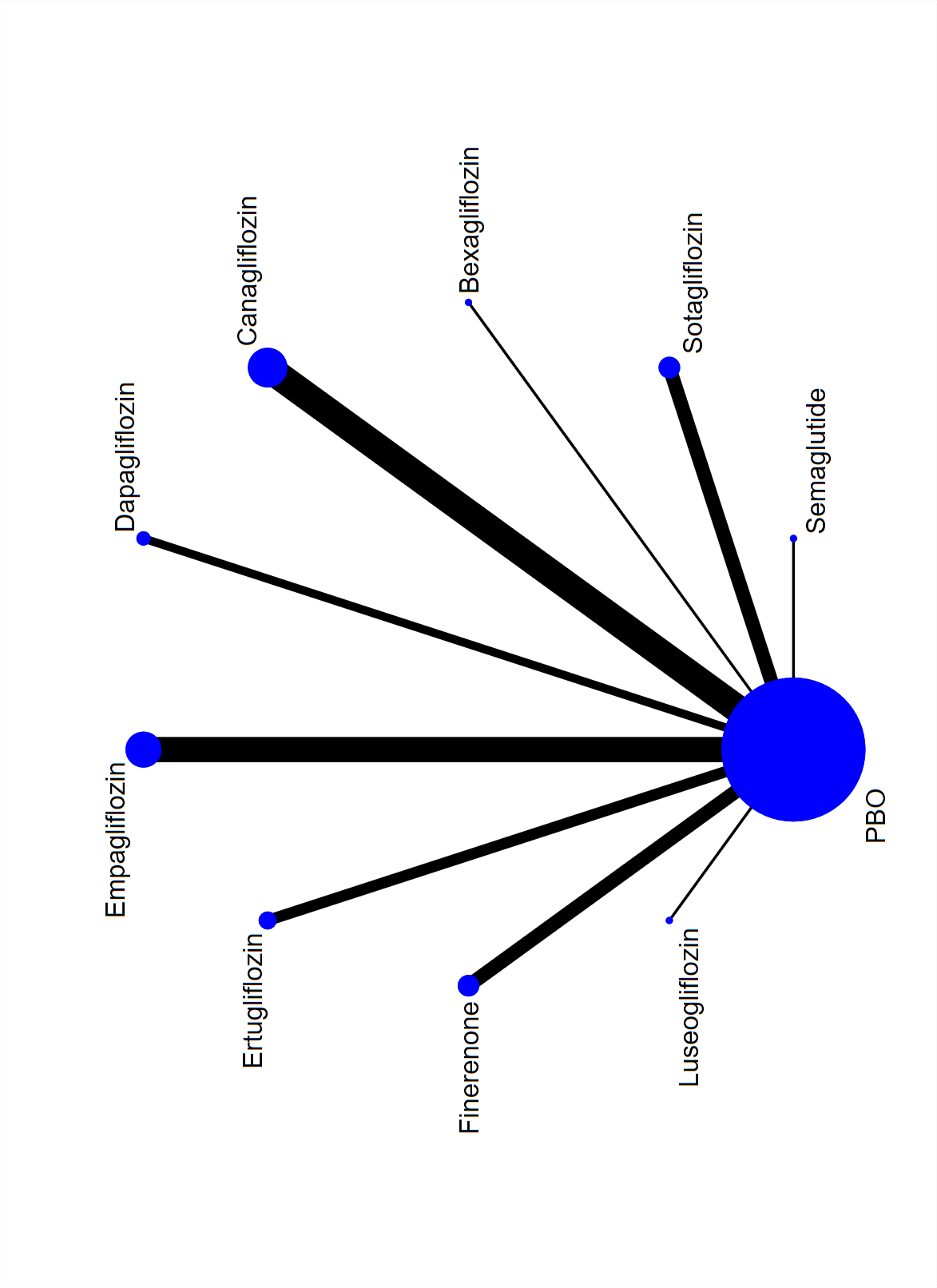
**

The width of the lines is directly proportional to the number of trials comparing each pair of treatments, while the size of each circle is directly proportional to the number of randomized participants (sample size).

**i. Hypoglycemia Outcome: Network Plots.**

**
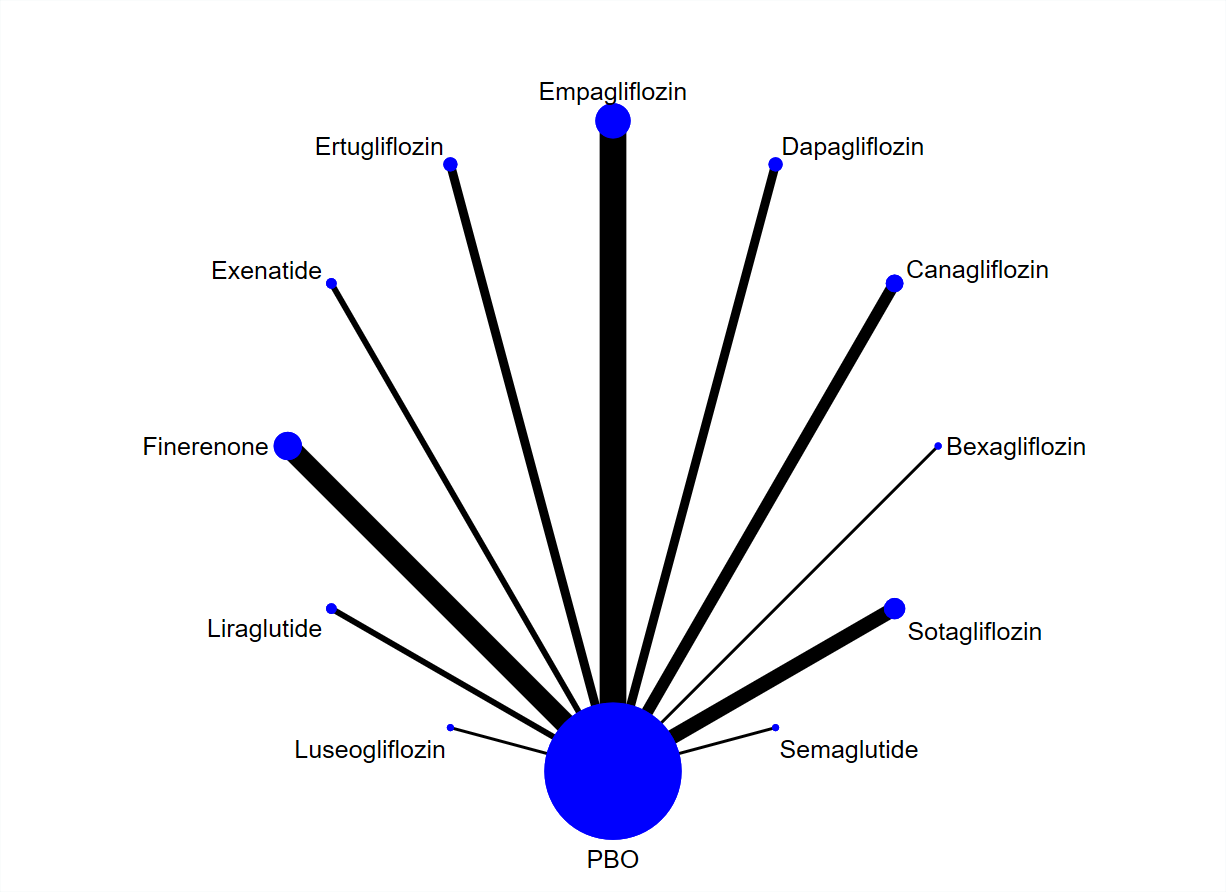
**

The width of the lines is directly proportional to the number of trials comparing each pair of treatments, while the size of each circle is directly proportional to the number of randomized participants (sample size).

**j. AKI Outcome: Network Plots.**

**
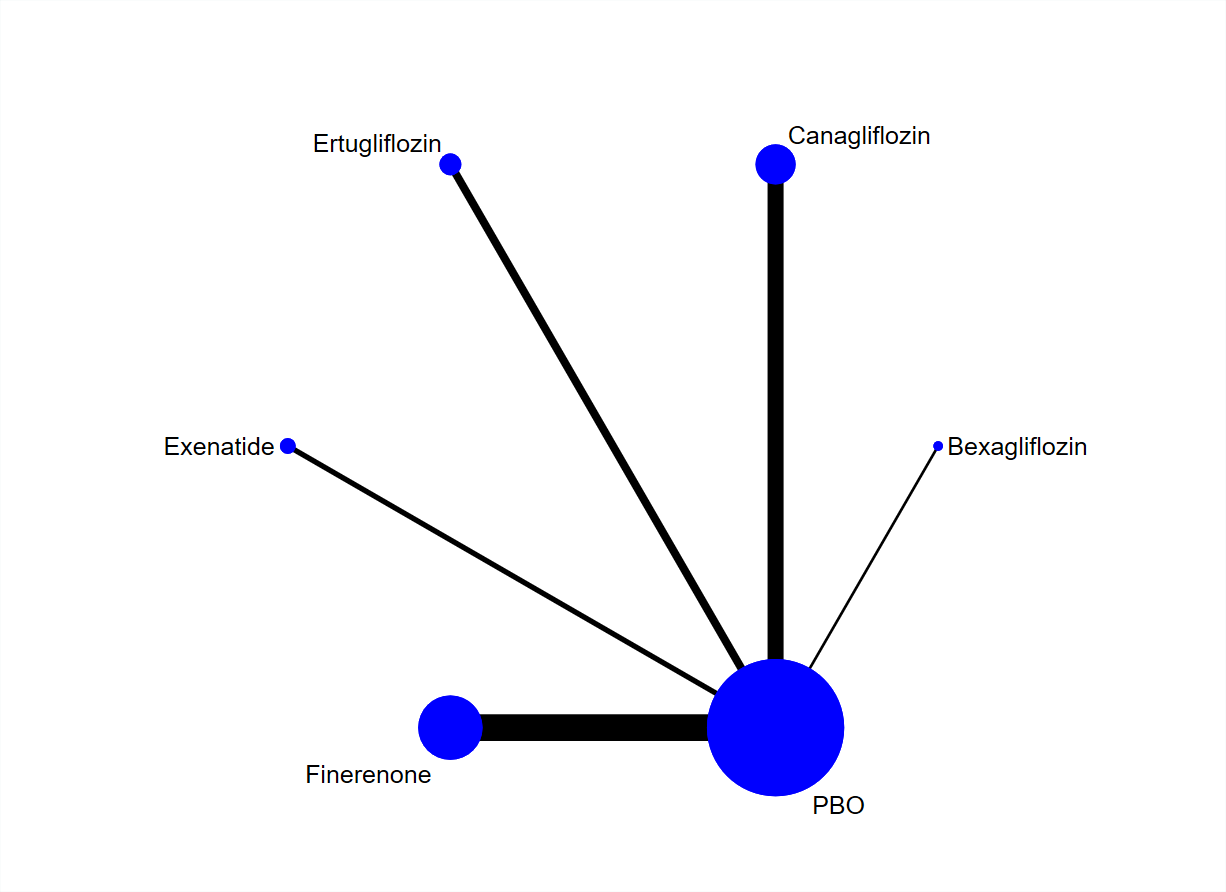
**

The width of the lines is directly proportional to the number of trials comparing each pair of treatments, while the size of each circle is directly proportional to the number of randomized participants (sample size).

**Appendix 4 Risk of bias in included studies**

| **Unique ID** | 1 | **Study ID** | 1 | **Assessor** | G and J |
| --- | --- | --- | --- | --- | --- |
| **Ref or Label** | 10.1111/dom.12090 | **Aim** | assignment to intervention (the 'intention-to-treat' effect) |  |  |
| **Experimental** | Canagliflozin | **Comparator** | PBO | **Source** | Journal article(s) with results of the trial |
| **Outcome** | ①②③④⑤⑥⑦⑩ | **Results** | ①②③④⑤⑥⑦⑩ | **Weight** | 1 |
| **Domain** | **Signalling question** | | | **Response** | **Comments** |
| **Bias arising from the randomization process** | 1.1 Was the allocation sequence random? | | | Y | Randomization and Study Treatments Eligible subjects were randomly assigned to receive once-daily oral doses of canagliflozin 100 or 300 mg or placebo in a 1 : 1 : 1 ratio using an Interactive Voice Response System/Interactive Web Response System. |
|  | 1.2 Was the allocation sequence concealed until participants were enrolled and assigned to interventions? | | | Y |  |
|  | 1.3 Did baseline differences between intervention groups suggest a problem with the randomization process? | | | N | Baseline demographic and disease characteristics were similar across treatment groups |
|  | **Risk of bias judgement** | | | **Low** |  |
| **Bias due to deviations from intended interventions** | 2.1.Were participants aware of their assigned intervention during the trial? | | | PN | Randomization and Study Treatments Eligible subjects were randomly assigned to receive once-daily oral doses of canagliflozin 100 or 300 mg or placebo in a 1 : 1 : 1 ratio using an Interactive Voice Response System/Interactive Web Response System. |
|  | 2.2.Were carers and people delivering the interventions aware of participants' assigned intervention during the trial? | | | PN |  |
|  |  | | |  |  |
|  | 2.3. If Y/PY/NI to 2.1 or 2.2: Were there deviations from the intended intervention that arose because of the experimental context? | | | NA |  |
|  | 2.4 If Y/PY to 2.3: Were these deviations likely to have affected the outcome? | | | NA |  |
|  | 2.5. If Y/PY/NI to 2.4: Were these deviations from intended intervention balanced between groups? | | | NA |  |
|  | 2.6 Was an appropriate analysis used to estimate the effect of assignment to intervention? | | | Y | Efficacy analyses were conducted using the modified intentto-treat (mITT) population, which consisted of all randomized subjects who received ≥1 dose of study drug, according to the randomized treatment assignment. |
|  | 2.7 If N/PN/NI to 2.6: Was there potential for a substantial impact (on the result) of the failure to analyse participants in the group to which they were randomized? | | | NA |  |
|  | **Risk of bias judgement** | | | **Low** |  |
| **Bias due to missing outcome data** | 3.1 Were data for this outcome available for all, or nearly all, participants randomized? | | | Y | Of the 272 randomized subjects, 269 received ≥1 dose of study drug and were included in the mITT analysis population |
|  | 3.2 If N/PN/NI to 3.1: Is there evidence that result was not biased by missing outcome data? | | | NA |  |
|  | 3.3 If N/PN to 3.2: Could missingness in the outcome depend on its true value? | | | NA |  |
|  | 3.4 If Y/PY/NI to 3.3: Is it likely that missingness in the outcome depended on its true value? | | | NA |  |
|  | **Risk of bias judgement** | | | **Low** |  |
| **Bias in measurement of the outcome** | 4.1 Was the method of measuring the outcome inappropriate? | | | PN |  |
|  | 4.2 Could measurement or ascertainment of the outcome have differed between intervention groups? | | | PN |  |
|  | 4.3 Were outcome assessors aware of the intervention received by study participants? | | | NI | Not explicitly mentioned |
|  | 4.4 If Y/PY/NI to 4.3: Could assessment of the outcome have been influenced by knowledge of intervention received? | | | PN |  |
|  | 4.5 If Y/PY/NI to 4.4: Is it likely that assessment of the outcome was influenced by knowledge of intervention received? | | | NA |  |
|  | **Risk of bias judgement** | | | **Low** |  |
| **Bias in selection of the reported result** | 5.1 Were the data that produced this result analysed in accordance with a pre-specified analysis plan that was finalized before unblinded outcome data were available for analysis? | | | Y |  |
|  | 5.2 ... multiple eligible outcome measurements (e.g. scales, definitions, time points) within the outcome domain? | | | PN |  |
|  | 5.3 ... multiple eligible analyses of the data? | | | PN |  |
|  | **Risk of bias judgement** | | | **Low** |  |
| **Overall bias** | **Risk of bias judgement** | | | **Low** |  |
| **Unique ID** | 2 | **Study ID** | 2 | **Assessor** | G and J |
| **Ref or Label** | China Modern Medicine, Volume 23, Number 30, October 2016 | **Aim** | assignment to intervention (the 'intention-to-treat' effect) |  |  |
| **Experimental** | Liraglutide | **Comparator** | PBO | **Source** | Journal article(s) with results of the trial |
| **Outcome** | ①②③④⑤⑧ | **Results** | ①②③④⑤⑧ | **Weight** | 1 |
| **Domain** | **Signalling question** | | | **Response** | **Comments** |
| **Bias arising from the randomization process** | 1.1 Was the allocation sequence random? | | | Y | Random number table method |
|  | 1.2 Was the allocation sequence concealed until participants were enrolled and assigned to interventions? | | | PY |  |
|  | 1.3 Did baseline differences between intervention groups suggest a problem with the randomization process? | | | N | The baseline comparability was good |
|  | **Risk of bias judgement** | | | **Low** |  |
| **Bias due to deviations from intended interventions** | 2.1.Were participants aware of their assigned intervention during the trial? | | | PY | The experimental group was treated with subcutaneous injection of Liraglutide, and the control group was treated with insulin or oral hypoglycemic medicines, and the mode of administration may be different. |
|  | 2.2.Were carers and people delivering the interventions aware of participants' assigned intervention during the trial? | | | PY |  |
|  | 2.3. If Y/PY/NI to 2.1 or 2.2: Were there deviations from the intended intervention that arose because of the experimental context? | | | NI | Not explicitly mentioned |
|  | 2.4 If Y/PY to 2.3: Were these deviations likely to have affected the outcome? | | | NA |  |
|  | 2.5. If Y/PY/NI to 2.4: Were these deviations from intended intervention balanced between groups? | | | NA |  |
|  | 2.6 Was an appropriate analysis used to estimate the effect of assignment to intervention? | | | PY | SPSS 13.0 statistical software was used to analyze the data. The measurement data were expressed as mean ± standard deviation (x±s), t test was used, the count data were expressed as percentage (%), χ2 test was used, and P<0.05 was considered statistically significant. |
|  | 2.7 If N/PN/NI to 2.6: Was there potential for a substantial impact (on the result) of the failure to analyse participants in the group to which they were randomized? | | | NA |  |
|  | **Risk of bias judgement** | | | **Some concerns** |  |
| **Bias due to missing outcome data** | 3.1 Were data for this outcome available for all, or nearly all, participants randomized? | | | Y | Few were lost to follow-up |
|  | 3.2 If N/PN/NI to 3.1: Is there evidence that result was not biased by missing outcome data? | | | NA |  |
|  | 3.3 If N/PN to 3.2: Could missingness in the outcome depend on its true value? | | | NA |  |
|  | 3.4 If Y/PY/NI to 3.3: Is it likely that missingness in the outcome depended on its true value? | | | NA |  |
|  | **Risk of bias judgement** | | | **Low** |  |
| **Bias in measurement of the outcome** | 4.1 Was the method of measuring the outcome inappropriate? | | | N |  |
|  | 4.2 Could measurement or ascertainment of the outcome have differed between intervention groups? | | | N |  |
|  | 4.3 Were outcome assessors aware of the intervention received by study participants? | | | NI | Not explicitly mentioned |
|  | 4.4 If Y/PY/NI to 4.3: Could assessment of the outcome have been influenced by knowledge of intervention received? | | | PN |  |
|  | 4.5 If Y/PY/NI to 4.4: Is it likely that assessment of the outcome was influenced by knowledge of intervention received? | | | NA |  |
|  | **Risk of bias judgement** | | | **Low** |  |
| **Bias in selection of the reported result** | 5.1 Were the data that produced this result analysed in accordance with a pre-specified analysis plan that was finalized before unblinded outcome data were available for analysis? | | | Y |  |
|  | 5.2 ... multiple eligible outcome measurements (e.g. scales, definitions, time points) within the outcome domain? | | | N |  |
|  | 5.3 ... multiple eligible analyses of the data? | | | N |  |
|  | **Risk of bias judgement** | | | **Low** |  |
| **Overall bias** | **Risk of bias judgement** | | | **Some concerns** |  |
| **Unique ID** | 3 | **Study ID** | 3 | **Assessor** | G and J |
| **Ref or Label** | Famous doctors,2022,(17):162-164. | **Aim** | assignment to intervention (the 'intention-to-treat' effect) |  |  |
| **Experimental** | Liraglutide | **Comparator** | PBO | **Source** | Journal article(s) with results of the trial |
| **Outcome** | ①②⑧⑩ | **Results** | ①②⑧⑩ | **Weight** | 1 |
| **Domain** | **Signalling question** | | | **Response** | **Comments** |
| **Bias arising from the randomization process** | 1.1 Was the allocation sequence random? | | | N | According to the different treatment methods, they were divided into the control group and the study group, 26 cases in each group. Patients were informed. |
|  | 1.2 Was the allocation sequence concealed until participants were enrolled and assigned to interventions? | | | PN |  |
|  | 1.3 Did baseline differences between intervention groups suggest a problem with the randomization process? | | | N | There was no significant difference in general data between 2 groups (P > 0.05). |
|  | **Risk of bias judgement** | | | **High** |  |
| **Bias due to deviations from intended interventions** | 2.1.Were participants aware of their assigned intervention during the trial? | | | Y | According to the different treatment methods, they were divided into the control group and the study group, 26 cases in each group. Patients were informed. |
|  | 2.2.Were carers and people delivering the interventions aware of participants' assigned intervention during the trial? | | | Y |  |
|  | 2.3. If Y/PY/NI to 2.1 or 2.2: Were there deviations from the intended intervention that arose because of the experimental context? | | | PY | All subjects in each group were aware of their grouping |
|  | 2.4 If Y/PY to 2.3: Were these deviations likely to have affected the outcome? | | | NI | Difficult to judge |
|  | 2.5. If Y/PY/NI to 2.4: Were these deviations from intended intervention balanced between groups? | | | NI | Not explicitly mentioned |
|  | 2.6 Was an appropriate analysis used to estimate the effect of assignment to intervention? | | | Y |  |
|  | 2.7 If N/PN/NI to 2.6: Was there potential for a substantial impact (on the result) of the failure to analyse participants in the group to which they were randomized? | | | NA |  |
|  | **Risk of bias judgement** | | | **High** |  |
| **Bias due to missing outcome data** | 3.1 Were data for this outcome available for all, or nearly all, participants randomized? | | | Y | No patients dropped out |
|  | 3.2 If N/PN/NI to 3.1: Is there evidence that result was not biased by missing outcome data? | | | NA |  |
|  | 3.3 If N/PN to 3.2: Could missingness in the outcome depend on its true value? | | | NA |  |
|  | 3.4 If Y/PY/NI to 3.3: Is it likely that missingness in the outcome depended on its true value? | | | NA |  |
|  | **Risk of bias judgement** | | | **Low** |  |
| **Bias in measurement of the outcome** | 4.1 Was the method of measuring the outcome inappropriate? | | | N | Outcome measures were clearly defined |
|  | 4.2 Could measurement or ascertainment of the outcome have differed between intervention groups? | | | N | Outcome measures were clearly defined |
|  | 4.3 Were outcome assessors aware of the intervention received by study participants? | | | Y | According to the different treatment methods, they were divided into the control group and the study group, 26 cases in each group. Patients were informed. |
|  | 4.4 If Y/PY/NI to 4.3: Could assessment of the outcome have been influenced by knowledge of intervention received? | | | PN | Outcome measures were clearly defined |
|  | 4.5 If Y/PY/NI to 4.4: Is it likely that assessment of the outcome was influenced by knowledge of intervention received? | | | NA |  |
|  | **Risk of bias judgement** | | | **Low** |  |
| **Bias in selection of the reported result** | 5.1 Were the data that produced this result analysed in accordance with a pre-specified analysis plan that was finalized before unblinded outcome data were available for analysis? | | | Y | SPSS 23.0 statistical software was used for data analysis. Measurement data were expressed as (x± s) and t test was used. Count data were expressed as rate (%) and χ2 test was used. P < 0.05 was considered statistically significant. |
|  | 5.2 ... multiple eligible outcome measurements (e.g. scales, definitions, time points) within the outcome domain? | | | N | Not explicitly mentioned |
|  | 5.3 ... multiple eligible analyses of the data? | | | N | Not explicitly mentioned |
|  | **Risk of bias judgement** | | | **Low** |  |
| **Overall bias** | **Risk of bias judgement** | | | **High** |  |
| **Unique ID** | 4 | **Study ID** | NCT01164501 | **Assessor** | G and J |
| **Ref or Label** | 10.1016/S2213-8587(13)70208-0 | **Aim** | assignment to intervention (the 'intention-to-treat' effect) |  |  |
| **Experimental** | Empagliflozin | **Comparator** | PBO | **Source** | Journal article(s) with results of the trial; Trial protocol |
| **Outcome** | ①②③④⑤⑥⑦⑧⑩ | **Results** | ①②③④⑤⑥⑦⑧⑩ | **Weight** | 1 |
| **Domain** | **Signalling question** | | | **Response** | **Comments** |
| **Bias arising from the randomization process** | 1.1 Was the allocation sequence random? | | | Y | Randomisation was done by the study sponsor via an interactive response system using a computer-generated random sequence |
|  | 1.2 Was the allocation sequence concealed until participants were enrolled and assigned to interventions? | | | Y |  |
|  | 1.3 Did baseline differences between intervention groups suggest a problem with the randomization process? | | | N | Demographics and baseline characteristics were balanced between groups |
|  | **Risk of bias judgement** | | | **Low** |  |
| **Bias due to deviations from intended interventions** | 2.1.Were participants aware of their assigned intervention during the trial? | | | N | Treatment allocation during the treatment period was masked from patients, investigators, and those involved in analysing trial data. |
|  | 2.2.Were carers and people delivering the interventions aware of participants' assigned intervention during the trial? | | | N |  |
|  | 2.3. If Y/PY/NI to 2.1 or 2.2: Were there deviations from the intended intervention that arose because of the experimental context? | | | NA |  |
|  | 2.4 If Y/PY to 2.3: Were these deviations likely to have affected the outcome? | | | NA |  |
|  | 2.5. If Y/PY/NI to 2.4: Were these deviations from intended intervention balanced between groups? | | | NA |  |
|  | 2.6 Was an appropriate analysis used to estimate the effect of assignment to intervention? | | | Y | The data analysis methods are described in detail |
|  | 2.7 If N/PN/NI to 2.6: Was there potential for a substantial impact (on the result) of the failure to analyse participants in the group to which they were randomized? | | | NA |  |
|  | **Risk of bias judgement** | | | **Low** |  |
| **Bias due to missing outcome data** | 3.1 Were data for this outcome available for all, or nearly all, participants randomized? | | | Y |  |
|  | 3.2 If N/PN/NI to 3.1: Is there evidence that result was not biased by missing outcome data? | | | NA |  |
|  | 3.3 If N/PN to 3.2: Could missingness in the outcome depend on its true value? | | | NA |  |
|  | 3.4 If Y/PY/NI to 3.3: Is it likely that missingness in the outcome depended on its true value? | | | NA |  |
|  | **Risk of bias judgement** | | | **Low** |  |
| **Bias in measurement of the outcome** | 4.1 Was the method of measuring the outcome inappropriate? | | | N |  |
|  | 4.2 Could measurement or ascertainment of the outcome have differed between intervention groups? | | | PN | Outcome measures were clearly defined |
|  | 4.3 Were outcome assessors aware of the intervention received by study participants? | | | N | Treatment allocation during the treatment period was masked from patients, investigators, and those involved in analysing trial data. |
|  | 4.4 If Y/PY/NI to 4.3: Could assessment of the outcome have been influenced by knowledge of intervention received? | | | NA |  |
|  | 4.5 If Y/PY/NI to 4.4: Is it likely that assessment of the outcome was influenced by knowledge of intervention received? | | | NA |  |
|  | **Risk of bias judgement** | | | **Low** |  |
| **Bias in selection of the reported result** | 5.1 Were the data that produced this result analysed in accordance with a pre-specified analysis plan that was finalized before unblinded outcome data were available for analysis? | | | Y |  |
|  | 5.2 ... multiple eligible outcome measurements (e.g. scales, definitions, time points) within the outcome domain? | | | N |  |
|  | 5.3 ... multiple eligible analyses of the data? | | | N |  |
|  | **Risk of bias judgement** | | | **Low** |  |
| **Overall bias** | **Risk of bias judgement** | | | **Low** |  |
|  |  |  |  |  |  |
|  |  |  |  |  |  |
| **Unique ID** | 5 | **Study ID** | NCT02540993 and NCT02545049 | **Assessor** | G and J |
| **Ref or Label** | 10.1016/j.xkme.2023.100704 | **Aim** | assignment to intervention (the 'intention-to-treat' effect) |  |  |
| **Experimental** | Finerenone | **Comparator** | PBO | **Source** | Journal article(s) with results of the trial; Trial protocol |
| **Outcome** | ⑩ | **Results** | ⑩ | **Weight** | 1 |
| **Domain** | **Signalling question** | | | **Response** | **Comments** |
| **Bias arising from the randomization process** | 1.1 Was the allocation sequence random? | | | NI | The only information about the randomization method is that the study was randomized |
|  | 1.2 Was the allocation sequence concealed until participants were enrolled and assigned to interventions? | | | Y |  |
|  | 1.3 Did baseline differences between intervention groups suggest a problem with the randomization process? | | | N | Comparable at baseline |
|  | **Risk of bias judgement** | | | **Low** |  |
| **Bias due to deviations from intended interventions** | 2.1.Were participants aware of their assigned intervention during the trial? | | | N | Patients were randomly assigned (1:1) to receive doubleblind, once-daily oral treatment with finerenone (at titrated doses of 10 or 20 mg) or a matching placebo |
|  | 2.2.Were carers and people delivering the interventions aware of participants' assigned intervention during the trial? | | | N |  |
|  | 2.3. If Y/PY/NI to 2.1 or 2.2: Were there deviations from the intended intervention that arose because of the experimental context? | | | NA |  |
|  | 2.4 If Y/PY to 2.3: Were these deviations likely to have affected the outcome? | | | NA |  |
|  | 2.5. If Y/PY/NI to 2.4: Were these deviations from intended intervention balanced between groups? | | | NA |  |
|  | 2.6 Was an appropriate analysis used to estimate the effect of assignment to intervention? | | | Y | The statistical methods are described in detail |
|  | 2.7 If N/PN/NI to 2.6: Was there potential for a substantial impact (on the result) of the failure to analyse participants in the group to which they were randomized? | | | NA |  |
|  | **Risk of bias judgement** | | | **Low** |  |
| **Bias due to missing outcome data** | 3.1 Were data for this outcome available for all, or nearly all, participants randomized? | | | Y | Small number of missing |
|  | 3.2 If N/PN/NI to 3.1: Is there evidence that result was not biased by missing outcome data? | | | NA |  |
|  | 3.3 If N/PN to 3.2: Could missingness in the outcome depend on its true value? | | | NA |  |
|  | 3.4 If Y/PY/NI to 3.3: Is it likely that missingness in the outcome depended on its true value? | | | NA |  |
|  | **Risk of bias judgement** | | | **Low** |  |
| **Bias in measurement of the outcome** | 4.1 Was the method of measuring the outcome inappropriate? | | | N |  |
|  | 4.2 Could measurement or ascertainment of the outcome have differed between intervention groups? | | | N |  |
|  | 4.3 Were outcome assessors aware of the intervention received by study participants? | | | N | Patients were randomly assigned (1:1) to receive doubleblind, once-daily oral treatment with finerenone (at titrated doses of 10 or 20 mg) or a matching placebo |
|  | 4.4 If Y/PY/NI to 4.3: Could assessment of the outcome have been influenced by knowledge of intervention received? | | | NA |  |
|  | 4.5 If Y/PY/NI to 4.4: Is it likely that assessment of the outcome was influenced by knowledge of intervention received? | | | NA |  |
|  | **Risk of bias judgement** | | | **Low** |  |
| **Bias in selection of the reported result** | 5.1 Were the data that produced this result analysed in accordance with a pre-specified analysis plan that was finalized before unblinded outcome data were available for analysis? | | | Y | The data analysis methods are described in detail |
|  | 5.2 ... multiple eligible outcome measurements (e.g. scales, definitions, time points) within the outcome domain? | | | N |  |
|  | 5.3 ... multiple eligible analyses of the data? | | | N | Not explicitly mentioned |
|  | **Risk of bias judgement** | | | **Low** |  |
| **Overall bias** | **Risk of bias judgement** | | | **Low** |  |
| **Unique ID** | 6 | **Study ID** | 6 | **Assessor** | G and J |
| **Ref or Label** | 10.1159/000364909 | **Aim** | assignment to intervention (the 'intention-to-treat' effect) |  |  |
| **Experimental** | Canagliflozin | **Comparator** | PBO | **Source** | Journal article(s) with results of the trial; Trial protocol |
| **Outcome** | ①②③④⑥⑦⑩ | **Results** | ①②③④⑥⑦⑩ | **Weight** | 1 |
| **Domain** | **Signalling question** | | | **Response** | **Comments** |
| **Bias arising from the randomization process** | 1.1 Was the allocation sequence random? | | | NI | The only information about the randomization method is that the study was randomized |
|  | 1.2 Was the allocation sequence concealed until participants were enrolled and assigned to interventions? | | | NI |  |
|  | 1.3 Did baseline differences between intervention groups suggest a problem with the randomization process? | | | N | The baseline demographic characteristics were similar  among the treatment arms |
|  | **Risk of bias judgement** | | | **Some concerns** |  |
| **Bias due to deviations from intended interventions** | 2.1.Were participants aware of their assigned intervention during the trial? | | | N | Data were pooled from the cohorts enrolled in four randomized, placebo-controlled. |
|  | 2.2.Were carers and people delivering the interventions aware of participants' assigned intervention during the trial? | | | PN |  |
|  | 2.3. If Y/PY/NI to 2.1 or 2.2: Were there deviations from the intended intervention that arose because of the experimental context? | | | NA |  |
|  | 2.4 If Y/PY to 2.3: Were these deviations likely to have affected the outcome? | | | NA |  |
|  | 2.5. If Y/PY/NI to 2.4: Were these deviations from intended intervention balanced between groups? | | | NA |  |
|  | 2.6 Was an appropriate analysis used to estimate the effect of assignment to intervention? | | | Y |  |
|  | 2.7 If N/PN/NI to 2.6: Was there potential for a substantial impact (on the result) of the failure to analyse participants in the group to which they were randomized? | | | NA |  |
|  | **Risk of bias judgement** | | | **Low** |  |
| **Bias due to missing outcome data** | 3.1 Were data for this outcome available for all, or nearly all, participants randomized? | | | Y | Fewer dropouts |
|  | 3.2 If N/PN/NI to 3.1: Is there evidence that result was not biased by missing outcome data? | | | NA |  |
|  | 3.3 If N/PN to 3.2: Could missingness in the outcome depend on its true value? | | | NA |  |
|  | 3.4 If Y/PY/NI to 3.3: Is it likely that missingness in the outcome depended on its true value? | | | NA |  |
|  | **Risk of bias judgement** | | | **Low** |  |
| **Bias in measurement of the outcome** | 4.1 Was the method of measuring the outcome inappropriate? | | | N |  |
|  | 4.2 Could measurement or ascertainment of the outcome have differed between intervention groups? | | | PN |  |
|  | 4.3 Were outcome assessors aware of the intervention received by study participants? | | | PN |  |
|  | 4.4 If Y/PY/NI to 4.3: Could assessment of the outcome have been influenced by knowledge of intervention received? | | | NA |  |
|  | 4.5 If Y/PY/NI to 4.4: Is it likely that assessment of the outcome was influenced by knowledge of intervention received? | | | NA |  |
|  | **Risk of bias judgement** | | | **Low** |  |
| **Bias in selection of the reported result** | 5.1 Were the data that produced this result analysed in accordance with a pre-specified analysis plan that was finalized before unblinded outcome data were available for analysis? | | | PY |  |
|  | 5.2 ... multiple eligible outcome measurements (e.g. scales, definitions, time points) within the outcome domain? | | | N |  |
|  | 5.3 ... multiple eligible analyses of the data? | | | N |  |
|  | **Risk of bias judgement** | | | **Low** |  |
| **Overall bias** | **Risk of bias judgement** | | | **Some concerns** |  |
| **Unique ID** | 7 | **Study ID** | NCT01177813 (study 1); NCT01159600 (study 2); NCT01159600 (study 3); NCT01210001 (study 4); and NCT01164501 (study 5). | **Assessor** | G and J |
| **Ref or Label** | 10.1007/s00125-016-4008-2 | **Aim** | assignment to intervention (the 'intention-to-treat' effect) |  |  |
| **Experimental** | Empagliflozin | **Comparator** | PBO | **Source** | Journal article(s) with results of the trial; Trial protocol |
| **Outcome** | ①②④⑤⑥⑦⑧⑩ | **Results** | ①②④⑤⑥⑦⑧⑩ | **Weight** | 1 |
| **Domain** | **Signalling question** | | | **Response** | **Comments** |
| **Bias arising from the randomization process** | 1.1 Was the allocation sequence random? | | | PY | Randomization was performed, but there was no mention of concealment. |
|  | 1.2 Was the allocation sequence concealed until participants were enrolled and assigned to interventions? | | | NI |  |
|  | 1.3 Did baseline differences between intervention groups suggest a problem with the randomization process? | | | N | Comparable at baseline |
|  | **Risk of bias judgement** | | | **Some concerns** |  |
| **Bias due to deviations from intended interventions** | 2.1.Were participants aware of their assigned intervention during the trial? | | | PN | Not explicitly mentioned |
|  | 2.2.Were carers and people delivering the interventions aware of participants' assigned intervention during the trial? | | | PN |  |
|  | 2.3. If Y/PY/NI to 2.1 or 2.2: Were there deviations from the intended intervention that arose because of the experimental context? | | | NA |  |
|  | 2.4 If Y/PY to 2.3: Were these deviations likely to have affected the outcome? | | | NA |  |
|  | 2.5. If Y/PY/NI to 2.4: Were these deviations from intended intervention balanced between groups? | | | NA |  |
|  | 2.6 Was an appropriate analysis used to estimate the effect of assignment to intervention? | | | PY |  |
|  | 2.7 If N/PN/NI to 2.6: Was there potential for a substantial impact (on the result) of the failure to analyse participants in the group to which they were randomized? | | | NA |  |
|  | **Risk of bias judgement** | | | **Low** |  |
| **Bias due to missing outcome data** | 3.1 Were data for this outcome available for all, or nearly all, participants randomized? | | | PY |  |
|  | 3.2 If N/PN/NI to 3.1: Is there evidence that result was not biased by missing outcome data? | | | NA |  |
|  | 3.3 If N/PN to 3.2: Could missingness in the outcome depend on its true value? | | | NA |  |
|  | 3.4 If Y/PY/NI to 3.3: Is it likely that missingness in the outcome depended on its true value? | | | NA |  |
|  | **Risk of bias judgement** | | | **Low** |  |
| **Bias in measurement of the outcome** | 4.1 Was the method of measuring the outcome inappropriate? | | | N | The measurement method is appropriate |
|  | 4.2 Could measurement or ascertainment of the outcome have differed between intervention groups? | | | PN | Outcome measurement or ascertainment did not differ significantly between the intervention groups |
|  | 4.3 Were outcome assessors aware of the intervention received by study participants? | | | NI | Not explicitly mentioned |
|  | 4.4 If Y/PY/NI to 4.3: Could assessment of the outcome have been influenced by knowledge of intervention received? | | | PN |  |
|  | 4.5 If Y/PY/NI to 4.4: Is it likely that assessment of the outcome was influenced by knowledge of intervention received? | | | NA |  |
|  | **Risk of bias judgement** | | | **Low** |  |
| **Bias in selection of the reported result** | 5.1 Were the data that produced this result analysed in accordance with a pre-specified analysis plan that was finalized before unblinded outcome data were available for analysis? | | | PY |  |
|  | 5.2 ... multiple eligible outcome measurements (e.g. scales, definitions, time points) within the outcome domain? | | | N |  |
|  | 5.3 ... multiple eligible analyses of the data? | | | N |  |
|  | **Risk of bias judgement** | | | **Low** |  |
| **Overall bias** | **Risk of bias judgement** | | | **Some concerns** |  |
| **Unique ID** | 8 | **Study ID** | NCT03242252 | **Assessor** | G and J |
| **Ref or Label** | 10.1111/dom.15019 | **Aim** | assignment to intervention (the 'intention-to-treat' effect) |  |  |
| **Experimental** | Sotagliflozin | **Comparator** | PBO | **Source** | Journal article(s) with results of the trial; Trial protocol |
| **Outcome** | ①②④⑥⑦⑧⑩ | **Results** | ①②④⑥⑦⑧⑩ | **Weight** | 1 |
| **Domain** | **Signalling question** | | | **Response** | **Comments** |
| **Bias arising from the randomization process** | 1.1 Was the allocation sequence random? | | | NI | The only information about the randomization method is that the study was randomized |
|  | 1.2 Was the allocation sequence concealed until participants were enrolled and assigned to interventions? | | | Y |  |
|  | 1.3 Did baseline differences between intervention groups suggest a problem with the randomization process? | | | N | The baseline was uniformly comparable |
|  | **Risk of bias judgement** | | | **Low** |  |
| **Bias due to deviations from intended interventions** | 2.1.Were participants aware of their assigned intervention during the trial? | | | N | This is a phase 3, multicentre, randomized, double-blind, placebocontrolled study  To maintain blinding, laboratory values such as fasting plasma glucose (FPG), HbA1c and urinary glucose values were assessed at a central laboratory and masked to study sites and patients from randomization until study end. |
|  | 2.2.Were carers and people delivering the interventions aware of participants' assigned intervention during the trial? | | | NI |  |
|  | 2.3. If Y/PY/NI to 2.1 or 2.2: Were there deviations from the intended intervention that arose because of the experimental context? | | | PN |  |
|  | 2.4 If Y/PY to 2.3: Were these deviations likely to have affected the outcome? | | | NA |  |
|  | 2.5. If Y/PY/NI to 2.4: Were these deviations from intended intervention balanced between groups? | | | NA |  |
|  | 2.6 Was an appropriate analysis used to estimate the effect of assignment to intervention? | | | PY |  |
|  | 2.7 If N/PN/NI to 2.6: Was there potential for a substantial impact (on the result) of the failure to analyse participants in the group to which they were randomized? | | | NA |  |
|  | **Risk of bias judgement** | | | **Low** |  |
| **Bias due to missing outcome data** | 3.1 Were data for this outcome available for all, or nearly all, participants randomized? | | | PY | Few were lost to follow-up. |
|  | 3.2 If N/PN/NI to 3.1: Is there evidence that result was not biased by missing outcome data? | | | NA |  |
|  | 3.3 If N/PN to 3.2: Could missingness in the outcome depend on its true value? | | | NA |  |
|  | 3.4 If Y/PY/NI to 3.3: Is it likely that missingness in the outcome depended on its true value? | | | NA |  |
|  | **Risk of bias judgement** | | | **Low** |  |
| **Bias in measurement of the outcome** | 4.1 Was the method of measuring the outcome inappropriate? | | | N |  |
|  | 4.2 Could measurement or ascertainment of the outcome have differed between intervention groups? | | | PN |  |
|  | 4.3 Were outcome assessors aware of the intervention received by study participants? | | | NI | Not explicitly mentioned |
|  | 4.4 If Y/PY/NI to 4.3: Could assessment of the outcome have been influenced by knowledge of intervention received? | | | PN |  |
|  | 4.5 If Y/PY/NI to 4.4: Is it likely that assessment of the outcome was influenced by knowledge of intervention received? | | | NA |  |
|  | **Risk of bias judgement** | | | **Low** |  |
| **Bias in selection of the reported result** | 5.1 Were the data that produced this result analysed in accordance with a pre-specified analysis plan that was finalized before unblinded outcome data were available for analysis? | | | PY |  |
|  | 5.2 ... multiple eligible outcome measurements (e.g. scales, definitions, time points) within the outcome domain? | | | PN |  |
|  | 5.3 ... multiple eligible analyses of the data? | | | PN |  |
|  | **Risk of bias judgement** | | | **Low** |  |
| **Overall bias** | **Risk of bias judgement** | | | **Low** |  |
| **Unique ID** | 9 | **Study ID** | NCT02914691 | **Assessor** | G and J |
| **Ref or Label** | 10.2337/dc22-1157 | **Aim** | assignment to intervention (the 'intention-to-treat' effect) |  |  |
| **Experimental** | Dapagliflozin | **Comparator** | PBO | **Source** | Journal article(s) with results of the trial; Trial protocol |
| **Outcome** | ①②③④⑤⑥ | **Results** | ①②③④⑤⑥ | **Weight** | 1 |
| **Domain** | **Signalling question** | | | **Response** | **Comments** |
| **Bias arising from the randomization process** | 1.1 Was the allocation sequence random? | | | NI | The only information about the randomization method is that the study was randomized  The Capital Region Pharmacy, Copenhagen, Denmark, performed the randomization and masking of study medication. |
|  | 1.2 Was the allocation sequence concealed until participants were enrolled and assigned to interventions? | | | Y |  |
|  | 1.3 Did baseline differences between intervention groups suggest a problem with the randomization process? | | | N | The baseline was uniformly comparable |
|  | **Risk of bias judgement** | | | **Low** |  |
| **Bias due to deviations from intended interventions** | 2.1.Were participants aware of their assigned intervention during the trial? | | | N | It's a double-blind, randomized, controlled, crossover trial.  Participants, study personnel, and investigators were all masked to study medication |
|  | 2.2.Were carers and people delivering the interventions aware of participants' assigned intervention during the trial? | | | N |  |
|  | 2.3. If Y/PY/NI to 2.1 or 2.2: Were there deviations from the intended intervention that arose because of the experimental context? | | | NA |  |
|  | 2.4 If Y/PY to 2.3: Were these deviations likely to have affected the outcome? | | | NA |  |
|  | 2.5. If Y/PY/NI to 2.4: Were these deviations from intended intervention balanced between groups? | | | NA |  |
|  | 2.6 Was an appropriate analysis used to estimate the effect of assignment to intervention? | | | Y |  |
|  | 2.7 If N/PN/NI to 2.6: Was there potential for a substantial impact (on the result) of the failure to analyse participants in the group to which they were randomized? | | | NA |  |
|  | **Risk of bias judgement** | | | **Low** |  |
| **Bias due to missing outcome data** | 3.1 Were data for this outcome available for all, or nearly all, participants randomized? | | | Y |  |
|  | 3.2 If N/PN/NI to 3.1: Is there evidence that result was not biased by missing outcome data? | | | NA |  |
|  | 3.3 If N/PN to 3.2: Could missingness in the outcome depend on its true value? | | | NA |  |
|  | 3.4 If Y/PY/NI to 3.3: Is it likely that missingness in the outcome depended on its true value? | | | NA |  |
|  | **Risk of bias judgement** | | | **Low** |  |
| **Bias in measurement of the outcome** | 4.1 Was the method of measuring the outcome inappropriate? | | | N |  |
|  | 4.2 Could measurement or ascertainment of the outcome have differed between intervention groups? | | | PN |  |
|  | 4.3 Were outcome assessors aware of the intervention received by study participants? | | | N | Participants, study personnel, and investigators were all masked to study medication. |
|  | 4.4 If Y/PY/NI to 4.3: Could assessment of the outcome have been influenced by knowledge of intervention received? | | | NA |  |
|  | 4.5 If Y/PY/NI to 4.4: Is it likely that assessment of the outcome was influenced by knowledge of intervention received? | | | NA |  |
|  | **Risk of bias judgement** | | | **Low** |  |
| **Bias in selection of the reported result** | 5.1 Were the data that produced this result analysed in accordance with a pre-specified analysis plan that was finalized before unblinded outcome data were available for analysis? | | | PY | The only information about the randomization method is that the study was randomized |
|  | 5.2 ... multiple eligible outcome measurements (e.g. scales, definitions, time points) within the outcome domain? | | | PN |  |
|  | 5.3 ... multiple eligible analyses of the data? | | | N |  |
|  | **Risk of bias judgement** | | | **Low** |  |
| **Overall bias** | **Risk of bias judgement** | | | **Low** |  |
| **Unique ID** | 10 | **Study ID** | UMIN000031454 | **Assessor** | G and J |
| **Ref or Label** | 10.1177/147916411878287 | **Aim** | assignment to intervention (the 'intention-to-treat' effect) |  |  |
| **Experimental** | Canagliflozin | **Comparator** | PBO | **Source** | Journal article(s) with results of the trial |
| **Outcome** | ①②④⑤ | **Results** | ①②④⑤ | **Weight** | 1 |
| **Domain** | **Signalling question** | | | **Response** | **Comments** |
| **Bias arising from the randomization process** | 1.1 Was the allocation sequence random? | | | NI | The only information about the randomization method is that the study was randomized |
|  | 1.2 Was the allocation sequence concealed until participants were enrolled and assigned to interventions? | | | NI |  |
|  | 1.3 Did baseline differences between intervention groups suggest a problem with the randomization process? | | | N | There were no significant differences between the two groups in baseline demographic, |
|  | **Risk of bias judgement** | | | **Some concerns** |  |
| **Bias due to deviations from intended interventions** | 2.1.Were participants aware of their assigned intervention during the trial? | | | N |  |
|  | 2.2.Were carers and people delivering the interventions aware of participants' assigned intervention during the trial? | | | PN |  |
|  | 2.3. If Y/PY/NI to 2.1 or 2.2: Were there deviations from the intended intervention that arose because of the experimental context? | | | NA |  |
|  | 2.4 If Y/PY to 2.3: Were these deviations likely to have affected the outcome? | | | NA |  |
|  | 2.5. If Y/PY/NI to 2.4: Were these deviations from intended intervention balanced between groups? | | | NA |  |
|  | 2.6 Was an appropriate analysis used to estimate the effect of assignment to intervention? | | | PY |  |
|  | 2.7 If N/PN/NI to 2.6: Was there potential for a substantial impact (on the result) of the failure to analyse participants in the group to which they were randomized? | | | NA |  |
|  | **Risk of bias judgement** | | | **Low** |  |
| **Bias due to missing outcome data** | 3.1 Were data for this outcome available for all, or nearly all, participants randomized? | | | Y | Only 2 patients were lost to follow-up. |
|  | 3.2 If N/PN/NI to 3.1: Is there evidence that result was not biased by missing outcome data? | | | NA |  |
|  | 3.3 If N/PN to 3.2: Could missingness in the outcome depend on its true value? | | | NA |  |
|  | 3.4 If Y/PY/NI to 3.3: Is it likely that missingness in the outcome depended on its true value? | | | NA |  |
|  | **Risk of bias judgement** | | | **Low** |  |
| **Bias in measurement of the outcome** | 4.1 Was the method of measuring the outcome inappropriate? | | | N |  |
|  | 4.2 Could measurement or ascertainment of the outcome have differed between intervention groups? | | | PN |  |
|  | 4.3 Were outcome assessors aware of the intervention received by study participants? | | | NI | Not explicitly mentioned |
|  | 4.4 If Y/PY/NI to 4.3: Could assessment of the outcome have been influenced by knowledge of intervention received? | | | N | All of them were clear indicators |
|  | 4.5 If Y/PY/NI to 4.4: Is it likely that assessment of the outcome was influenced by knowledge of intervention received? | | | NA |  |
|  | **Risk of bias judgement** | | | **Low** |  |
| **Bias in selection of the reported result** | 5.1 Were the data that produced this result analysed in accordance with a pre-specified analysis plan that was finalized before unblinded outcome data were available for analysis? | | | PY |  |
|  | 5.2 ... multiple eligible outcome measurements (e.g. scales, definitions, time points) within the outcome domain? | | | N | All of them were clear indicators |
|  | 5.3 ... multiple eligible analyses of the data? | | | N |  |
|  | **Risk of bias judgement** | | | **Low** |  |
| **Overall bias** | **Risk of bias judgement** | | | **Some concerns** |  |
| **Unique ID** | 11 | **Study ID** | NCT04061200 | **Assessor** | G and J |
| **Ref or Label** | 10.1111/dom.15287 | **Aim** | assignment to intervention (the 'intention-to-treat' effect) |  |  |
| **Experimental** | Semaglutide | **Comparator** | PBO | **Source** | Journal article(s) with results of the trial; Trial protocol; Non-commercial trial registry record (e.g. ClinicalTrials.gov record) |
| **Outcome** | ①②⑥⑦⑧ | **Results** | ①②⑥⑦⑧ | **Weight** | 1 |
| **Domain** | **Signalling question** | | | **Response** | **Comments** |
| **Bias arising from the randomization process** | 1.1 Was the allocation sequence random? | | | Y | One person not involved in the study had access to the computergenerated random semaglutide/placebo allocation sequence. |
|  | 1.2 Was the allocation sequence concealed until participants were enrolled and assigned to interventions? | | | Y |  |
|  | 1.3 Did baseline differences between intervention groups suggest a problem with the randomization process? | | | N |  |
|  | **Risk of bias judgement** | | | **Low** |  |
| **Bias due to deviations from intended interventions** | 2.1.Were participants aware of their assigned intervention during the trial? | | | Y | It is a randomized, double-blind, placebo-controlled, parallelgroup. |
|  | 2.2.Were carers and people delivering the interventions aware of participants' assigned intervention during the trial? | | | PN |  |
|  | 2.3. If Y/PY/NI to 2.1 or 2.2: Were there deviations from the intended intervention that arose because of the experimental context? | | | PN |  |
|  | 2.4 If Y/PY to 2.3: Were these deviations likely to have affected the outcome? | | | NA |  |
|  | 2.5. If Y/PY/NI to 2.4: Were these deviations from intended intervention balanced between groups? | | | NA |  |
|  | 2.6 Was an appropriate analysis used to estimate the effect of assignment to intervention? | | | PY |  |
|  | 2.7 If N/PN/NI to 2.6: Was there potential for a substantial impact (on the result) of the failure to analyse participants in the group to which they were randomized? | | | NA |  |
|  | **Risk of bias judgement** | | | **Low** |  |
| **Bias due to missing outcome data** | 3.1 Were data for this outcome available for all, or nearly all, participants randomized? | | | Y | More than 95% of the patients completed the trial |
|  | 3.2 If N/PN/NI to 3.1: Is there evidence that result was not biased by missing outcome data? | | | NA |  |
|  | 3.3 If N/PN to 3.2: Could missingness in the outcome depend on its true value? | | | NA |  |
|  | 3.4 If Y/PY/NI to 3.3: Is it likely that missingness in the outcome depended on its true value? | | | NA |  |
|  | **Risk of bias judgement** | | | **Low** |  |
| **Bias in measurement of the outcome** | 4.1 Was the method of measuring the outcome inappropriate? | | | N |  |
|  | 4.2 Could measurement or ascertainment of the outcome have differed between intervention groups? | | | PN | The inspection indicators are clear |
|  | 4.3 Were outcome assessors aware of the intervention received by study participants? | | | Y | Investigators, participants and treating physicians were blinded to treatment allocation. |
|  | 4.4 If Y/PY/NI to 4.3: Could assessment of the outcome have been influenced by knowledge of intervention received? | | | PN | The inspection indicators are clear |
|  | 4.5 If Y/PY/NI to 4.4: Is it likely that assessment of the outcome was influenced by knowledge of intervention received? | | | NA |  |
|  | **Risk of bias judgement** | | | **Low** |  |
| **Bias in selection of the reported result** | 5.1 Were the data that produced this result analysed in accordance with a pre-specified analysis plan that was finalized before unblinded outcome data were available for analysis? | | | Y |  |
|  | 5.2 ... multiple eligible outcome measurements (e.g. scales, definitions, time points) within the outcome domain? | | | PN |  |
|  | 5.3 ... multiple eligible analyses of the data? | | | PN |  |
|  | **Risk of bias judgement** | | | **Low** |  |
| **Overall bias** | **Risk of bias judgement** | | | **Low** |  |
| **Unique ID** | 12 | **Study ID** | NCT02547935 | **Assessor** | G and J |
| **Ref or Label** | 10.1016/S2213-8587（19）30086-5 | **Aim** | assignment to intervention (the 'intention-to-treat' effect) |  |  |
| **Experimental** | Dapagliflozin | **Comparator** | PBO | **Source** | Journal article(s) with results of the trial; Trial protocol; Non-commercial trial registry record (e.g. ClinicalTrials.gov record) |
| **Outcome** | ①②③④⑦⑧⑩ | **Results** | ①②③④⑦⑧⑩ | **Weight** | 1 |
| **Domain** | **Signalling question** | | | **Response** | **Comments** |
| **Bias arising from the randomization process** | 1.1 Was the allocation sequence random? | | | Y | The investigators at the study centres assigned each patient according to an identification number provided by the interactive voice–web response system. In each phase, medications for each treatment group were supplied in identical bottles labelled appropriately to maintain masking within the study. |
|  | 1.2 Was the allocation sequence concealed until participants were enrolled and assigned to interventions? | | | Y |  |
|  | 1.3 Did baseline differences between intervention groups suggest a problem with the randomization process? | | | N | Baseline demographic, clinical, and biochemical characteristics, including concomitant medications, were similar between the treatment groups |
|  | **Risk of bias judgement** | | | **Low** |  |
| **Bias due to deviations from intended interventions** | 2.1.Were participants aware of their assigned intervention during the trial? | | | N | Participants, treating clinicians, and all study personnel (apart from personnel analysing pharmacokinetic data) were masked to the assigned treatment group |
|  | 2.2.Were carers and people delivering the interventions aware of participants' assigned intervention during the trial? | | | N |  |
|  | 2.3. If Y/PY/NI to 2.1 or 2.2: Were there deviations from the intended intervention that arose because of the experimental context? | | | NA |  |
|  | 2.4 If Y/PY to 2.3: Were these deviations likely to have affected the outcome? | | | NA |  |
|  | 2.5. If Y/PY/NI to 2.4: Were these deviations from intended intervention balanced between groups? | | | NA |  |
|  | 2.6 Was an appropriate analysis used to estimate the effect of assignment to intervention? | | | PY |  |
|  | 2.7 If N/PN/NI to 2.6: Was there potential for a substantial impact (on the result) of the failure to analyse participants in the group to which they were randomized? | | | NA |  |
|  | **Risk of bias judgement** | | | **Low** |  |
| **Bias due to missing outcome data** | 3.1 Were data for this outcome available for all, or nearly all, participants randomized? | | | Y | More than 95% of the subjects completed the trial |
|  | 3.2 If N/PN/NI to 3.1: Is there evidence that result was not biased by missing outcome data? | | | NA |  |
|  | 3.3 If N/PN to 3.2: Could missingness in the outcome depend on its true value? | | | NA |  |
|  | 3.4 If Y/PY/NI to 3.3: Is it likely that missingness in the outcome depended on its true value? | | | NA |  |
|  | **Risk of bias judgement** | | | **Low** |  |
| **Bias in measurement of the outcome** | 4.1 Was the method of measuring the outcome inappropriate? | | | N |  |
|  | 4.2 Could measurement or ascertainment of the outcome have differed between intervention groups? | | | PN |  |
|  | 4.3 Were outcome assessors aware of the intervention received by study participants? | | | N | Participants, treating clinicians, and all study personnel (apart from personnel analysing pharmacokinetic data) were masked to the assigned treatment group |
|  | 4.4 If Y/PY/NI to 4.3: Could assessment of the outcome have been influenced by knowledge of intervention received? | | | NA |  |
|  | 4.5 If Y/PY/NI to 4.4: Is it likely that assessment of the outcome was influenced by knowledge of intervention received? | | | NA |  |
|  | **Risk of bias judgement** | | | **Low** |  |
| **Bias in selection of the reported result** | 5.1 Were the data that produced this result analysed in accordance with a pre-specified analysis plan that was finalized before unblinded outcome data were available for analysis? | | | PY |  |
|  | 5.2 ... multiple eligible outcome measurements (e.g. scales, definitions, time points) within the outcome domain? | | | PN |  |
|  | 5.3 ... multiple eligible analyses of the data? | | | PN |  |
|  | **Risk of bias judgement** | | | **Low** |  |
| **Overall bias** | **Risk of bias judgement** | | | **Low** |  |
| **Unique ID** | 13 | **Study ID** | NCT02540993 | **Assessor** | G and J |
| **Ref or Label** | 10.1159/000532102 | **Aim** | assignment to intervention (the 'intention-to-treat' effect) |  |  |
| **Experimental** | Finerenone | **Comparator** | PBO | **Source** | Journal article(s) with results of the trial; Trial protocol; Non-commercial trial registry record (e.g. ClinicalTrials.gov record) |
| **Outcome** | ④⑥⑧⑨⑩ | **Results** | ④⑥⑧⑨⑩ | **Weight** | 1 |
| **Domain** | **Signalling question** | | | **Response** | **Comments** |
| **Bias arising from the randomization process** | 1.1 Was the allocation sequence random? | | | Y | Interactive voice response system/interactive Web response system |
|  | 1.2 Was the allocation sequence concealed until participants were enrolled and assigned to interventions? | | | Y |  |
|  | 1.3 Did baseline differences between intervention groups suggest a problem with the randomization process? | | | N | The baseline was uniformly comparable |
|  | **Risk of bias judgement** | | | **Low** |  |
| **Bias due to deviations from intended interventions** | 2.1.Were participants aware of their assigned intervention during the trial? | | | N | Masking：Quadruple (Participant, Care Provider, Investigator, Outcomes Assessor) |
|  | 2.2.Were carers and people delivering the interventions aware of participants' assigned intervention during the trial? | | | N |  |
|  | 2.3. If Y/PY/NI to 2.1 or 2.2: Were there deviations from the intended intervention that arose because of the experimental context? | | | NA |  |
|  | 2.4 If Y/PY to 2.3: Were these deviations likely to have affected the outcome? | | | NA |  |
|  | 2.5. If Y/PY/NI to 2.4: Were these deviations from intended intervention balanced between groups? | | | NA |  |
|  | 2.6 Was an appropriate analysis used to estimate the effect of assignment to intervention? | | | Y |  |
|  | 2.7 If N/PN/NI to 2.6: Was there potential for a substantial impact (on the result) of the failure to analyse participants in the group to which they were randomized? | | | NA |  |
|  | **Risk of bias judgement** | | | **Low** |  |
| **Bias due to missing outcome data** | 3.1 Were data for this outcome available for all, or nearly all, participants randomized? | | | PY |  |
|  | 3.2 If N/PN/NI to 3.1: Is there evidence that result was not biased by missing outcome data? | | | NA |  |
|  | 3.3 If N/PN to 3.2: Could missingness in the outcome depend on its true value? | | | NA |  |
|  | 3.4 If Y/PY/NI to 3.3: Is it likely that missingness in the outcome depended on its true value? | | | NA |  |
|  | **Risk of bias judgement** | | | **Low** |  |
| **Bias in measurement of the outcome** | 4.1 Was the method of measuring the outcome inappropriate? | | | N |  |
|  | 4.2 Could measurement or ascertainment of the outcome have differed between intervention groups? | | | PN |  |
|  | 4.3 Were outcome assessors aware of the intervention received by study participants? | | | N | Masking：Quadruple (Participant, Care Provider, Investigator, Outcomes Assessor) |
|  | 4.4 If Y/PY/NI to 4.3: Could assessment of the outcome have been influenced by knowledge of intervention received? | | | NA |  |
|  | 4.5 If Y/PY/NI to 4.4: Is it likely that assessment of the outcome was influenced by knowledge of intervention received? | | | NA |  |
|  | **Risk of bias judgement** | | | **Low** |  |
| **Bias in selection of the reported result** | 5.1 Were the data that produced this result analysed in accordance with a pre-specified analysis plan that was finalized before unblinded outcome data were available for analysis? | | | PY |  |
|  | 5.2 ... multiple eligible outcome measurements (e.g. scales, definitions, time points) within the outcome domain? | | | PN |  |
|  | 5.3 ... multiple eligible analyses of the data? | | | PN |  |
|  | **Risk of bias judgement** | | | **Low** |  |
| **Overall bias** | **Risk of bias judgement** | | | **Low** |  |
| **Unique ID** | 14 | **Study ID** | NCT00637273, NCT00676338,NCT02229383, NCT02229396, NCT00641056,NCT01652729, NCT00935532, NCT01003184. | **Assessor** | G and J |
| **Ref or Label** | 10.1007/s13300-020-00815-z | **Aim** | assignment to intervention (the 'intention-to-treat' effect) |  |  |
| **Experimental** | Exenatide | **Comparator** | PBO | **Source** | Journal article(s) with results of the trial; Trial protocol; Non-commercial trial registry record (e.g. ClinicalTrials.gov record) |
| **Outcome** | ②⑦⑨⑩ | **Results** | ②⑦⑨⑩ | **Weight** | 1 |
| **Domain** | **Signalling question** | | | **Response** | **Comments** |
| **Bias arising from the randomization process** | 1.1 Was the allocation sequence random? | | | PY | This post hoc analysis evaluated the pooled safety and efficacy data from eight previously published double-blind or open-label studies |
|  | 1.2 Was the allocation sequence concealed until participants were enrolled and assigned to interventions? | | | PY |  |
|  | 1.3 Did baseline differences between intervention groups suggest a problem with the randomization process? | | | N |  |
|  | **Risk of bias judgement** | | | **Low** |  |
| **Bias due to deviations from intended interventions** | 2.1.Were participants aware of their assigned intervention during the trial? | | | N | This post hoc analysis evaluated the pooled safety and efficacy data from eight previously published double-blind or open-label studies |
|  | 2.2.Were carers and people delivering the interventions aware of participants' assigned intervention during the trial? | | | N |  |
|  | 2.3. If Y/PY/NI to 2.1 or 2.2: Were there deviations from the intended intervention that arose because of the experimental context? | | | NA |  |
|  | 2.4 If Y/PY to 2.3: Were these deviations likely to have affected the outcome? | | | NA |  |
|  | 2.5. If Y/PY/NI to 2.4: Were these deviations from intended intervention balanced between groups? | | | NA |  |
|  | 2.6 Was an appropriate analysis used to estimate the effect of assignment to intervention? | | | PY |  |
|  | 2.7 If N/PN/NI to 2.6: Was there potential for a substantial impact (on the result) of the failure to analyse participants in the group to which they were randomized? | | | NA |  |
|  | **Risk of bias judgement** | | | **Low** |  |
| **Bias due to missing outcome data** | 3.1 Were data for this outcome available for all, or nearly all, participants randomized? | | | PY |  |
|  | 3.2 If N/PN/NI to 3.1: Is there evidence that result was not biased by missing outcome data? | | | NA |  |
|  | 3.3 If N/PN to 3.2: Could missingness in the outcome depend on its true value? | | | NA |  |
|  | 3.4 If Y/PY/NI to 3.3: Is it likely that missingness in the outcome depended on its true value? | | | NA |  |
|  | **Risk of bias judgement** | | | **Low** |  |
| **Bias in measurement of the outcome** | 4.1 Was the method of measuring the outcome inappropriate? | | | N | The measurement method is appropriate |
|  | 4.2 Could measurement or ascertainment of the outcome have differed between intervention groups? | | | PN | The measurement method is appropriate |
|  | 4.3 Were outcome assessors aware of the intervention received by study participants? | | | N | Part of the study was double-blind, non-four-blind |
|  | 4.4 If Y/PY/NI to 4.3: Could assessment of the outcome have been influenced by knowledge of intervention received? | | | NA |  |
|  | 4.5 If Y/PY/NI to 4.4: Is it likely that assessment of the outcome was influenced by knowledge of intervention received? | | | NA |  |
|  | **Risk of bias judgement** | | | **Low** |  |
| **Bias in selection of the reported result** | 5.1 Were the data that produced this result analysed in accordance with a pre-specified analysis plan that was finalized before unblinded outcome data were available for analysis? | | | PY | It is mentioned in the article or in the study protocol |
|  | 5.2 ... multiple eligible outcome measurements (e.g. scales, definitions, time points) within the outcome domain? | | | PN | The outcome measures are clear |
|  | 5.3 ... multiple eligible analyses of the data? | | | PN | The outcome measures are clear |
|  | **Risk of bias judgement** | | | **Low** |  |
| **Overall bias** | **Risk of bias judgement** | | | **Low** |  |
| **Unique ID** | 15 | **Study ID** | NCT02413398 | **Assessor** | G and J |
| **Ref or Label** | 10.1111/dom.13413 | **Aim** | assignment to intervention (the 'intention-to-treat' effect) |  |  |
| **Experimental** | Dapagliflozin | **Comparator** | PBO | **Source** | Company-owned trial registry record (e.g. GSK Clinical Study Register record); Conference abstract(s) about the trial |
| **Outcome** | ①②④⑥⑦⑧⑩ | **Results** | ①②④⑥⑦⑧⑩ | **Weight** | 1 |
| **Domain** | **Signalling question** | | | **Response** | **Comments** |
| **Bias arising from the randomization process** | 1.1 Was the allocation sequence random? | | | Y | Eligible patients were assigned a unique randomization code using an interactive voice response system (IVRS) or interactive web response system (IWRS). |
|  | 1.2 Was the allocation sequence concealed until participants were enrolled and assigned to interventions? | | | Y |  |
|  | 1.3 Did baseline differences between intervention groups suggest a problem with the randomization process? | | | N | Demographics and baseline characteristics were balanced between treatment groups |
|  | **Risk of bias judgement** | | | **Low** |  |
| **Bias due to deviations from intended interventions** | 2.1.Were participants aware of their assigned intervention during the trial? | | | N | Masking：Double (Participant, Investigator) |
|  | 2.2.Were carers and people delivering the interventions aware of participants' assigned intervention during the trial? | | | NI |  |
|  | 2.3. If Y/PY/NI to 2.1 or 2.2: Were there deviations from the intended intervention that arose because of the experimental context? | | | NI | It is not explicitly mentioned in the article |
|  | 2.4 If Y/PY to 2.3: Were these deviations likely to have affected the outcome? | | | NA |  |
|  | 2.5. If Y/PY/NI to 2.4: Were these deviations from intended intervention balanced between groups? | | | NA |  |
|  | 2.6 Was an appropriate analysis used to estimate the effect of assignment to intervention? | | | PY | There was no group assignment according to treatment, and eligible participants after randomization were not excluded. |
|  | 2.7 If N/PN/NI to 2.6: Was there potential for a substantial impact (on the result) of the failure to analyse participants in the group to which they were randomized? | | | NA |  |
|  | **Risk of bias judgement** | | | **Some concerns** |  |
| **Bias due to missing outcome data** | 3.1 Were data for this outcome available for all, or nearly all, participants randomized? | | | Y | 97.5% of the subjects completed the trial |
|  | 3.2 If N/PN/NI to 3.1: Is there evidence that result was not biased by missing outcome data? | | | NA |  |
|  | 3.3 If N/PN to 3.2: Could missingness in the outcome depend on its true value? | | | NA |  |
|  | 3.4 If Y/PY/NI to 3.3: Is it likely that missingness in the outcome depended on its true value? | | | NA |  |
|  | **Risk of bias judgement** | | | **Low** |  |
| **Bias in measurement of the outcome** | 4.1 Was the method of measuring the outcome inappropriate? | | | N | The measurement method is appropriate |
|  | 4.2 Could measurement or ascertainment of the outcome have differed between intervention groups? | | | PN | The measures were the same across the intervention groups |
|  | 4.3 Were outcome assessors aware of the intervention received by study participants? | | | PY | Masking:Double (Participant, Investigator) |
|  | 4.4 If Y/PY/NI to 4.3: Could assessment of the outcome have been influenced by knowledge of intervention received? | | | PN | In this paper, the judgment of outcome indicators is more rigorous |
|  | 4.5 If Y/PY/NI to 4.4: Is it likely that assessment of the outcome was influenced by knowledge of intervention received? | | | NA |  |
|  | **Risk of bias judgement** | | | **Low** |  |
| **Bias in selection of the reported result** | 5.1 Were the data that produced this result analysed in accordance with a pre-specified analysis plan that was finalized before unblinded outcome data were available for analysis? | | | PY | The study plan and statistical analysis were rigorous |
|  | 5.2 ... multiple eligible outcome measurements (e.g. scales, definitions, time points) within the outcome domain? | | | PN | The judgment method of outcome indicators in this paper is relatively clear |
|  | 5.3 ... multiple eligible analyses of the data? | | | PN | The judgment method of outcome indicators in this paper is relatively clear |
|  | **Risk of bias judgement** | | | **Low** |  |
| **Overall bias** | **Risk of bias judgement** | | | **Some concerns** |  |
| **Unique ID** | 16 | **Study ID** | NCT00663260 | **Assessor** | G and J |
| **Ref or Label** | 10.1038/ki.2013.356 | **Aim** | assignment to intervention (the 'intention-to-treat' effect) |  |  |
| **Experimental** | Dapagliflozin | **Comparator** | PBO | **Source** | Journal article(s) with results of the trial; Non-commercial trial registry record (e.g. ClinicalTrials.gov record) |
| **Outcome** | ①②④⑤⑥⑦⑧⑩ | **Results** | ①②④⑤⑥⑦⑧⑩ | **Weight** | 1 |
| **Domain** | **Signalling question** | | | **Response** | **Comments** |
| **Bias arising from the randomization process** | 1.1 Was the allocation sequence random? | | | NI | The only information about the randomization method is that the study was randomized  The article makes no mention of concealment. |
|  | 1.2 Was the allocation sequence concealed until participants were enrolled and assigned to interventions? | | | NI |  |
|  | 1.3 Did baseline differences between intervention groups suggest a problem with the randomization process? | | | PN | It is not explicitly mentioned |
|  | **Risk of bias judgement** | | | **Some concerns** |  |
| **Bias due to deviations from intended interventions** | 2.1.Were participants aware of their assigned intervention during the trial? | | | N | Masking: Double (Participant, Investigator) |
|  | 2.2.Were carers and people delivering the interventions aware of participants' assigned intervention during the trial? | | | NI |  |
|  | 2.3. If Y/PY/NI to 2.1 or 2.2: Were there deviations from the intended intervention that arose because of the experimental context? | | | NI | It is not explicitly mentioned |
|  | 2.4 If Y/PY to 2.3: Were these deviations likely to have affected the outcome? | | | NA |  |
|  | 2.5. If Y/PY/NI to 2.4: Were these deviations from intended intervention balanced between groups? | | | NA |  |
|  | 2.6 Was an appropriate analysis used to estimate the effect of assignment to intervention? | | | PY | Participants who did not receive the assigned intervention were not excluded, participants were not assigned according to the intervention they received, and eligible trial participants after randomization were not excluded |
|  | 2.7 If N/PN/NI to 2.6: Was there potential for a substantial impact (on the result) of the failure to analyse participants in the group to which they were randomized? | | | NA |  |
|  | **Risk of bias judgement** | | | **Some concerns** |  |
| **Bias due to missing outcome data** | 3.1 Were data for this outcome available for all, or nearly all, participants randomized? | | | PY |  |
|  | 3.2 If N/PN/NI to 3.1: Is there evidence that result was not biased by missing outcome data? | | | NA |  |
|  | 3.3 If N/PN to 3.2: Could missingness in the outcome depend on its true value? | | | NA |  |
|  | 3.4 If Y/PY/NI to 3.3: Is it likely that missingness in the outcome depended on its true value? | | | NA |  |
|  | **Risk of bias judgement** | | | **Low** |  |
| **Bias in measurement of the outcome** | 4.1 Was the method of measuring the outcome inappropriate? | | | N | The measurement tools and methods were clear |
|  | 4.2 Could measurement or ascertainment of the outcome have differed between intervention groups? | | | PN | The measurement tools and methods were clear |
|  | 4.3 Were outcome assessors aware of the intervention received by study participants? | | | NI | Masking: Double (Participant, Investigator) |
|  | 4.4 If Y/PY/NI to 4.3: Could assessment of the outcome have been influenced by knowledge of intervention received? | | | PN | In this paper, the judgment of outcome indicators is more rigorous |
|  | 4.5 If Y/PY/NI to 4.4: Is it likely that assessment of the outcome was influenced by knowledge of intervention received? | | | NA |  |
|  | **Risk of bias judgement** | | | **Low** |  |
| **Bias in selection of the reported result** | 5.1 Were the data that produced this result analysed in accordance with a pre-specified analysis plan that was finalized before unblinded outcome data were available for analysis? | | | PY | The description of statistical methods is relatively clear |
|  | 5.2 ... multiple eligible outcome measurements (e.g. scales, definitions, time points) within the outcome domain? | | | PN | The measurement methods of outcome indicators in this article are relatively clear, and the article does not mention multiple measurement |
|  | 5.3 ... multiple eligible analyses of the data? | | | PN | Statistical methods have been clearly described |
|  | **Risk of bias judgement** | | | **Low** |  |
| **Overall bias** | **Risk of bias judgement** | | | **Some concerns** |  |
| **Unique ID** | 17 | **Study ID** | NCT02836873 | **Assessor** | G and J |
| **Ref or Label** | 10.1053/j.ajkd.2019.03.417 | **Aim** | assignment to intervention (the 'intention-to-treat' effect) |  |  |
| **Experimental** | Bexagliflozin | **Comparator** | PBO | **Source** | Journal article(s) with results of the trial; Trial protocol; Non-commercial trial registry record (e.g. ClinicalTrials.gov record) |
| **Outcome** | ①②④⑥⑦⑧⑨ | **Results** | ①②④⑥⑦⑧⑨ | **Weight** | 1 |
| **Domain** | **Signalling question** | | | **Response** | **Comments** |
| **Bias arising from the randomization process** | 1.1 Was the allocation sequence random? | | | Y | Randomization and study drug allocation were performed through a central interactive web response system. |
|  | 1.2 Was the allocation sequence concealed until participants were enrolled and assigned to interventions? | | | Y |  |
|  | 1.3 Did baseline differences between intervention groups suggest a problem with the randomization process? | | | N | Baseline characteristics, including variables used to stratify randomization , and early withdrawal rates were similar between treatment groups. |
|  | **Risk of bias judgement** | | | **Low** |  |
| **Bias due to deviations from intended interventions** | 2.1.Were participants aware of their assigned intervention during the trial? | | | N | Masking: Triple (Participant, Investigator, Outcomes Assessor) |
|  | 2.2.Were carers and people delivering the interventions aware of participants' assigned intervention during the trial? | | | PN |  |
|  | 2.3. If Y/PY/NI to 2.1 or 2.2: Were there deviations from the intended intervention that arose because of the experimental context? | | | NA |  |
|  | 2.4 If Y/PY to 2.3: Were these deviations likely to have affected the outcome? | | | NA |  |
|  | 2.5. If Y/PY/NI to 2.4: Were these deviations from intended intervention balanced between groups? | | | NA |  |
|  | 2.6 Was an appropriate analysis used to estimate the effect of assignment to intervention? | | | Y | Effectiveness analyses were performed in an intention-totreat manner. |
|  | 2.7 If N/PN/NI to 2.6: Was there potential for a substantial impact (on the result) of the failure to analyse participants in the group to which they were randomized? | | | NA |  |
|  | **Risk of bias judgement** | | | **Low** |  |
| **Bias due to missing outcome data** | 3.1 Were data for this outcome available for all, or nearly all, participants randomized? | | | PY | 94.8% of the subjects completed the study |
|  | 3.2 If N/PN/NI to 3.1: Is there evidence that result was not biased by missing outcome data? | | | NA |  |
|  | 3.3 If N/PN to 3.2: Could missingness in the outcome depend on its true value? | | | NA |  |
|  | 3.4 If Y/PY/NI to 3.3: Is it likely that missingness in the outcome depended on its true value? | | | NA |  |
|  | **Risk of bias judgement** | | | **Low** |  |
| **Bias in measurement of the outcome** | 4.1 Was the method of measuring the outcome inappropriate? | | | PN | The measurement method is relatively clear |
|  | 4.2 Could measurement or ascertainment of the outcome have differed between intervention groups? | | | PN | The measurement method is relatively clear |
|  | 4.3 Were outcome assessors aware of the intervention received by study participants? | | | N | Masking: Triple (Participant, Investigator, Outcomes Assessor) |
|  | 4.4 If Y/PY/NI to 4.3: Could assessment of the outcome have been influenced by knowledge of intervention received? | | | NA |  |
|  | 4.5 If Y/PY/NI to 4.4: Is it likely that assessment of the outcome was influenced by knowledge of intervention received? | | | NA |  |
|  | **Risk of bias judgement** | | | **Low** |  |
| **Bias in selection of the reported result** | 5.1 Were the data that produced this result analysed in accordance with a pre-specified analysis plan that was finalized before unblinded outcome data were available for analysis? | | | PY | The data analysis method is described in detail |
|  | 5.2 ... multiple eligible outcome measurements (e.g. scales, definitions, time points) within the outcome domain? | | | PN | The measurement methods of outcome indicators in this article are relatively clear, and the article does not mention multiple measurement |
|  | 5.3 ... multiple eligible analyses of the data? | | | PN | Statistical methods have been clearly described |
|  | **Risk of bias judgement** | | | **Low** |  |
| **Overall bias** | **Risk of bias judgement** | | | **Low** |  |
| **Unique ID** | 18 | **Study ID** | 18 | **Assessor** | G and J |
| **Ref or Label** | 10.1016/j.clinthera.2015.10.025 | **Aim** | assignment to intervention (the 'intention-to-treat' effect) |  |  |
| **Experimental** | Luseogliflozin | **Comparator** | PBO | **Source** | Journal article(s) with results of the trial; Trial protocol |
| **Outcome** | ①②③④⑤⑥⑦⑧⑩ | **Results** | ①②③④⑤⑥⑦⑧⑩ | **Weight** | 1 |
| **Domain** | **Signalling question** | | | **Response** | **Comments** |
| **Bias arising from the randomization process** | 1.1 Was the allocation sequence random? | | | NI | The only information about the randomization method is that the study was randomized  The article makes no mention of concealment. |
|  | 1.2 Was the allocation sequence concealed until participants were enrolled and assigned to interventions? | | | NI |  |
|  | 1.3 Did baseline differences between intervention groups suggest a problem with the randomization process? | | | N | The demographic and baseline characteristics of patients were balanced between both groups. |
|  | **Risk of bias judgement** | | | **Some concerns** |  |
| **Bias due to deviations from intended interventions** | 2.1.Were participants aware of their assigned intervention during the trial? | | | N | This is a double-blind study |
|  | 2.2.Were carers and people delivering the interventions aware of participants' assigned intervention during the trial? | | | NI |  |
|  | 2.3. If Y/PY/NI to 2.1 or 2.2: Were there deviations from the intended intervention that arose because of the experimental context? | | | PN |  |
|  | 2.4 If Y/PY to 2.3: Were these deviations likely to have affected the outcome? | | | NA |  |
|  | 2.5. If Y/PY/NI to 2.4: Were these deviations from intended intervention balanced between groups? | | | NA |  |
|  | 2.6 Was an appropriate analysis used to estimate the effect of assignment to intervention? | | | PY | The article is not explicitly mentioned, and the context is available |
|  | 2.7 If N/PN/NI to 2.6: Was there potential for a substantial impact (on the result) of the failure to analyse participants in the group to which they were randomized? | | | NA |  |
|  | **Risk of bias judgement** | | | **Low** |  |
| **Bias due to missing outcome data** | 3.1 Were data for this outcome available for all, or nearly all, participants randomized? | | | PY | 93.43% of the subjects completed the study |
|  | 3.2 If N/PN/NI to 3.1: Is there evidence that result was not biased by missing outcome data? | | | NA |  |
|  | 3.3 If N/PN to 3.2: Could missingness in the outcome depend on its true value? | | | NA |  |
|  | 3.4 If Y/PY/NI to 3.3: Is it likely that missingness in the outcome depended on its true value? | | | NA |  |
|  | **Risk of bias judgement** | | | **Low** |  |
| **Bias in measurement of the outcome** | 4.1 Was the method of measuring the outcome inappropriate? | | | PN | The method of outcome measures was appropriate for the outcome of the assessment. |
|  | 4.2 Could measurement or ascertainment of the outcome have differed between intervention groups? | | | PN | The method of outcome measures was appropriate for the outcome of the assessment. |
|  | 4.3 Were outcome assessors aware of the intervention received by study participants? | | | NI | It is not explicitly mentioned. |
|  | 4.4 If Y/PY/NI to 4.3: Could assessment of the outcome have been influenced by knowledge of intervention received? | | | PN | The evaluation method of outcome indicators in this article is relatively clear |
|  | 4.5 If Y/PY/NI to 4.4: Is it likely that assessment of the outcome was influenced by knowledge of intervention received? | | | NA |  |
|  | **Risk of bias judgement** | | | **Low** |  |
| **Bias in selection of the reported result** | 5.1 Were the data that produced this result analysed in accordance with a pre-specified analysis plan that was finalized before unblinded outcome data were available for analysis? | | | PY | The statistical methods described in this article are relatively clear |
|  | 5.2 ... multiple eligible outcome measurements (e.g. scales, definitions, time points) within the outcome domain? | | | PN | The statistical methods described in this article are relatively clear |
|  | 5.3 ... multiple eligible analyses of the data? | | | PN | The statistical methods described in this article are relatively clear |
|  | **Risk of bias judgement** | | | **Low** |  |
| **Overall bias** | **Risk of bias judgement** | | | **Some concerns** |  |
| **Unique ID** | 19 | **Study ID** | NCT03594110 NCT00885118 NCT00789035 NCT00558571 NCT00749190 NCT01011868 NCT01193218 NCT01210001 NCT01177813 NCT01159600 NCT01289990 NCT01131676 NCT01164501 NCT01370005 NCT01306214 NCT01649297 NCT01947855 NCT02589639 | **Assessor** | G and J |
| **Ref or Label** | 10.2337/dc21-2034 | **Aim** | assignment to intervention (the 'intention-to-treat' effect) |  |  |
| **Experimental** | Empagliflozin | **Comparator** | PBO | **Source** | Journal article(s) with results of the trial |
| **Outcome** | ⑦⑧ | **Results** | ⑦⑧ | **Weight** | 1 |
| **Domain** | **Signalling question** | | | **Response** | **Comments** |
| **Bias arising from the randomization process** | 1.1 Was the allocation sequence random? | | | PY |  |
|  | 1.2 Was the allocation sequence concealed until participants were enrolled and assigned to interventions? | | | NI |  |
|  | 1.3 Did baseline differences between intervention groups suggest a problem with the randomization process? | | | N |  |
|  | **Risk of bias judgement** | | | **Some concerns** |  |
| **Bias due to deviations from intended interventions** | 2.1.Were participants aware of their assigned intervention during the trial? | | | N | No studies of open-label treatment or active comparators were included. |
|  | 2.2.Were carers and people delivering the interventions aware of participants' assigned intervention during the trial? | | | NI |  |
|  | 2.3. If Y/PY/NI to 2.1 or 2.2: Were there deviations from the intended intervention that arose because of the experimental context? | | | PN |  |
|  | 2.4 If Y/PY to 2.3: Were these deviations likely to have affected the outcome? | | | NA |  |
|  | 2.5. If Y/PY/NI to 2.4: Were these deviations from intended intervention balanced between groups? | | | NA |  |
|  | 2.6 Was an appropriate analysis used to estimate the effect of assignment to intervention? | | | PY |  |
|  | 2.7 If N/PN/NI to 2.6: Was there potential for a substantial impact (on the result) of the failure to analyse participants in the group to which they were randomized? | | | NA |  |
|  | **Risk of bias judgement** | | | **Low** |  |
| **Bias due to missing outcome data** | 3.1 Were data for this outcome available for all, or nearly all, participants randomized? | | | PY |  |
|  | 3.2 If N/PN/NI to 3.1: Is there evidence that result was not biased by missing outcome data? | | | NA |  |
|  | 3.3 If N/PN to 3.2: Could missingness in the outcome depend on its true value? | | | NA |  |
|  | 3.4 If Y/PY/NI to 3.3: Is it likely that missingness in the outcome depended on its true value? | | | NA |  |
|  | **Risk of bias judgement** | | | **Low** |  |
| **Bias in measurement of the outcome** | 4.1 Was the method of measuring the outcome inappropriate? | | | PN |  |
|  | 4.2 Could measurement or ascertainment of the outcome have differed between intervention groups? | | | PN |  |
|  | 4.3 Were outcome assessors aware of the intervention received by study participants? | | | NI |  |
|  | 4.4 If Y/PY/NI to 4.3: Could assessment of the outcome have been influenced by knowledge of intervention received? | | | PN |  |
|  | 4.5 If Y/PY/NI to 4.4: Is it likely that assessment of the outcome was influenced by knowledge of intervention received? | | | NA |  |
|  | **Risk of bias judgement** | | | **Low** |  |
| **Bias in selection of the reported result** | 5.1 Were the data that produced this result analysed in accordance with a pre-specified analysis plan that was finalized before unblinded outcome data were available for analysis? | | | PY |  |
|  | 5.2 ... multiple eligible outcome measurements (e.g. scales, definitions, time points) within the outcome domain? | | | PN |  |
|  | 5.3 ... multiple eligible analyses of the data? | | | PN |  |
|  | **Risk of bias judgement** | | | **Low** |  |
| **Overall bias** | **Risk of bias judgement** | | | **Some concerns** |  |
| **Unique ID** | 20 | **Study ID** | NCT02540993 and NCT02545049 | **Assessor** | G and J |
| **Ref or Label** | 10.2215/CJN.00000000000000149 | **Aim** | assignment to intervention (the 'intention-to-treat' effect) |  |  |
| **Experimental** | Finerenone | **Comparator** | PBO | **Source** | Journal article(s) with results of the trial; Trial protocol; Non-commercial trial registry record (e.g. ClinicalTrials.gov record) |
| **Outcome** | ②④⑨⑩ | **Results** | ②④⑨⑩ | **Weight** | 1 |
| **Domain** | **Signalling question** | | | **Response** | **Comments** |
| **Bias arising from the randomization process** | 1.1 Was the allocation sequence random? | | | NI | The only information about the randomization method is that the study was randomized. All the patients and study personnel (with the exception of the independent data monitoring committee) had no role in the treatment assignments |
|  | 1.2 Was the allocation sequence concealed until participants were enrolled and assigned to interventions? | | | Y |  |
|  | 1.3 Did baseline differences between intervention groups suggest a problem with the randomization process? | | | N |  |
|  | **Risk of bias judgement** | | | **Low** |  |
| **Bias due to deviations from intended interventions** | 2.1.Were participants aware of their assigned intervention during the trial? | | | N | Masking: Quadruple (Participant, Care Provider, Investigator, Outcomes Assessor) |
|  | 2.2.Were carers and people delivering the interventions aware of participants' assigned intervention during the trial? | | | N |  |
|  | 2.3. If Y/PY/NI to 2.1 or 2.2: Were there deviations from the intended intervention that arose because of the experimental context? | | | NA |  |
|  | 2.4 If Y/PY to 2.3: Were these deviations likely to have affected the outcome? | | | NA |  |
|  | 2.5. If Y/PY/NI to 2.4: Were these deviations from intended intervention balanced between groups? | | | NA |  |
|  | 2.6 Was an appropriate analysis used to estimate the effect of assignment to intervention? | | | PY |  |
|  | 2.7 If N/PN/NI to 2.6: Was there potential for a substantial impact (on the result) of the failure to analyse participants in the group to which they were randomized? | | | NA |  |
|  | **Risk of bias judgement** | | | **Low** |  |
| **Bias due to missing outcome data** | 3.1 Were data for this outcome available for all, or nearly all, participants randomized? | | | PY |  |
|  | 3.2 If N/PN/NI to 3.1: Is there evidence that result was not biased by missing outcome data? | | | NA |  |
|  | 3.3 If N/PN to 3.2: Could missingness in the outcome depend on its true value? | | | NA |  |
|  | 3.4 If Y/PY/NI to 3.3: Is it likely that missingness in the outcome depended on its true value? | | | NA |  |
|  | **Risk of bias judgement** | | | **Low** |  |
| **Bias in measurement of the outcome** | 4.1 Was the method of measuring the outcome inappropriate? | | | PN |  |
|  | 4.2 Could measurement or ascertainment of the outcome have differed between intervention groups? | | | PN |  |
|  | 4.3 Were outcome assessors aware of the intervention received by study participants? | | | N | Masking: Quadruple (Participant, Care Provider, Investigator, Outcomes Assessor) |
|  | 4.4 If Y/PY/NI to 4.3: Could assessment of the outcome have been influenced by knowledge of intervention received? | | | NA |  |
|  | 4.5 If Y/PY/NI to 4.4: Is it likely that assessment of the outcome was influenced by knowledge of intervention received? | | | NA |  |
|  | **Risk of bias judgement** | | | **Low** |  |
| **Bias in selection of the reported result** | 5.1 Were the data that produced this result analysed in accordance with a pre-specified analysis plan that was finalized before unblinded outcome data were available for analysis? | | | PY |  |
|  | 5.2 ... multiple eligible outcome measurements (e.g. scales, definitions, time points) within the outcome domain? | | | PN |  |
|  | 5.3 ... multiple eligible analyses of the data? | | | PN |  |
|  | **Risk of bias judgement** | | | **Low** |  |
| **Overall bias** | **Risk of bias judgement** | | | **Low** |  |
| **Unique ID** | 21 | **Study ID** | NCT02065791 | **Assessor** | G and J |
| **Ref or Label** | 10.1161/CIRCULATIONAHA.119.042007 | **Aim** | assignment to intervention (the 'intention-to-treat' effect) |  |  |
| **Experimental** | Canagliflozin | **Comparator** | PBO | **Source** | Journal article(s) with results of the trial; Trial protocol; Non-commercial trial registry record (e.g. ClinicalTrials.gov record) |
| **Outcome** | ⑦⑧⑨⑩ | **Results** | ⑦⑧⑨⑩ | **Weight** | 1 |
| **Domain** | **Signalling question** | | | **Response** | **Comments** |
| **Bias arising from the randomization process** | 1.1 Was the allocation sequence random? | | | Y | Randomization is performed centrally through an interactive web response system using a computer-generated randomization schedule prepared by the study sponsor. |
|  | 1.2 Was the allocation sequence concealed until participants were enrolled and assigned to interventions? | | | Y |  |
|  | 1.3 Did baseline differences between intervention groups suggest a problem with the randomization process? | | | N |  |
|  | **Risk of bias judgement** | | | **Low** |  |
| **Bias due to deviations from intended interventions** | 2.1.Were participants aware of their assigned intervention during the trial? | | | N | Patients and all study staff will remain blinded to individual treatment allocation until the completion of the study. |
|  | 2.2.Were carers and people delivering the interventions aware of participants' assigned intervention during the trial? | | | N |  |
|  | 2.3. If Y/PY/NI to 2.1 or 2.2: Were there deviations from the intended intervention that arose because of the experimental context? | | | NA |  |
|  | 2.4 If Y/PY to 2.3: Were these deviations likely to have affected the outcome? | | | NA |  |
|  | 2.5. If Y/PY/NI to 2.4: Were these deviations from intended intervention balanced between groups? | | | NA |  |
|  | 2.6 Was an appropriate analysis used to estimate the effect of assignment to intervention? | | | Y |  |
|  | 2.7 If N/PN/NI to 2.6: Was there potential for a substantial impact (on the result) of the failure to analyse participants in the group to which they were randomized? | | | NA |  |
|  | **Risk of bias judgement** | | | **Low** |  |
| **Bias due to missing outcome data** | 3.1 Were data for this outcome available for all, or nearly all, participants randomized? | | | PY |  |
|  | 3.2 If N/PN/NI to 3.1: Is there evidence that result was not biased by missing outcome data? | | | NA |  |
|  | 3.3 If N/PN to 3.2: Could missingness in the outcome depend on its true value? | | | NA |  |
|  | 3.4 If Y/PY/NI to 3.3: Is it likely that missingness in the outcome depended on its true value? | | | NA |  |
|  | **Risk of bias judgement** | | | **Low** |  |
| **Bias in measurement of the outcome** | 4.1 Was the method of measuring the outcome inappropriate? | | | N |  |
|  | 4.2 Could measurement or ascertainment of the outcome have differed between intervention groups? | | | N | Patients and all study staff will remain blinded to individual treatment allocation until the completion of the study. |
|  | 4.3 Were outcome assessors aware of the intervention received by study participants? | | | PN |  |
|  | 4.4 If Y/PY/NI to 4.3: Could assessment of the outcome have been influenced by knowledge of intervention received? | | | NA |  |
|  | 4.5 If Y/PY/NI to 4.4: Is it likely that assessment of the outcome was influenced by knowledge of intervention received? | | | NA |  |
|  | **Risk of bias judgement** | | | **Low** |  |
| **Bias in selection of the reported result** | 5.1 Were the data that produced this result analysed in accordance with a pre-specified analysis plan that was finalized before unblinded outcome data were available for analysis? | | | PY |  |
|  | 5.2 ... multiple eligible outcome measurements (e.g. scales, definitions, time points) within the outcome domain? | | | PN |  |
|  | 5.3 ... multiple eligible analyses of the data? | | | PN |  |
|  | **Risk of bias judgement** | | | **Low** |  |
| **Overall bias** | **Risk of bias judgement** | | | **Low** |  |
| **Unique ID** | 22 | **Study ID** | NCT03436693 | **Assessor** | G and J |
| **Ref or Label** | 10.1111/jdi.13888 | **Aim** | assignment to intervention (the 'intention-to-treat' effect) |  |  |
| **Experimental** | Canagliflozin | **Comparator** | PBO | **Source** |  |
| **Outcome** | ①②④⑤⑦⑧⑩ | **Results** | ①②④⑤⑦⑧⑩ | **Weight** | 1 |
| **Domain** | **Signalling question** | | | **Response** | **Comments** |
| **Bias arising from the randomization process** | 1.1 Was the allocation sequence random? | | | NI | The only information about the randomization method is that the study was randomized |
|  | 1.2 Was the allocation sequence concealed until participants were enrolled and assigned to interventions? | | | NI |  |
|  | 1.3 Did baseline differences between intervention groups suggest a problem with the randomization process? | | | N |  |
|  | **Risk of bias judgement** | | | **Some concerns** |  |
| **Bias due to deviations from intended interventions** | 2.1.Were participants aware of their assigned intervention during the trial? | | | N | Masking:Quadruple (Participant, Care Provider, Investigator, Outcomes Assessor) |
|  | 2.2.Were carers and people delivering the interventions aware of participants' assigned intervention during the trial? | | | N |  |
|  | 2.3. If Y/PY/NI to 2.1 or 2.2: Were there deviations from the intended intervention that arose because of the experimental context? | | | NA |  |
|  | 2.4 If Y/PY to 2.3: Were these deviations likely to have affected the outcome? | | | NA |  |
|  | 2.5. If Y/PY/NI to 2.4: Were these deviations from intended intervention balanced between groups? | | | NA |  |
|  | 2.6 Was an appropriate analysis used to estimate the effect of assignment to intervention? | | | PY |  |
|  | 2.7 If N/PN/NI to 2.6: Was there potential for a substantial impact (on the result) of the failure to analyse participants in the group to which they were randomized? | | | NA |  |
|  | **Risk of bias judgement** | | | **Low** |  |
| **Bias due to missing outcome data** | 3.1 Were data for this outcome available for all, or nearly all, participants randomized? | | | PY |  |
|  | 3.2 If N/PN/NI to 3.1: Is there evidence that result was not biased by missing outcome data? | | | NA |  |
|  | 3.3 If N/PN to 3.2: Could missingness in the outcome depend on its true value? | | | NA |  |
|  | 3.4 If Y/PY/NI to 3.3: Is it likely that missingness in the outcome depended on its true value? | | | NA |  |
|  | **Risk of bias judgement** | | | **Low** |  |
| **Bias in measurement of the outcome** | 4.1 Was the method of measuring the outcome inappropriate? | | | N |  |
|  | 4.2 Could measurement or ascertainment of the outcome have differed between intervention groups? | | | PN | Patients and all study staff will remain blinded to individual treatment allocation until the completion of the study. |
|  | 4.3 Were outcome assessors aware of the intervention received by study participants? | | | N | Masking: Quadruple (Participant, Care Provider, Investigator, Outcomes Assessor) |
|  | 4.4 If Y/PY/NI to 4.3: Could assessment of the outcome have been influenced by knowledge of intervention received? | | | NA |  |
|  | 4.5 If Y/PY/NI to 4.4: Is it likely that assessment of the outcome was influenced by knowledge of intervention received? | | | NA |  |
|  | **Risk of bias judgement** | | | **Low** |  |
| **Bias in selection of the reported result** | 5.1 Were the data that produced this result analysed in accordance with a pre-specified analysis plan that was finalized before unblinded outcome data were available for analysis? | | | PY |  |
|  | 5.2 ... multiple eligible outcome measurements (e.g. scales, definitions, time points) within the outcome domain? | | | PN |  |
|  | 5.3 ... multiple eligible analyses of the data? | | | PN |  |
|  | **Risk of bias judgement** | | | **Low** |  |
| **Overall bias** | **Risk of bias judgement** | | | **Low** |  |
| **Unique ID** | 23 | **Study ID** | NCT02065791 | **Assessor** | G and J |
| **Ref or Label** | 10.2215/CJN.10140620 | **Aim** | assignment to intervention (the 'intention-to-treat' effect) |  |  |
| **Experimental** | Canagliflozin | **Comparator** | PBO | **Source** | Journal article(s) with results of the trial; Trial protocol; Non-commercial trial registry record (e.g. ClinicalTrials.gov record) |
| **Outcome** | ②⑨⑩ | **Results** | ②⑨⑩ | **Weight** | 1 |
| **Domain** | **Signalling question** | | | **Response** | **Comments** |
| **Bias arising from the randomization process** | 1.1 Was the allocation sequence random? | | | Y | Randomization is performed centrally through an interactive web response system using a computer-generated randomization schedule prepared by the study sponsor. |
|  | 1.2 Was the allocation sequence concealed until participants were enrolled and assigned to interventions? | | | Y |  |
|  | 1.3 Did baseline differences between intervention groups suggest a problem with the randomization process? | | | N |  |
|  | **Risk of bias judgement** | | | **Low** |  |
| **Bias due to deviations from intended interventions** | 2.1.Were participants aware of their assigned intervention during the trial? | | | N | Patients and all study staff will remain blinded to individual treatment allocation until the completion of the study. |
|  | 2.2.Were carers and people delivering the interventions aware of participants' assigned intervention during the trial? | | | N |  |
|  | 2.3. If Y/PY/NI to 2.1 or 2.2: Were there deviations from the intended intervention that arose because of the experimental context? | | | NA |  |
|  | 2.4 If Y/PY to 2.3: Were these deviations likely to have affected the outcome? | | | NA |  |
|  | 2.5. If Y/PY/NI to 2.4: Were these deviations from intended intervention balanced between groups? | | | NA |  |
|  | 2.6 Was an appropriate analysis used to estimate the effect of assignment to intervention? | | | Y |  |
|  | 2.7 If N/PN/NI to 2.6: Was there potential for a substantial impact (on the result) of the failure to analyse participants in the group to which they were randomized? | | | NA |  |
|  | **Risk of bias judgement** | | | **Low** |  |
| **Bias due to missing outcome data** | 3.1 Were data for this outcome available for all, or nearly all, participants randomized? | | | PY |  |
|  | 3.2 If N/PN/NI to 3.1: Is there evidence that result was not biased by missing outcome data? | | | NA |  |
|  | 3.3 If N/PN to 3.2: Could missingness in the outcome depend on its true value? | | | NA |  |
|  | 3.4 If Y/PY/NI to 3.3: Is it likely that missingness in the outcome depended on its true value? | | | NA |  |
|  | **Risk of bias judgement** | | | **Low** |  |
| **Bias in measurement of the outcome** | 4.1 Was the method of measuring the outcome inappropriate? | | | N | Patients and all study staff will remain blinded to individual treatment allocation until the completion of the study. |
|  | 4.2 Could measurement or ascertainment of the outcome have differed between intervention groups? | | | N |  |
|  | 4.3 Were outcome assessors aware of the intervention received by study participants? | | | PN |  |
|  | 4.4 If Y/PY/NI to 4.3: Could assessment of the outcome have been influenced by knowledge of intervention received? | | | NA |  |
|  | 4.5 If Y/PY/NI to 4.4: Is it likely that assessment of the outcome was influenced by knowledge of intervention received? | | | NA |  |
|  | **Risk of bias judgement** | | | **Low** |  |
| **Bias in selection of the reported result** | 5.1 Were the data that produced this result analysed in accordance with a pre-specified analysis plan that was finalized before unblinded outcome data were available for analysis? | | | PY |  |
|  | 5.2 ... multiple eligible outcome measurements (e.g. scales, definitions, time points) within the outcome domain? | | | PN |  |
|  | 5.3 ... multiple eligible analyses of the data? | | | PN |  |
|  | **Risk of bias judgement** | | | **Low** |  |
| **Overall bias** | **Risk of bias judgement** | | | **Low** |  |
| **Unique ID** | 24 | **Study ID** | NCT01986855 | **Assessor** | G and J |
| **Ref or Label** | 10.1007/s13300-017-0337-5 | **Aim** | assignment to intervention (the 'intention-to-treat' effect) |  |  |
| **Experimental** | Ertugliflozin | **Comparator** | PBO | **Source** | Journal article(s) with results of the trial; Trial protocol; Non-commercial trial registry record (e.g. ClinicalTrials.gov record) |
| **Outcome** | ①②⑦⑩ | **Results** | ①②⑦⑩ | **Weight** | 1 |
| **Domain** | **Signalling question** | | | **Response** | **Comments** |
| **Bias arising from the randomization process** | 1.1 Was the allocation sequence random? | | | Y | Randomization was implemented centrally using an interactive voice response system/integrated web response system. |
|  | 1.2 Was the allocation sequence concealed until participants were enrolled and assigned to interventions? | | | PY |  |
|  | 1.3 Did baseline differences between intervention groups suggest a problem with the randomization process? | | | N | The baseline demographics of the treatment groups were similar |
|  | **Risk of bias judgement** | | | **Low** |  |
| **Bias due to deviations from intended interventions** | 2.1.Were participants aware of their assigned intervention during the trial? | | | N | The sponsor was unblinded after the week 26 database lock to permit the writing of the phase A clinical study report. |
|  | 2.2.Were carers and people delivering the interventions aware of participants' assigned intervention during the trial? | | | NI |  |
|  | 2.3. If Y/PY/NI to 2.1 or 2.2: Were there deviations from the intended intervention that arose because of the experimental context? | | | PN |  |
|  | 2.4 If Y/PY to 2.3: Were these deviations likely to have affected the outcome? | | | NA |  |
|  | 2.5. If Y/PY/NI to 2.4: Were these deviations from intended intervention balanced between groups? | | | NA |  |
|  | 2.6 Was an appropriate analysis used to estimate the effect of assignment to intervention? | | | PY |  |
|  | 2.7 If N/PN/NI to 2.6: Was there potential for a substantial impact (on the result) of the failure to analyse participants in the group to which they were randomized? | | | NA |  |
|  | **Risk of bias judgement** | | | **Low** |  |
| **Bias due to missing outcome data** | 3.1 Were data for this outcome available for all, or nearly all, participants randomized? | | | PY |  |
|  | 3.2 If N/PN/NI to 3.1: Is there evidence that result was not biased by missing outcome data? | | | NA |  |
|  | 3.3 If N/PN to 3.2: Could missingness in the outcome depend on its true value? | | | NA |  |
|  | 3.4 If Y/PY/NI to 3.3: Is it likely that missingness in the outcome depended on its true value? | | | NA |  |
|  | **Risk of bias judgement** | | | **Low** |  |
| **Bias in measurement of the outcome** | 4.1 Was the method of measuring the outcome inappropriate? | | | PN |  |
|  | 4.2 Could measurement or ascertainment of the outcome have differed between intervention groups? | | | PN |  |
|  | 4.3 Were outcome assessors aware of the intervention received by study participants? | | | PY | The sponsor was unblinded after the week 26 database lock to permit the writing of the phase A clinical study report. |
|  | 4.4 If Y/PY/NI to 4.3: Could assessment of the outcome have been influenced by knowledge of intervention received? | | | PN |  |
|  | 4.5 If Y/PY/NI to 4.4: Is it likely that assessment of the outcome was influenced by knowledge of intervention received? | | | NA |  |
|  | **Risk of bias judgement** | | | **Low** |  |
| **Bias in selection of the reported result** | 5.1 Were the data that produced this result analysed in accordance with a pre-specified analysis plan that was finalized before unblinded outcome data were available for analysis? | | | PY |  |
|  | 5.2 ... multiple eligible outcome measurements (e.g. scales, definitions, time points) within the outcome domain? | | | PN |  |
|  | 5.3 ... multiple eligible analyses of the data? | | | PN |  |
|  | **Risk of bias judgement** | | | **Low** |  |
| **Overall bias** | **Risk of bias judgement** | | | **Low** |  |
| **Unique ID** | 25 | **Study ID** | NCT01986881 | **Assessor** | G and J |
| **Ref or Label** | 10.1136/bmjdrc-2021-002484 | **Aim** | assignment to intervention (the 'intention-to-treat' effect) |  |  |
| **Experimental** | Ertugliflozin | **Comparator** | PBO | **Source** | Journal article(s) with results of the trial; Trial protocol; Non-commercial trial registry record (e.g. ClinicalTrials.gov record) |
| **Outcome** | ①②④⑥⑦⑧⑨⑩ | **Results** | ①②④⑥⑦⑧⑨⑩ | **Weight** | 1 |
| **Domain** | **Signalling question** | | | **Response** | **Comments** |
| **Bias arising from the randomization process** | 1.1 Was the allocation sequence random? | | | Y | Masking: Double (Participant, Investigator) |
|  | 1.2 Was the allocation sequence concealed until participants were enrolled and assigned to interventions? | | | PY |  |
|  | 1.3 Did baseline differences between intervention groups suggest a problem with the randomization process? | | | N | Baseline demographic and clinical characteristics were well balanced across treatment groups in the subgroup of patients with CKD stage 3 |
|  | **Risk of bias judgement** | | | **Low** |  |
| **Bias due to deviations from intended interventions** | 2.1. Were participants aware of their assigned intervention during the trial? | | | N | Masking: Double (Participant, Investigator) |
|  | 2.2. Were carers and people delivering the interventions aware of participants' assigned intervention during the trial? | | | NI |  |
|  | 2.3. If Y/PY/NI to 2.1 or 2.2: Were there deviations from the intended intervention that arose because of the experimental context? | | | PN |  |
|  | 2.4 If Y/PY to 2.3: Were these deviations likely to have affected the outcome? | | | NA |  |
|  | 2.5. If Y/PY/NI to 2.4: Were these deviations from intended intervention balanced between groups? | | | NA |  |
|  | 2.6 Was an appropriate analysis used to estimate the effect of assignment to intervention? | | | PY |  |
|  | 2.7 If N/PN/NI to 2.6: Was there potential for a substantial impact (on the result) of the failure to analyse participants in the group to which they were randomized? | | | NA |  |
|  | **Risk of bias judgement** | | | **Low** |  |
| **Bias due to missing outcome data** | 3.1 Were data for this outcome available for all, or nearly all, participants randomized? | | | PY |  |
|  | 3.2 If N/PN/NI to 3.1: Is there evidence that result was not biased by missing outcome data? | | | NA |  |
|  | 3.3 If N/PN to 3.2: Could missingness in the outcome depend on its true value? | | | NA |  |
|  | 3.4 If Y/PY/NI to 3.3: Is it likely that missingness in the outcome depended on its true value? | | | NA |  |
|  | **Risk of bias judgement** | | | **Low** |  |
| **Bias in measurement of the outcome** | 4.1 Was the method of measuring the outcome inappropriate? | | | PN |  |
|  | 4.2 Could measurement or ascertainment of the outcome have differed between intervention groups? | | | PN |  |
|  | 4.3 Were outcome assessors aware of the intervention received by study participants? | | | PY | The sponsor was unblinded after the week 26 database lock to permit the writing of the phase A clinical study report. |
|  | 4.4 If Y/PY/NI to 4.3: Could assessment of the outcome have been influenced by knowledge of intervention received? | | | PN |  |
|  | 4.5 If Y/PY/NI to 4.4: Is it likely that assessment of the outcome was influenced by knowledge of intervention received? | | | NA |  |
|  | **Risk of bias judgement** | | | **Low** |  |
| **Bias in selection of the reported result** | 5.1 Were the data that produced this result analysed in accordance with a pre-specified analysis plan that was finalized before unblinded outcome data were available for analysis? | | | PY |  |
|  | 5.2 ... multiple eligible outcome measurements (e.g. scales, definitions, time points) within the outcome domain? | | | PN |  |
|  | 5.3 ... multiple eligible analyses of the data? | | | PN |  |
|  | **Risk of bias judgement** | | | **Low** |  |
| **Overall bias** | **Risk of bias judgement** | | | **Low** |  |
| **Unique ID** | 26 | **Study ID** | NCT03242018 | **Assessor** | G and J |
| **Ref or Label** | 10.1111/dom.14513 | **Aim** | assignment to intervention (the 'intention-to-treat' effect) |  |  |
| **Experimental** | Sotagliflozin | **Comparator** | PBO | **Source** | Journal article(s) with results of the trial; Trial protocol; Non-commercial trial registry record (e.g. ClinicalTrials.gov record) |
| **Outcome** | ①②④⑥⑦⑧⑩ | **Results** | ①②④⑥⑦⑧⑩ | **Weight** | 1 |
| **Domain** | **Signalling question** | | | **Response** | **Comments** |
| **Bias arising from the randomization process** | 1.1 Was the allocation sequence random? | | | NI | The only information about the randomization method is that the study was randomized Masking: Quadruple (Participant,Care Provider, Investigator, Outcomes Assessor) |
|  | 1.2 Was the allocation sequence concealed until participants were enrolled and assigned to interventions? | | | PY |  |
|  | 1.3 Did baseline differences between intervention groups suggest a problem with the randomization process? | | | N | Baseline characteristics were balanced between treatment groups |
|  | **Risk of bias judgement** | | | **Low** |  |
| **Bias due to deviations from intended interventions** | 2.1. Were participants aware of their assigned intervention during the trial? | | | N | Masking: Quadruple (Participant, Care Provider, Investigator, Outcomes Assessor) |
|  | 2.2. Were carers and people delivering the interventions aware of participants' assigned intervention during the trial? | | | N |  |
|  | 2.3. If Y/PY/NI to 2.1 or 2.2: Were there deviations from the intended intervention that arose because of the experimental context? | | | NA |  |
|  | 2.4 If Y/PY to 2.3: Were these deviations likely to have affected the outcome? | | | NA |  |
|  | 2.5. If Y/PY/NI to 2.4: Were these deviations from intended intervention balanced between groups? | | | NA |  |
|  | 2.6 Was an appropriate analysis used to estimate the effect of assignment to intervention? | | | Y | intention-to-treat (ITT) |
|  | 2.7 If N/PN/NI to 2.6: Was there potential for a substantial impact (on the result) of the failure to analyse participants in the group to which they were randomized? | | | NA |  |
|  | **Risk of bias judgement** | | | **Low** |  |
| **Bias due to missing outcome data** | 3.1 Were data for this outcome available for all, or nearly all, participants randomized? | | | PY |  |
|  | 3.2 If N/PN/NI to 3.1: Is there evidence that result was not biased by missing outcome data? | | | NA |  |
|  | 3.3 If N/PN to 3.2: Could missingness in the outcome depend on its true value? | | | NA |  |
|  | 3.4 If Y/PY/NI to 3.3: Is it likely that missingness in the outcome depended on its true value? | | | NA |  |
|  | **Risk of bias judgement** | | | **Low** |  |
| **Bias in measurement of the outcome** | 4.1 Was the method of measuring the outcome inappropriate? | | | PN |  |
|  | 4.2 Could measurement or ascertainment of the outcome have differed between intervention groups? | | | PN |  |
|  | 4.3 Were outcome assessors aware of the intervention received by study participants? | | | N | Masking: Quadruple (Participant, Care Provider, Investigator, Outcomes Assessor) |
|  | 4.4 If Y/PY/NI to 4.3: Could assessment of the outcome have been influenced by knowledge of intervention received? | | | NA |  |
|  | 4.5 If Y/PY/NI to 4.4: Is it likely that assessment of the outcome was influenced by knowledge of intervention received? | | | NA |  |
|  | **Risk of bias judgement** | | | **Low** |  |
| **Bias in selection of the reported result** | 5.1 Were the data that produced this result analysed in accordance with a pre-specified analysis plan that was finalized before unblinded outcome data were available for analysis? | | | PY |  |
|  | 5.2 ... multiple eligible outcome measurements (e.g. scales, definitions, time points) within the outcome domain? | | | PN |  |
|  | 5.3 ... multiple eligible analyses of the data? | | | PN |  |
|  | **Risk of bias judgement** | | | **Low** |  |
| **Overall bias** | **Risk of bias judgement** | | | **Low** |  |
| **Unique ID** | 27 | **Study ID** | NCT03315143 | **Assessor** | G and J |
| **Ref or Label** | 10.1056/NEJMoa2030186 | **Aim** | assignment to intervention (the 'intention-to-treat' effect) |  |  |
| **Experimental** | Sotagliflozin | **Comparator** | PBO | **Source** |  |
| **Outcome** | ⑦⑧⑩ | **Results** | ⑦⑧⑩ | **Weight** | 1 |
| **Domain** | **Signalling question** | | | **Response** | **Comments** |
| **Bias arising from the randomization process** | 1.1 Was the allocation sequence random? | | | PY | An independent data and safety monitoring board oversaw the trial. |
|  | 1.2 Was the allocation sequence concealed until participants were enrolled and assigned to interventions? | | | PY |  |
|  | 1.3 Did baseline differences between intervention groups suggest a problem with the randomization process? | | | N |  |
|  | **Risk of bias judgement** | | | **Low** |  |
| **Bias due to deviations from intended interventions** | 2.1.Were participants aware of their assigned intervention during the trial? | | | N | Masking: Quadruple (Participant, Care Provider, Investigator, Outcomes Assessor) |
|  | 2.2.Were carers and people delivering the interventions aware of participants' assigned intervention during the trial? | | | N |  |
|  | 2.3. If Y/PY/NI to 2.1 or 2.2: Were there deviations from the intended intervention that arose because of the experimental context? | | | NA |  |
|  | 2.4 If Y/PY to 2.3: Were these deviations likely to have affected the outcome? | | | NA |  |
|  | 2.5. If Y/PY/NI to 2.4: Were these deviations from intended intervention balanced between groups? | | | NA |  |
|  | 2.6 Was an appropriate analysis used to estimate the effect of assignment to intervention? | | | Y | Patients in the ITT population |
|  | 2.7 If N/PN/NI to 2.6: Was there potential for a substantial impact (on the result) of the failure to analyse participants in the group to which they were randomized? | | | NA |  |
|  | **Risk of bias judgement** | | | **Low** |  |
| **Bias due to missing outcome data** | 3.1 Were data for this outcome available for all, or nearly all, participants randomized? | | | PY |  |
|  | 3.2 If N/PN/NI to 3.1: Is there evidence that result was not biased by missing outcome data? | | | NA |  |
|  | 3.3 If N/PN to 3.2: Could missingness in the outcome depend on its true value? | | | NA |  |
|  | 3.4 If Y/PY/NI to 3.3: Is it likely that missingness in the outcome depended on its true value? | | | NA |  |
|  | **Risk of bias judgement** | | | **Low** |  |
| **Bias in measurement of the outcome** | 4.1 Was the method of measuring the outcome inappropriate? | | | PN |  |
|  | 4.2 Could measurement or ascertainment of the outcome have differed between intervention groups? | | | PN |  |
|  | 4.3 Were outcome assessors aware of the intervention received by study participants? | | | N | Masking: Quadruple (Participant, Care Provider, Investigator, Outcomes Assessor) |
|  | 4.4 If Y/PY/NI to 4.3: Could assessment of the outcome have been influenced by knowledge of intervention received? | | | NA |  |
|  | 4.5 If Y/PY/NI to 4.4: Is it likely that assessment of the outcome was influenced by knowledge of intervention received? | | | NA |  |
|  | **Risk of bias judgement** | | | **Low** |  |
| **Bias in selection of the reported result** | 5.1 Were the data that produced this result analysed in accordance with a pre-specified analysis plan that was finalized before unblinded outcome data were available for analysis? | | | PY |  |
|  | 5.2 ... multiple eligible outcome measurements (e.g. scales, definitions, time points) within the outcome domain? | | | PN |  |
|  | 5.3 ... multiple eligible analyses of the data? | | | PN |  |
|  | **Risk of bias judgement** | | | **Low** |  |
| **Overall bias** | **Risk of bias judgement** | | | **Low** |  |
| **Unique ID** | 28 | **Study ID** | NCT02545049 | **Assessor** | G and J |
| **Ref or Label** | 10.1056/NEJMoa2110956 | **Aim** | assignment to intervention (the 'intention-to-treat' effect) |  |  |
| **Experimental** | Finerenone | **Comparator** | PBO | **Source** |  |
| **Outcome** | ①④⑥⑦⑧⑨⑩ | **Results** | ①④⑥⑦⑧⑨⑩ | **Weight** | 1 |
| **Domain** | **Signalling question** | | | **Response** | **Comments** |
| **Bias arising from the randomization process** | 1.1 Was the allocation sequence random? | | | NI | The only information about the randomization method is that the study was randomized |
|  | 1.2 Was the allocation sequence concealed until participants were enrolled and assigned to interventions? | | | PY |  |
|  | 1.3 Did baseline differences between intervention groups suggest a problem with the randomization process? | | | N |  |
|  | **Risk of bias judgement** | | | **Low** |  |
| **Bias due to deviations from intended interventions** | 2.1.Were participants aware of their assigned intervention during the trial? | | | N | Masking: Quadruple (Participant, Care Provider, Investigator, Outcomes Assessor) |
|  | 2.2.Were carers and people delivering the interventions aware of participants' assigned intervention during the trial? | | | N |  |
|  | 2.3. If Y/PY/NI to 2.1 or 2.2: Were there deviations from the intended intervention that arose because of the experimental context? | | | NA |  |
|  | 2.4 If Y/PY to 2.3: Were these deviations likely to have affected the outcome? | | | NA |  |
|  | 2.5. If Y/PY/NI to 2.4: Were these deviations from intended intervention balanced between groups? | | | NA |  |
|  | 2.6 Was an appropriate analysis used to estimate the effect of assignment to intervention? | | | PY |  |
|  | 2.7 If N/PN/NI to 2.6: Was there potential for a substantial impact (on the result) of the failure to analyse participants in the group to which they were randomized? | | | NA |  |
|  | **Risk of bias judgement** | | | **Low** |  |
| **Bias due to missing outcome data** | 3.1 Were data for this outcome available for all, or nearly all, participants randomized? | | | PY |  |
|  | 3.2 If N/PN/NI to 3.1: Is there evidence that result was not biased by missing outcome data? | | | NA |  |
|  | 3.3 If N/PN to 3.2: Could missingness in the outcome depend on its true value? | | | NA |  |
|  | 3.4 If Y/PY/NI to 3.3: Is it likely that missingness in the outcome depended on its true value? | | | NA |  |
|  | **Risk of bias judgement** | | | **Low** |  |
| **Bias in measurement of the outcome** | 4.1 Was the method of measuring the outcome inappropriate? | | | PN |  |
|  | 4.2 Could measurement or ascertainment of the outcome have differed between intervention groups? | | | PN |  |
|  | 4.3 Were outcome assessors aware of the intervention received by study participants? | | | N | Masking: Quadruple (Participant, Care Provider, Investigator, Outcomes Assessor) |
|  | 4.4 If Y/PY/NI to 4.3: Could assessment of the outcome have been influenced by knowledge of intervention received? | | | NA |  |
|  | 4.5 If Y/PY/NI to 4.4: Is it likely that assessment of the outcome was influenced by knowledge of intervention received? | | | NA |  |
|  | **Risk of bias judgement** | | | **Low** |  |
| **Bias in selection of the reported result** | 5.1 Were the data that produced this result analysed in accordance with a pre-specified analysis plan that was finalized before unblinded outcome data were available for analysis? | | | PY |  |
|  | 5.2 ... multiple eligible outcome measurements (e.g. scales, definitions, time points) within the outcome domain? | | | PN |  |
|  | 5.3 ... multiple eligible analyses of the data? | | | PN |  |
|  | **Risk of bias judgement** | | | **Low** |  |
| **Overall bias** | **Risk of bias judgement** | | | **Low** |  |
| **Unique ID** | 29 | **Study ID** | NCT02540993 | **Assessor** | G and J |
| **Ref or Label** | 10.1056/NEJMoa2025845 | **Aim** | assignment to intervention (the 'intention-to-treat' effect) |  |  |
| **Experimental** | Finerenone | **Comparator** | PBO | **Source** | Journal article(s) with results of the trial; Trial protocol; Non-commercial trial registry record (e.g. ClinicalTrials.gov record) |
| **Outcome** | ①②④⑥⑦⑧⑨⑩ | **Results** | ①②④⑥⑦⑧⑨⑩ | **Weight** | 1 |
| **Domain** | **Signalling question** | | | **Response** | **Comments** |
| **Bias arising from the randomization process** | 1.1 Was the allocation sequence random? | | | NI | The only information about the randomization method is that the study was randomized |
|  | 1.2 Was the allocation sequence concealed until participants were enrolled and assigned to interventions? | | | PY |  |
|  | 1.3 Did baseline differences between intervention groups suggest a problem with the randomization process? | | | N |  |
|  | **Risk of bias judgement** | | | **Low** |  |
| **Bias due to deviations from intended interventions** | 2.1.Were participants aware of their assigned intervention during the trial? | | | N | Masking: Quadruple (Participant, Care Provider, Investigator, Outcomes Assessor) |
|  | 2.2.Were carers and people delivering the interventions aware of participants' assigned intervention during the trial? | | | N |  |
|  | 2.3. If Y/PY/NI to 2.1 or 2.2: Were there deviations from the intended intervention that arose because of the experimental context? | | | NA |  |
|  | 2.4 If Y/PY to 2.3: Were these deviations likely to have affected the outcome? | | | NA |  |
|  | 2.5. If Y/PY/NI to 2.4: Were these deviations from intended intervention balanced between groups? | | | NA |  |
|  | 2.6 Was an appropriate analysis used to estimate the effect of assignment to intervention? | | | PY |  |
|  | 2.7 If N/PN/NI to 2.6: Was there potential for a substantial impact (on the result) of the failure to analyse participants in the group to which they were randomized? | | | NA |  |
|  | **Risk of bias judgement** | | | **Low** |  |
| **Bias due to missing outcome data** | 3.1 Were data for this outcome available for all, or nearly all, participants randomized? | | | PY |  |
|  | 3.2 If N/PN/NI to 3.1: Is there evidence that result was not biased by missing outcome data? | | | NA |  |
|  | 3.3 If N/PN to 3.2: Could missingness in the outcome depend on its true value? | | | NA |  |
|  | 3.4 If Y/PY/NI to 3.3: Is it likely that missingness in the outcome depended on its true value? | | | NA |  |
|  | **Risk of bias judgement** | | | **Low** |  |
| **Bias in measurement of the outcome** | 4.1 Was the method of measuring the outcome inappropriate? | | | PN |  |
|  | 4.2 Could measurement or ascertainment of the outcome have differed between intervention groups? | | | PN |  |
|  | 4.3 Were outcome assessors aware of the intervention received by study participants? | | | N | Masking: Quadruple (Participant, Care Provider, Investigator, Outcomes Assessor) |
|  | 4.4 If Y/PY/NI to 4.3: Could assessment of the outcome have been influenced by knowledge of intervention received? | | | NA |  |
|  | 4.5 If Y/PY/NI to 4.4: Is it likely that assessment of the outcome was influenced by knowledge of intervention received? | | | NA |  |
|  | **Risk of bias judgement** | | | **Low** |  |
| **Bias in selection of the reported result** | 5.1 Were the data that produced this result analysed in accordance with a pre-specified analysis plan that was finalized before unblinded outcome data were available for analysis? | | | PY |  |
|  | 5.2 ... multiple eligible outcome measurements (e.g. scales, definitions, time points) within the outcome domain? | | | PN |  |
|  | 5.3 ... multiple eligible analyses of the data? | | | PN |  |
|  | **Risk of bias judgement** | | | **Low** |  |
| **Overall bias** | **Risk of bias judgement** | | | **Low** |  |
| **Unique ID** | 30 | **Study ID** | NCT02540993 | **Assessor** | G and J |
| **Ref or Label** | 10.1161/CIRCULATIONAHA.120.051898 | **Aim** | assignment to intervention (the 'intention-to-treat' effect) |  |  |
| **Experimental** | Finerenone | **Comparator** | PBO | **Source** | Journal article(s) with results of the trial; Trial protocol; Non-commercial trial registry record (e.g. ClinicalTrials.gov record) |
| **Outcome** | ②④⑦⑧⑨⑩ | **Results** | ②④⑦⑧⑨⑩ | **Weight** | 1 |
| **Domain** | **Signalling question** | | | **Response** | **Comments** |
| **Bias arising from the randomization process** | 1.1 Was the allocation sequence random? | | | NI | The only information about the randomization method is that the study was randomized |
|  | 1.2 Was the allocation sequence concealed until participants were enrolled and assigned to interventions? | | | PY |  |
|  | 1.3 Did baseline differences between intervention groups suggest a problem with the randomization process? | | | N |  |
|  | **Risk of bias judgement** | | | **Low** |  |
| **Bias due to deviations from intended interventions** | 2.1.Were participants aware of their assigned intervention during the trial? | | | N | Masking: Quadruple (Participant, Care Provider, Investigator, Outcomes Assessor) |
|  | 2.2.Were carers and people delivering the interventions aware of participants' assigned intervention during the trial? | | | N |  |
|  | 2.3. If Y/PY/NI to 2.1 or 2.2: Were there deviations from the intended intervention that arose because of the experimental context? | | | NA |  |
|  | 2.4 If Y/PY to 2.3: Were these deviations likely to have affected the outcome? | | | NA |  |
|  | 2.5. If Y/PY/NI to 2.4: Were these deviations from intended intervention balanced between groups? | | | NA |  |
|  | 2.6 Was an appropriate analysis used to estimate the effect of assignment to intervention? | | | PY |  |
|  | 2.7 If N/PN/NI to 2.6: Was there potential for a substantial impact (on the result) of the failure to analyse participants in the group to which they were randomized? | | | NA |  |
|  | **Risk of bias judgement** | | | **Low** |  |
| **Bias due to missing outcome data** | 3.1 Were data for this outcome available for all, or nearly all, participants randomized? | | | PY |  |
|  | 3.2 If N/PN/NI to 3.1: Is there evidence that result was not biased by missing outcome data? | | | NA |  |
|  | 3.3 If N/PN to 3.2: Could missingness in the outcome depend on its true value? | | | NA |  |
|  | 3.4 If Y/PY/NI to 3.3: Is it likely that missingness in the outcome depended on its true value? | | | NA |  |
|  | **Risk of bias judgement** | | | **Low** |  |
| **Bias in measurement of the outcome** | 4.1 Was the method of measuring the outcome inappropriate? | | | PN |  |
|  | 4.2 Could measurement or ascertainment of the outcome have differed between intervention groups? | | | PN |  |
|  | 4.3 Were outcome assessors aware of the intervention received by study participants? | | | N | Masking: Quadruple (Participant, Care Provider, Investigator, Outcomes Assessor) |
|  | 4.4 If Y/PY/NI to 4.3: Could assessment of the outcome have been influenced by knowledge of intervention received? | | | NA |  |
|  | 4.5 If Y/PY/NI to 4.4: Is it likely that assessment of the outcome was influenced by knowledge of intervention received? | | | NA |  |
|  | **Risk of bias judgement** | | | **Low** |  |
| **Bias in selection of the reported result** | 5.1 Were the data that produced this result analysed in accordance with a pre-specified analysis plan that was finalized before unblinded outcome data were available for analysis? | | | PY |  |
|  | 5.2 ... multiple eligible outcome measurements (e.g. scales, definitions, time points) within the outcome domain? | | | PN |  |
|  | 5.3 ... multiple eligible analyses of the data? | | | PN |  |
|  | **Risk of bias judgement** | | | **Low** |  |
| **Overall bias** | **Risk of bias judgement** | | | **Low** |  |
| **Unique ID** | 31 | **Study ID** | NCT01874431 | **Assessor** | G and J |
| **Ref or Label** | 10.1097/HJH.0000000000003330 | **Aim** | assignment to intervention (the 'intention-to-treat' effect) |  |  |
| **Experimental** | Finerenone | **Comparator** | PBO | **Source** | Journal article(s) with results of the trial; Trial protocol; Non-commercial trial registry record (e.g. ClinicalTrials.gov record) |
| **Outcome** | ④⑩ | **Results** | ④⑩ | **Weight** | 1 |
| **Domain** | **Signalling question** | | | **Response** | **Comments** |
| **Bias arising from the randomization process** | 1.1 Was the allocation sequence random? | | | Y | Randomization was done centrally by an interactive voice/web response system using computer-generated randomization lists, and participants, investigators, and the sponsor’s clinical team were blinded to treatment allocation. |
|  | 1.2 Was the allocation sequence concealed until participants were enrolled and assigned to interventions? | | | Y |  |
|  | 1.3 Did baseline differences between intervention groups suggest a problem with the randomization process? | | | N |  |
|  | **Risk of bias judgement** | | | **Low** |  |
| **Bias due to deviations from intended interventions** | 2.1.Were participants aware of their assigned intervention during the trial? | | | N | Randomization was done centrally by an interactive voice/web response system using computer-generated randomization lists, and participants, investigators, and the sponsor’s clinical team were blinded to treatment allocation. |
|  | 2.2.Were carers and people delivering the interventions aware of participants' assigned intervention during the trial? | | | N |  |
|  | 2.3. If Y/PY/NI to 2.1 or 2.2: Were there deviations from the intended intervention that arose because of the experimental context? | | | NA |  |
|  | 2.4 If Y/PY to 2.3: Were these deviations likely to have affected the outcome? | | | NA |  |
|  | 2.5. If Y/PY/NI to 2.4: Were these deviations from intended intervention balanced between groups? | | | NA |  |
|  | 2.6 Was an appropriate analysis used to estimate the effect of assignment to intervention? | | | PY |  |
|  | 2.7 If N/PN/NI to 2.6: Was there potential for a substantial impact (on the result) of the failure to analyse participants in the group to which they were randomized? | | | NA |  |
|  | **Risk of bias judgement** | | | **Low** |  |
| **Bias due to missing outcome data** | 3.1 Were data for this outcome available for all, or nearly all, participants randomized? | | | PY |  |
|  | 3.2 If N/PN/NI to 3.1: Is there evidence that result was not biased by missing outcome data? | | | NA |  |
|  | 3.3 If N/PN to 3.2: Could missingness in the outcome depend on its true value? | | | NA |  |
|  | 3.4 If Y/PY/NI to 3.3: Is it likely that missingness in the outcome depended on its true value? | | | NA |  |
|  | **Risk of bias judgement** | | | **Low** |  |
| **Bias in measurement of the outcome** | 4.1 Was the method of measuring the outcome inappropriate? | | | N |  |
|  | 4.2 Could measurement or ascertainment of the outcome have differed between intervention groups? | | | PN |  |
|  | 4.3 Were outcome assessors aware of the intervention received by study participants? | | | N | Randomization was done centrally by an interactive voice/web response system using computer-generated randomization lists, and participants, investigators, and the sponsor’s clinical team were blinded to treatment allocation. |
|  | 4.4 If Y/PY/NI to 4.3: Could assessment of the outcome have been influenced by knowledge of intervention received? | | | NA |  |
|  | 4.5 If Y/PY/NI to 4.4: Is it likely that assessment of the outcome was influenced by knowledge of intervention received? | | | NA |  |
|  | **Risk of bias judgement** | | | **Low** |  |
| **Bias in selection of the reported result** | 5.1 Were the data that produced this result analysed in accordance with a pre-specified analysis plan that was finalized before unblinded outcome data were available for analysis? | | | PY |  |
|  | 5.2 ... multiple eligible outcome measurements (e.g. scales, definitions, time points) within the outcome domain? | | | PN |  |
|  | 5.3 ... multiple eligible analyses of the data? | | | PN |  |
|  | **Risk of bias judgement** | | | **Low** |  |
| **Overall bias** | **Risk of bias judgement** | | | **Low** |  |
| **Unique ID** | 32 | **Study ID** | NCT01874431 | **Assessor** | G and J |
| **Ref or Label** | 10.1001/jama.2015.10081 | **Aim** | assignment to intervention (the 'intention-to-treat' effect) |  |  |
| **Experimental** | Finerenone | **Comparator** | PBO | **Source** | Journal article(s) with results of the trial; Trial protocol; Non-commercial trial registry record (e.g. ClinicalTrials.gov record) |
| **Outcome** | ②⑩ | **Results** | ②⑩ | **Weight** | 1 |
| **Domain** | **Signalling question** | | | **Response** | **Comments** |
| **Bias arising from the randomization process** | 1.1 Was the allocation sequence random? | | | Y | Randomization was done centrally by an interactive voice/web response system using computer-generated randomization lists, and participants, investigators, and the sponsor’s clinical team were blinded to treatment allocation. |
|  | 1.2 Was the allocation sequence concealed until participants were enrolled and assigned to interventions? | | | Y |  |
|  | 1.3 Did baseline differences between intervention groups suggest a problem with the randomization process? | | | N |  |
|  | **Risk of bias judgement** | | | **Low** |  |
| **Bias due to deviations from intended interventions** | 2.1.Were participants aware of their assigned intervention during the trial? | | | N | Randomization was done centrally by an interactive voice/web response system using computer-generated randomization lists, and participants, investigators, and the sponsor’s clinical team were blinded to treatment allocation. |
|  | 2.2.Were carers and people delivering the interventions aware of participants' assigned intervention during the trial? | | | N |  |
|  | 2.3. If Y/PY/NI to 2.1 or 2.2: Were there deviations from the intended intervention that arose because of the experimental context? | | | NA |  |
|  | 2.4 If Y/PY to 2.3: Were these deviations likely to have affected the outcome? | | | NA |  |
|  | 2.5. If Y/PY/NI to 2.4: Were these deviations from intended intervention balanced between groups? | | | NA |  |
|  | 2.6 Was an appropriate analysis used to estimate the effect of assignment to intervention? | | | PY |  |
|  | 2.7 If N/PN/NI to 2.6: Was there potential for a substantial impact (on the result) of the failure to analyse participants in the group to which they were randomized? | | | NA |  |
|  | **Risk of bias judgement** | | | **Low** |  |
| **Bias due to missing outcome data** | 3.1 Were data for this outcome available for all, or nearly all, participants randomized? | | | PY |  |
|  | 3.2 If N/PN/NI to 3.1: Is there evidence that result was not biased by missing outcome data? | | | NA |  |
|  | 3.3 If N/PN to 3.2: Could missingness in the outcome depend on its true value? | | | NA |  |
|  | 3.4 If Y/PY/NI to 3.3: Is it likely that missingness in the outcome depended on its true value? | | | NA |  |
|  | **Risk of bias judgement** | | | **Low** |  |
| **Bias in measurement of the outcome** | 4.1 Was the method of measuring the outcome inappropriate? | | | N |  |
|  | 4.2 Could measurement or ascertainment of the outcome have differed between intervention groups? | | | PN |  |
|  | 4.3 Were outcome assessors aware of the intervention received by study participants? | | | N | Randomization was done centrally by an interactive voice/web response system using computer-generated randomization lists, and participants, investigators, and the sponsor’s clinical team were blinded to treatment allocation. |
|  | 4.4 If Y/PY/NI to 4.3: Could assessment of the outcome have been influenced by knowledge of intervention received? | | | NA |  |
|  | 4.5 If Y/PY/NI to 4.4: Is it likely that assessment of the outcome was influenced by knowledge of intervention received? | | | NA |  |
|  | **Risk of bias judgement** | | | **Low** |  |
| **Bias in selection of the reported result** | 5.1 Were the data that produced this result analysed in accordance with a pre-specified analysis plan that was finalized before unblinded outcome data were available for analysis? | | | PY |  |
|  | 5.2 ... multiple eligible outcome measurements (e.g. scales, definitions, time points) within the outcome domain? | | | PN |  |
|  | 5.3 ... multiple eligible analyses of the data? | | | PN |  |
|  | **Risk of bias judgement** | | | **Low** |  |
| **Overall bias** | **Risk of bias judgement** | | | **Low** |  |
| **Unique ID** | 33 | **Study ID** | NCT01131676 | **Assessor** | G and J |
| **Ref or Label** | 10.1161/CIRCULATIONAHA.117.028268 | **Aim** | assignment to intervention (the 'intention-to-treat' effect) |  |  |
| **Experimental** | Empagliflozin | **Comparator** | PBO | **Source** | Journal article(s) with results of the trial; Trial protocol; Non-commercial trial registry record (e.g. ClinicalTrials.gov record) |
| **Outcome** | ①③④⑥ | **Results** | ①③④⑥ | **Weight** | 1 |
| **Domain** | **Signalling question** | | | **Response** | **Comments** |
| **Bias arising from the randomization process** | 1.1 Was the allocation sequence random? | | | NI | The only information about the randomization method is that the study was randomized |
|  | 1.2 Was the allocation sequence concealed until participants were enrolled and assigned to interventions? | | | PY |  |
|  | 1.3 Did baseline differences between intervention groups suggest a problem with the randomization process? | | | N |  |
|  | **Risk of bias judgement** | | | **Low** |  |
| **Bias due to deviations from intended interventions** | 2.1.Were participants aware of their assigned intervention during the trial? | | | N | double-blind |
|  | 2.2.Were carers and people delivering the interventions aware of participants' assigned intervention during the trial? | | | NI |  |
|  | 2.3. If Y/PY/NI to 2.1 or 2.2: Were there deviations from the intended intervention that arose because of the experimental context? | | | PN |  |
|  | 2.4 If Y/PY to 2.3: Were these deviations likely to have affected the outcome? | | | NA |  |
|  | 2.5. If Y/PY/NI to 2.4: Were these deviations from intended intervention balanced between groups? | | | NA |  |
|  | 2.6 Was an appropriate analysis used to estimate the effect of assignment to intervention? | | | PY |  |
|  | 2.7 If N/PN/NI to 2.6: Was there potential for a substantial impact (on the result) of the failure to analyse participants in the group to which they were randomized? | | | NA |  |
|  | **Risk of bias judgement** | | | **Low** |  |
| **Bias due to missing outcome data** | 3.1 Were data for this outcome available for all, or nearly all, participants randomized? | | | PY |  |
|  | 3.2 If N/PN/NI to 3.1: Is there evidence that result was not biased by missing outcome data? | | | NA |  |
|  | 3.3 If N/PN to 3.2: Could missingness in the outcome depend on its true value? | | | NA |  |
|  | 3.4 If Y/PY/NI to 3.3: Is it likely that missingness in the outcome depended on its true value? | | | NA |  |
|  | **Risk of bias judgement** | | | **Low** |  |
| **Bias in measurement of the outcome** | 4.1 Was the method of measuring the outcome inappropriate? | | | PN |  |
|  | 4.2 Could measurement or ascertainment of the outcome have differed between intervention groups? | | | PN |  |
|  | 4.3 Were outcome assessors aware of the intervention received by study participants? | | | NI | double blind |
|  | 4.4 If Y/PY/NI to 4.3: Could assessment of the outcome have been influenced by knowledge of intervention received? | | | PN |  |
|  | 4.5 If Y/PY/NI to 4.4: Is it likely that assessment of the outcome was influenced by knowledge of intervention received? | | | NA |  |
|  | **Risk of bias judgement** | | | **Low** |  |
| **Bias in selection of the reported result** | 5.1 Were the data that produced this result analysed in accordance with a pre-specified analysis plan that was finalized before unblinded outcome data were available for analysis? | | | PY |  |
|  | 5.2 ... multiple eligible outcome measurements (e.g. scales, definitions, time points) within the outcome domain? | | | PN |  |
|  | 5.3 ... multiple eligible analyses of the data? | | | PN |  |
|  | **Risk of bias judgement** | | | **Low** |  |
| **Overall bias** | **Risk of bias judgement** | | | **Low** |  |
| **Unique ID** | 34 | **Study ID** | NCT02065791 | **Assessor** | G and J |
| **Ref or Label** | 10.1056/NEJMoa1811744 | **Aim** | assignment to intervention (the 'intention-to-treat' effect) |  |  |
| **Experimental** | Canagliflozin | **Comparator** | PBO | **Source** |  |
| **Outcome** | ①②④⑤⑦⑧⑨⑩ | **Results** | ①②④⑤⑦⑧⑨⑩ | **Weight** | 1 |
| **Domain** | **Signalling question** | | | **Response** | **Comments** |
| **Bias arising from the randomization process** | 1.1 Was the allocation sequence random? | | | Y | Randomization is performed centrally through an interactive web response system using a computer-generated randomization schedule prepared by the study sponsor. |
|  | 1.2 Was the allocation sequence concealed until participants were enrolled and assigned to interventions? | | | Y |  |
|  | 1.3 Did baseline differences between intervention groups suggest a problem with the randomization process? | | | N |  |
|  | **Risk of bias judgement** | | | **Low** |  |
| **Bias due to deviations from intended interventions** | 2.1.Were participants aware of their assigned intervention during the trial? | | | N | Patients and all study staff will remain blinded to individual treatment allocation until the completion of the study. |
|  | 2.2.Were carers and people delivering the interventions aware of participants' assigned intervention during the trial? | | | N |  |
|  | 2.3. If Y/PY/NI to 2.1 or 2.2: Were there deviations from the intended intervention that arose because of the experimental context? | | | NA |  |
|  | 2.4 If Y/PY to 2.3: Were these deviations likely to have affected the outcome? | | | NA |  |
|  | 2.5. If Y/PY/NI to 2.4: Were these deviations from intended intervention balanced between groups? | | | NA |  |
|  | 2.6 Was an appropriate analysis used to estimate the effect of assignment to intervention? | | | Y |  |
|  | 2.7 If N/PN/NI to 2.6: Was there potential for a substantial impact (on the result) of the failure to analyse participants in the group to which they were randomized? | | | NA |  |
|  | **Risk of bias judgement** | | | **Low** |  |
| **Bias due to missing outcome data** | 3.1 Were data for this outcome available for all, or nearly all, participants randomized? | | | PY |  |
|  | 3.2 If N/PN/NI to 3.1: Is there evidence that result was not biased by missing outcome data? | | | NA |  |
|  | 3.3 If N/PN to 3.2: Could missingness in the outcome depend on its true value? | | | NA |  |
|  | 3.4 If Y/PY/NI to 3.3: Is it likely that missingness in the outcome depended on its true value? | | | NA |  |
|  | **Risk of bias judgement** | | | **Low** |  |
| **Bias in measurement of the outcome** | 4.1 Was the method of measuring the outcome inappropriate? | | | N | Patients and all study staff will remain blinded to individual treatment allocation until the completion of the study. |
|  | 4.2 Could measurement or ascertainment of the outcome have differed between intervention groups? | | | N |  |
|  | 4.3 Were outcome assessors aware of the intervention received by study participants? | | | PN |  |
|  | 4.4 If Y/PY/NI to 4.3: Could assessment of the outcome have been influenced by knowledge of intervention received? | | | NA |  |
|  | 4.5 If Y/PY/NI to 4.4: Is it likely that assessment of the outcome was influenced by knowledge of intervention received? | | | NA |  |
|  | **Risk of bias judgement** | | | **Low** |  |
| **Bias in selection of the reported result** | 5.1 Were the data that produced this result analysed in accordance with a pre-specified analysis plan that was finalized before unblinded outcome data were available for analysis? | | | PY |  |
|  | 5.2 ... multiple eligible outcome measurements (e.g. scales, definitions, time points) within the outcome domain? | | | PN |  |
|  | 5.3 ... multiple eligible analyses of the data? | | | PN |  |
|  | **Risk of bias judgement** | | | **Low** |  |
| **Overall bias** | **Risk of bias judgement** | | | **Low** |  |
| **Unique ID** | 35 | **Study ID** | NCT02540993 and NCT02065791 | **Assessor** | G and J |
| **Ref or Label** | 10.1093/ndt/gfab336 | **Aim** | assignment to intervention (the 'intention-to-treat' effect) |  |  |
| **Experimental** | Finerenone | **Comparator** | PBO | **Source** | Journal article(s) with results of the trial; Trial protocol; Non-commercial trial registry record (e.g. ClinicalTrials.gov record) |
| **Outcome** | ⑨⑩ | **Results** | ⑨⑩ | **Weight** | 1 |
| **Domain** | **Signalling question** | | | **Response** | **Comments** |
| **Bias arising from the randomization process** | 1.1 Was the allocation sequence random? | | | Y |  |
|  | 1.2 Was the allocation sequence concealed until participants were enrolled and assigned to interventions? | | | PY |  |
|  | 1.3 Did baseline differences between intervention groups suggest a problem with the randomization process? | | | N |  |
|  | **Risk of bias judgement** | | | **Low** |  |
| **Bias due to deviations from intended interventions** | 2.1.Were participants aware of their assigned intervention during the trial? | | | N |  |
|  | 2.2.Were carers and people delivering the interventions aware of participants' assigned intervention during the trial? | | | PN |  |
|  | 2.3. If Y/PY/NI to 2.1 or 2.2: Were there deviations from the intended intervention that arose because of the experimental context? | | | NA |  |
|  | 2.4 If Y/PY to 2.3: Were these deviations likely to have affected the outcome? | | | NA |  |
|  | 2.5. If Y/PY/NI to 2.4: Were these deviations from intended intervention balanced between groups? | | | NA |  |
|  | 2.6 Was an appropriate analysis used to estimate the effect of assignment to intervention? | | | PY |  |
|  | 2.7 If N/PN/NI to 2.6: Was there potential for a substantial impact (on the result) of the failure to analyse participants in the group to which they were randomized? | | | NA |  |
|  | **Risk of bias judgement** | | | **Low** |  |
| **Bias due to missing outcome data** | 3.1 Were data for this outcome available for all, or nearly all, participants randomized? | | | PY |  |
|  | 3.2 If N/PN/NI to 3.1: Is there evidence that result was not biased by missing outcome data? | | | NA |  |
|  | 3.3 If N/PN to 3.2: Could missingness in the outcome depend on its true value? | | | NA |  |
|  | 3.4 If Y/PY/NI to 3.3: Is it likely that missingness in the outcome depended on its true value? | | | NA |  |
|  | **Risk of bias judgement** | | | **Low** |  |
| **Bias in measurement of the outcome** | 4.1 Was the method of measuring the outcome inappropriate? | | | PN |  |
|  | 4.2 Could measurement or ascertainment of the outcome have differed between intervention groups? | | | PN |  |
|  | 4.3 Were outcome assessors aware of the intervention received by study participants? | | | PN |  |
|  | 4.4 If Y/PY/NI to 4.3: Could assessment of the outcome have been influenced by knowledge of intervention received? | | | NA |  |
|  | 4.5 If Y/PY/NI to 4.4: Is it likely that assessment of the outcome was influenced by knowledge of intervention received? | | | NA |  |
|  | **Risk of bias judgement** | | | **Low** |  |
| **Bias in selection of the reported result** | 5.1 Were the data that produced this result analysed in accordance with a pre-specified analysis plan that was finalized before unblinded outcome data were available for analysis? | | | PY |  |
|  | 5.2 ... multiple eligible outcome measurements (e.g. scales, definitions, time points) within the outcome domain? | | | PN |  |
|  | 5.3 ... multiple eligible analyses of the data? | | | PN |  |
|  | **Risk of bias judgement** | | | **Low** |  |
| **Overall bias** | **Risk of bias judgement** | | | **Low** |  |
| **Unique ID** | 36 | **Study ID** | NCT02065791 | **Assessor** | G and J |
| **Ref or Label** | 10.1111/jdi.13624 | **Aim** | assignment to intervention (the 'intention-to-treat' effect) |  |  |
| **Experimental** | Canagliflozin | **Comparator** | PBO | **Source** | Journal article(s) with results of the trial; Trial protocol; Non-commercial trial registry record (e.g. ClinicalTrials.gov record) |
| **Outcome** | ①②④⑤⑦⑨⑩ | **Results** | ①②④⑤⑦⑨⑩ | **Weight** | 1 |
| **Domain** | **Signalling question** | | | **Response** | **Comments** |
| **Bias arising from the randomization process** | 1.1 Was the allocation sequence random? | | | Y | Randomization is performed centrally through an interactive web response system using a computer-generated randomization schedule prepared by the study sponsor. |
|  | 1.2 Was the allocation sequence concealed until participants were enrolled and assigned to interventions? | | | Y |  |
|  | 1.3 Did baseline differences between intervention groups suggest a problem with the randomization process? | | | N |  |
|  | **Risk of bias judgement** | | | **Low** |  |
| **Bias due to deviations from intended interventions** | 2.1.Were participants aware of their assigned intervention during the trial? | | | N |  |
|  | 2.2.Were carers and people delivering the interventions aware of participants' assigned intervention during the trial? | | | N |  |
|  | 2.3. If Y/PY/NI to 2.1 or 2.2: Were there deviations from the intended intervention that arose because of the experimental context? | | | NA |  |
|  | 2.4 If Y/PY to 2.3: Were these deviations likely to have affected the outcome? | | | NA |  |
|  | 2.5. If Y/PY/NI to 2.4: Were these deviations from intended intervention balanced between groups? | | | NA |  |
|  | 2.6 Was an appropriate analysis used to estimate the effect of assignment to intervention? | | | Y |  |
|  | 2.7 If N/PN/NI to 2.6: Was there potential for a substantial impact (on the result) of the failure to analyse participants in the group to which they were randomized? | | | NA |  |
|  | **Risk of bias judgement** | | | **Low** |  |
| **Bias due to missing outcome data** | 3.1 Were data for this outcome available for all, or nearly all, participants randomized? | | | PY |  |
|  | 3.2 If N/PN/NI to 3.1: Is there evidence that result was not biased by missing outcome data? | | | NA |  |
|  | 3.3 If N/PN to 3.2: Could missingness in the outcome depend on its true value? | | | NA |  |
|  | 3.4 If Y/PY/NI to 3.3: Is it likely that missingness in the outcome depended on its true value? | | | NA |  |
|  | **Risk of bias judgement** | | | **Low** |  |
| **Bias in measurement of the outcome** | 4.1 Was the method of measuring the outcome inappropriate? | | | N |  |
|  | 4.2 Could measurement or ascertainment of the outcome have differed between intervention groups? | | | N |  |
|  | 4.3 Were outcome assessors aware of the intervention received by study participants? | | | PN |  |
|  | 4.4 If Y/PY/NI to 4.3: Could assessment of the outcome have been influenced by knowledge of intervention received? | | | NA |  |
|  | 4.5 If Y/PY/NI to 4.4: Is it likely that assessment of the outcome was influenced by knowledge of intervention received? | | | NA |  |
|  | **Risk of bias judgement** | | | **Low** |  |
| **Bias in selection of the reported result** | 5.1 Were the data that produced this result analysed in accordance with a pre-specified analysis plan that was finalized before unblinded outcome data were available for analysis? | | | PY |  |
|  | 5.2 ... multiple eligible outcome measurements (e.g. scales, definitions, time points) within the outcome domain? | | | PN |  |
|  | 5.3 ... multiple eligible analyses of the data? | | | PN |  |
|  | **Risk of bias judgement** | | | **Low** |  |
| **Overall bias** | **Risk of bias judgement** | | | **Low** |  |
| **Unique ID** | 37 | **Study ID** | NCT02540993 | **Assessor** | G and J |
| **Ref or Label** | 10.1159/000531997 | **Aim** | assignment to intervention (the 'intention-to-treat' effect) |  |  |
| **Experimental** | Finerenone | **Comparator** | PBO | **Source** | Journal article(s) with results of the trial; Trial protocol; Non-commercial trial registry record (e.g. ClinicalTrials.gov record) |
| **Outcome** | ⑨⑩ | **Results** | ⑨⑩ | **Weight** | 1 |
| **Domain** | **Signalling question** | | | **Response** | **Comments** |
| **Bias arising from the randomization process** | 1.1 Was the allocation sequence random? | | | NI | The only information about the randomization method is that the study was randomized |
|  | 1.2 Was the allocation sequence concealed until participants were enrolled and assigned to interventions? | | | PY |  |
|  | 1.3 Did baseline differences between intervention groups suggest a problem with the randomization process? | | | N |  |
|  | **Risk of bias judgement** | | | **Low** |  |
| **Bias due to deviations from intended interventions** | 2.1.Were participants aware of their assigned intervention during the trial? | | | N | Masking: Quadruple (Participant, Care Provider, Investigator, Outcomes Assessor) |
|  | 2.2.Were carers and people delivering the interventions aware of participants' assigned intervention during the trial? | | | N |  |
|  | 2.3. If Y/PY/NI to 2.1 or 2.2: Were there deviations from the intended intervention that arose because of the experimental context? | | | NA |  |
|  | 2.4 If Y/PY to 2.3: Were these deviations likely to have affected the outcome? | | | NA |  |
|  | 2.5. If Y/PY/NI to 2.4: Were these deviations from intended intervention balanced between groups? | | | NA |  |
|  | 2.6 Was an appropriate analysis used to estimate the effect of assignment to intervention? | | | PY |  |
|  | 2.7 If N/PN/NI to 2.6: Was there potential for a substantial impact (on the result) of the failure to analyse participants in the group to which they were randomized? | | | NA |  |
|  | **Risk of bias judgement** | | | **Low** |  |
| **Bias due to missing outcome data** | 3.1 Were data for this outcome available for all, or nearly all, participants randomized? | | | PY |  |
|  | 3.2 If N/PN/NI to 3.1: Is there evidence that result was not biased by missing outcome data? | | | NA |  |
|  | 3.3 If N/PN to 3.2: Could missingness in the outcome depend on its true value? | | | NA |  |
|  | 3.4 If Y/PY/NI to 3.3: Is it likely that missingness in the outcome depended on its true value? | | | NA |  |
|  | **Risk of bias judgement** | | | **Low** |  |
| **Bias in measurement of the outcome** | 4.1 Was the method of measuring the outcome inappropriate? | | | PN |  |
|  | 4.2 Could measurement or ascertainment of the outcome have differed between intervention groups? | | | PN |  |
|  | 4.3 Were outcome assessors aware of the intervention received by study participants? | | | N | Masking: Quadruple (Participant, Care Provider, Investigator, Outcomes Assessor) |
|  | 4.4 If Y/PY/NI to 4.3: Could assessment of the outcome have been influenced by knowledge of intervention received? | | | NA |  |
|  | 4.5 If Y/PY/NI to 4.4: Is it likely that assessment of the outcome was influenced by knowledge of intervention received? | | | NA |  |
|  | **Risk of bias judgement** | | | **Low** |  |
| **Bias in selection of the reported result** | 5.1 Were the data that produced this result analysed in accordance with a pre-specified analysis plan that was finalized before unblinded outcome data were available for analysis? | | | PY |  |
|  | 5.2 ... multiple eligible outcome measurements (e.g. scales, definitions, time points) within the outcome domain? | | | PN |  |
|  | 5.3 ... multiple eligible analyses of the data? | | | PN |  |
|  | **Risk of bias judgement** | | | **Low** |  |
| **Overall bias** | **Risk of bias judgement** | | | **Low** |  |
| **Unique ID** | 38 | **Study ID** | NCT02540993 and NCT02545049 | **Assessor** | G and J |
| **Ref or Label** | 10.1093/eurheartj/ehab777 | **Aim** | assignment to intervention (the 'intention-to-treat' effect) |  |  |
| **Experimental** | Finerenone | **Comparator** | PBO | **Source** | Journal article(s) with results of the trial; Trial protocol; Non-commercial trial registry record (e.g. ClinicalTrials.gov record) |
| **Outcome** | ④⑦⑧⑨⑩ | **Results** | ④⑦⑧⑨⑩ | **Weight** | 1 |
| **Domain** | **Signalling question** | | | **Response** | **Comments** |
| **Bias arising from the randomization process** | 1.1 Was the allocation sequence random? | | | NI | The only information about the randomization method is that the study was randomized. All the patients and study personnel (with the exception of the independent data monitoring committee) had no role in the treatment assignments |
|  | 1.2 Was the allocation sequence concealed until participants were enrolled and assigned to interventions? | | | Y |  |
|  | 1.3 Did baseline differences between intervention groups suggest a problem with the randomization process? | | | N |  |
|  | **Risk of bias judgement** | | | **Low** |  |
| **Bias due to deviations from intended interventions** | 2.1.Were participants aware of their assigned intervention during the trial? | | | N | Masking: Quadruple (Participant, Care Provider, Investigator, Outcomes Assessor) |
|  | 2.2.Were carers and people delivering the interventions aware of participants' assigned intervention during the trial? | | | N |  |
|  | 2.3. If Y/PY/NI to 2.1 or 2.2: Were there deviations from the intended intervention that arose because of the experimental context? | | | NA |  |
|  | 2.4 If Y/PY to 2.3: Were these deviations likely to have affected the outcome? | | | NA |  |
|  | 2.5. If Y/PY/NI to 2.4: Were these deviations from intended intervention balanced between groups? | | | NA |  |
|  | 2.6 Was an appropriate analysis used to estimate the effect of assignment to intervention? | | | PY |  |
|  | 2.7 If N/PN/NI to 2.6: Was there potential for a substantial impact (on the result) of the failure to analyse participants in the group to which they were randomized? | | | NA |  |
|  | **Risk of bias judgement** | | | **Low** |  |
| **Bias due to missing outcome data** | 3.1 Were data for this outcome available for all, or nearly all, participants randomized? | | | PY |  |
|  | 3.2 If N/PN/NI to 3.1: Is there evidence that result was not biased by missing outcome data? | | | NA |  |
|  | 3.3 If N/PN to 3.2: Could missingness in the outcome depend on its true value? | | | NA |  |
|  | 3.4 If Y/PY/NI to 3.3: Is it likely that missingness in the outcome depended on its true value? | | | NA |  |
|  | **Risk of bias judgement** | | | **Low** |  |
| **Bias in measurement of the outcome** | 4.1 Was the method of measuring the outcome inappropriate? | | | PN |  |
|  | 4.2 Could measurement or ascertainment of the outcome have differed between intervention groups? | | | PN |  |
|  | 4.3 Were outcome assessors aware of the intervention received by study participants? | | | N | Masking: Quadruple (Participant, Care Provider, Investigator, Outcomes Assessor) |
|  | 4.4 If Y/PY/NI to 4.3: Could assessment of the outcome have been influenced by knowledge of intervention received? | | | NA |  |
|  | 4.5 If Y/PY/NI to 4.4: Is it likely that assessment of the outcome was influenced by knowledge of intervention received? | | | NA |  |
|  | **Risk of bias judgement** | | | **Low** |  |
| **Bias in selection of the reported result** | 5.1 Were the data that produced this result analysed in accordance with a pre-specified analysis plan that was finalized before unblinded outcome data were available for analysis? | | | PY |  |
|  | 5.2 ... multiple eligible outcome measurements (e.g. scales, definitions, time points) within the outcome domain? | | | PN |  |
|  | 5.3 ... multiple eligible analyses of the data? | | | PN |  |
|  | **Risk of bias judgement** | | | **Low** |  |
| **Overall bias** | **Risk of bias judgement** | | | **Low** |  |
| **Unique ID** | 39 | **Study ID** | NCT02540993 and NCT02545049 | **Assessor** | G and J |
| **Ref or Label** | 10.1111/dom.15305 | **Aim** | assignment to intervention (the 'intention-to-treat' effect) |  |  |
| **Experimental** | Finerenone | **Comparator** | PBO | **Source** | Journal article(s) with results of the trial; Trial protocol; Non-commercial trial registry record (e.g. ClinicalTrials.gov record) |
| **Outcome** | ⑩ | **Results** | ⑩ | **Weight** | 1 |
| **Domain** | **Signalling question** | | | **Response** | **Comments** |
| **Bias arising from the randomization process** | 1.1 Was the allocation sequence random? | | | NI | The only information about the randomization method is that the study was randomized. All the patients and study personnel (with the exception of the independent data monitoring committee) had no role in the treatment assignments |
|  | 1.2 Was the allocation sequence concealed until participants were enrolled and assigned to interventions? | | | Y |  |
|  | 1.3 Did baseline differences between intervention groups suggest a problem with the randomization process? | | | N |  |
|  | **Risk of bias judgement** | | | **Low** |  |
| **Bias due to deviations from intended interventions** | 2.1.Were participants aware of their assigned intervention during the trial? | | | N | Masking: Quadruple (Participant, Care Provider, Investigator, Outcomes Assessor) |
|  | 2.2.Were carers and people delivering the interventions aware of participants' assigned intervention during the trial? | | | N |  |
|  | 2.3. If Y/PY/NI to 2.1 or 2.2: Were there deviations from the intended intervention that arose because of the experimental context? | | | NA |  |
|  | 2.4 If Y/PY to 2.3: Were these deviations likely to have affected the outcome? | | | NA |  |
|  | 2.5. If Y/PY/NI to 2.4: Were these deviations from intended intervention balanced between groups? | | | NA |  |
|  | 2.6 Was an appropriate analysis used to estimate the effect of assignment to intervention? | | | PY |  |
|  | 2.7 If N/PN/NI to 2.6: Was there potential for a substantial impact (on the result) of the failure to analyse participants in the group to which they were randomized? | | | NA |  |
|  | **Risk of bias judgement** | | | **Low** |  |
| **Bias due to missing outcome data** | 3.1 Were data for this outcome available for all, or nearly all, participants randomized? | | | PY |  |
|  | 3.2 If N/PN/NI to 3.1: Is there evidence that result was not biased by missing outcome data? | | | NA |  |
|  | 3.3 If N/PN to 3.2: Could missingness in the outcome depend on its true value? | | | NA |  |
|  | 3.4 If Y/PY/NI to 3.3: Is it likely that missingness in the outcome depended on its true value? | | | NA |  |
|  | **Risk of bias judgement** | | | **Low** |  |
| **Bias in measurement of the outcome** | 4.1 Was the method of measuring the outcome inappropriate? | | | PN |  |
|  | 4.2 Could measurement or ascertainment of the outcome have differed between intervention groups? | | | PN |  |
|  | 4.3 Were outcome assessors aware of the intervention received by study participants? | | | N | Masking: Quadruple (Participant, Care Provider, Investigator, Outcomes Assessor) |
|  | 4.4 If Y/PY/NI to 4.3: Could assessment of the outcome have been influenced by knowledge of intervention received? | | | NA |  |
|  | 4.5 If Y/PY/NI to 4.4: Is it likely that assessment of the outcome was influenced by knowledge of intervention received? | | | NA |  |
|  | **Risk of bias judgement** | | | **Low** |  |
| **Bias in selection of the reported result** | 5.1 Were the data that produced this result analysed in accordance with a pre-specified analysis plan that was finalized before unblinded outcome data were available for analysis? | | | PY |  |
|  | 5.2 ... multiple eligible outcome measurements (e.g. scales, definitions, time points) within the outcome domain? | | | PN |  |
|  | 5.3 ... multiple eligible analyses of the data? | | | PN |  |
|  | **Risk of bias judgement** | | | **Low** |  |
| **Overall bias** | **Risk of bias judgement** | | | **Low** |  |

**Appendix 5** Assessments of Inconsistencies (**Inconsistency Test Based on Side-Splitting Approach**)

**a. Glycosylated hemoglobin (HbA1c)**

| **Side** | **Direct** |  | **Indirect** |  | **Difference** |  |  |
| --- | --- | --- | --- | --- | --- | --- | --- |
|  | **Coef.** | **Std. Err.** | **Coef.** | **Std. Err.** | **Coef.** | **Std. Err.** | **P>\|z\|** |
| A I * | .4466374 | .2229837 | .1177926 | 5.2700670 | .3288448 | 5.2747820 | 0.950 |
| B I * | .2396864 | .0920700 | .8913647 | 23.9154900 | −.6516783 | 23.9156800 | 0.978 |
| C I * | .1811908 | .1310821 | .8799261 | 31.6243000 | −.6987352 | 31.6245900 | 0.982 |
| D I * | .3350001 | .0698424 | .8525556 | 20.0090500 | −.5175555 | 20.0091800 | 0.979 |
| E I * | .1782375 | .1057078 | .8941022 | 31.6146000 | −.7158647 | 31.6147800 | 0.982 |
| F I * | −.0198746 | .1179452 | .8915006 | 36.5058800 | −.9113752 | 36.5060700 | 0.980 |
| G I * | .3735851 | .2403288 | .8932304 | 44.7252200 | −.5196453 | 44.7258500 | 0.991 |
| H I * | .2402659 | .2594306 | .8175633 | 63.2547500 | −.5772974 | 63.2554300 | 0.993 |
| I J * | −.2157498 | .3219627 | −.8921334 | 63.2394400 | .6763836 | 63.2402600 | 0.991 |
| I K * | −.0876807 | .1130079 | −.8922048 | 31.6076900 | .8045241 | 31.6078900 | 0.980 |

A: Bexagliflozin; B: Canagliflozin; C: Dapagliflozin; D: Empagliflozin; E: Ertugliflozin; F: Finerenone; G: Liraglutide; H: Luseogliflozin; I: PBO; J: Semaglutide; K: Sotagliflozin.

**b. Estimate glomerular filtration rate (eGFR)**

| **Side** | **Direct** |  | **Indirect** |  | **Difference** |  | **P>\|z\|** |
| --- | --- | --- | --- | --- | --- | --- | --- |
|  | **Coef.** | **Std. Err.** | **Coef.** | **Std. Err** | **Coef.** | **Std. Err** |  |
| A J * | .321686 | .273889 | .0089754 | 5.198498 | .3127106 | 5.205709 | 0.952 |
| B J * | .046643 | .0990551 | .6494112 | 21.08417 | −.6027683 | 21.08441 | 0.977 |
| C J * | .1730319 | .153337 | .6336665 | 31.62473 | −.4606346 | 31.62511 | 0.988 |
| D J * | −.0697665 | .129859 | .6598375 | 28.29203 | −.729604 | 28.29234 | 0.979 |
| E J * | .2316044 | .1323304 | .6455644 | 31.62748 | −.41396 | 31.62775 | 0.990 |
| F J * | .0115717 | .1854032 | .6447585 | 44.72114 | −.6331868 | 44.72152 | 0.989 |
| G J * | .0081426 | .1209887 | .6397261 | 28.28509 | −.6315835 | 28.28536 | 0.982 |
| H J * | −.7673318 | .2727157 | .6387613 | 44.73217 | −1.406093 | 44.733 | 0.975 |
| I J * | .3202685 | .3034199 | .5422647 | 63.25926 | −.2219962 | 63.26013 | 0.997 |
| J K * | .1965161 | .3034199 | −.6417039 | 63.24723 | .83822 | 63.24824 | 0.989 |
| J L * | −.0856333 | .3034199 | −.639845 | 31.6347 | .5542117 | 31.63501 | 0.986 |

A: Bexagliflozin; B: Canagliflozin; C: Dapagliflozin; D: Empagliflozin; E: Ertugliflozinl; F: Exenatide; G: Finerenone; H: Liraglutide; I: Luseogliflozin; J: PBO; K: Semaglutide; L: Sotagliflozin.

**c. Low−Density Lipoprotein Cholesterol (LDL−C)**

| **Side** | **Direct** |  | **Indirect** |  | **Difference** |  |  |
| --- | --- | --- | --- | --- | --- | --- | --- |
|  | **Coef.** | **Std. Err.** | **Coef.** | **Std. Err.** | **Coef.** | **Std. Err.** | **P>\|z\|** |
| A F * | .055023 | .0591282 | .068368 | 9.536667 | −.013345 | 9.536848 | 0.999 |
| B F * | −.0803346 | .1276014 | .1116811 | 44.70312 | −.1920157 | 44.70331 | 0.997 |
| C F * | −.0516284 | .0370046 | .1193548 | 23.92221 | −.1709832 | 23.92223 | 0.994 |
| D F * | 2.041465 | .3235343 | .1435069 | 63.24242 | 1.897958 | 63.24322 | 0.976 |
| E F * | .0655671 | .180506 | .0896985 | 63.26148 | −.0241313 | 63.26189 | 1.000 |

A: Canagliflozin; B: Dapagliflozin; C: Empagliflozin; D: Liraglutide; E: Luseogliflozin; F: PBO.

**d. Systolic blood pressure (SBP)**

| **Side** | **Direct** |  | **Indirect** |  | **Difference** |  |  |
| --- | --- | --- | --- | --- | --- | --- | --- |
|  | **Coef.** | **Std. Err.** | **Coef.** | **Std. Err.** | **Coef.** | **Std. Err.** | **P>\|z\|** |
| A I * | .3882831 | .1188976 | .0949971 | −.7761772 | .293286 | 5.131031 | 0.954 |
| B I * | .2326156 | .0529576 | .772444 | 25.8088 | −.5398285 | 25.80886 | 0.983 |
| C I * | .2176514 | .0643432 | .7742798 | 28.28374 | −.5566283 | 28.28381 | 0.984 |
| D I * | .310044 | .0286185 | .7271109 | 21.06064 | −.4170669 | 21.06066 | 0.984 |
| E I * | .1014809 | .0439067 | .7774499 | 36.37614 | −.675969 | 36.37616 | 0.985 |
| F I * | .0994736 | .0263981 | .7751701 | 21.0754 | −.6756965 | 21.07542 | 0.974 |
| G I * | .0925272 | .2574105 | .7777882 | 63.24022 | −.685261 | 63.24073 | 0.991 |
| H I * | .0457622 | .176427 | .7620557 | 63.25782 | −.7162936 | 63.25822 | 0.991 |
| I J * | −.0277996 | .0584681 | −.7761772 | 31.61617 | .7483776 | 31.61622 | 0.981 |

A: Bexagliflozin; B: Canagliflozin; C: Dapagliflozin; D: Empagliflozin; E: Ertugliflozin; F: Finerenone; G: Liraglutide; H: Luseogliflozin; I: PBO; J: Sotagliflozin

**e. Diastolic blood pressure (DBP)**

| **Side** | **Direct** |  | **Indirect** |  | **Difference** |  |  |
| --- | --- | --- | --- | --- | --- | --- | --- |
|  | **Coef.** | **Std. Err.** | **Coef.** | **Std. Err.** | **Coef.** | **Std. Err.** | **P>\|z\|** |
| A F * | .1928961 | .1040256 | .0837555 | 9.999776 | .1091406 | 10.00032 | 0.991 |
| B F * | .1236088 | .124552 | .3838171 | 36.51747 | −.2602083 | 36.51769 | 0.994 |
| C F * | .2310157 | .0918813 | .3838171 | 28.27611 | −.140572 | 28.27627 | 0.996 |
| D F * | −.1277405 | .2847814 | .3836814 | 63.23744 | −.5114218 | 63.23806 | 0.994 |
| E F * | .1511195 | .2144103 | .3388635 | 63.23744 | −.187744 | 63.2555 | 0.998 |

A: Canagliflozin; B: Dapagliflozin; C: Empagliflozin; D: Liraglutide; E: Luseogliflozin; F: PBO.

**f. Body Weight**

| **Side** | **Direct** |  | **Indirect** |  | **Difference** |  |  |
| --- | --- | --- | --- | --- | --- | --- | --- |
|  | **Coef.** | **Std. Err.** | **Coef.** | **Std. Err.** | **Coef.** | **Std. Err.** | **P>\|z\|** |
| A H * | .0619617 | .160815 | .0868831 | 5.97717 | −.0249215 | 5.979333 | 0.997 |
| B H * | .0619617 | .0951815 | .1277369 | 36.532 | .0599745 | 36.53212 | 0.999 |
| C H * | −.1369148 | .1206372 | .1294602 | 36.53332 | −.266375 | 36.53354 | 0.994 |
| D H * | .3788315 | .0497716 | .0662537 | 21.07625 | .3125778 | 21.07632 | 0.988 |
| E H * | .1432265 | .0793801 | .1284874 | 36.52963 | .014739 | 36.52971 | 1.000 |
| F H * | −.0028521 | .0657468 | .1241284 | 31.61922 | −.1269806 | 31.61929 | 0.997 |
| G H * | .1636383 | .2078118 | .0731732 | 63.23319 | .0904651 | 63.23368 | 0.999 |
| H I * | −.1412066 | .2817997 | −.1239592 | 63.26039 | −.0172474 | 63.26102 | 1.000 |
| H J * | −.0130895 | .0815019 | −.1238888 | 31.60816 | .1107993 | 31.60826 | 0.997 |

A: Bexagliflozin; B: Canagliflozin; C: Dapagliflozin; D: Empagliflozin; E: Ertugliflozin; F: Finerenone; G: Luseogliflozin; H: PBO; I: Semaglutide; J: Sotagliflozin.

**g. Adverse Events (any AE)**

| **Side** | **Direct** |  | **Indirect** |  | **Difference** |  |  |  |
| --- | --- | --- | --- | --- | --- | --- | --- | --- |
|  | **Coef.** | **Std. Err.** | **Coef.** | **Std. Err.** | **Coef.** | **Std. Err.** | **P>\|z\|** |  |
| A I * | .1793891 | .0368538 | .0066138 | 12.24423 | .1727754 | 12.24428 | 0.989 |  |
| B I * | .0934933 | .1545235 | .3581873 | 84.44151 | −.264694 | 84.44166 | 0.997 |  |
| C I * | .2036221 | .1259349 | .3470078 | 71.68172 | −.1433857 | 71.68187 | 0.998 |  |
| D I * | −.0365337 | .1063207 | .3711301 | 96.25067 | −.4076638 | 96.25077 | 0.997 |  |
| E I * | −.2311189 | .0907078 | .3855202 | 79.18923 | −.6166392 | 79.18929 | 0.994 |  |
| F I * | .0281246 | .0189019 | .3543853 | 37.39392 | −.3262606 | 37.39393 | 0.993 |  |
| G I * | −1.54e−10 | .5829831 | .3587458 | 132.9392 | −.3587458 | 132.9405 | 0.998 |  |
| H I * | −.2353477 | .3643799 | .4280517 | 133.0317 | −.6633994 | 133.0325 | 0.996 |  |
| I J * | −.0337715 | .0402089 | −.3585276 | 68.04415 | .3247561 | 68.04417 | 0.996 |  |

A: Canagliflozin; B: Dapagliflozin; C: Empagliflozin; D: Ertugliflozin; E: Exenatide; F: Finerenone; G: Liraglutide; H: Luseogliflozin; I: PBO; J: Sotagliflozin.**h**

**h.Urinary tract infection (UTI)**

| **Side** | **Direct** |  | **Indirect** |  | **Difference** |  |  |
| --- | --- | --- | --- | --- | --- | --- | --- |
|  | **Coef.** | **Std. Err.** | **Coef.** | **Std. Err.** | **Coef.** | **Std. Err.** | **P>\|z\|** |
| A H * | −.815486 | .5517473 | −.0298158 | 17.26952 | −.7856702 | 17.27833 | 0.964 |
| B H * | −.1119147 | .0537793 | −1.620892 | 73.61355 | 1.508977 | 73.61358 | 0.984 |
| C H * | .0668552 | .298035 | −1.651864 | 149.3188 | 1.71872 | 149.3192 | 0.991 |
| D H * | .002003 | .044039 | −1.67908 | 59.76656 | 1.681083 | 59.76658 | 0.978 |
| E H * | −.0228073 | .0991385 | −1.636368 | 95.19253 | 1.613561 | 95.19261 | 0.986 |
| F H * | .0173226 | .0452118 | −1.63255 | 114.1586 | 1.649872 | 114.1586 | 0.988 |
| G H * | −1.340675 | 1.521948 | −.8928856 | 388.6579 | −.4477893 | 388.6653 | 0.999 |
| H I * | 1.677261 | 1.570947 | 1.042708 | 294.7856 | .6345523 | 294.7952 | 0.998 |
| H J * | .0431002 | .05708 | 1.627936 | 89.08092 | −1.584836 | 89.08095 | 0.986 |

A: Bexagliflozin; B: Canagliflozin; C: Dapagliflozin; D: Empagliflozin; E: Ertugliflozin; F: Finerenone; G: Luseogliflozin; H: PBO; I: Semaglutide; J: Sotagliflozin.

**i.** **Hypoglycemia**

| **Side** | **Direct** |  | **Indirect** |  | **Difference** |  |  |
| --- | --- | --- | --- | --- | --- | --- | --- |
|  | **Coef.** | **Std. Err.** | **Coef.** | **Std. Err.** | **Coef.** | **Std. Err.** | **P>\|z\|** |
| A J * | −.0174648 | .2678244 | .0530364 | 13.77695 | −.0705012 | 13.77956 | 0.996 |
| B J * | .0676671 | .0732471 | −.0344658 | 91.16662 | .102133 | 91.16665 | 0.999 |
| C J * | .2706197 | .2102189 | −.0947895 | 104.2662 | .3654093 | 104.2665 | 0.997 |
| D J * | .1213352 | .0591847 | −.0601521 | 48.32194 | .1814873 | 48.32188 | 0.997 |
| E J * | .0616987 | .0806882 | −.0546568 | 76.20817 | .1163555 | 76.20824 | 0.999 |
| F J * | .2469557 | .1326689 | −.0458983 | 122.9116 | .292854 | 122.9116 | 0.998 |
| G J * | .1648139 | .051927 | −.0345341 | 100.7646 | .199348 | 100.7646 | 0.998 |
| H J * | −.4685667 | .5669147 | .0053234 | 135.2326 | −.4738901 | 135.2342 | 0.997 |
| I J * | .671641 | .8375943 | −.1455967 | 317.6269 | .8172376 | 317.6281 | 0.998 |
| J K * | −.7282385 | 1.254172 | −.0825953 | 290.2737 | −.6456432 | 290.2747 | 0.998 |
| J L * | −.0097029 | .0684475 | .0376022 | 62.57354 | −.0473051 | 62.5736 | 0.999 |

A: Bexagliflozin; B: Canagliflozin; C: Dapagliflozin; D: Empagliflozin; E: Ertugliflozin; F: Exenatide; G: Finerenone; H: Liraglutide; I: Luseogliflozin; J: PBO; K: Semaglutide; L: Sotagliflozin.

**j. Acute kidney injury (AKI)**

| **Side** | **Direct** |  | **Indirect** |  | **Difference** |  |  |
| --- | --- | --- | --- | --- | --- | --- | --- |
|  | **Coef.** | **Std. Err.** | **Coef.** | **Std. Err.** | **Coef.** | **Std. Err.** | **P>\|z\|** |
| A F * | −.2876821 | .5523491 | .0593939 | 34.38616 | −.3470759 | 34.3906 | 0.992 |
| B F * | .134315 | .0859164 | −.5741966 | 115.0821 | .7085116 | 115.0822 | 0.995 |
| C F * | .1801711 | .1626295 | −.6284564 | 162.8374 | .8086275 | 162.8375 | 0.996 |
| D F * | −.2107056 | 1.156033 | −.7967921 | 813.0846 | .5860865 | 813.0859 | 0.999 |
| E F * | .0629263 | .0494529 | −.5778834 | 100.6762 | .6408096 | 100.6762 | 0.995 |

A: Bexagliflozin; B: Canagliflozin; C: Ertugliflozin; D: Exenatide; E: Finerenone; F: PBO.

**Appendix 6** **League Table of each outcome**

a. **League Table of** HbA1c.

| Empagliflozin |  |  |  |  |  |  |  |  |  |  |
| --- | --- | --- | --- | --- | --- | --- | --- | --- | --- | --- |
| 0.37  (−1.29, 2.02) | Semaglutide |  |  |  |  |  |  |  |  |  |
| −0.00  (−0.22, 0.21) | −0.37  (−2.03, 1.29) | Canagliflozin |  |  |  |  |  |  |  |  |
| −0.02  (−0.38, 0.33) | −0.39  (−2.07, 1.29) | −0.02  (−0.41, 0.36) | Bexagliflozin |  |  |  |  |  |  |  |
| −0.07  (−0.38, 0.25) | −0.44  (−2.11, 1.24) | −0.07  (−0.41, 0.28) | −0.05  (−0.49, 0.40) | Dapagliflozin |  |  |  |  |  |  |
| −0.08  (−0.49, 0.33) | −0.45  (−2.14, 1.25) | −0.08  (−0.51, 0.36) | −0.06  (−0.57, 0.46) | −0.01  (−0.49, 0.47) | Liraglutide |  |  |  |  |  |
| −0.16  (−0.57, 0.24) | −0.53  (−2.22, 1.16) | −0.16  (−0.59, 0.27) | −0.14  (−0.66, 0.38) | −0.09  (−0.58, 0.39) | −0.08  (−0.63, 0.47) | Luseogliflozin |  |  |  |  |
| −0.16  (−0.37, 0.05) | −0.52  (−2.18, 1.13) | −0.16  (−0.41, 0.09) | −0.13  (−0.51, 0.24) | −0.09  (−0.43, 0.25) | −0.08  (−0.51, 0.35) | 0.01  (−0.42, 0.43) | Ertugliflozin |  |  |  |
| −0.19  (−0.49, 0.10) | −0.56  (−2.23, 1.11) | −0.19  (−0.52, 0.14) | −0.17  (−0.60, 0.26) | −0.12  (−0.52, 0.27) | −0.11  (−0.58, 0.36) | −0.03  (−0.50, 0.45) | −0.03  (−0.35, 0.29) | Sotagliflozin |  |  |
| **−0.33**  **(−0.45, −0.22)** | −0.7  (−2.35, 0.95) | **−0.33**  **(****−0.52, −0.15)** | −0.31  (−0.65, 0.03) | −0.26  (−0.56, 0.03) | −0.25  (−0.64, 0.13) | −0.17  (−0.56, 0.22) | −0.18  (−0.35, −0.00) | −0.14  (−0.41, 0.13) | PBO |  |
| **−0.38**  **(−0.62, −0.14)** | −0.74  (−2.40, 0.92) | **−0.38**  **(−0.65, −0.10)** | −0.35  (−0.75, 0.04) | −0.31  (−0.66, 0.05) | −0.3  (−0.73, 0.14) | −0.21  (−0.65, 0.23) | −0.22  (−0.49, 0.05) | −0.19  (−0.52, 0.15) | −0.04  (−0.25, 0.16) | Finerenone |

b. **League Table of** eGFR.

| Semaglutide |  |  |  |  |  |  |  |  |  |  |  |
| --- | --- | --- | --- | --- | --- | --- | --- | --- | --- | --- | --- |
| 2.73  (−8.85, 14.30) | Empagliflozin |  |  |  |  |  |  |  |  |  |  |
| 4.00  (−7.22, 15.22) | 1.27  (−1.57, 4.11) | PBO |  |  |  |  |  |  |  |  |  |
| 4.17  (−7.24, 15.58) | 1.44  (−2.01, 4.90) | 0.17  (−1.92, 2.26) | Canagliflozin |  |  |  |  |  |  |  |  |
| 4.25  (−7.23, 15.73) | 1.52  (−2.23, 5.28) | 0.25  (−2.17, 2.67) | 0.08  (−3.16, 3.32) | Finerenone |  |  |  |  |  |  |  |
| 4.28  (−7.59, 16.15) | 1.55  (−3.25, 6.36) | 0.28  (−3.60, 4.16) | 0.11  (−4.29, 4.51) | 0.03  (−4.55, 4.61) | Exenatide |  |  |  |  |  |  |
| 4.30  (−8.43, 17.03) | 1.57  (−5.07, 8.22) | 0.30  (−5.71, 6.31) | 0.13  (−6.23, 6.49) | 0.05  (−6.43, 6.53) | 0.02  (−7.13, 7.17) | Liraglutide |  |  |  |  |  |
| 4.85  (−6.68, 16.39) | 2.13  (−1.77, 6.02) | 0.85  (−1.81, 3.52) | 0.68  (−2.71, 4.07) | 0.60  (−3.00, 4.21) | 0.57  (−4.14, 5.28) | 0.55  (−6.02, 7.12) | Sotagliflozin |  |  |  |  |
| 6.31  (−5.39, 18.01) | 3.59  (−0.79, 7.96) | 2.31  (−1.01, 5.63) | 2.14  (−1.78, 6.07) | 2.06  (−2.05, 6.18) | 2.03  (−3.08, 7.14) | 2.01  (−4.85, 8.88) | 1.46  (−2.80, 5.72) | Dapagliflozin |  |  |  |
| 6.90  (−5.64, 19.44) | 4.17  (−2.10, 10.45) | 2.90  (−2.70, 8.50) | 2.73  (−3.24, 8.70) | 2.65  (−3.45, 8.75) | 2.62  (−4.19, 9.43) | 2.60  (−5.61, 10.81) | 2.05  (−4.15, 8.25) | 0.59  (−5.92, 7.10) | Luseogliflozin |  |  |
| 6.89  (−5.47, 19.24) | 4.16  (−1.74, 10.06) | 2.89  (−2.28, 8.06) | 2.72  (−2.86, 8.30) | 2.64  (−3.07, 8.35) | 2.61  (−3.86, 9.07) | 2.59  (−5.34, 10.51) | 2.03  (−3.78, 7.85) | 0.58  (−5.57, 6.72) | −0.01  (−7.63, 7.61) | Bexagliflozin |  |
| 6.42  (−5.08, 17.93) | 3.70  (−0.13, 7.52) | 2.42  (−0.13, 4.98) | 2.25  (−1.05, 5.56) | 2.17  (−1.35, 5.70) | 2.14  (−2.50, 6.79) | 2.12  (−4.40, 8.65) | 1.57  (−2.12, 5.27) | 0.11  (−4.08, 4.31) | −0.48  (−6.63, 5.68) | −0.46  (−6.23, 5.31) | Ertugliflozin |

**c. League Table of LDL-C.**

| Liraglutide |  |  |  |  |  |
| --- | --- | --- | --- | --- | --- |
| **−1.45 (****−1.87, −1.04)** | Canagliflozin |  |  |  |  |
| **−1.41 (****−2.01, −0.81)** | 0.05 (−0.42, 0.52) | Luseoglifozin |  |  |  |
| **−1.50 (−1.89, −1.11)** | −0.05 (−0.18, 0.08) | −0.09 (−0.55, 0.36) | PBO |  |  |
| **−1.58 (****−2.05, −1.10)** | −0.12 (−0.42, 0.18) | −0.17 (−0.70, 0.35) | −0.08 (−0.34, 0.19) | Dapagliflozin |  |
| **−1.56 (****−1.96, −1.15)** | −0.10 (−0.26, 0.06) | −0.15 (−0.61, 0.31) | −0.06 (−0.15, 0.04) | 0.02 (−0.27, 0.30) | Empagliflozin |

**d. League Table of SBP**

| Bexagliflozin |  |  |  |  |  |  |  |  |  |
| --- | --- | --- | --- | --- | --- | --- | --- | --- | --- |
| −1.19  (−4.56, 2.19) | Empagliflozin |  |  |  |  |  |  |  |  |
| −1.73  (−5.64, 2.17) | −0.55  (−2.81, 1.72) | Dapagliflozin |  |  |  |  |  |  |  |
| −2.36  (−5.93, 1.21) | −1.17  (−2.76, 0.41) | −0.63  (−3.17, 1.92) | Canagliflozin |  |  |  |  |  |  |
| −3.32  (−15.61, 8.97) | −2.13  (−14.00, 9.74) | −1.59  (−13.62, 10.45) | −0.96  (−12.89, 10.97) | Liraglutide |  |  |  |  |  |
| **−3.87**  **(−7.26, −0.49)** | **−2.69**  **(−3.78, −1.59)** | −2.14  (−4.42, 0.14) | −1.51  (−3.13, 0.10) | −0.55  (−12.43, 11.32) | Finerenone |  |  |  |  |
| **−4.01**  **(−7.53, −0.50)** | **−2.83**  **(−4.33, −1.32)** | −2.28  (−4.75, 0.19) | −1.65  (−3.55, 0.24) | −0.70  (−12.61, 11.22) | −0.14  (−1.67, 1.38) | Ertugliflozin |  |  |  |
| −4.72  (−11.10, 1.66) | −3.53  (−9.06, 2.00) | −2.99  (−8.85, 2.88) | −2.36  (−8.01, 3.29) | −1.40  (−14.45, 11.65) | −0.85  (−6.38, 4.69) | −0.70  (−6.32, 4.91) | Luseogliflozin |  |  |
| **−4.98**  **(−9.10, −0.87)** | **−3.80**  **(−6.41, −1.19)** | −3.25  (−6.52, 0.02) | −2.62  (−5.48, 0.23) | −1.66  (−13.77, 10.44) | −1.11  (−3.73, 1.51) | −0.97  (−3.76, 1.82) | −0.26  (−6.27, 5.75) | Sotagliflozin |  |
| **−5.52**  **(****−8.80, −2.24)** | **−4.33**  **(****−5.13, −3.53)** | **−3.79**  **(****−5.91, −1.66)** | **−3.16**  **(****−4.56, −1.75)** | −2.20  (−14.05, 9.64) | **−1.65**  **(****−2.48, −0.81)** | **−1.50**  **(****−2.78, −0.23)** | −0.80  (−6.27, 4.67) | −0.54  (−3.02, 1.95) | PBO |

**e. League Table of DBP.**

| Empagliflozin |  |  |  |  |  |
| --- | --- | --- | --- | --- | --- |
| −0.27 (−2.34, 1.79) | Canagliflozin |  |  |  |  |
| −0.26 (−4.63, 4.11) | 0.01 (−4.45, 4.48) | Luseogliflozin |  |  |  |
| −0.55 (−3.15, 2.06) | −0.27 (−3.03, 2.48) | −0.29 (−5.02, 4.45) | Dapagliflozin |  |  |
| −3.66 (−10.95, 3.62) | −3.39 (−10.73, 3.95) | −3.40 (−11.69, 4.89) | −3.11 (−10.62, 4.39) | Liraglutide |  |
| **−1.86 (−3.18, −40.54)** | −1.59 (−3.19, 0.02) | −1.60 (−5.77, 2.57) | −1.31 (−3.55, 0.92) | 1.80 (−5.37, 8.96) | PBO |

**f. League Table of Body Weight.**

| Canagliflozin |  |  |  |  |  |  |  |  |  |
| --- | --- | --- | --- | --- | --- | --- | --- | --- | --- |
| −1.45  (−4.40, 1.50) | Ertugliflozin |  |  |  |  |  |  |  |  |
| −1.67  (−6.62, 3.29) | −0.22  (−4.74, 4.31) | Luseogliflozin |  |  |  |  |  |  |  |
| −2.52  (−5.05, 0.02) | −1.07  (−2.59, 0.46) | −0.85  (−5.11, 3.41) | Empagliflozin |  |  |  |  |  |  |
| −2.21  (−8.41, 3.99) | −0.76  (−6.62, 5.10) | −0.54  (−7.62, 6.54) | 0.31  (−5.35, 5.97) | Semaglutide |  |  |  |  |  |
| −2.51  (−7.85, 2.83) | −1.07  (−6.01, 3.87) | −0.85  (−7.19, 5.50) | 0.00  (−4.70, 4.70) | −0.31  (−7.66, 7.05) | Bexagliflozin |  |  |  |  |
| **−3.55**  **(−6.75, −0.34)** | −2.10  (−4.58, 0.38) | −1.88  (−6.57, 2.81) | −1.03  (−3.00, 0.94) | −1.34  (−7.33, 4.65) | −1.03  (−6.13, 4.06) | Sotagliflozin |  |  |  |
| **−3.81**  **(−6.34, −1.27)** | **−2.36**  **(−3.87, −0.84)** | −2.14  (−6.40, 2.12) | **−1.29**  **(−1.42, −1.16)** | −1.60  (−7.26, 4.06) | −1.29  (−5.99, 3.41) | −0.26  (−2.22, 1.70) | PBO |  |  |
| **−3.94**  **(−6.73, −1.16)** | **−2.50**  **(−4.40, −0.59)** | −2.28  (−6.69, 2.14) | **−1.43**  **(−2.58, −0.28)** | −1.74  (−7.51, 4.04) | −1.43  (−6.27, 3.41) | −0.40  (−2.67, 1.88) | −0.14  (−1.28, 1.01) | Finerenone |  |
| **−6.44**  **(−10.52, −2.37)** | **−4.99**  **(−8.53, −1.46)** | −4.78  (−10.10, 0.55) | **−3.93**  **(−7.12, −0.74)** | −4.24  (−10.73, 2.26) | −3.93  (−9.61, 1.75) | −2.90  (−6.64, 0.85) | −2.64  (−5.83, 0.55) | −2.50  (−5.89, 0.89) | Dapagliflozin |

**g. League Table of any AE.**

| Sotagliflozin |  |  |  |  |  |  |  |  |  |
| --- | --- | --- | --- | --- | --- | --- | --- | --- | --- |
| 0.97  (0.89, 1.05) | PBO |  |  |  |  |  |  |  |  |
| 0.76  (0.37, 1.57) | 0.79  (0.39, 1.61) | Luseogliflozin |  |  |  |  |  |  |  |
| 0.97  (0.31, 3.04) | 1.00  (0.32, 3.13) | 1.27  (0.33, 4.87) | Liraglutide |  |  |  |  |  |  |
| 0.99  (0.91, 1.08) | 1.03  (0.99, 1.07) | 1.30  (0.64, 2.66) | 1.03  (0.33, 3.23) | Finerenone |  |  |  |  |  |
| **0.77**  **(0.63, 0.93)** | **0.79**  **(0.66, 0.95)** | 1.00  (0.48, 2.10) | 0.79  (0.25, 2.52) | **0.77**  **(0.64, 0.93)** | Exenatide |  |  |  |  |
| 0.93  (0.75, 1.16) | 0.96  (0.78, 1.19) | 1.22  (0.58, 2.57) | 0.96  (0.30, 3.08) | 0.94  (0.76, 1.16) | 1.21  (0.92, 1.60) | Ertugliflozin |  |  |  |
| 1.19  (0.91, 1.54) | 1.23  (0.96, 1.57) | 1.55  (0.73, 3.30) | 1.23  (0.38, 3.95) | 1.19  (0.93, 1.53) | **1.54**  **(1.14, 2.09)** | 1.27  (0.92, 1.76) | Empagliflozin |  |  |
| 1.06  (0.78, 1.45) | 1.10  (0.81, 1.49) | 1.39  (0.64, 3.02) | 1.10  (0.34, 3.58) | 1.07  (0.79, 1.45) | 1.38  (0.97, 1.97) | 1.14  (0.79, 1.64) | 0.90  (0.61, 1.32) | Dapagliflozin |  |
| **1.16**  **(1.04, 1.29)** | **1.20**  **(1.11, 1.29)** | 1.51  (0.74, 3.10) | 1.20  (0.38, 3.76) | 1**.16**  **(1.07, 1.26)** | **1.51**  **(1.24, 1.83)** | 1.24  (1.00, 1.55) | 0.98  (0.75, 1.26) | 1.09  (0.80, 1.49) | Canagliflozin |

**h. League Table of UTI.**

| Sotagliflozin |  |  |  |  |  |  |  |  |
| --- | --- | --- | --- | --- | --- | --- | --- | --- |
| 0.20  (0.01, 4.25) | Semaglutide |  |  |  |  |  |  |  |
| 1.04  (0.93, 1.17) | 5.35  (0.25, 116.29) | PBO |  |  |  |  |  |  |
| 0.27  (0.01, 5.41) | 1.40  (0.02, 101.84) | 0.26  (0.01, 5.17) | Luseogliflozin |  |  |  |  |  |
| 1.06  (0.92, 1.23) | 5.44  (0.25, 118.48) | 1.02  (0.93, 1.11) | 3.89  (0.20, 76.88) | Finerenone |  |  |  |  |
| 1.02  (0.82, 1.28) | 5.23  (0.24, 114.37) | 0.98  (0.80, 1.19) | 3.74  (0.19, 74.22) | 0.96  (0.78, 1.19) | Ertugliflozin |  |  |  |
| 1.05  (0.91, 1.20) | 5.36  (0.25, 116.67) | 1.00  (0.92, 1.09) | 3.83  (0.19, 75.70) | 0.98  (0.87, 1.11) | 1.03  (0.83, 1.27) | Empagliflozin |  |  |
| 1.12  (0.62, 2.02) | 5.72  (0.25, 131.35) | 1.07  (0.60, 1.92) | 4.09  (0.20, 85.38) | 1.05  (0.58, 1.90) | 1.09  (0.59, 2.02) | 1.07  (0.59, 1.93) | Dapagliflozin |  |
| 0.93  (0.80, 1.09) | 4.78  (0.22, 104.17) | **0.89**  **(0.80, 0.99)** | 3.42  (0.17, 67.59) | 0.88  (0.77, 1.01) | 0.91  (0.73, 1.14) | 0.89  (0.78, 1.02) | 0.84  (0.46, 1.51) | Canagliflozin |

**i. League Table of** **Hypoglycemia.**

| Luseogliflozin |  |  |  |  |  |  |  |  |  |  |  |
| --- | --- | --- | --- | --- | --- | --- | --- | --- | --- | --- | --- |
| 0.65  (0.12, 3.45) | Exenatide |  |  |  |  |  |  |  |  |  |  |
| 0.67  (0.12, 3.64) | 1.02  (0.63, 1.67) | Dapagliflozin |  |  |  |  |  |  |  |  |  |
| 0.94  (0.05, 18.16) | 0.62  (0.05, 7.32) | 0.63  (0.05, 7.65) | Semaglutide |  |  |  |  |  |  |  |  |
| 0.60  (0.12, 3.12) | 1.09  (0.82, 1.43) | 1.11  (0.73, 1.70) | 0.57  (0.05, 6.66) | Finerenone |  |  |  |  |  |  |  |
| 0.58  (0.11, 2.99) | 0.88  (0.67, 1.16) | 1.16  (0.76, 1.78) | 0.55  (0.05, 6.39) | 0.96  (0.83, 1.11) | Empagliflozin |  |  |  |  |  |  |
| 0.55  (0.11, 2.84) | 0.84  (0.62, 1.13) | 0.82  (0.53, 1.26) | 0.52  (0.04, 6.06) | 0.91  (0.76, 1.08) | 0.95  (0.79, 1.14) | Canagliflozin |  |  |  |  |  |
| 0.54  (0.10, 2.83) | 0.83  (0.61, 1.13) | 1.23  (0.79, 1.92) | 0.51  (0.04, 6.03) | 0.90  (0.75, 1.09) | 1.06  (0.87, 1.29) | 1.01  (0.81, 1.25) | Ertugliflozin |  |  |  |  |
| 0.50  (0.09, 2.81) | 0.77  (0.43, 1.38) | 0.75  (0.38, 1.46) | 0.47  (0.04, 5.86) | 0.83  (0.49, 1.42) | 0.87  (0.51, 1.49) | 0.92  (0.53, 1.58) | 0.92  (0.53, 1.60) | Bexagliflozin |  |  |  |
| 1.94  (0.37, 10.06) | 1.27  (0.94, 1.70) | 1.30  (0.84, 2.00) | 2.05  (0.17, 24.05) | 1.17  (0.99, 1.38) | 1.12  (0.93, 1.34) | 1.06  (0.87, 1.29) | 1.05  (0.86, 1.30) | 0.97  (0.57, 1.67) | Sotagliflozin |  |  |
| 1.96  (0.38, 10.11) | 1.28  (0.99, 1.66) | 1.31  (0.87, 1.98) | 0.48  (0.04, 5.64) | **1.18**  **(1.07, 1.31)** | **1.13**  **(1.01, 1.27)** | 1.07  (0.93, 1.24) | 1.06  (0.91, 1.25) | 0.98  (0.58, 1.66) | 0.99  (0.87, 1.13) | PBO |  |
| 0.32  (0.04, 2.32) | 2.05  (0.65, 6.40) | 2.09  (0.64, 6.85) | 0.30  (0.02, 4.49) | 1.88  (0.62, 5.75) | 1.80  (0.59, 5.51) | 1.71  (0.56, 5.24) | 1.70  (0.55, 5.22) | 1.57  (0.46, 5.37) | 0.62  (0.20, 1.90) | 0.63  (0.21, 1.90) | Liraglutide |

**j. League Table of** **AKI.**

| PBO |  |  |  |  |  |
| --- | --- | --- | --- | --- | --- |
| 1.06 (0.97, 1.17) | Finerenone |  |  |  |  |
| 0.81 (0.08, 7.81) | 0.76 (0.08, 7.35) | Exenatide |  |  |  |
| 1.20 (0.87, 1.65) | 1.12 (0.81, 1.57) | 1.48 (0.15, 14.57) | Ertugliflozin |  |  |
| 1.14 (0.97, 1.35) | 1.07 (0.88, 1.30) | 1.41 (0.15, 13.70) | 0.96 (0.67, 1.37) | Canagliflozin |  |
| 0.75 (0.25, 2.21) | 0.70 (0.24, 2.09) | 0.93 (0.08, 11.41) | 0.63 (0.20, 1.94) | 0.66 (0.22, 1.96) | Bexagliflozin |

**The value in each cell represents the impact of the row treatment regimen on the column treatment regimen. Categorical variables were assessed using odds ratio (OR), while continuous variables were evaluated using mean difference (MD). Statistical results were determined based on a 95% confidence interval (95%CI). Results were considered statistically significant if the 95%CI did not include null values (0 for MD and 1 for OR).**

**Appendix 7 HbA1c Outcome: Results.**

**a. HbA1c Outcome: Network forest.**

**
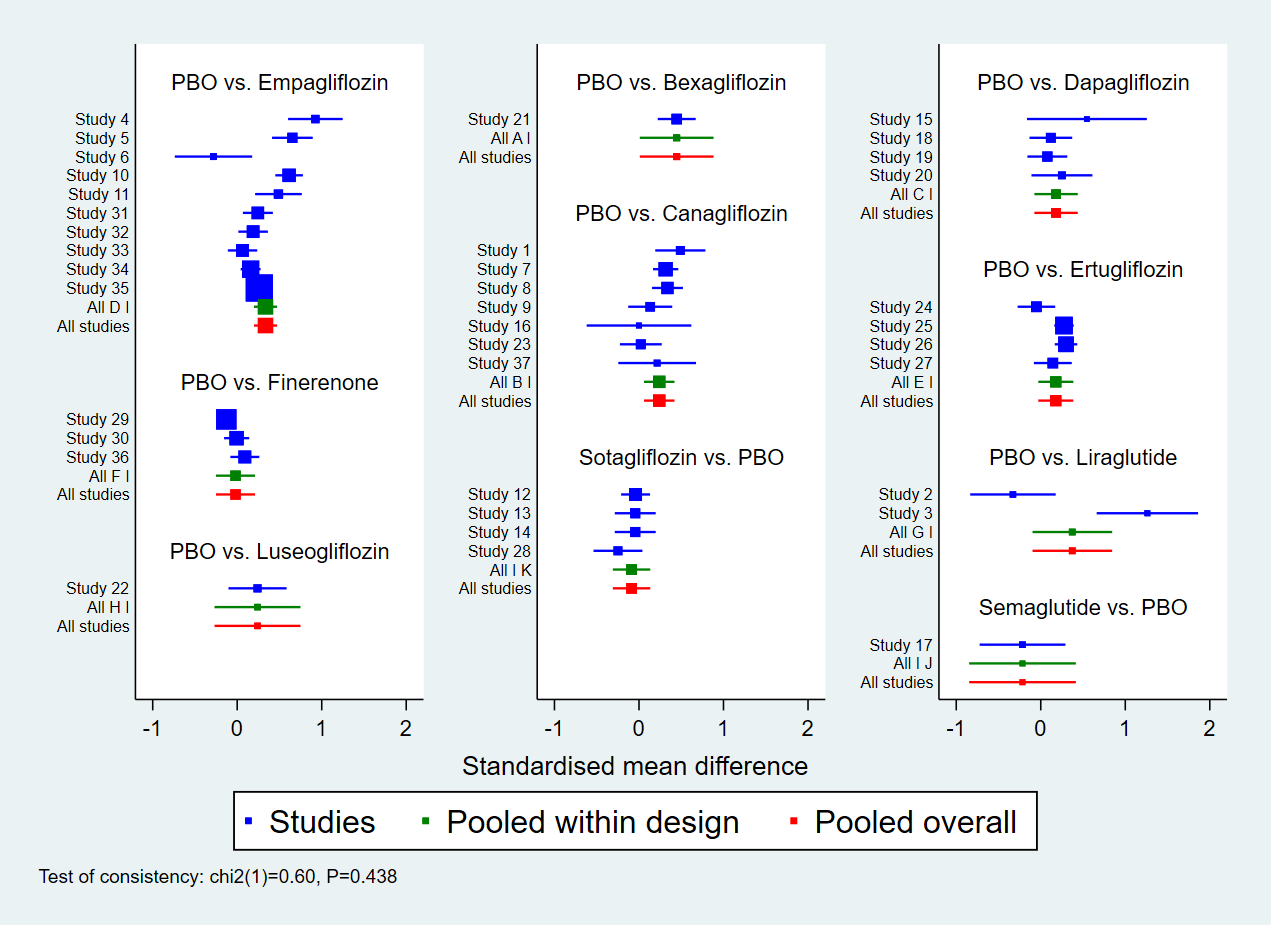
**

**b. HbA1c Outcome: Surface under the cumulative ranking curves (SUCRAs) plots**


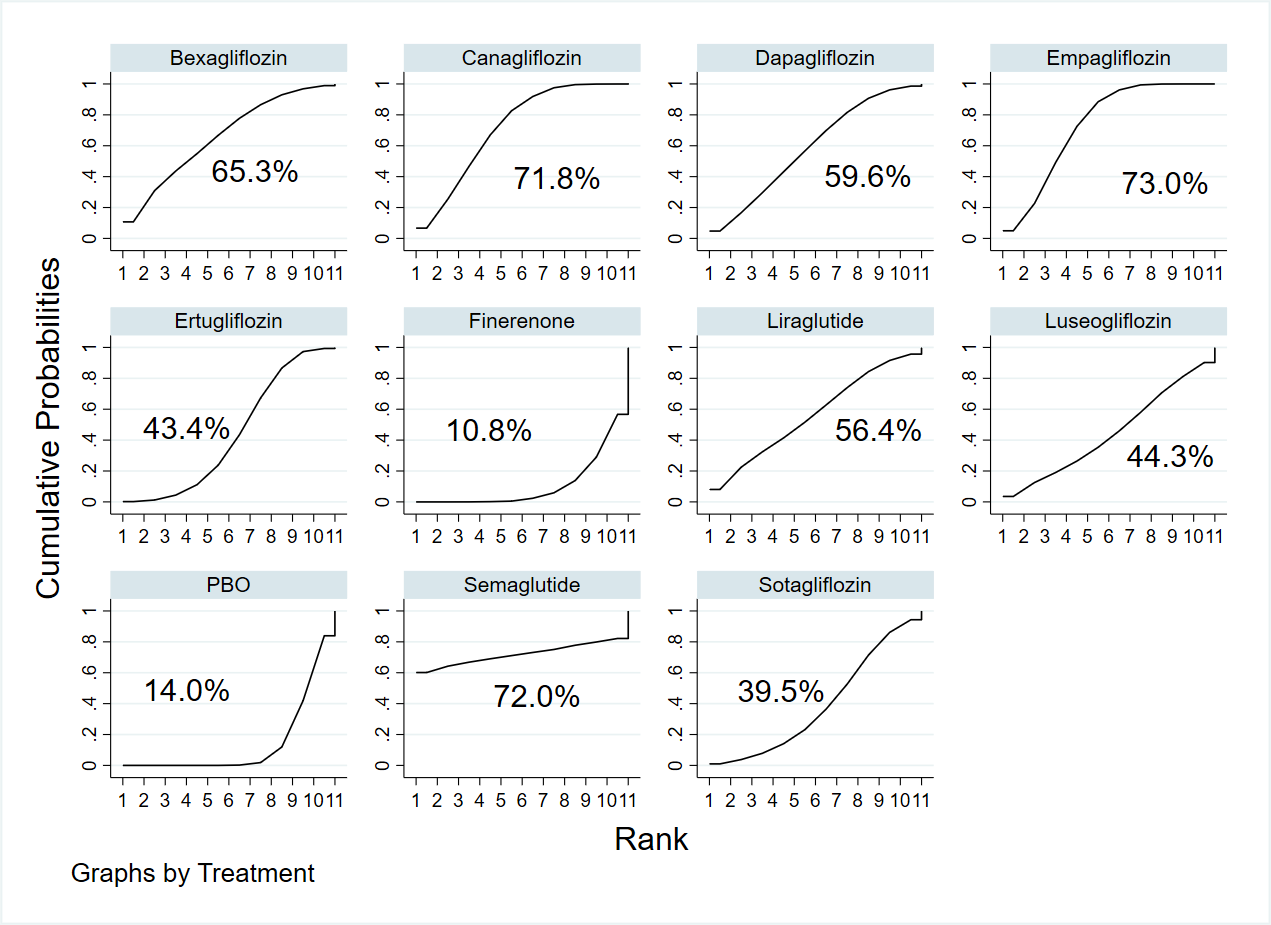


The cumulative probability curve for the HbA1c treatment network illustrates the estimated likelihood of each treatment. A higher SUCRA probability indicates a greater likelihood of being the optimal treatment.

**Appendix 8 eGFR Outcome: Results**

**a. eGFR Outcome: Network forest.**

**
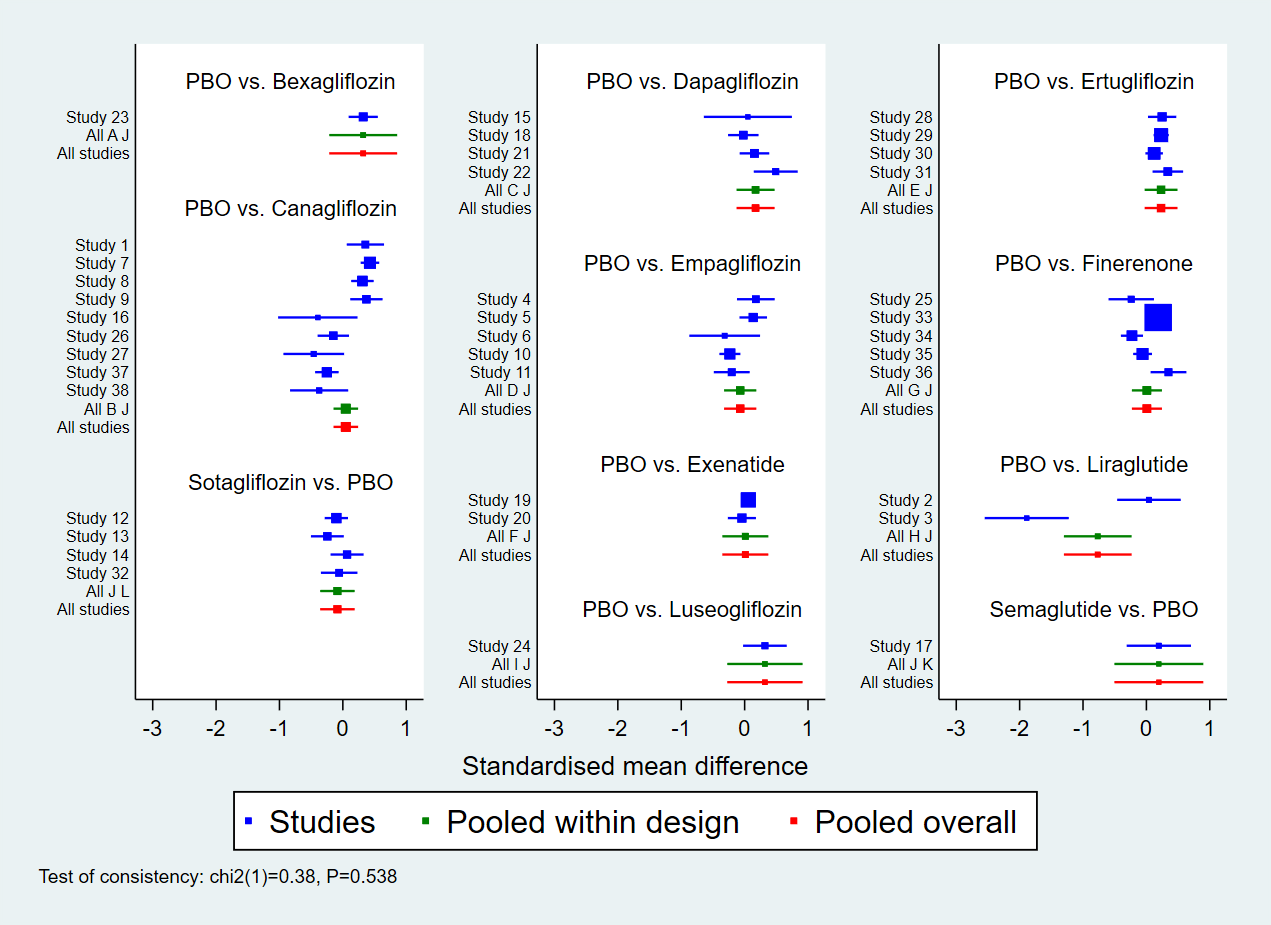
**

**b. eGFR Outcome: Surface under the cumulative ranking curves (SUCRAs) plots**


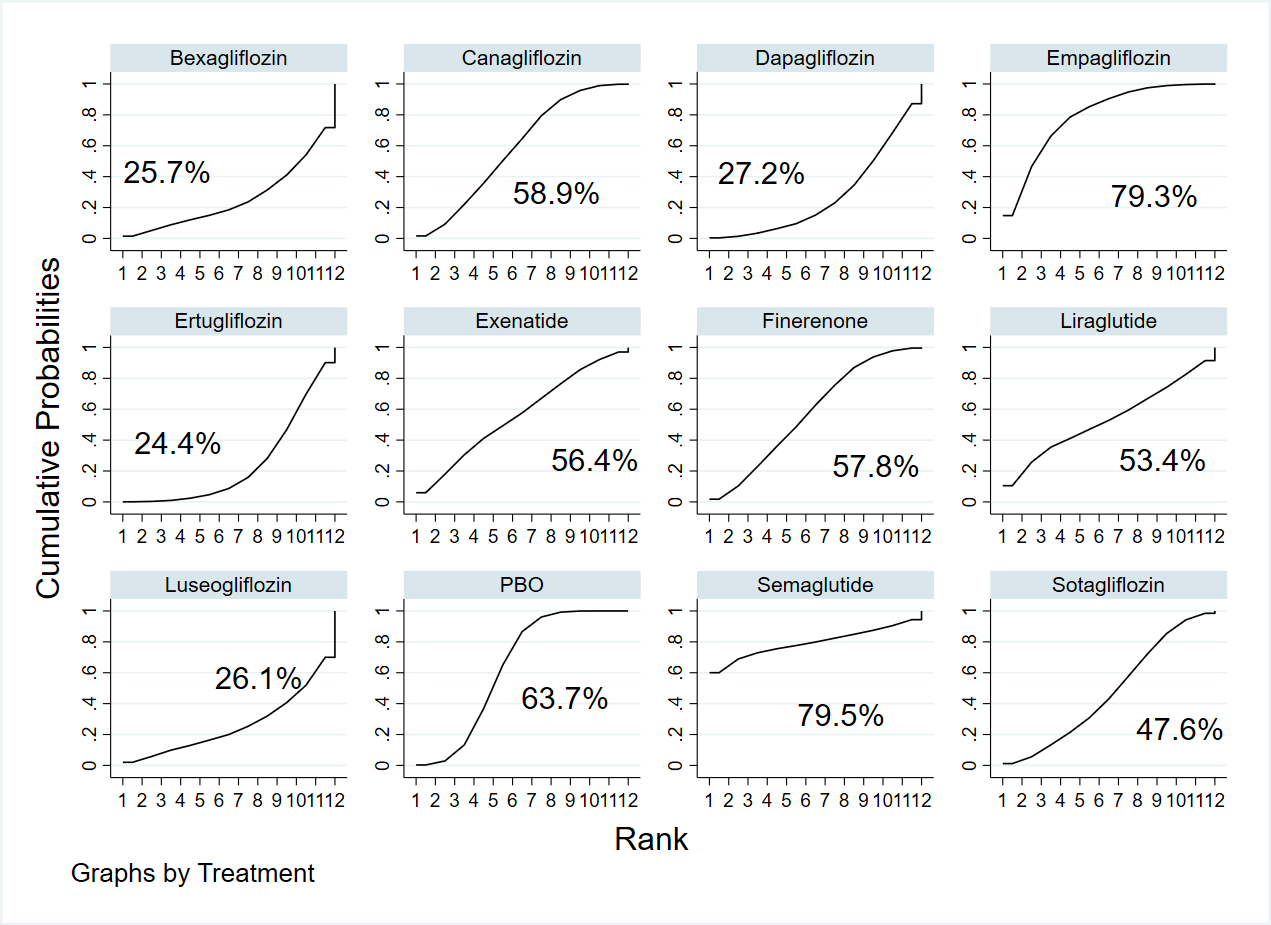


The cumulative probability curve for the eGFR treatment network illustrates the estimated likelihood of each treatment. A higher SUCRA probability indicates a greater likelihood of being the optimal treatment.

**Appendix 9 LDL-C Outcome: Results**

**a. LDL-C Outcome: Network forest.**

**
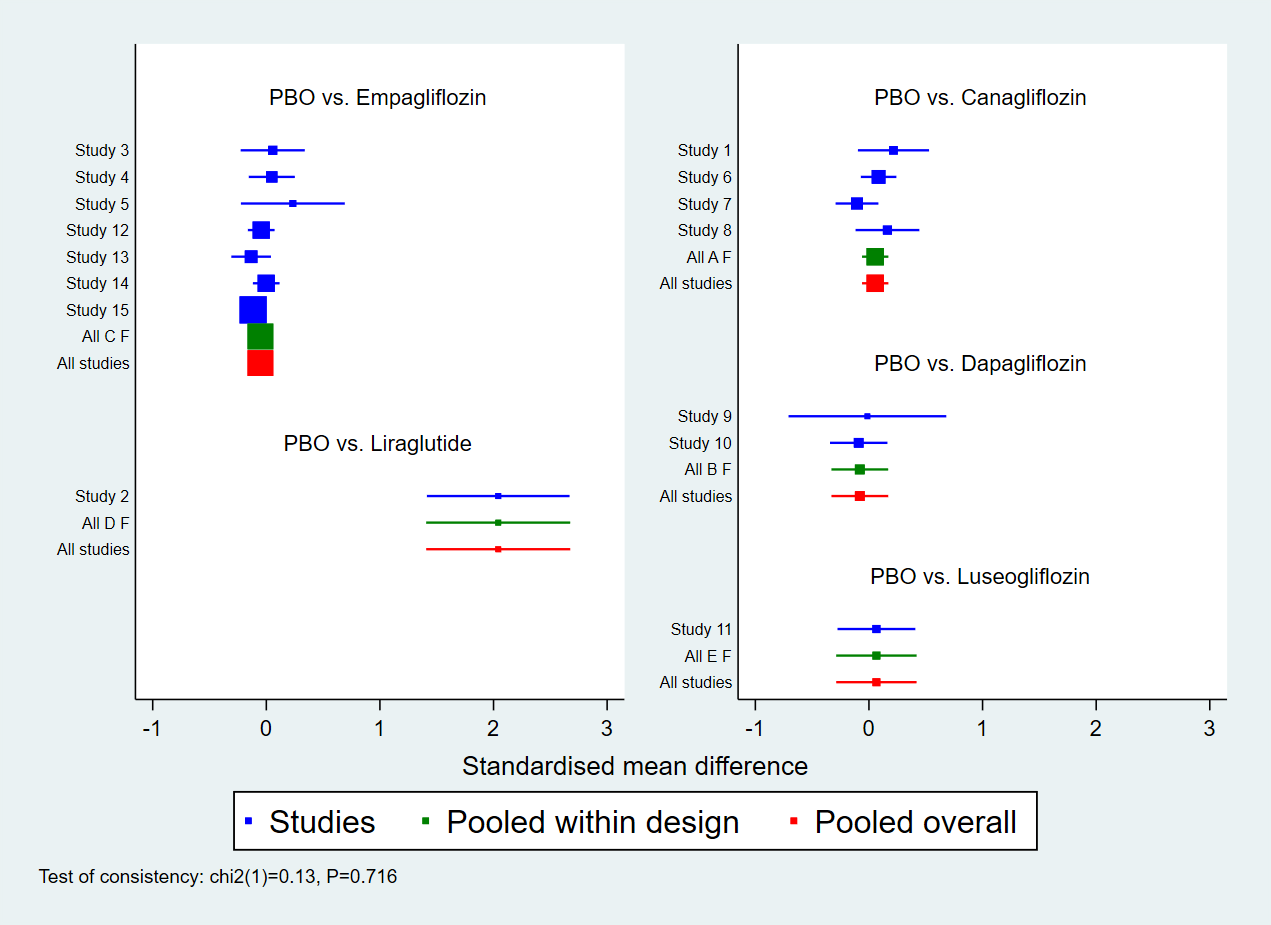
**

**b. LDL-C Outcome: Surface under the cumulative ranking curves (SUCRAs) plots**


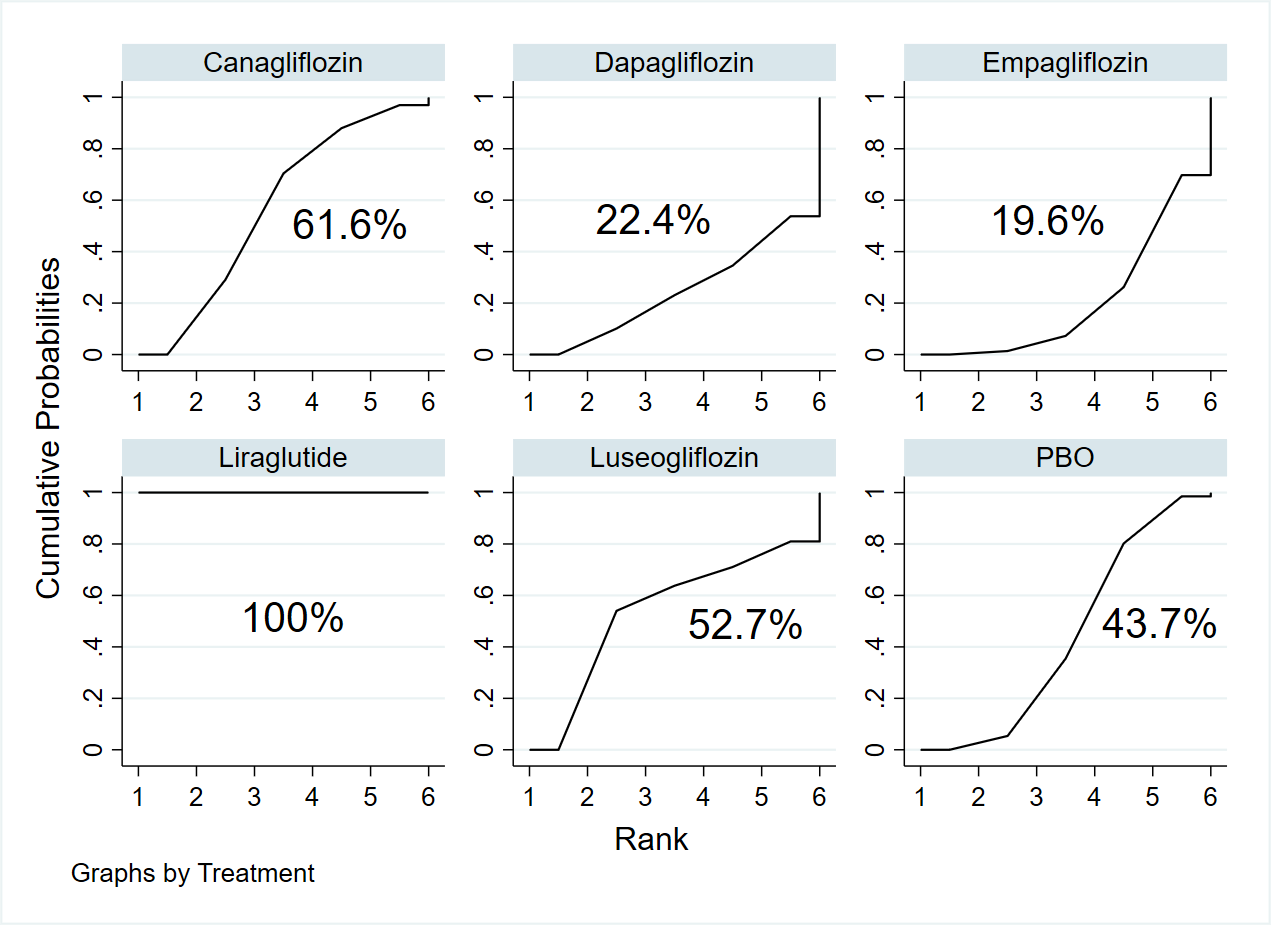


The cumulative probability curve for the LDL-C treatment network illustrates the estimated likelihood of each treatment. A higher SUCRA probability indicates a greater likelihood of being the optimal treatment.

**Appendix 10 SBP Outcome: Results.**

**a. SBP Outcome: Network forest.**

**
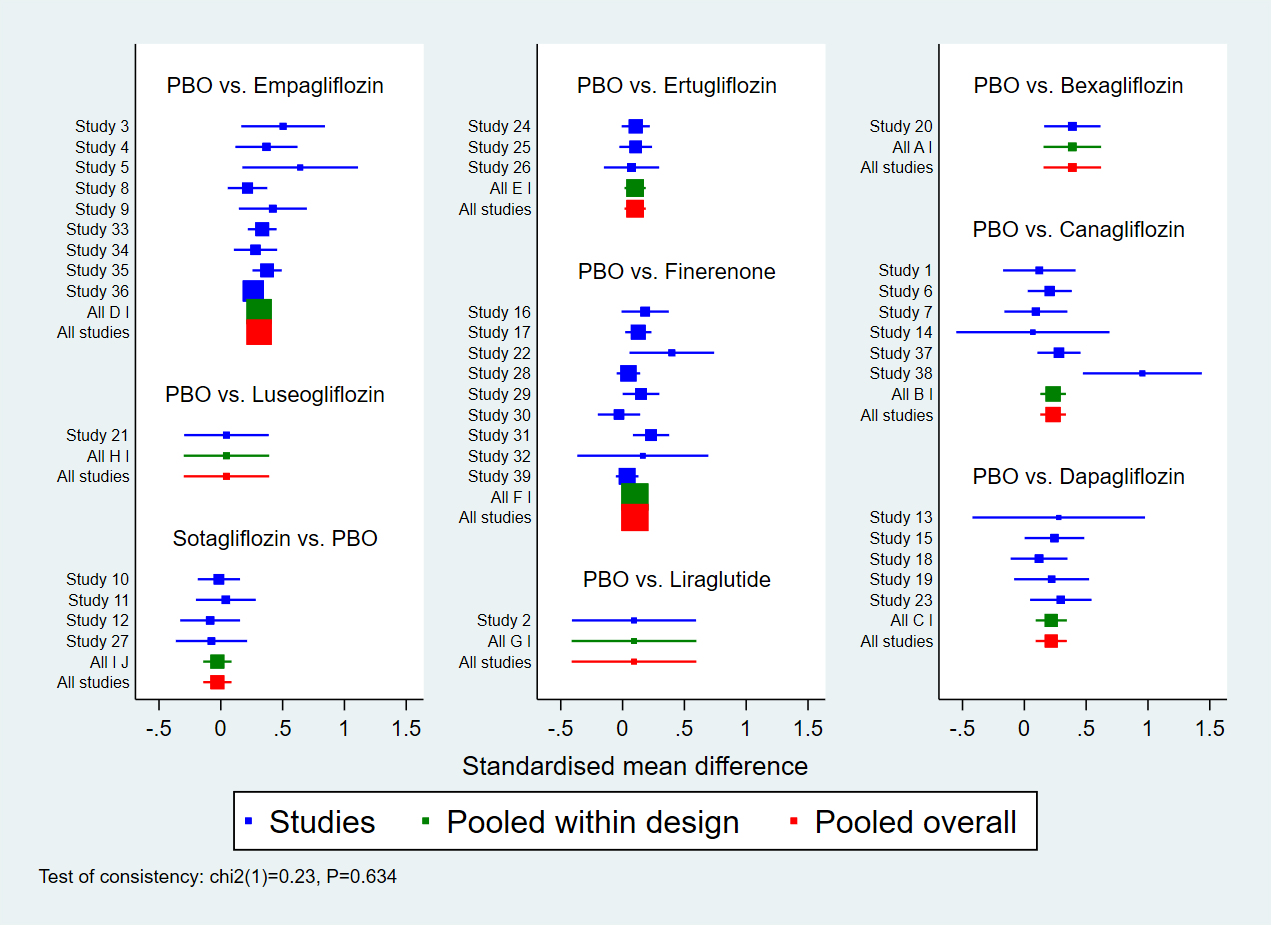
**

**b. SBP Outcome: Surface under the cumulative ranking curves (SUCRAs) plots**


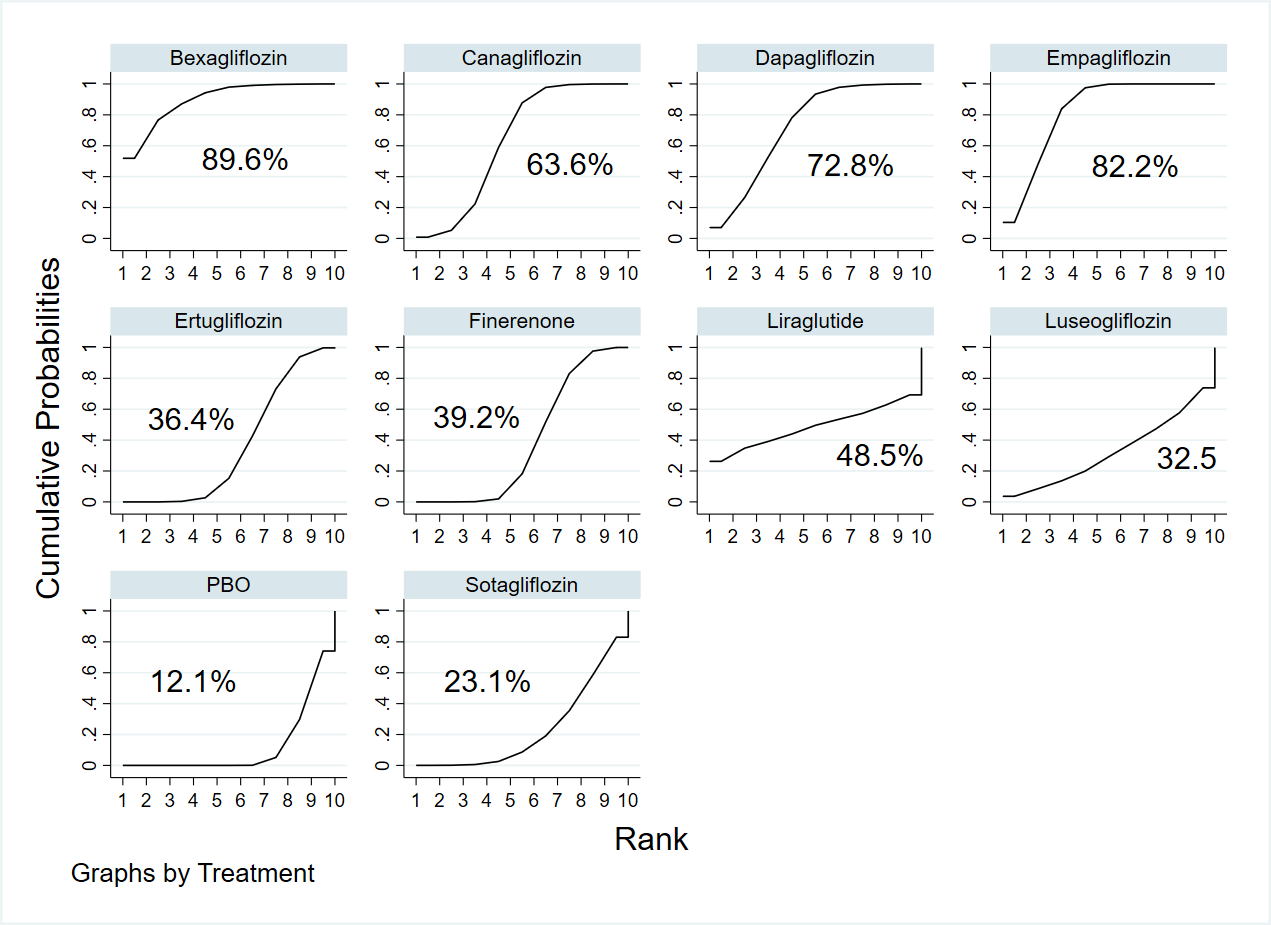


The cumulative probability curve for the SBP treatment network illustrates the estimated likelihood of each treatment. A higher SUCRA probability indicates a greater likelihood of being the optimal treatment.

**Appendix 11 DBP Outcome: Results.**

**a. DBP Outcome: Network forest.**

**
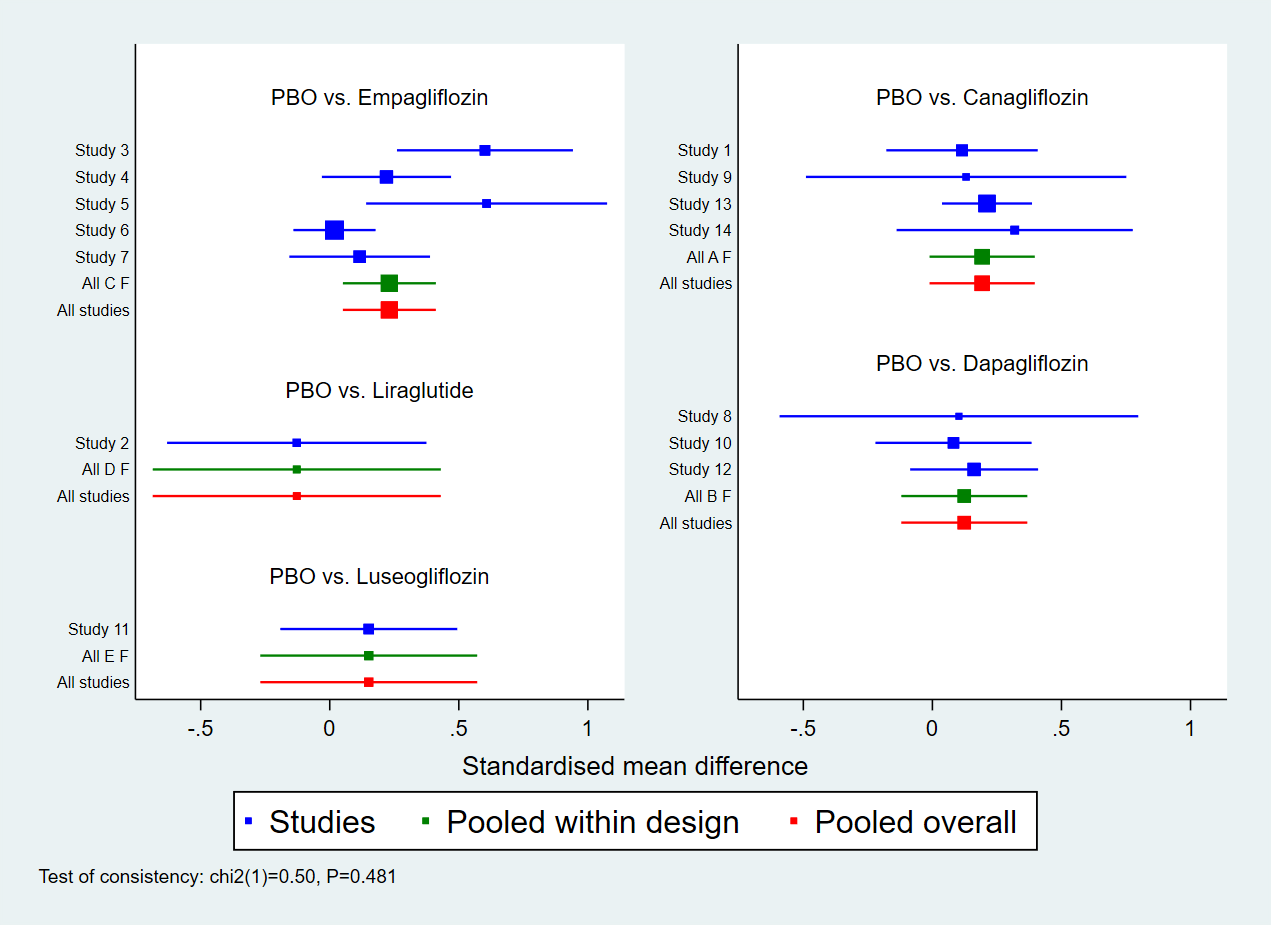
**

**b. DBP Outcome: Surface under the cumulative ranking curves (SUCRAs) plots**


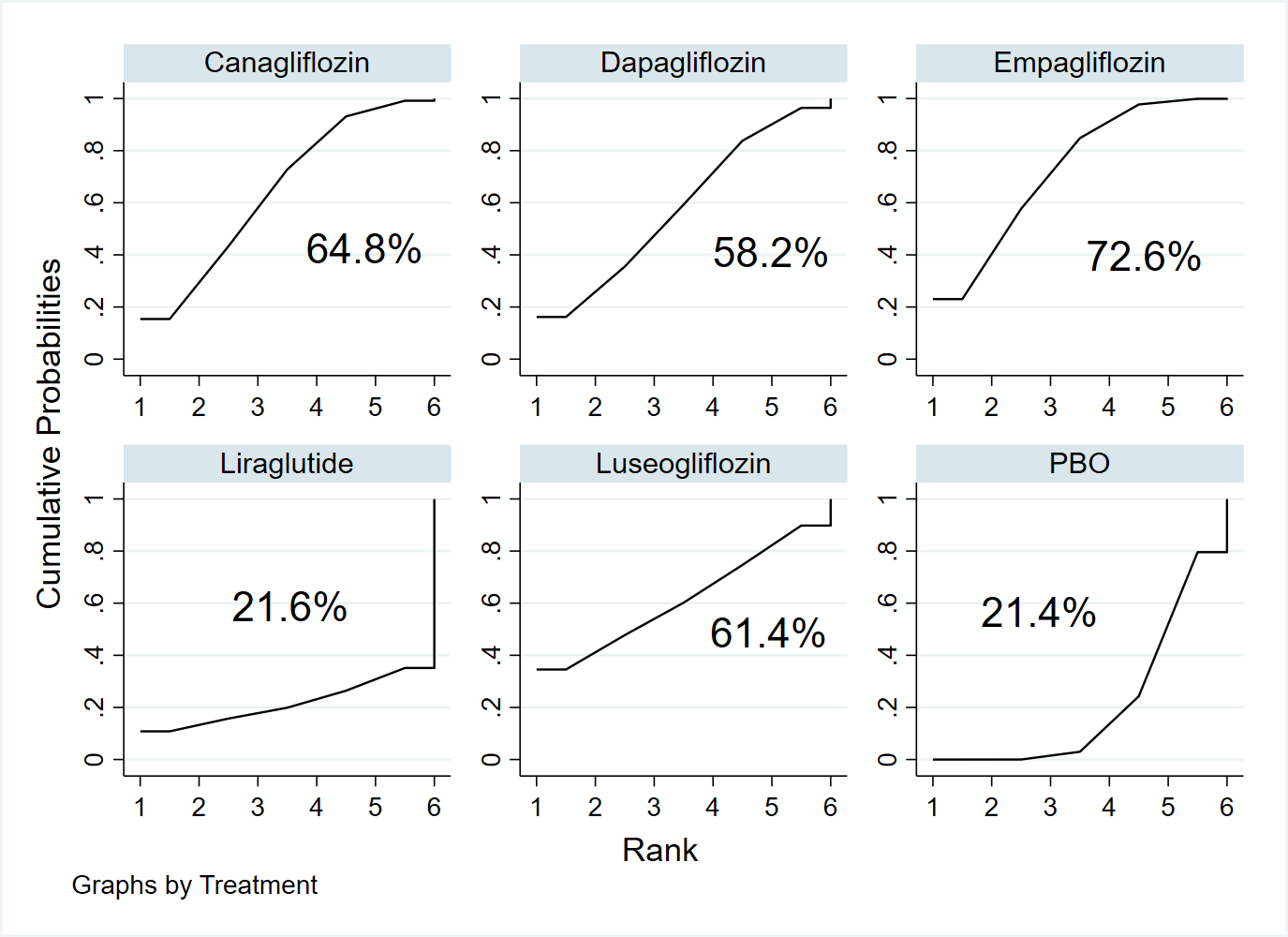


The cumulative probability curve for the DBP treatment network illustrates the estimated likelihood of each treatment. A higher SUCRA probability indicates a greater likelihood of being the optimal treatment.

**Appendix 12 Body Weight Outcome: Results.**

**a. Body Weight Outcome: Network forest.**

**
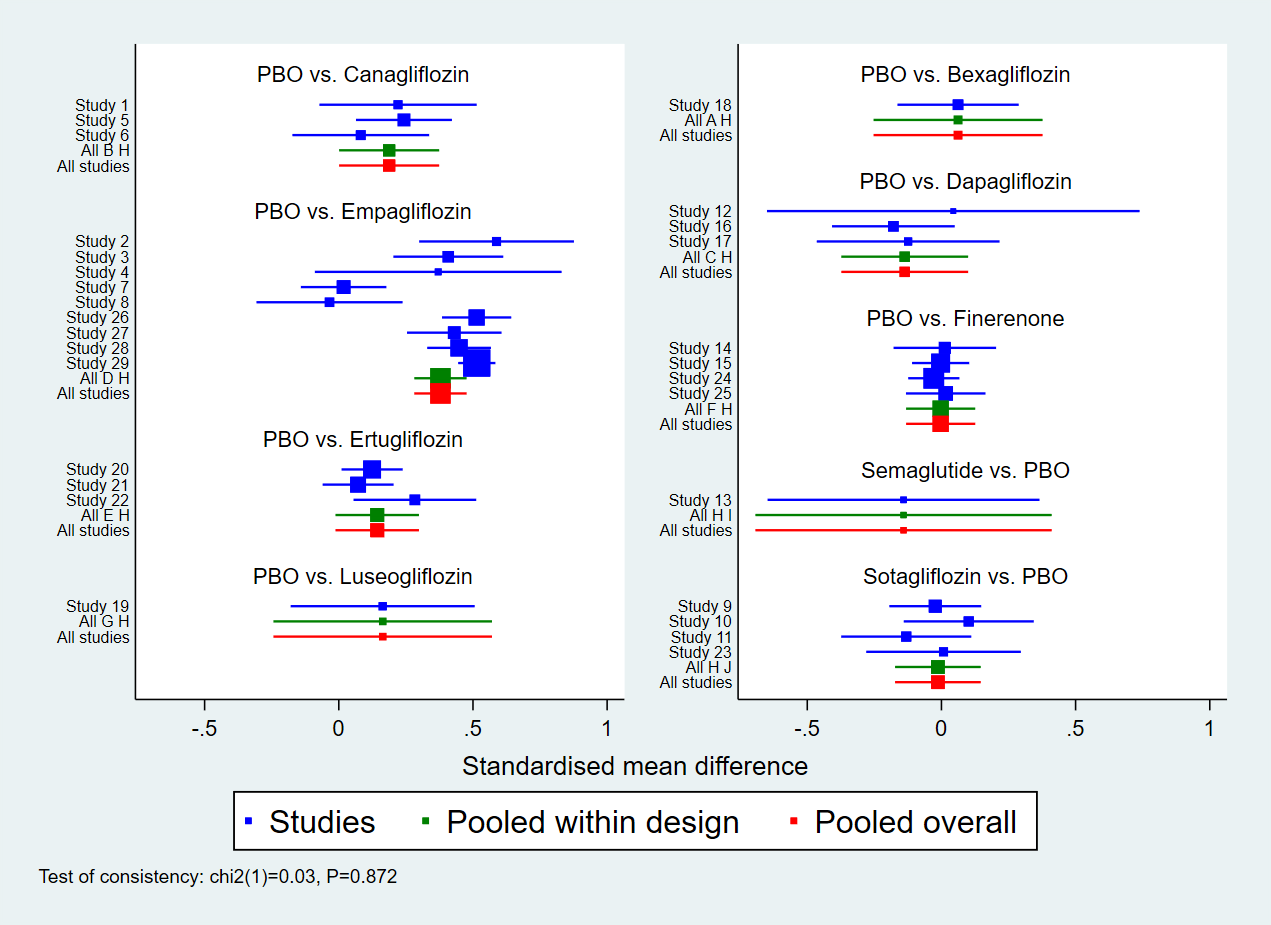
**

**b. Body Weight Outcome: Surface under the cumulative ranking curves (SUCRAs) plots**


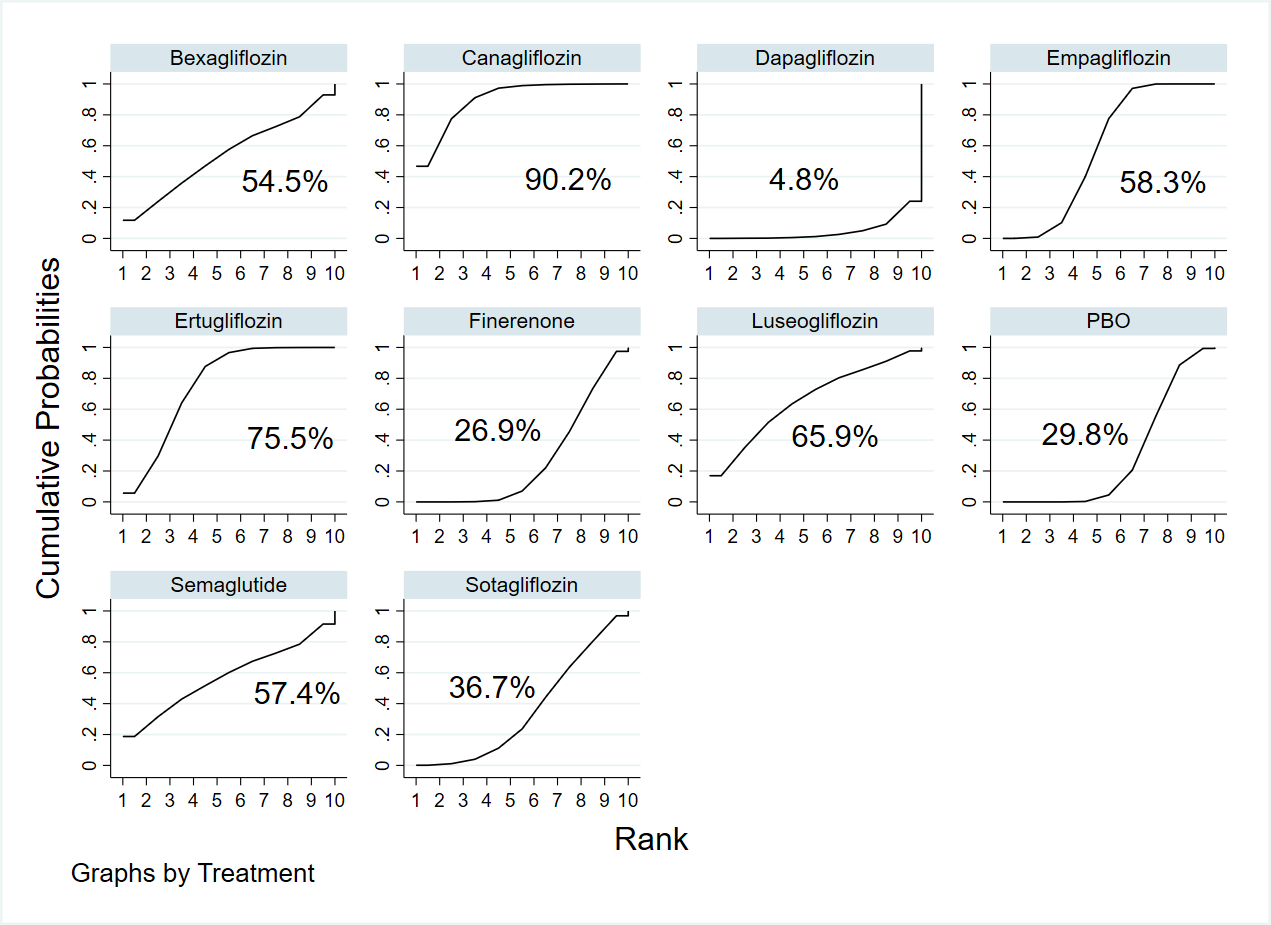


The cumulative probability curve for the Body Weight treatment network illustrates the estimated likelihood of each treatment. A higher SUCRA probability indicates a greater likelihood of being the optimal treatment.

**Appendix 13 Any AE Outcome: Results.**

**a. Any AE Outcome: Network forest.**

**
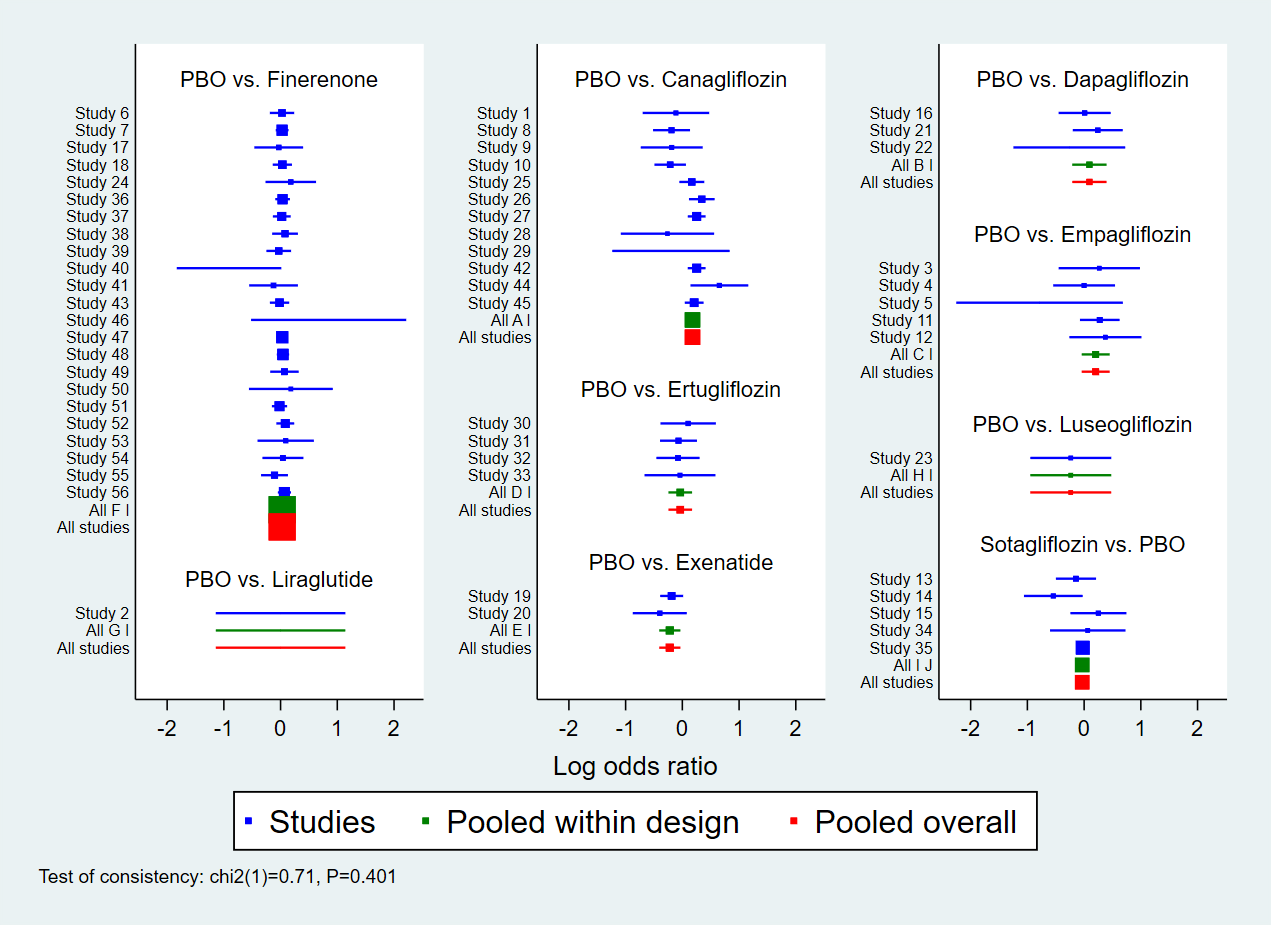
**

**b. Any AE Outcome: Surface under the cumulative ranking curves (SUCRAs) plots**


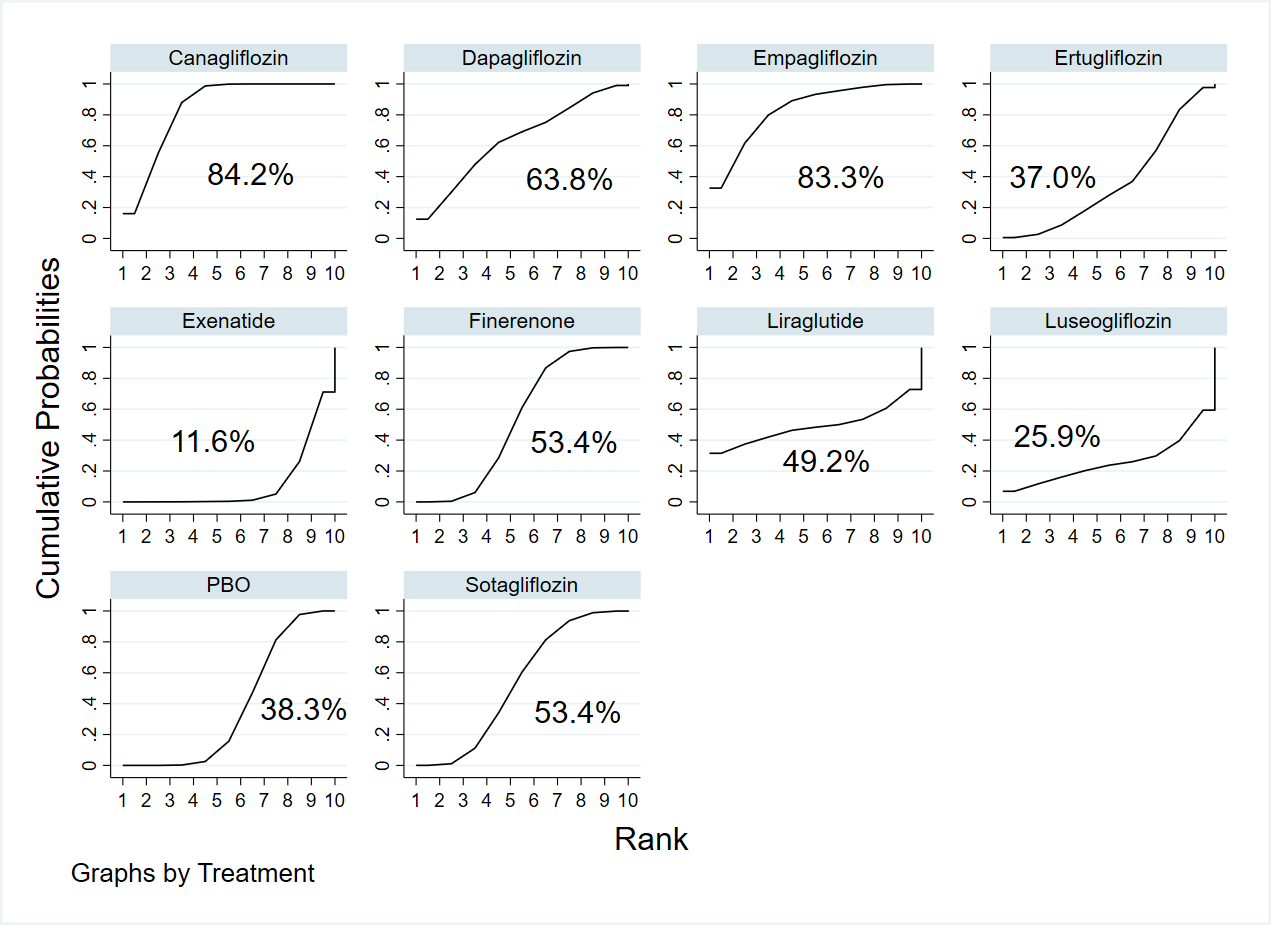


The cumulative probability curve for the any AE treatment network illustrates the estimated likelihood of each treatment. A higher SUCRA probability indicates a greater likelihood of being the optimal treatment.

**Appendix 14 UTI Outcome: Results**

**a. UTI Outcome: Network forest.**

**
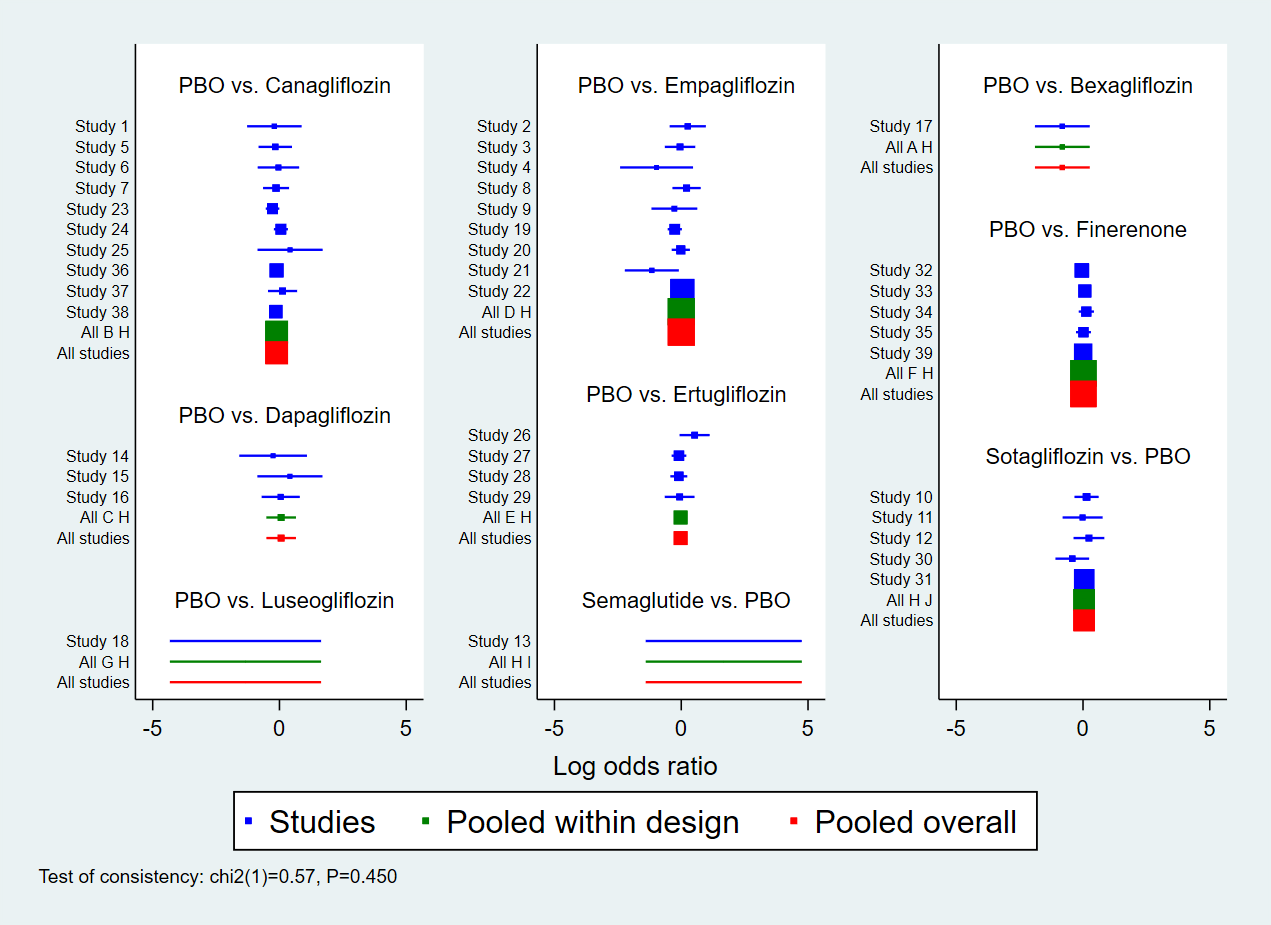
**

**b. UTI Outcome: Surface under the cumulative ranking curves (SUCRAs) plots**


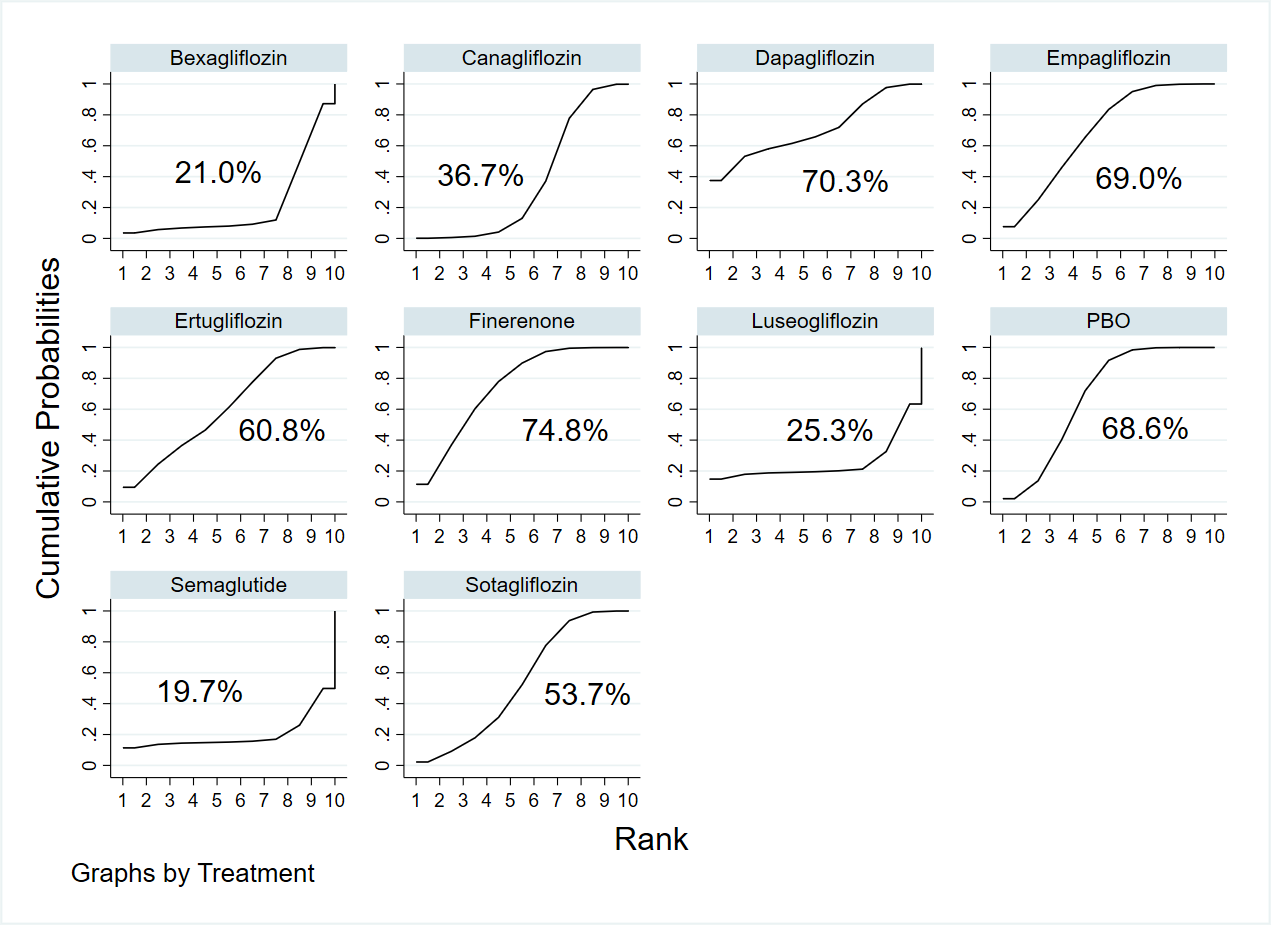


The cumulative probability curve for the UTI treatment network illustrates the estimated likelihood of each treatment. A higher SUCRA probability indicates a greater likelihood of being the optimal treatment.

**Appendix 15 Hypoglycemia Outcome: Results**

**a. Hypoglycemia Outcome: Network forest.**

**
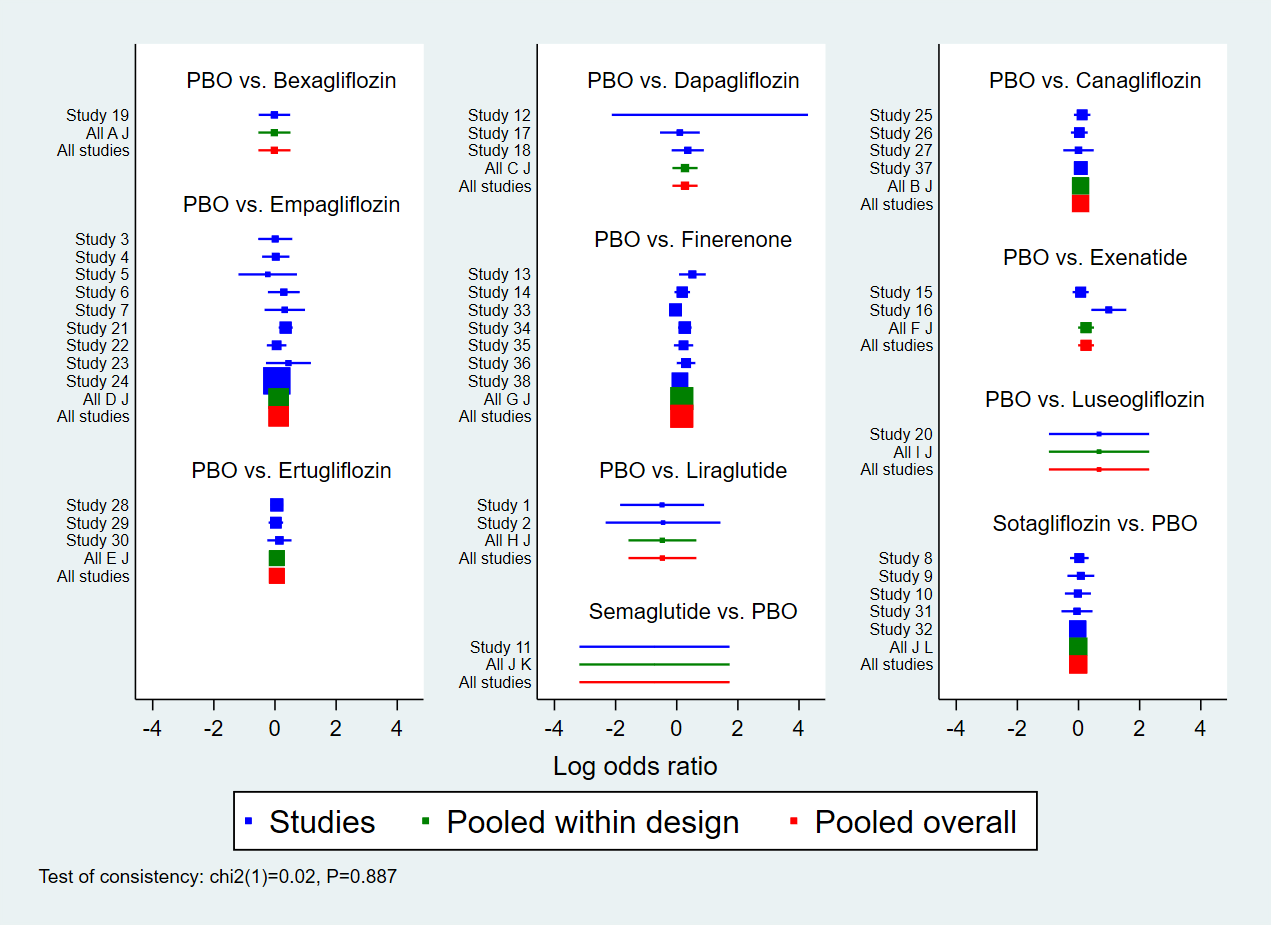
**

**b. Hypoglycemia Outcome: Surface under the cumulative ranking curves (SUCRAs) plots**


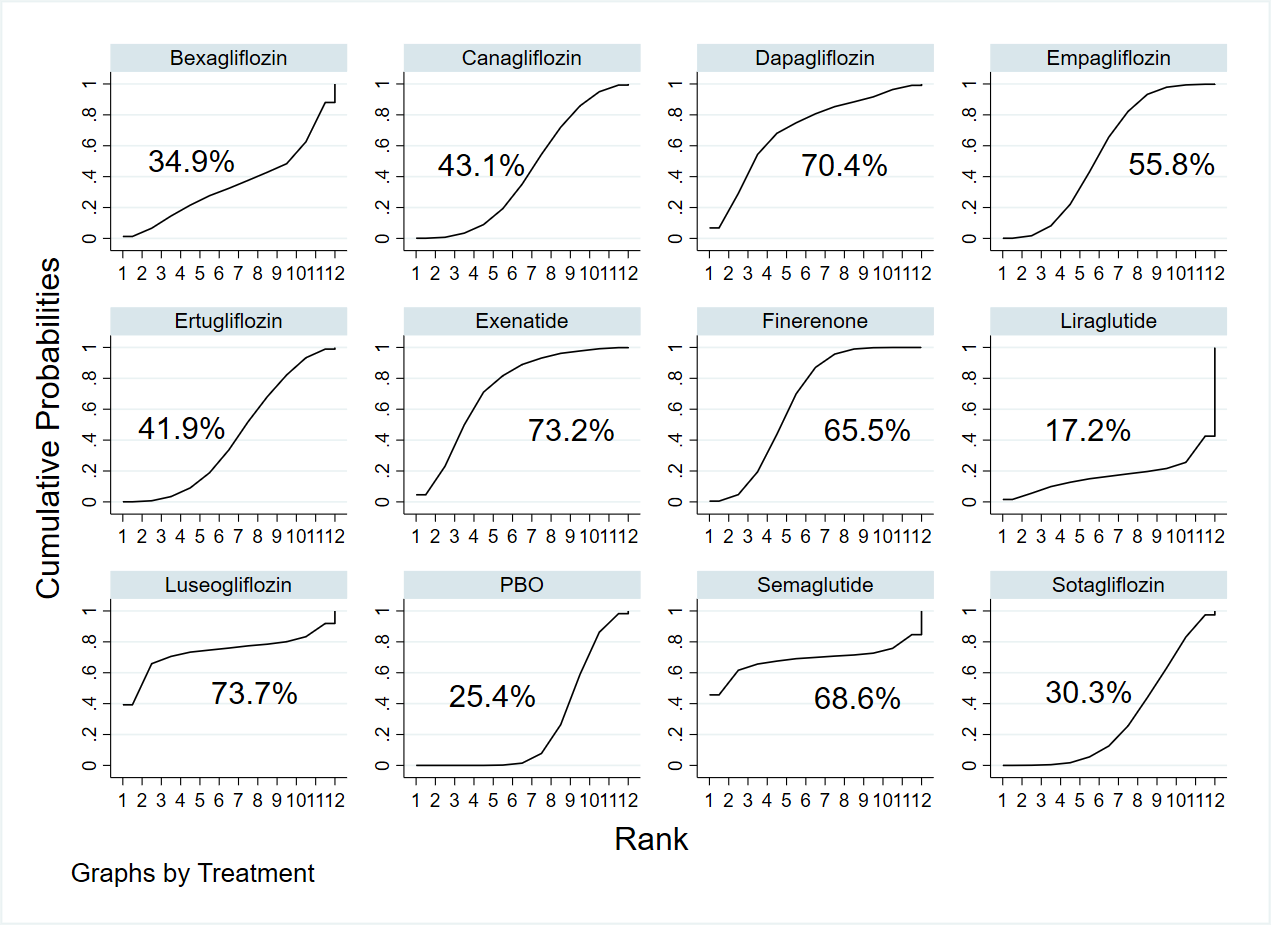


The cumulative probability curve for the Hypoglycemia treatment network illustrates the estimated likelihood of each treatment. A higher SUCRA probability indicates a greater likelihood of being the optimal treatment.

**Appendix 16 AKI Outcome: Results**

**a. AKI Outcome: Network forest.**

**
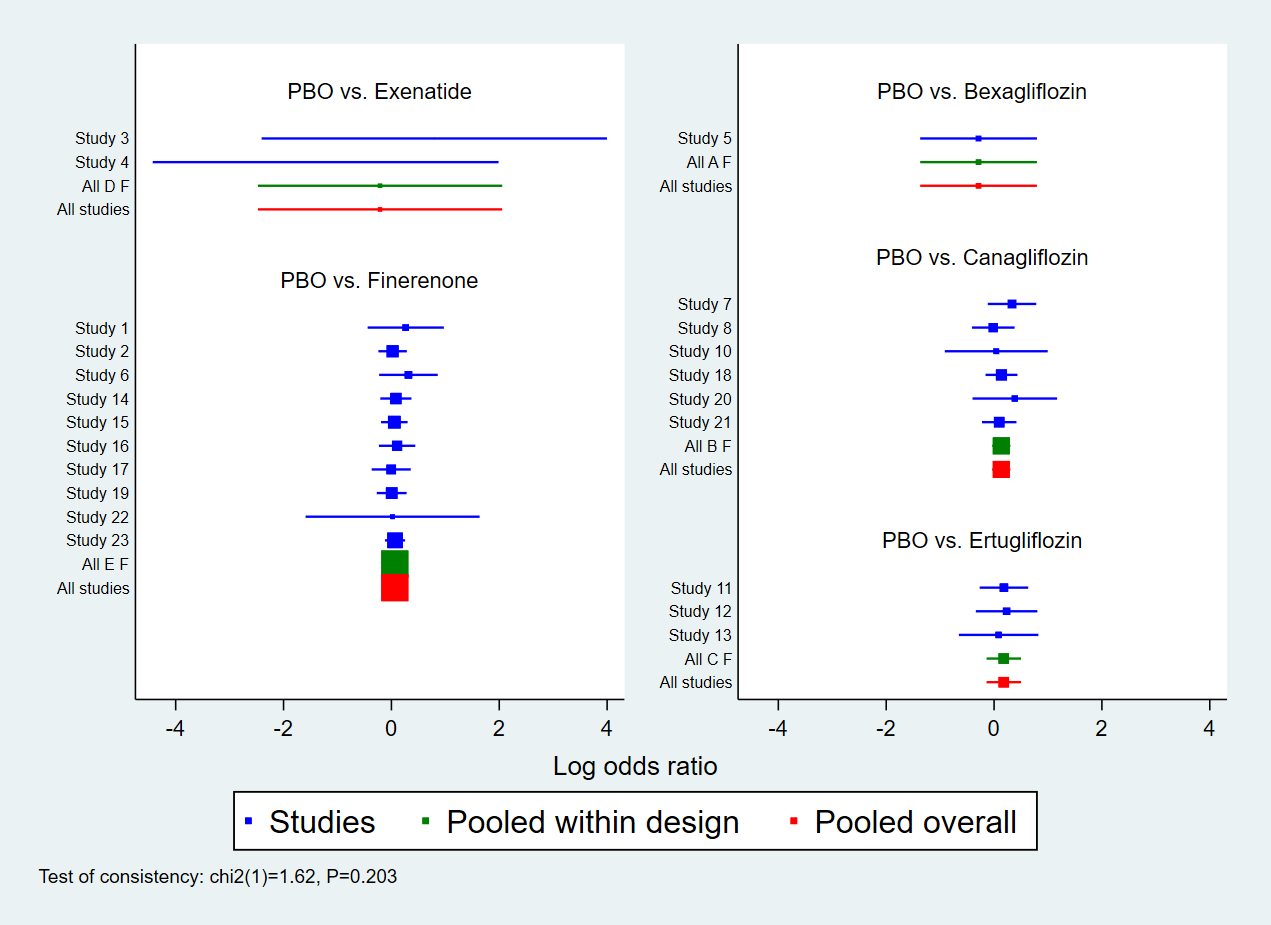
**

**b. AKI Outcome: Surface under the cumulative ranking curves (SUCRAs) plots**


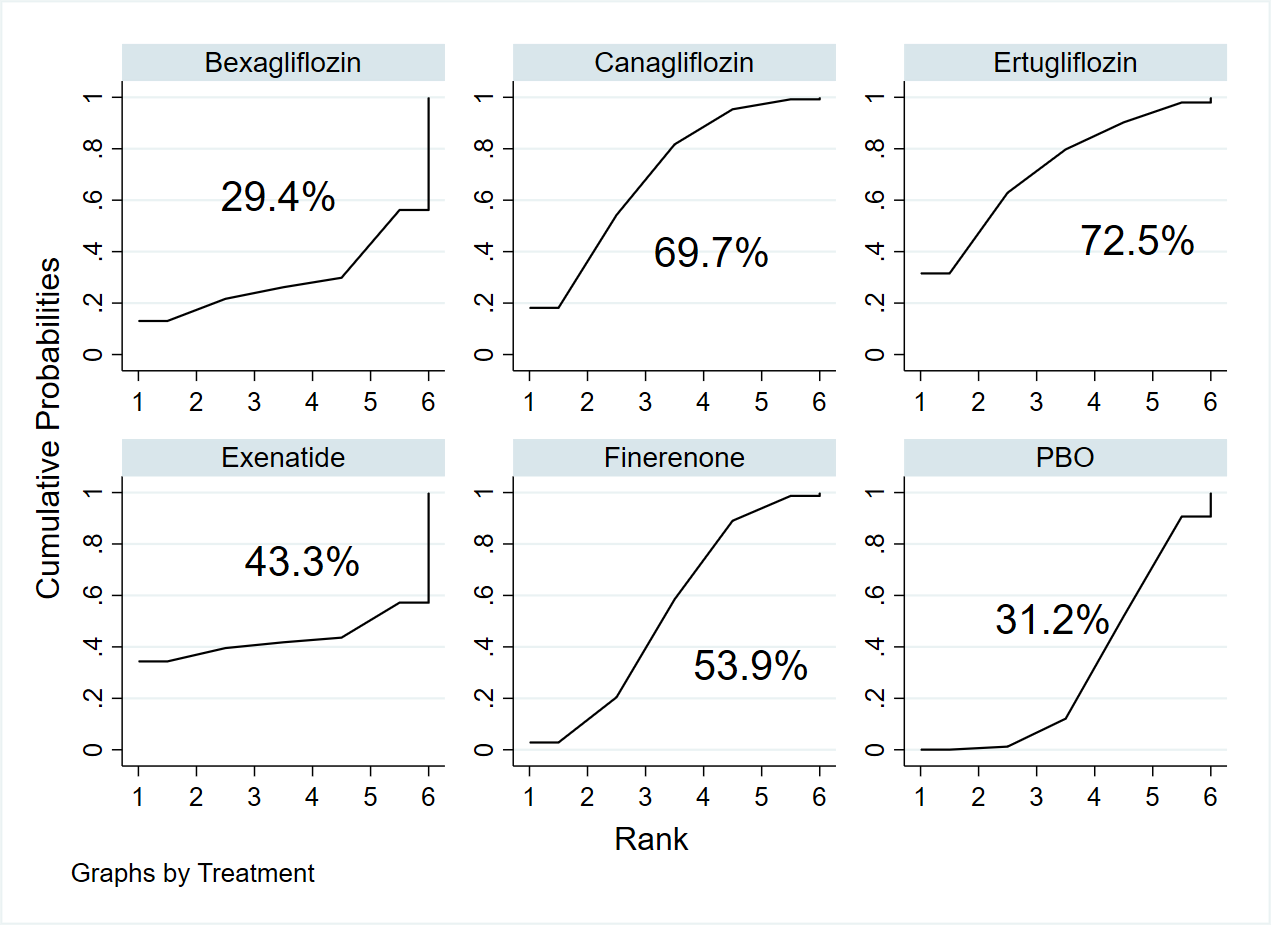


The cumulative probability curve for the AKI treatment network illustrates the estimated likelihood of each treatment. A higher SUCRA probability indicates a greater likelihood of being the optimal treatment.

**Appendix 17 Funnel plots of each outcome indicator**

**a. HbA1c outcome: comparison adjusted funnel plot**


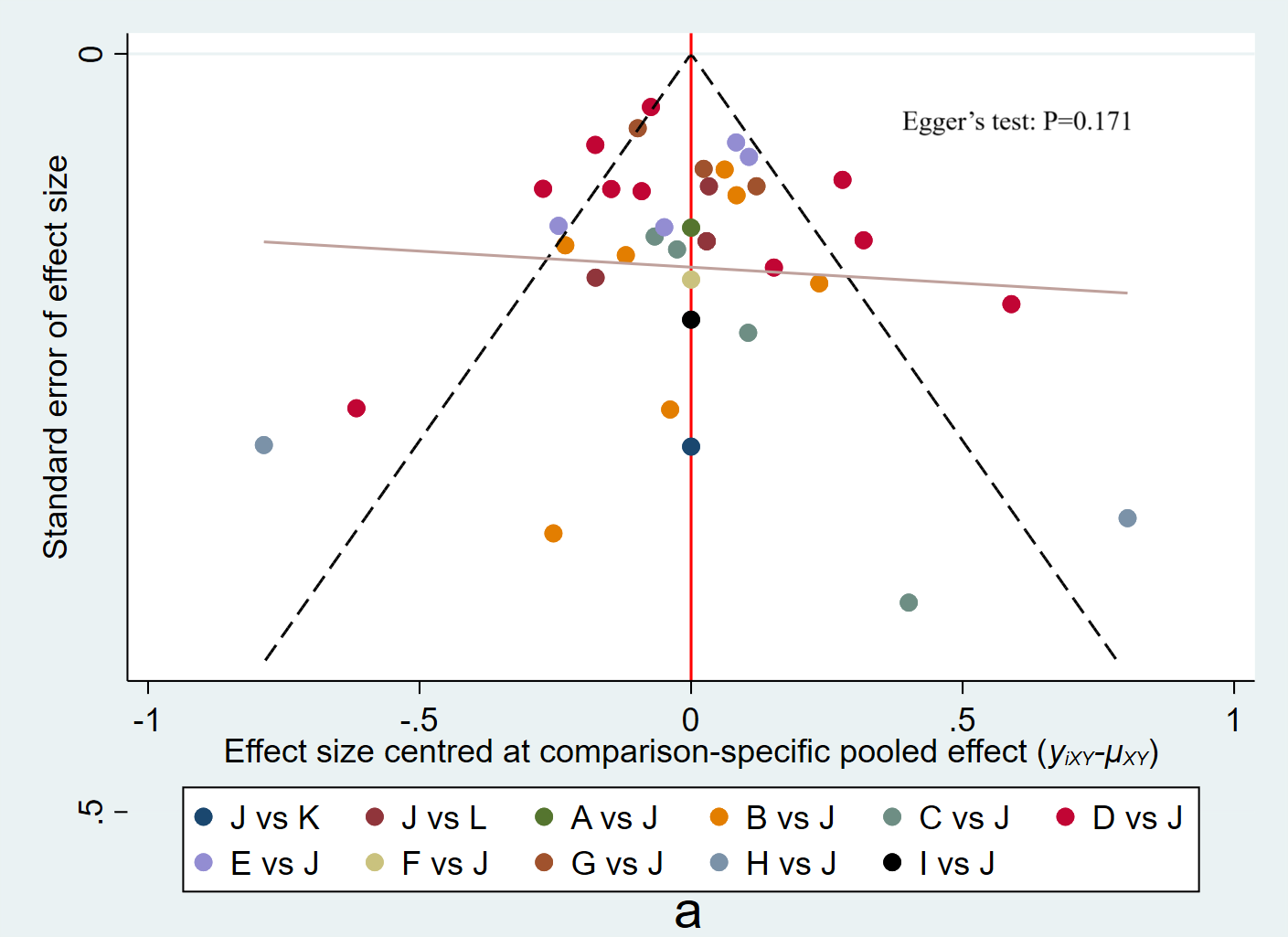


**b. eGFR outcome: comparison adjusted funnel plot**


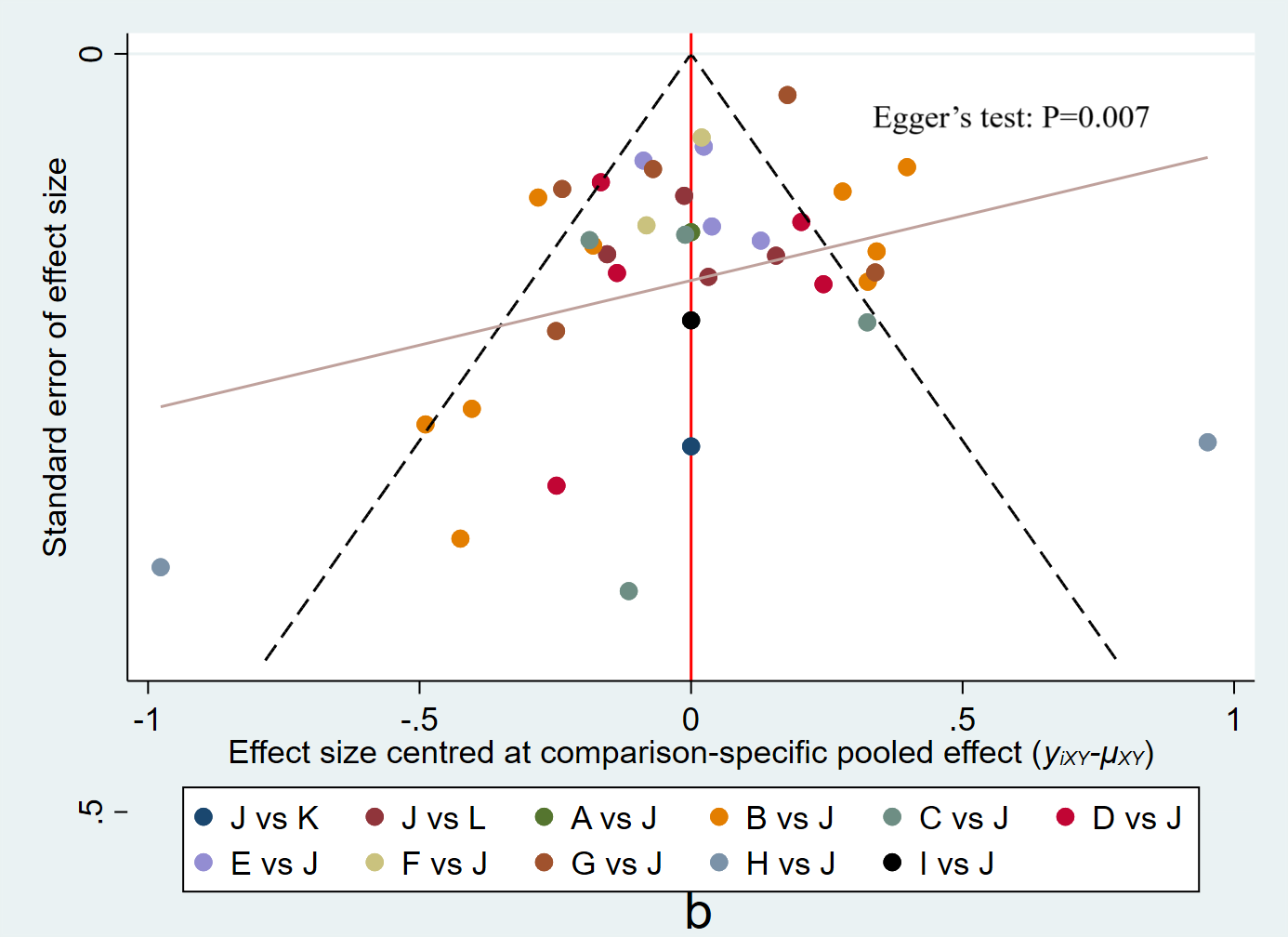


**c. LDL−C outcome: comparison adjusted funnel plot** **Egger’s test: P=0.096**


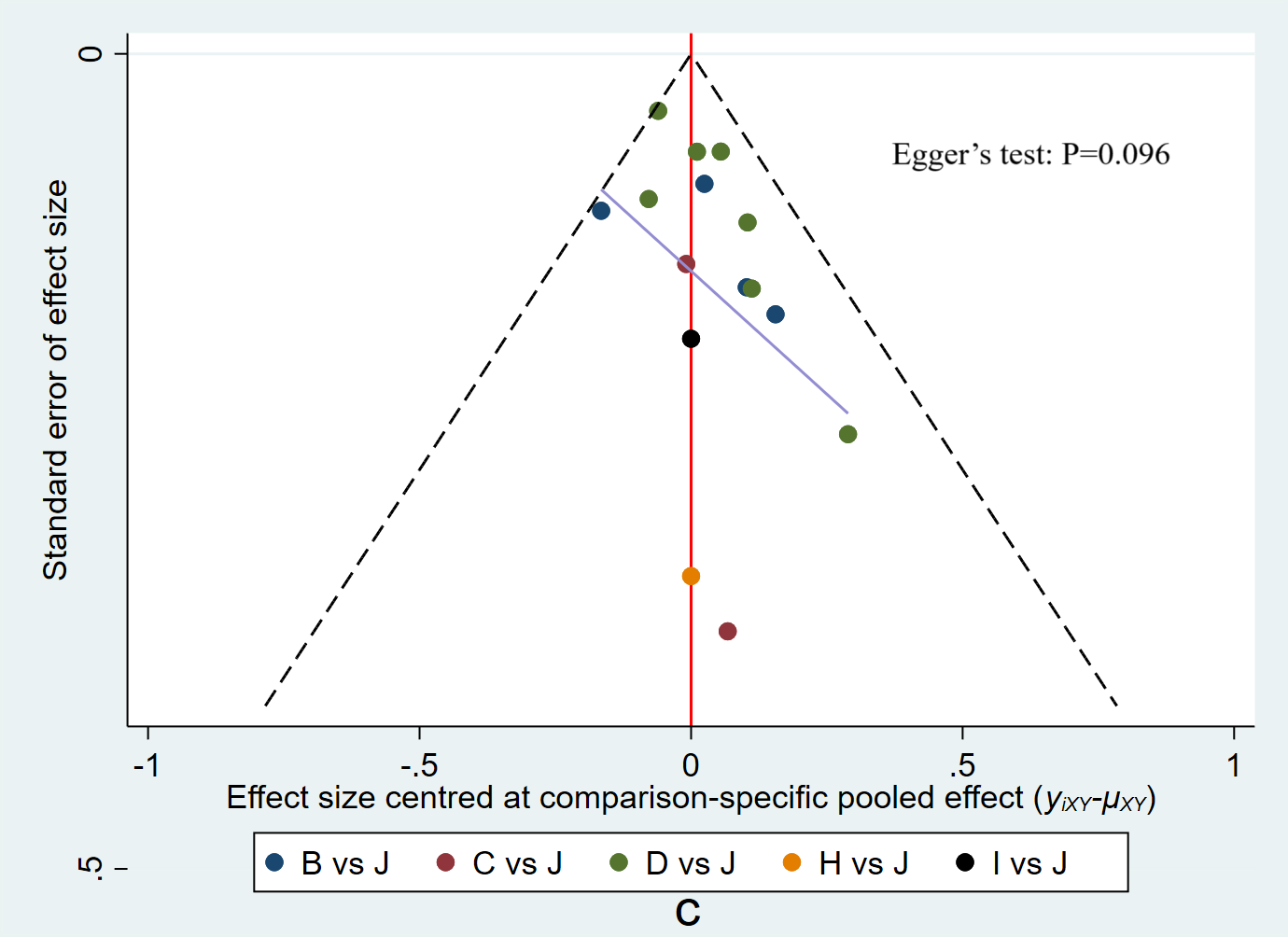


**d. SBP outcome: comparison adjusted funnel plot**


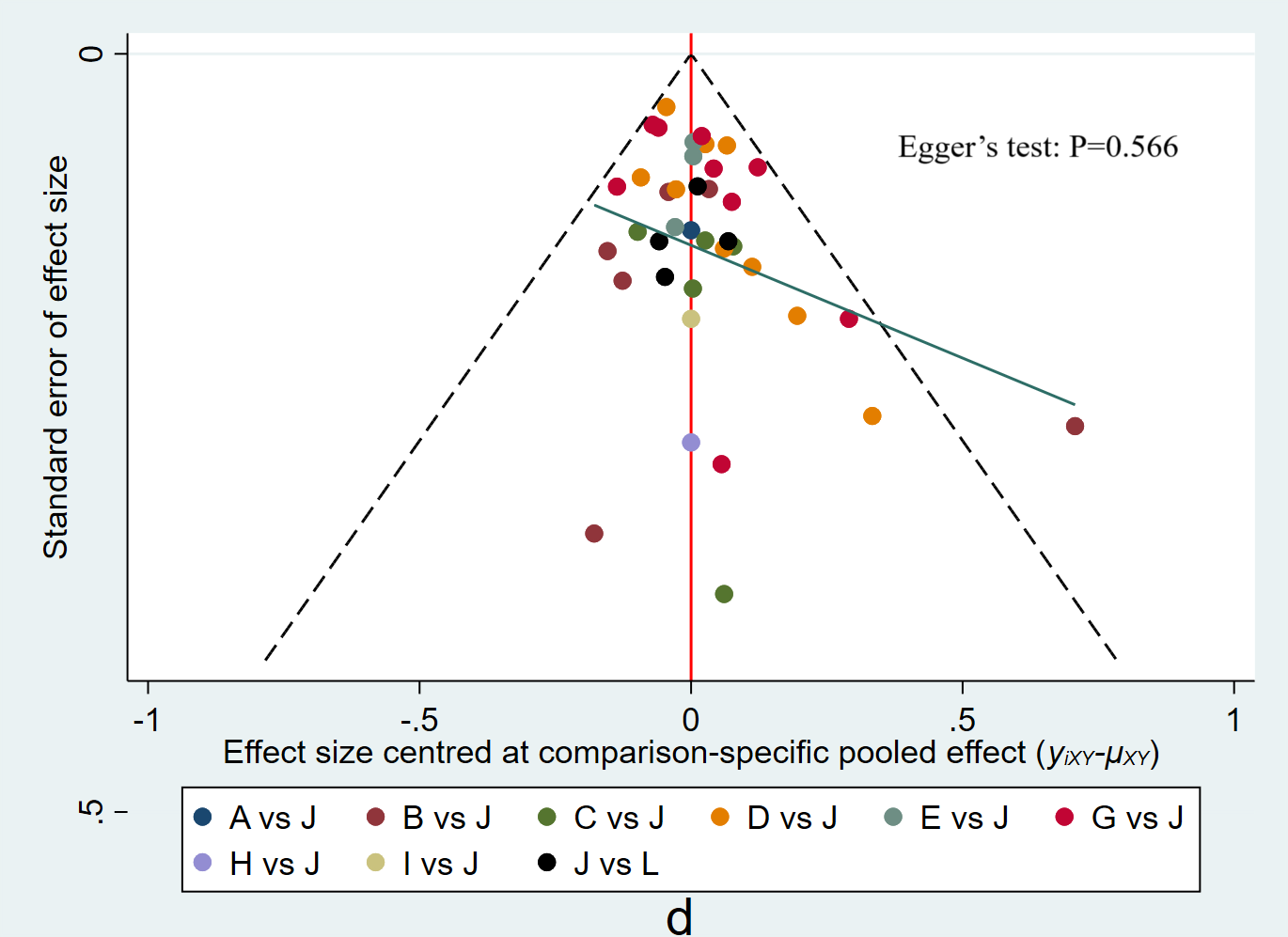


**e. DBP outcome: comparison adjusted funnel plot**
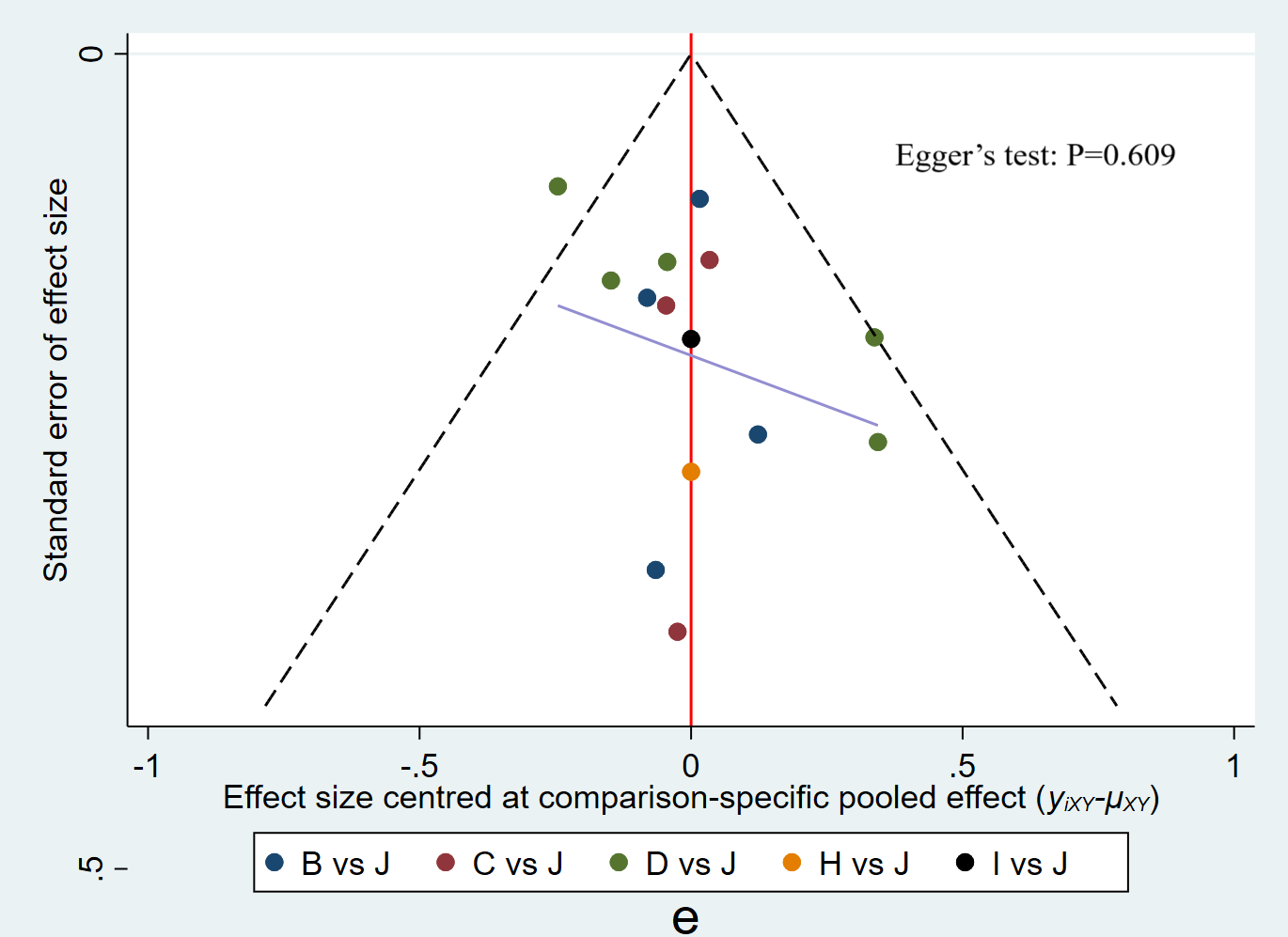


**f. Body Weight outcome: comparison adjusted funnel plot**
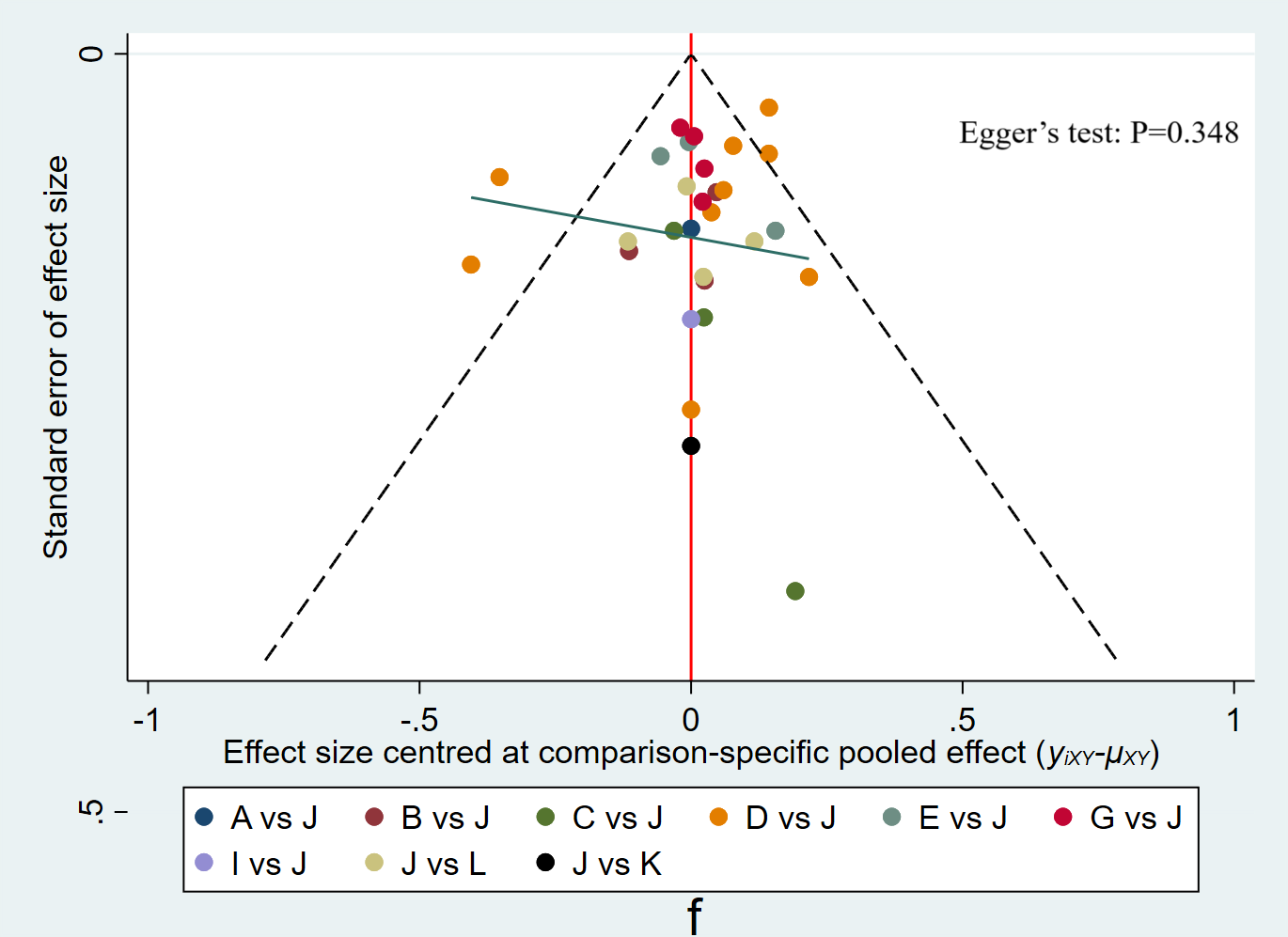


**g. any AE outcome: comparison adjusted funnel plot**
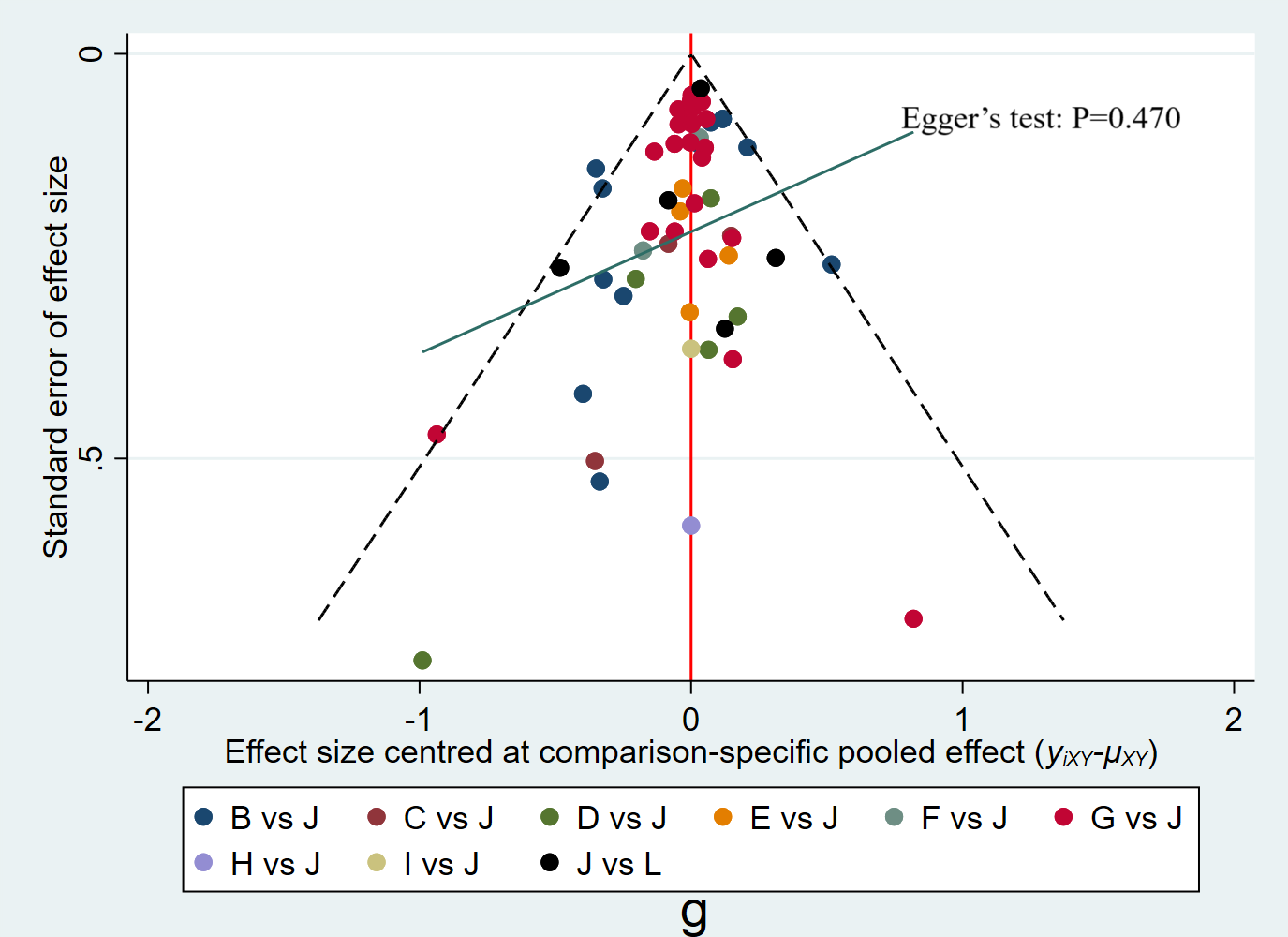


**h. UTI outcome: comparison adjusted funnel plot**


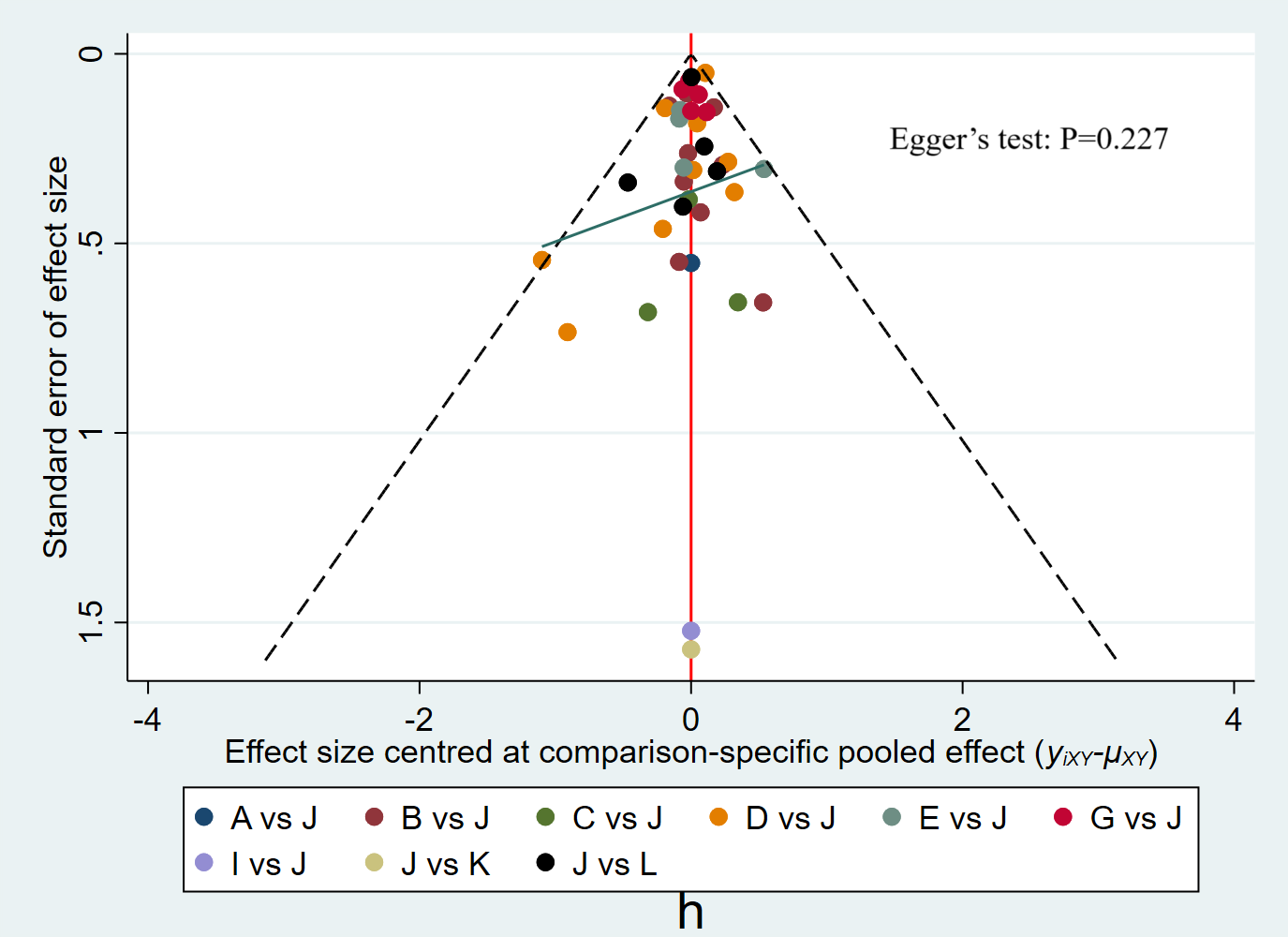


**i. Hypoglycemia outcome: comparison adjusted funnel plot**


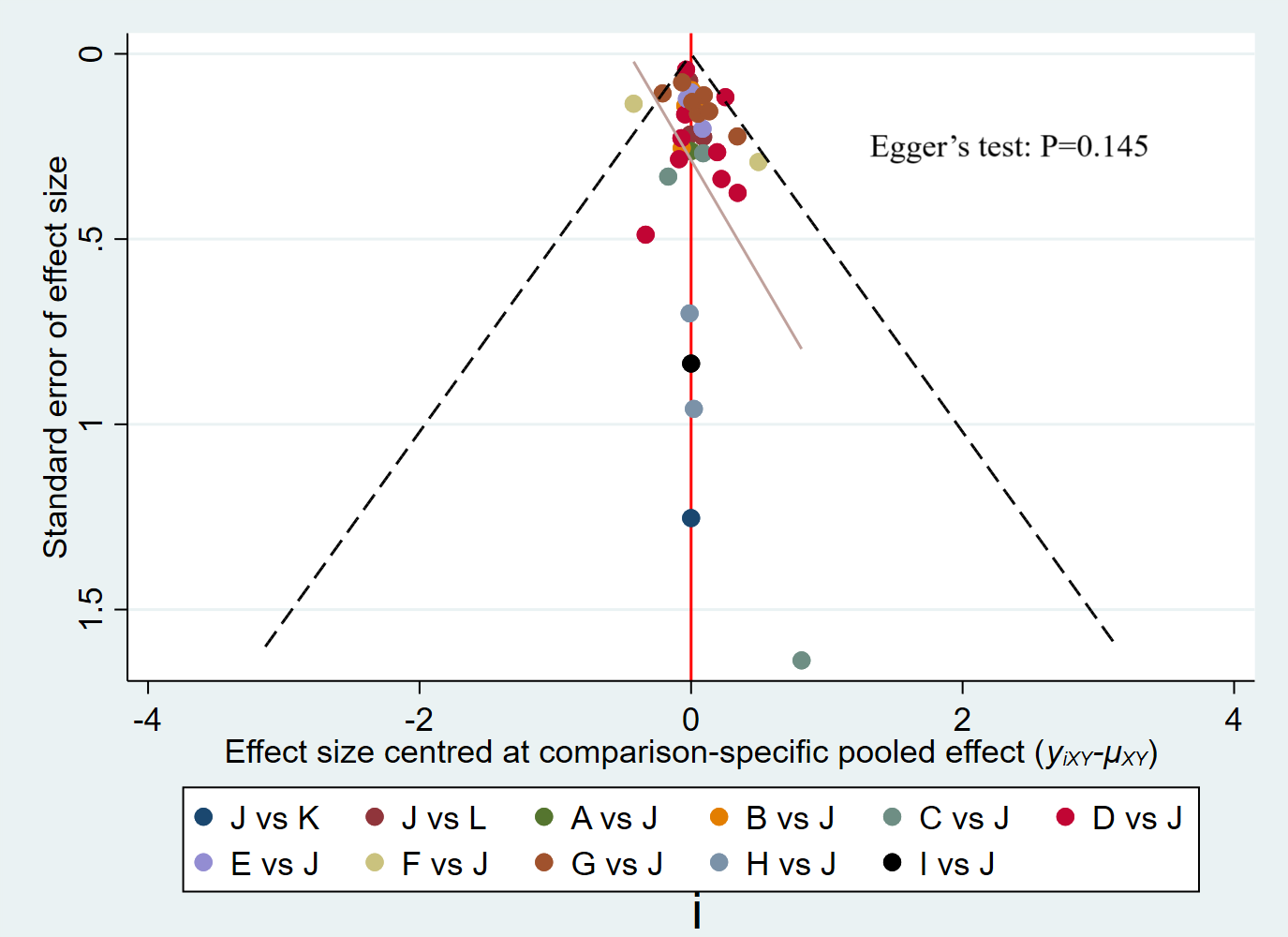


**j. AKI outcome: comparison adjusted funnel plot**


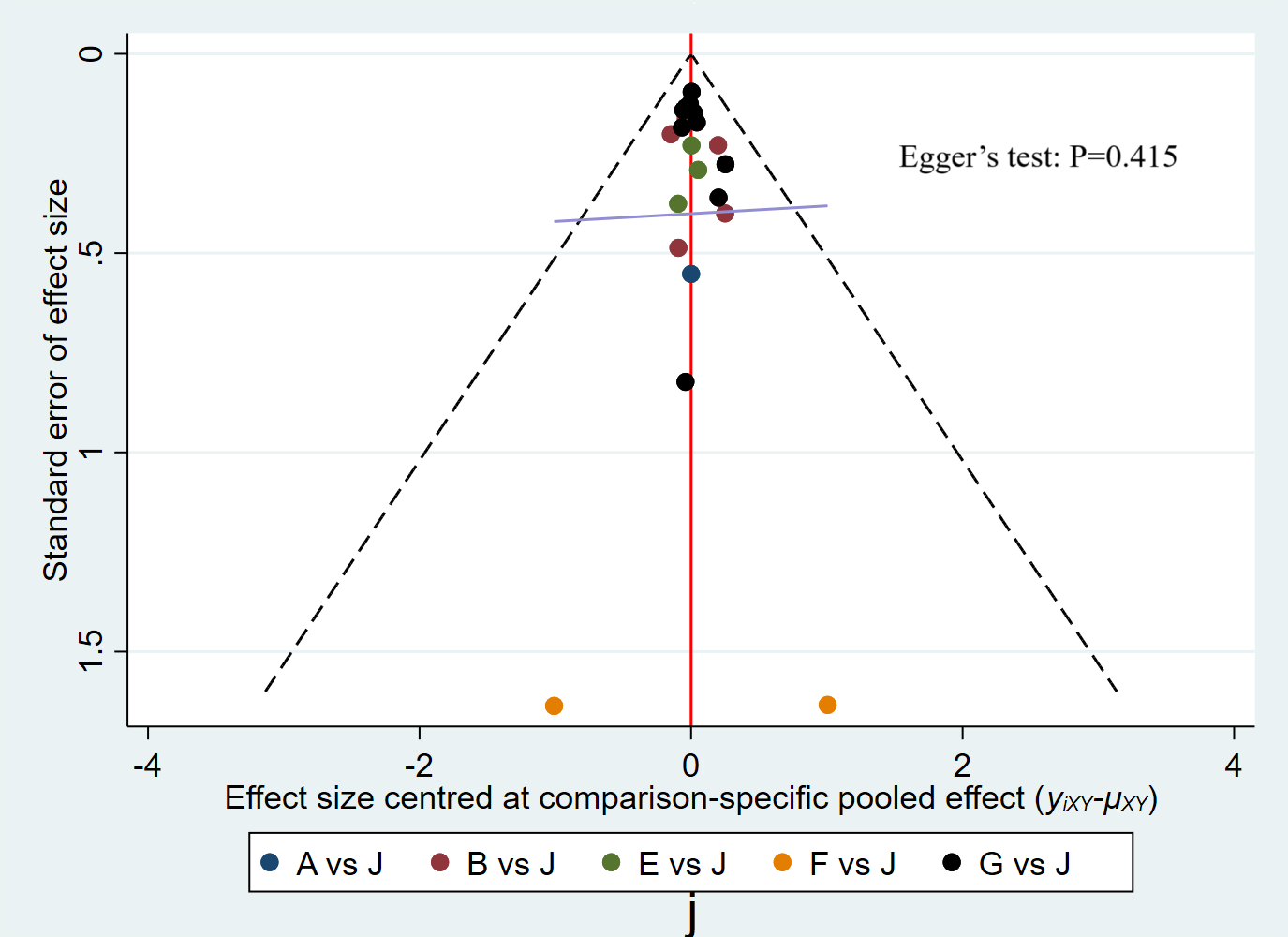


The dots in the diagram represent the literature included in the analysis. In an unbiased scenario, these points on the funnel plot should exhibit a symmetrical distribution around the estimated true value of independent studies, forming an inverted funnel shape. However, if bias exists, an asymmetric funnel plot will be observed. The degree of asymmetry directly correlates with the extent of deviation.

A: Bexagliflozin, B: Canagliflozin, C: Dapagliflozin, D: Empagliflozin, E: Ertugliflozin, F: Exenatide, G: Finerenone, H: Liraglutide, I: Luseogliflozin, J: PBO, K: Semaglutide, L: Sotagliflozin.

**Appendix 18 Included studies**

| **No.** | **author** | **Published Year** | **Sample size** | | | **Age, Mean, y(SD)** | | | **treatment** | | | **outcomes** |
| --- | --- | --- | --- | --- | --- | --- | --- | --- | --- | --- | --- | --- |
|  |  |  | **PBO group** | **treatment group**  **1** | **treatment group**  **2** | **PBO group** | **treatment group**  **1** | **treatment group**  **2** | **PBO group** | **treatment group**  **1** | **treatment group**  **2** |  |
| 1 | J.-F. Yale | 2013 | 90 | 90 | 89 | 68.2 (8.4) | 69.5 (8.2) | 67.9 (8.2) | PBO | Canagliflozin | Canagliflozin | ①②③④⑤⑥⑦⑩ |
| 2 | Zuoping Chen | 2016 | 32 | 32 | NI | NI | NI | NI | PBO | Liraglutide | NI | ①②③④⑤⑧ |
| 3 | Weizhen Gao | 2022 | 26 | 26 | NI | 60.25 (1.52) | 60.32 (1.66) | NI | PBO | Liraglutide | NI | ①②⑧⑩ |
| 4 | Anthony H Barnett | 2014 | 95 | 98 | 97 | 62.6 (8.1) | 63.2 (8.5) | 62.0 (8.4) | PBO | Empagliflozin | Empagliflozin | ①②③④⑤⑥⑦⑧⑩ |
|  |  |  | 187 | 187 | NI | 65.1 (8.2) | 64.6 (8.9) | 64.6 (8.9) | PBO | Empagliflozin | NI | ①②③④⑤⑥⑦⑧⑩ |
|  |  |  | 37 | 37 | NI | 62.9 (11.9) | 65.4 (10.2) | 65.4 (10.2) | PBO | Empagliflozin | NI | ①②③④⑤⑥⑦⑧⑩ |
| **No.** | **author** | **Published Year** | **Sample size** | | | **Age, Mean, y(SD)** | | | **treatment** | | | **outcomes** |
|  |  |  | **PBO group** | **treatment group**  **1** | **treatment group**  **2** | **PBO group** | **treatment group**  **1** | **treatment group**  **2** | **PBO group** | **treatment group**  **1** | **treatment group**  **2** |  |
| 5 | Sylvia E. Rosas | 2023 | 1034 | 1065 | NI | 64.2 (9.7) | | NI | PBO | Finerenone | NI | ⑩ |
| 6 | Hala Yamout | 2014 | 266 | 216 | 239 | 66.3 (7.5) | 66.2 (8.0) | 66.3 (6.9) | PBO | Canagliflozin | Canagliflozin | ①②③④⑥⑦⑩ |
|  |  |  | 116 | 122 | 126 | 68.4 (7.6) | 69.5 (7.9) | 68.1 (8.1) | PBO | Canagliflozin | Canagliflozin | ①②③④⑥⑦⑩ |
| 7 | David Cherney | 2016 | 248 | 388 | NI | 60.4 (10.2) | 58.4 (10.5) | NI | PBO | Empagliflozin | NI | ①②④⑤⑥⑦⑧⑩ |
|  |  |  | 87 | 128 | NI | 60.5 (10.3) | 59.3 (9.9) | NI | PBO | Empagliflozin | NI | ①②④⑤⑥⑦⑧⑩ |
| 8 | David Z.Cherney | 2022 | 260 | 263 | 264 | 69.3 (8.1) | 69.6 (7.5) | 69.5 (8.2) | PBO | Sotagliflozin | Sotagliflozin | ①②④⑥⑦⑧⑩ |
| **No.** | **author** | **Published Year** | **Sample size** | | | **Age, Mean, y(SD)** | | | **treatment** | | | **outcomes** |
|  |  |  | **PBO group** | **treatment group**  **1** | **treatment group**  **2** | **PBO group** | **treatment group**  **1** | **treatment group**  **2** | **PBO group** | **treatment group**  **1** | **treatment group**  **2** |  |
| 9 | Viktor Rotbain Curovic | 2022 | 15 | 17 | NI | 62.3 (7.8) | 63.7 (9.0) | NI | PBO | Dapagliflozin | NI | ①②③④⑤⑥ |
| 10 | Hiroyuki Takashima | 2018 | 20 | 20 | NI | 65.4 (10.4) | 64.7 (9.8) | NI | PBO | Dapagliflozin | NI | ①②④⑤ |
| 11 | Suvanjaa Sivalingam | 2023 | 30 | 30 | NI | 69.4 (9.1) | 70.5 (6.8) | NI | PBO | Semaglutide | NI | ①②⑥⑦⑧ |
| 12 | Carol Pollock | 2019 | 148 | 145 | NI | 64.7 (8.5) | 64.7 (8.6) | NI | PBO | Dapagliflozin | NI | ①②③④⑦⑧⑩ |
| 13 | Daisuke Koya | 2023 | 2831 | 2827 | NI | 63 (10) | | NI | PBO | Finerenone | NI | ④⑥⑧⑨⑩ |
| 14 | Cristian Guja | 2020 | 1043 | 772 | NI | 56.9 (9.5) | 56.9 (9.3) | NI | PBO | Exenatide | NI | ②⑦⑨⑩ |
|  |  |  | 207 | 182 | NI | 62.2 (9.0) | 62.5 (9.0) | NI | PBO | Exenatide | NI | ②⑧⑨⑩ |
| **No.** | **author** | **Published Year** | **Sample size** | | | **Age, Mean, y(SD)** | | | **treatment** | | | **outcomes** |
|  |  |  | **PBO group** | **treatment group**  **1** | **treatment group**  **2** | **PBO group** | **treatment group**  **1** | **treatment group**  **2** | **PBO group** | **treatment group**  **1** | **treatment group**  **2** |  |
| 15 | Paola Fioretto | 2018 | 161 | 160 | NI | 66.2 (NI) | 65.3 (NI) | NI | PBO | Dapagliflozin | NI | ①②④⑥⑦⑧⑩ |
| 16 | Donald E. Kohan | 2013 | 84 | 83 | 85 | 67 (8.6) | 66 (8.9) | 68 (7.7) | PBO | Dapagliflozin | Dapagliflozin | ①②④⑤⑥⑦⑧⑩ |
| 17 | Andrew S. Allegretti | 2019 | 155 | 157 | NI | 69.9 (8.29) | 69.3 (8.36) | NI | PBO | Bexagliflozin | NI | ①②④⑥⑦⑧⑨ |
| 18 | Masakazu Haneda | 2016 | 50 | 95 | NI | 68.4 (8.9) | 67.9 (8.9) | NI | PBO | Luseogliflozin | NI | ①②③④⑤⑥⑦⑧⑩ |
| 19 | Katherine R. Tuttle | 2022 | 519 | 1003 | NI | 67.1 (8.1) | 67.1 (7.5) | NI | PBO | Empagliflozin | NI | ⑦⑧ |
|  |  |  | 277 | 445 | NI | 67.9 (8.2) | 67.7 (8.7) | NI | PBO | Empagliflozin | NI | ⑦⑧ |
|  |  |  | 52 | 71 | NI | 63.7 (10.7) | 68.8 (9.1) | NI | PBO | Empagliflozin | NI | ⑦⑧ |
| **No.** | **author** | **Published Year** | **Sample size** | | | **Age, Mean, y(SD)** | | | **treatment** | | | **outcomes** |
|  |  |  | **PBO group** | **treatment group**  **1** | **treatment group**  **2** | **PBO group** | **treatment group**  **1** | **treatment group**  **2** | **PBO group** | **treatment group**  **1** | **treatment group**  **2** |  |
| 20 | Pantelis Sarafidis | 2022 | 450 | 440 | NI | 67 (9) | 67 (9) | NI | PBO | Finerenone | NI | ②④⑨⑩ |
| 21 | Kenneth W. Mahaffey | 2019 | 1092 | 1089 | NI | 61.7 (9.4) | 61.1 (9.7) | NI | PBO | Canagliflozin | NI | ⑦⑧⑨⑩ |
|  |  |  | 1107 | 1113 | NI | 64.6 (8.9) | 64.6 (8.2) | NI | PBO | Canagliflozin | NI | ⑦⑧⑨⑩ |
| 22 | Takashi Wada | 2022 | 154 | 154 | NI | 62.4 (11.1) | 62.5 (10.5) | NI | PBO | Canagliflozin | NI | ①②④⑤⑦⑧⑩ |
| 23 | George Bakris | 2020 | 90 | 84 | NI | 66 (9) | 64 (10) | NI | PBO | Canagliflozin | NI | ②⑨⑩ |
| 24 | George Grunberger | 2018 | 154 | 158 | 155 | 67.5 (8.9) | 66.7 (8.3) | 67.5 (8.5) | PBO | Ertugliflozin | Ertugliflozin | ①②⑦⑩ |
| 25 | Samuel Dagogo-Jack | 2021 | 598 | 618 | 560 | 68.0 (7.5) | 68.3 (7.7) | 68.2 (7.5) | PBO | Ertugliflozin | Ertugliflozin | ①②④⑥⑦⑧⑨⑩ |
| **No.** | **author** | **Published Year** | **Sample size** | | | **Age, Mean, y(SD)** | | | **treatment** | | | **outcomes** |
|  |  |  | **PBO group** | **treatment group**  **1** | **treatment group**  **2** | **PBO group** | **treatment group**  **1** | **treatment group**  **2** | **PBO group** | **treatment group**  **1** | **treatment group**  **2** |  |
| 26 | David Z. I. Cherney | 2021 | 93 | 92 | 92 | 68.0 (8.3) | 66.8 (10) | 67.3 (9.6) | PBO | Sotagliflozin | Sotagliflozin | ①②④⑥⑦⑧⑩ |
| 27 | Deepak L. Bhatt | 2020 | 5292 | 5292 | NI | NI | NI | NI | PBO | Sotagliflozin | NI | ⑦⑧⑩ |
| 28 | B. Pitt, | 2021 | 3666 | 3686 | NI | 64.1 (10) | 64.1 (9.7) | NI | PBO | Finerenone | NI | ①④⑥⑦⑧⑨⑩ |
| 29 | George L. Bakris | 2020 | 2841 | 2833 | NI | 65.7 (9.2) | 65.4 (8.9) | NI | PBO | Finerenone | NI | ①②④⑥⑦⑧⑨⑩ |
| 30 | Gerasimos Filippatos | 2021 | 1302 | 1303 | NI | 67.1 (8.4) | 66 (8.2) | NI | PBO | Finerenone | NI | ②④⑦⑧⑨⑩ |
|  |  |  | 1539 | 1530 | NI | 64.5 (9.6) | 64.4 (9.4) | NI | PBO | Finerenone | NI | ②④⑦⑧⑨⑩ |
| 31 | Rajiv Agarwal | 2023 | 27 | 92 | NI | 60.8 (8.4) | NI | NI | PBO | Finerenone | NI | ④⑩ |
| **No.** | **author** | **Published Year** | **Sample size** | | | **Age, Mean, y(SD)** | | | **treatment** | | | **outcomes** |
|  |  |  | **PBO group** | **treatment group**  **1** | **treatment group**  **2** | **PBO group** | **treatment group**  **1** | **treatment group**  **2** | **PBO group** | **treatment group**  **1** | **treatment group**  **2** |  |
| 32 | George L. Bakris | 2015 | 94 | 727 | NI | 63.26 (8.68) | NI | NI | PBO | Finerenone | NI | ②⑩ |
| 33 | Christoph Wanner | 2018 | 752 | 1498 | NI | 66 (8.5) | 66.2 (8) | NI | PBO | Empagliflozin | NI | ①③④⑥ |
| 34 | V. Perkovic | 2019 | 2199 | 2202 | NI | 63.2 (9.2) | 62.9 (9.2) | NI | PBO | Canagliflozin | NI | ①②④⑤⑦⑧⑨⑩ |
| 35 | Rajiv Agarwal | 2021 | 2328 | 2291 | NI | 65.4 (9.3) | 65.2 (9.0) | NI | PBO | Finerenone | NI | ⑨⑩ |
| 36 | Takashi Wada | 2021 | 303 | 301 | NI | 60.9 (9.1) | 60.6 (9.1) | NI | PBO | Canagliflozin | NI | ①②④⑤⑦⑨⑩ |
|  |  |  | 1894 | 1899 | NI | 63.5 (9.2) | 63.2 (9.1) | NI | PBO | Canagliflozin | NI | ①②④⑤⑦⑨⑩ |
| 37 | Haitao Zhang | 2023 | 184 | 188 | NI | 60.68(10.13) | 59.85 (10.16) | NI | PBO | Finerenone | NI | ⑨⑩ |
| **No.** | **author** | **Published Year** | **Sample size** | | | **Age, Mean, y(SD)** | | | **treatment** | | | **outcomes** |
|  |  |  | **PBO group** | **treatment group**  **1** | **treatment group**  **2** | **PBO group** | **treatment group**  **1** | **treatment group**  **2** | **PBO group** | **treatment group**  **1** | **treatment group**  **2** |  |
| 38 | Rajiv Agarwal | 2022 | 6507 | 6519 | NI | 64.8 (9.7) | 64.7 (9.4) | NI | PBO | Finerenone | NI | ④⑦⑧⑨⑩ |
| 39 | Nikolaos Perakakis | 2023 | 5297 | 5340 | NI | 64.31 (9.56) | 64.29 (9.27) | NI | PBO | Finerenone | NI | ⑩ |
|  |  |  | 1025 | 1067 | NI | 61.71(10.08) | 61.94 (9.76) | NI | PBO | Finerenone | NI | ⑩ |
|  |  |  | 139 | 152 | NI | 72.40 (8.02) | 70.89 (7.06) | NI | PBO | Finerenone | NI | ⑩ |

Abbreviations: ①Change in HbA1c from baseline; ②Change in eGFR from baseline; ③Change in LDL-C from baseline; ④Change in SBP from baseline; ⑤Change in DBP from baseline; ⑥Change in body weight from baseline; ⑦UTI; ⑧Hypoglycemia; ⑨AKI; ⑩any AE. NI, No Information. PBO: PBO group.

**Appendix 19 References**

Afkarian, Maryam, Leila R. Zelnick, Yoshio N. Hall, Patrick J. Heagerty, Katherine Tuttle, Noel S. Weiss, and Ian H. De Boer. 2016. “Clinical Manifestations of Kidney Disease among US Adults with Diabetes, 1988-2014.” *JAMA - Journal of the American Medical Association* 316 (6): 602–10. https://doi.org/10.1001/jama.2016.10924.

Agarwal, Rajiv, Stefan D. Anker, Gerasimos Filippatos, Bertram Pitt, Peter Rossing, Luis M. Ruilope, John Boletis, et al. 2022. “Effects of Canagliflozin versus Finerenone on Cardiorenal Outcomes: Exploratory Post Hoc Analyses from FIDELIO-DKD Compared to Reported CREDENCE Results.” *Nephrology Dialysis Transplantation* 37 (7): 1261–69. https://doi.org/10.1093/ndt/gfab336.

Agarwal, Rajiv, Gerasimos Filippatos, Bertram Pitt, Stefan D. Anker, Peter Rossing, Amer Joseph, Peter Kolkhof, et al. 2022. “Cardiovascular and Kidney Outcomes with Finerenone in Patients with Type 2 Diabetes and Chronic Kidney Disease: The FIDELITY Pooled Analysis.” *European Heart Journal* 43 (6): 474-484A. https://doi.org/10.1093/eurheartj/ehab777.

Agarwal, Rajiv, Luis M. Ruilope, Gema Ruiz-Hurtado, Hermann Haller, Roland E. Schmieder, Stefan D. Anker, Gerasimos Filippatos, et al. 2023. “Effect of Finerenone on Ambulatory Blood Pressure in Chronic Kidney Disease in Type 2 Diabetes.” *Journal of Hypertension* 41 (2): 295–302. https://doi.org/10.1097/HJH.0000000000003330.

Allegretti, Andrew S., Wenbin Zhang, Wenjiong Zhou, Tara K. Thurber, Scott P. Rigby, Cynthia Bowman-Stroud, Carlos Trescoli, Pierre Serusclat, Mason W. Freeman, and Yuan Di C. Halvorsen. 2019. “Safety and Effectiveness of Bexagliflozin in Patients With Type 2 Diabetes Mellitus and Stage 3a/3b CKD.” *American Journal of Kidney Diseases* 74 (3): 328–37. https://doi.org/10.1053/j.ajkd.2019.03.417.

Apperloo, Ellen M, Brendon L Neuen, Robert A Fletcher, Niels Jongs, Stefan D Anker, Deepak L Bhatt, Javed Butler, et al. 2024. “Efficacy and Safety of SGLT2 Inhibitors with and without Glucagon-like Peptide 1 Receptor Agonists: A SMART-C Collaborative Meta-Analysis of Randomised Controlled Trials.” *The Lancet Diabetes & Endocrinology* 12 (8): 545–57. https://doi.org/10.1016/S2213-8587(24)00155-4.

Association, American Diabetes. 2020. “2. Classification and Diagnosis of Diabetes: Standards of Medical Care in Diabetes—2021.” *Diabetes Care* 44 (Supplement_1): S15–33. https://doi.org/10.2337/dc21-S002.

Bakris, George L., Rajiv Agarwal, Stefan D. Anker, Bertram Pitt, Luis M. Ruilope, Peter Rossing, Peter Kolkhof, et al. 2020. “Effect of Finerenone on Chronic Kidney Disease Outcomes in Type 2 Diabetes.” *New England Journal of Medicine* 383 (23): 2219–29. https://doi.org/10.1056/nejmoa2025845.

Bakris, George L., Rajiv Agarwal, Juliana C. Chan, Mark E. Cooper, Ron T. Gansevoort, Hermann Haller, Giuseppe Remuzzi, et al. 2015. “Effect of Finerenone on Albuminuria in Patients with Diabetic Nephropathy a Randomized Clinical Trial.” *JAMA - Journal of the American Medical Association* 314 (9): 884–94. https://doi.org/10.1001/jama.2015.10081.

Bakris, George L., Mark Williams, Lance Dworkin, William J. Elliott, Murray Epstein, Robert Toto, Katherine Tuttle, Janice Douglas, Willa Hsueh, and James Sowers. 2000. “Preserving Renal Function in Adults with Hypertension and Diabetes: A Consensus Approach.” *American Journal of Kidney Diseases* 36 (3): 646–61. https://doi.org/10.1053/ajkd.2000.16225.

Bakris, George, Megumi Oshima, Kenneth W. Mahaffey, Rajiv Agarwal, Christopher P. Cannon, George Capuano, David M. Charytan, et al. 2020. “Effects of Canagliflozin in Patients with Baseline EGFR <30 Ml/Min per 1.73 M2: Subgroup Analysis of the Randomized CREDENCE Trial.” *Clinical Journal of the American Society of Nephrology* 15 (12): 1705–14. https://doi.org/10.2215/CJN.10140620.

Barnett, Anthony H., Ambrish Mithal, Jenny Manassie, Russell Jones, Henning Rattunde, Hans J. Woerle, and Uli C. Broedl. 2014. “Efficacy and Safety of Empagliflozin Added to Existing Antidiabetes Treatment in Patients with Type 2 Diabetes and Chronic Kidney Disease: A Randomised, Double-Blind, Placebo-Controlled Trial.” *The Lancet Diabetes and Endocrinology* 2 (5): 369–84. https://doi.org/10.1016/S2213-8587(13)70208-0.

Bhatt, Deepak L., Michael Szarek, Bertram Pitt, Christopher P. Cannon, Lawrence A. Leiter, Darren K. McGuire, Julia B. Lewis, et al. 2021. “Sotagliflozin in Patients with Diabetes and Chronic Kidney Disease.” *New England Journal of Medicine* 384 (2): 129–39. https://doi.org/10.1056/nejmoa2030186.

Brown, Emily, Surya P. Rajeev, Daniel J. Cuthbertson, and John P. H. Wilding. 2019. “A Review of the Mechanism of Action, Metabolic Profile and Haemodynamic Effects of Sodium‐glucose Co‐transporter‐2 Inhibitors.” *Diabetes, Obesity and Metabolism* 21 (S2): 9–18. https://doi.org/10.1111/dom.13650.

Bzowyckyj, Andrew. 2020. “Managing the Multifaceted Nature of Type 2 Diabetes Using Once‐weekly Injectable GLP‐1 Receptor Agonist Therapy.” *Journal of Clinical Pharmacy and Therapeutics* 45 (S1): 7–16. https://doi.org/10.1111/jcpt.13229.

Cherney, David, Søren S. Lund, Bruce A. Perkins, Per Henrik Groop, Mark E. Cooper, Stefan Kaspers, Egon Pfarr, Hans J. Woerle, and Maximilian von Eynatten. 2016. “The Effect of Sodium Glucose Cotransporter 2 Inhibition with Empagliflozin on Microalbuminuria and Macroalbuminuria in Patients with Type 2 Diabetes.” *Diabetologia* 59 (9): 1860–70. https://doi.org/10.1007/s00125-016-4008-2.

Cherney, David Z.I., Ele Ferrannini, Guillermo E. Umpierrez, Anne L. Peters, Julio Rosenstock, Amy K. Carroll, Pablo Lapuerta, Phillip Banks, and Rajiv Agarwal. 2021. “Efficacy and Safety of Sotagliflozin in Patients with Type 2 Diabetes and Severe Renal Impairment.” *Diabetes, Obesity and Metabolism* 23 (12): 2632–42. https://doi.org/10.1111/dom.14513.

Cherney, David Z.I., Ele Ferrannini, Guillermo E. Umpierrez, Anne L. Peters, Julio Rosenstock, David R. Powell, Michael J. Davies, Phillip Banks, and Rajiv Agarwal. 2023. “Efficacy and Safety of Sotagliflozin in Patients with Type 2 Diabetes and Stage 3 Chronic Kidney Disease.” *Diabetes, Obesity and Metabolism* 25 (6): 1646–57. https://doi.org/10.1111/dom.15019.

Chilton, R., I. Tikkanen, C. P. Cannon, S. Crowe, H. J. Woerle, U. C. Broedl, and O. E. Johansen. 2015. “Effects of Empagliflozin on Blood Pressure and Markers of Arterial Stiffness and Vascular Resistance in Patients with Type 2 Diabetes.” *Diabetes, Obesity and Metabolism* 17 (12): 1180–93. https://doi.org/10.1111/dom.12572.

Chinese Diabetes Society. 2025. ‘Guideline for the prevention and treatment of diabetes mellitus in China (2024 edition)’. Chinese Journal of Diabetes Mellitus 17 (1): 16–139. https://doi.org/10.3760/cma.j.cn115791-20241203-00705.

Committee, American Diabetes Association Professional Practice. 2023. “11. Chronic Kidney Disease and Risk Management: Standards of Care in Diabetes—2024.” *Diabetes Care* 47 (Supplement_1): S219–30. https://doi.org/10.2337/dc24-S011.

Curovic, Viktor Rotbain, Mie Klessen Eickhoff, Teemu Rönkkä, Marie Frimodt-Møller, Tine Willum Hansen, Harald Mischak, Peter Rossing, Tarunveer Singh Ahluwalia, and Frederik Persson. 2022. “Dapagliflozin Improves the Urinary Proteomic Kidney-Risk Classifier CKD273 in Type 2 Diabetes with Albuminuria: A Randomized Clinical Trial.” *Diabetes Care* 45 (11): 2662–68. https://doi.org/10.2337/dc22-1157.

Dagogo-Jack, Samuel, Richard E. Pratley, David Z.I. Cherney, Darren K. McGuire, Francesco Cosentino, Weichung J. Shih, Jie Liu, et al. 2021. “Glycemic Efficacy and Safety of the SGLT2 Inhibitor Ertugliflozin in Patients with Type 2 Diabetes and Stage 3 Chronic Kidney Disease: An Analysis from the VERTIS CV Randomized Trial.” *BMJ Open Diabetes Research & Care* 9 (1). https://doi.org/10.1136/bmjdrc-2021-002484.

Drucker, Daniel J. 2016. “The Cardiovascular Biology of Glucagon-like Peptide-1.” *Cell Metabolism* 24 (1): 15–30. https://doi.org/10.1016/j.cmet.2016.06.009.

Filippatos, Gerasimos, Stefan D. Anker, Rajiv Agarwal, Bertram Pitt, Luis M. Ruilope, Peter Rossing, Peter Kolkhof, et al. 2021. “Finerenone and Cardiovascular Outcomes in Patients with Chronic Kidney Disease and Type 2 Diabetes.” *Circulation* 143 (6): 540–52. https://doi.org/10.1161/CIRCULATIONAHA.120.051898.

Filippatos, Theodosios D, and Moses S Elisaf. 2013. “Effects of Glucagon-like Peptide-1 Receptor Agonists on Renal Function.” *World Journal of Diabetes* 4 (5): 190. https://doi.org/10.4239/wjd.v4.i5.190.

Fioretto, Paola, Stefano Del Prato, John B. Buse, Ronald Goldenberg, Francesco Giorgino, Daniel Reyner, Anna Maria Langkilde, C. David Sjöström, and Peter Sartipy. 2018. “Efficacy and Safety of Dapagliflozin in Patients with Type 2 Diabetes and Moderate Renal Impairment (Chronic Kidney Disease Stage 3A): The DERIVE Study.” *Diabetes, Obesity and Metabolism* 20 (11): 2532–40. https://doi.org/10.1111/dom.13413.

Fujita, Hiroki, Tsukasa Morii, Hiromi Fujishima, Takehiro Sato, Tatsunori Shimizu, Mihoko Hosoba, Katsushi Tsukiyama, et al. 2014. “The Protective Roles of GLP-1R Signaling in Diabetic Nephropathy: Possible Mechanism and Therapeutic Potential.” *Kidney International* 85 (3): 579–89. https://doi.org/10.1038/ki.2013.427.

Garvey, W Timothy. 2022. “New Horizons. A New Paradigm for Treating to Target with Second-Generation Obesity Medications.” *The Journal of Clinical Endocrinology & Metabolism* 107 (4): e1339–47. https://doi.org/10.1210/clinem/dgab848.

Giglio, Rosaria Vincenza, Anca Pantea Stoian, Khalid Al-Rasadi, Maciej Banach, Angelo Maria Patti, Marcello Ciaccio, Ali A. Rizvi, and Manfredi Rizzo. 2021. “Novel Therapeutical Approaches to Managing Atherosclerotic Risk.” *International Journal of Molecular Sciences* 22 (9): 4633. https://doi.org/10.3390/ijms22094633.

Grunberger Sarah Camp Jeremy Johnson Susan Huyck Steven Terra James P Mancuso Zhi Wei Jiang Gregory Golm Samuel S Engel Brett Lauring, George G. n.d. “Ertugliflozin in Patients with Stage 3 Chronic Kidney Disease and Type 2 Diabetes Mellitus: The VERTIS RENAL Randomized Study.” https://doi.org/10.1007/s13300.

Guja Juan Frías Lisa Suchower Elise Hardy Galina Marr C David Sjöström Serge A Jabbour, Cristian P. n.d. “Safety and Efficacy of Exenatide Once Weekly in Participants with Type 2 Diabetes and Stage 2/3 Chronic Kidney Disease.” https://doi.org/10.6084/m9.figshare.12059103.

Haneda, Masakazu, Yutaka Seino, Nobuya Inagaki, Kohei Kaku, Takashi Sasaki, Atsushi Fukatsu, Haruka Kakiuchi, Yuri Sato, Soichi Sakai, and Yoshishige Samukawa. 2016. “Influence of Renal Function on the 52-Week Efficacy and Safety of the Sodium Glucose Cotransporter 2 Inhibitor Luseogliflozin in Japanese Patients with Type 2 Diabetes Mellitus.” *Clinical Therapeutics* 38 (1): 66-88.e20. https://doi.org/10.1016/j.clinthera.2015.10.025.

Heerspink, Hiddo J.L., Bruce A. Perkins, David H. Fitchett, Mansoor Husain, and David Z. I. Cherney. 2016. “Sodium Glucose Cotransporter 2 Inhibitors in the Treatment of Diabetes Mellitus.” *Circulation* 134 (10): 752–72. https://doi.org/10.1161/CIRCULATIONAHA.116.021887.

Hutton, Brian, Georgia Salanti, Deborah M. Caldwell, Anna Chaimani, Christopher H. Schmid, Chris Cameron, John P.A. Ioannidis, et al. 2015. “The PRISMA Extension Statement for Reporting of Systematic Reviews Incorporating Network Meta-Analyses of Health Care Interventions: Checklist and Explanations.” *Annals of Internal Medicine* 162 (11): 777–84. https://doi.org/10.7326/M14-2385.

Kidokoro, Kengo, David Z.I. Cherney, Andrea Bozovic, Hajime Nagasu, Minoru Satoh, Eiichiro Kanda, Tamaki Sasaki, and Naoki Kashihara. 2019. “Evaluation of Glomerular Hemodynamic Function by Empagliflozin in Diabetic Mice Using In Vivo Imaging.” *Circulation* 140 (4): 303–15. https://doi.org/10.1161/CIRCULATIONAHA.118.037418.

Kohan, Donald E., Paola Fioretto, Weihua Tang, and James F. List. 2014. “Long-Term Study of Patients with Type 2 Diabetes and Moderate Renal Impairment Shows That Dapagliflozin Reduces Weight and Blood Pressure but Does Not Improve Glycemic Control.” *Kidney International* 85 (4): 962–71. https://doi.org/10.1038/ki.2013.356.

Koya, Daisuke, Stefan D. Anker, Luis M. Ruilope, Peter Rossing, Zhi Hong Liu, Byung Wan Lee, Chien Te Lee, et al. 2023. “Cardiorenal Outcomes with Finerenone in Asian Patients with Chronic Kidney Disease and Type 2 Diabetes: A FIDELIO-DKD Post Hoc Analysis.” *American Journal of Nephrology* 54 (9–10): 370–78. https://doi.org/10.1159/000532102.

Leehey, David J., Jane H. Zhang, Nicholas V. Emanuele, Adam Whaley-Connell, Paul M. Palevsky, Robert F. Reilly, Peter Guarino, and Linda F. Fried. 2015. “BP and Renal Outcomes in Diabetic Kidney Disease.” *Clinical Journal of the American Society of Nephrology* 10 (12): 2159–69. https://doi.org/10.2215/CJN.02850315.

Liu, Chao, Hongrong Xu, Fei Yuan, Hanjing Chen, Lei Sheng, Weili Chen, Haisong Xie, Hongmei Xu, and Xuening Li. 2023. “Evaluating the Bioequivalence and Safety of Liraglutide Injection versus Victoza® in Healthy Chinese Subjects: A Randomized, Open, Two-Cycle, Self-Crossover Phase I Clinical Trial.” *Frontiers in Pharmacology* 14 (December). https://doi.org/10.3389/fphar.2023.1326865.

Lv, Ruolin, Lili Xu, Lin Che, Song Liu, Yangang Wang, and Bingzi Dong. 2023. “Cardiovascular-Renal Protective Effect and Molecular Mechanism of Finerenone in Type 2 Diabetic Mellitus.” *Frontiers in Endocrinology* 14 (February). https://doi.org/10.3389/fendo.2023.1125693.

Mahaffey, Kenneth W., Meg J. Jardine, Severine Bompoint, Christopher P. Cannon, Bruce Neal, Hiddo J.L. Heerspink, David M. Charytan, et al. 2019. “Canagliflozin and Cardiovascular and Renal Outcomes in Type 2 Diabetes Mellitus and Chronic Kidney Disease in Primary and Secondary Cardiovascular Prevention Groups: Results from the Randomized CREDENCE Trial.” *Circulation* 140 (9): 739–50. https://doi.org/10.1161/CIRCULATIONAHA.119.042007.

Mann, Johannes F.E., Vivian A. Fonseca, Neil R. Poulter, Itamar Raz, Thomas Idorn, Søren Rasmussen, Bernt Johan von Scholten, and Ofri Mosenzon. 2020. “Safety of Liraglutide in Type 2 Diabetes and Chronic Kidney Disease.” *Clinical Journal of the American Society of Nephrology* 15 (4): 465–73. https://doi.org/10.2215/CJN.11881019.

Moher, David, Alessandro Liberati, Jennifer Tetzlaff, and Douglas G. Altman. 2009. “Preferred Reporting Items for Systematic Reviews and Meta-Analyses: The PRISMA Statement.” *PLoS Medicine* 6 (7): e1000097. https://doi.org/10.1371/journal.pmed.1000097.

Nguyen, Bao‐Ngoc, Le Nguyen, Shweta Mital, Shawn Bugden, and Hai V. Nguyen. 2023. “Comparative Efficacy of Sodium‐glucose Co‐transporter‐2 Inhibitors, Glucagon‐like Peptide‐1 Receptor Agonists and Non‐steroidal Mineralocorticoid Receptor Antagonists in Chronic Kidney Disease and Type 2 Diabetes: A Systematic Review and Network Meta‐analysis.” *Diabetes, Obesity and Metabolism* 25 (6): 1614–23. https://doi.org/10.1111/dom.15009.

Park, Cheol Whee, Hyeong Wook Kim, Seung Hyun Ko, Ji Hee Lim, Gyeong Ryul Ryu, Hyun Wha Chung, Sang Woo Han, et al. 2007. “Long-Term Treatment of Glucagon-Like Peptide-1 Analog Exendin-4 Ameliorates Diabetic Nephropathy through Improving Metabolic Anomalies in Db/Db Mice.” *Journal of the American Society of Nephrology* 18 (4): 1227–38. https://doi.org/10.1681/ASN.2006070778.

Perakakis, Nikolaos, Stefan R. Bornstein, Andreas L. Birkenfeld, Andreas Linkermann, Münevver Demir, Stefan D. Anker, Gerasimos Filippatos, et al. 2024. “Efficacy of Finerenone in Patients with Type 2 Diabetes, Chronic Kidney Disease and Altered Markers of Liver Steatosis and Fibrosis: A FIDELITY Subgroup Analysis.” *Diabetes, Obesity and Metabolism* 26 (1): 191–200. https://doi.org/10.1111/dom.15305.

Perkovic, Vlado, Meg J. Jardine, Bruce Neal, Severine Bompoint, Hiddo J.L. Heerspink, David M. Charytan, Robert Edwards, et al. 2019. “Canagliflozin and Renal Outcomes in Type 2 Diabetes and Nephropathy.” *New England Journal of Medicine* 380 (24): 2295–2306. https://doi.org/10.1056/nejmoa1811744.

Pitt, Bertram, Gerasimos Filippatos, Rajiv Agarwal, Stefan D. Anker, George L. Bakris, Peter Rossing, Amer Joseph, et al. 2021. “Cardiovascular Events with Finerenone in Kidney Disease and Type 2 Diabetes.” *New England Journal of Medicine* 385 (24): 2252–63. https://doi.org/10.1056/nejmoa2110956.

Pollock, Carol, Bergur Stefánsson, Daniel Reyner, Peter Rossing, C. David Sjöström, David C. Wheeler, Anna Maria Langkilde, and Hiddo J.L. Heerspink. 2019. “Albuminuria-Lowering Effect of Dapagliflozin Alone and in Combination with Saxagliptin and Effect of Dapagliflozin and Saxagliptin on Glycaemic Control in Patients with Type 2 Diabetes and Chronic Kidney Disease (DELIGHT): A Randomised, Double-Blind, Placebo-Controlled Trial.” *The Lancet Diabetes and Endocrinology* 7 (6): 429–41. https://doi.org/10.1016/S2213-8587(19)30086-5.

Rosas, Sylvia E., Luis M. Ruilope, Stefan D. Anker, Bertram Pitt, Peter Rossing, Andres Angelo Cadena Bonfanti, Ricardo Correa-Rotter, et al. 2023. “Finerenone in Hispanic Patients With CKD and Type 2 Diabetes: A Post Hoc FIDELITY Analysis.” *Kidney Medicine* 5 (10). https://doi.org/10.1016/j.xkme.2023.100704.

Salanti, Georgia, Cinzia Del Giovane, Anna Chaimani, Deborah M. Caldwell, and Julian P. T. Higgins. 2014. ‘Evaluating the Quality of Evidence from a Network Meta-Analysis’. PLoS ONE 9 (7): e99682. https://doi.org/10.1371/journal.pone.0099682.

Sarafidis, Pantelis, Rajiv Agarwal, Bertram Pitt, Christoph Wanner, Gerasimos Filippatos, John Boletis, Katherine R. Tuttle, et al. 2023. “Outcomes with Finerenone in Participants with Stage 4 CKD and Type 2 Diabetes A FIDELITY Subgroup Analysis.” *Clinical Journal of the American Society of Nephrology* 18 (5): 602–12. https://doi.org/10.2215/CJN.0000000000000149.

Scilletta, Sabrina, Maurizio Di Marco, Nicoletta Miano, Agnese Filippello, Stefania Di Mauro, Alessandra Scamporrino, Marco Musmeci, et al. 2023. “Update on Diabetic Kidney Disease (DKD): Focus on Non-Albuminuric DKD and Cardiovascular Risk.” *Biomolecules*. MDPI. https://doi.org/10.3390/biom13050752.

Shah, Meera, and Adrian Vella. 2014. “Effects of GLP-1 on Appetite and Weight.” *Reviews in Endocrine and Metabolic Disorders*. Kluwer Academic Publishers. https://doi.org/10.1007/s11154-014-9289-5.

Shi, Qingyang, Kailei Nong, Per Olav Vandvik, Gordon H. Guyatt, Oliver Schnell, Lars Rydén, Nikolaus Marx, et al. 2023. “Benefits and Harms of Drug Treatment for Type 2 Diabetes: Systematic Review and Network Meta-Analysis of Randomised Controlled Trials.” *BMJ*. https://doi.org/10.1136/bmj-2022-074068.

Sivalingam, Suvanjaa, Victor Soendergaard Wasehuus, Viktor Rotbain Curovic, Martin Bæk Blond, Tine W. Hansen, Frederik Persson, and Peter Rossing. 2024. “Albuminuria-Lowering Effect of Adding Semaglutide on Top of Empagliflozin in Individuals with Type 2 Diabetes: A Randomized and Placebo-Controlled Study.” *Diabetes, Obesity and Metabolism* 26 (1): 54–64. https://doi.org/10.1111/dom.15287.

Skov, Jeppe, Anders Dejgaard, Jørgen Frøkiær, Jens Juul Holst, Thomas Jonassen, Søren Rittig, and Jens Sandahl Christiansen. 2013. “Glucagon-Like Peptide-1 (GLP-1): Effect on Kidney Hemodynamics and Renin-Angiotensin-Aldosterone System in Healthy Men.” *The Journal of Clinical Endocrinology & Metabolism* 98 (4): E664–71. https://doi.org/10.1210/jc.2012-3855.

Sterne, Jonathan A C, Jelena Savović, Matthew J Page, Roy G Elbers, Natalie S Blencowe, Isabelle Boutron, Christopher J Cates, et al. 2019. “RoB 2: A Revised Tool for Assessing Risk of Bias in Randomised Trials.” *BMJ*, August, l4898. https://doi.org/10.1136/bmj.l4898.

Sun, Feng, Shanshan Wu, Shuxia Guo, Kai Yu, Zhirong Yang, Lishi Li, Yuan Zhang, Xiaochi Quan, Linong Ji, and Siyan Zhan. 2015. “Impact of GLP-1 Receptor Agonists on Blood Pressure, Heart Rate and Hypertension among Patients with Type 2 Diabetes: A Systematic Review and Network Meta-Analysis.” *Diabetes Research and Clinical Practice* 110 (1): 26–37. https://doi.org/10.1016/j.diabres.2015.07.015.

Takashima, Hiroyuki, Yoshinori Yoshida, Chinami Nagura, Tetsuya Furukawa, Ritsukou Tei, Takashi Maruyama, Noriaki Maruyama, and Masanori Abe. 2018. “Renoprotective Effects of Canagliflozin, a Sodium Glucose Cotransporter 2 Inhibitor, in Type 2 Diabetes Patients with Chronic Kidney Disease: A Randomized Open-Label Prospective Trial.” *Diabetes and Vascular Disease Research* 15 (5): 469–72. https://doi.org/10.1177/1479164118782872.

Tanrıverdi, Mustafa, Mehmet Baştemir, Hadiye Demirbakan, Alperen Ünalan, Merve Türkmen, and Gülşen Özkan Tanrıverdi. 2023. “Association of SGLT-2 Inhibitors with Bacterial Urinary Tract Infection in Type 2 Diabetes.” *BMC Endocrine Disorders* 23 (1): 211. https://doi.org/10.1186/s12902-023-01464-6.

Thomas, Merlin C. 2014. “Renal Effects of Dapagliflozin in Patients with Type 2 Diabetes.” *Therapeutic Advances in Endocrinology and Metabolism* 5 (3): 53–61. https://doi.org/10.1177/2042018814544153.

Tuttle, Katherine R., Adeera Levin, Masaomi Nangaku, Takashi Kadowaki, Rajiv Agarwal, Sibylle J. Hauske, Amelie Elsaßer, et al. 2022. “Safety of Empagliflozin in Patients With Type 2 Diabetes and Chronic Kidney Disease: Pooled Analysis of Placebo-Controlled Clinical Trials.” *Diabetes Care* 45 (6): 1445–52. https://doi.org/10.2337/dc21-2034.

Vallon, Volker, and Scott C. Thomson. 2017. “Targeting Renal Glucose Reabsorption to Treat Hyperglycaemia: The Pleiotropic Effects of SGLT2 Inhibition.” *Diabetologia* 60 (2): 215–25. https://doi.org/10.1007/s00125-016-4157-3.

Wada, Takashi, Kazumi Mori-Anai, Yutaka Kawaguchi, Hideyuki Katsumata, Hidetaka Tsuda, Mitsutaka Iida, Kenji Arakawa, and Meg J. Jardine. 2022. “Renal, Cardiovascular and Safety Outcomes of Canagliflozin in Patients with Type 2 Diabetes and Nephropathy in East and South-East Asian Countries: Results from the Canagliflozin and Renal Events in Diabetes with Established Nephropathy Clinical Evaluation Trial.” *Journal of Diabetes Investigation* 13 (1): 54–64. https://doi.org/10.1111/jdi.13624.

Wada, Takashi, Kazumi Mori-Anai, Akiko Takahashi, Takahiro Matsui, Masaya Inagaki, Mitsutaka Iida, Ken Maruyama, and Hidetaka Tsuda. 2022. “Effect of Canagliflozin on the Decline of Estimated Glomerular Filtration Rate in Chronic Kidney Disease Patients with Type 2 Diabetes Mellitus: A Multicenter, Randomized, Double-Blind, Placebo-Controlled, Parallel-Group, Phase III Study in Japan.” *Journal of Diabetes Investigation* 13 (12): 1981–89. https://doi.org/10.1111/jdi.13888.

Wanner, Christoph. 2017. “EMPA-REG OUTCOME: The Nephrologist’s Point of View.” *The American Journal of Cardiology* 120 (1): S59–67. https://doi.org/10.1016/j.amjcard.2017.05.012.

Wanner, Christoph, John M. Lachin, Silvio E. Inzucchi, David Fitchett, Michaela Mattheus, Jyothis George, Hans J. Woerle, Uli C. Broedl, Maximilian Von Eynatten, and Bernard Zinman. 2018. “Empagliflozin and Clinical Outcomes in Patients with Type 2 Diabetes Mellitus, Established Cardiovascular Disease, and Chronic Kidney Disease.” *Circulation* 137 (2): 119–29. https://doi.org/10.1161/CIRCULATIONAHA.117.028268.

Weizhen Gao. 2022. “To observe the clinical effect of liraglutide in the treatment of type 2 diabetes mellitus with mild to moderate chronic kidney disease,” September, 162–64.

Wish, Jay B., and Pablo Pergola. 2022. ‘Evolution of Mineralocorticoid Receptor Antagonists in the Treatment of Chronic Kidney Disease Associated with Type 2 Diabetes Mellitus’. Mayo Clinic Proceedings: Innovations, Quality & Outcomes 6 (6): 536–51. https://doi.org/10.1016/j.mayocpiqo.2022.09.002.

Woods, T. Cooper, Ryousuke Satou, Kayoko Miyata, Akemi Katsurada, Courtney M. Dugas, Natasha C. Klingenberg, Vivian A. Fonseca, and L. Gabriel Navar. 2019. ‘Canagliflozin Prevents Intrarenal Angiotensinogen Augmentation and Mitigates Kidney Injury and Hypertension in Mouse Model of Type 2 Diabetes Mellitus’. American Journal of Nephrology 49 (4): 331–42. https://doi.org/10.1159/000499597.

Yale, J. F., G. Bakris, B. Cariou, D. Yue, E. David-Neto, L. Xi, K. Figueroa, E. Wajs, K. Usiskin, and G. Meininger. 2013. “Efficacy and Safety of Canagliflozin in Subjects with Type 2 Diabetes and Chronic Kidney Disease.” *Diabetes, Obesity and Metabolism* 15 (5): 463–73. https://doi.org/10.1111/dom.12090.

Yamada, Takayuki, Mako Wakabayashi, Abhinav Bhalla, Nitin Chopra, Hirotaka Miyashita, Takahisa Mikami, Hiroki Ueyama, et al. 2021. “Cardiovascular and Renal Outcomes with SGLT-2 Inhibitors versus GLP-1 Receptor Agonists in Patients with Type 2 Diabetes Mellitus and Chronic Kidney Disease: A Systematic Review and Network Meta-Analysis.” *Cardiovascular Diabetology* 20 (1): 14. https://doi.org/10.1186/s12933-020-01197-z.

Yamout, Hala, Vlado Perkovic, Melanie Davies, Vincent Woo, Dick De Zeeuw, Cristiana Mayer, Ujjwala Vijapurkar, et al. 2014. “Efficacy and Safety of Canagliflozin in Patients with Type 2 Diabetes and Stage 3 Nephropathy.” *American Journal of Nephrology* 40 (1): 64–74. https://doi.org/10.1159/000364909.

Zhang, Haitao, Jingyuan Xie, Chuanming Hao, Xuemei Li, Dalong Zhu, Hongguang Zheng, Xudong Xu, et al. 2023. “Finerenone in Patients with Chronic Kidney Disease and Type 2 Diabetes: The FIDELIO-DKD Subgroup from China.” *Kidney Diseases* 9 (6): 498–506. https://doi.org/10.1159/000531997.

Zhang, Ming-Zhu, Wujisiguleng Bao, Qi-Yan Zheng, Ya-Hui Wang, and Lu-Ying Sun. 2022. “Efficacy and Safety of Finerenone in Chronic Kidney Disease: A Systematic Review and Meta-Analysis of Randomized Clinical Trials.” *Frontiers in Pharmacology* 13 (February). https://doi.org/10.3389/fphar.2022.819327.

Zhang, Yaofu, Li Jiang, Junheng Wang, Tongxin Wang, Chieh Chien, Weijun Huang, Xiaozhe Fu, et al. 2022. “Network Meta-Analysis on the Effects of Finerenone versus SGLT2 Inhibitors and GLP-1 Receptor Agonists on Cardiovascular and Renal Outcomes in Patients with Type 2 Diabetes Mellitus and Chronic Kidney Disease.” *Cardiovascular Diabetology* 21 (1): 232. https://doi.org/10.1186/s12933-022-01676-5.

Zoja, Carlamaria, Christodoulos Xinaris, and Daniela Macconi. 2020. “Diabetic Nephropathy: Novel Molecular Mechanisms and Therapeutic Targets.” *Frontiers in Pharmacology* 11 (December). https://doi.org/10.3389/fphar.2020.586892.

Zuoping Chen. 2016. “To analyze the efficacy and safety of liraglutide in the treatment of type 2 diabetes mellitus with mild to moderate chronic kidney disease.”

Appendix 20: Grading the evidence for outcome of the network meta-analysis using CINeMA

20.1 Summary of study limitations of the included studies (HbA1c)


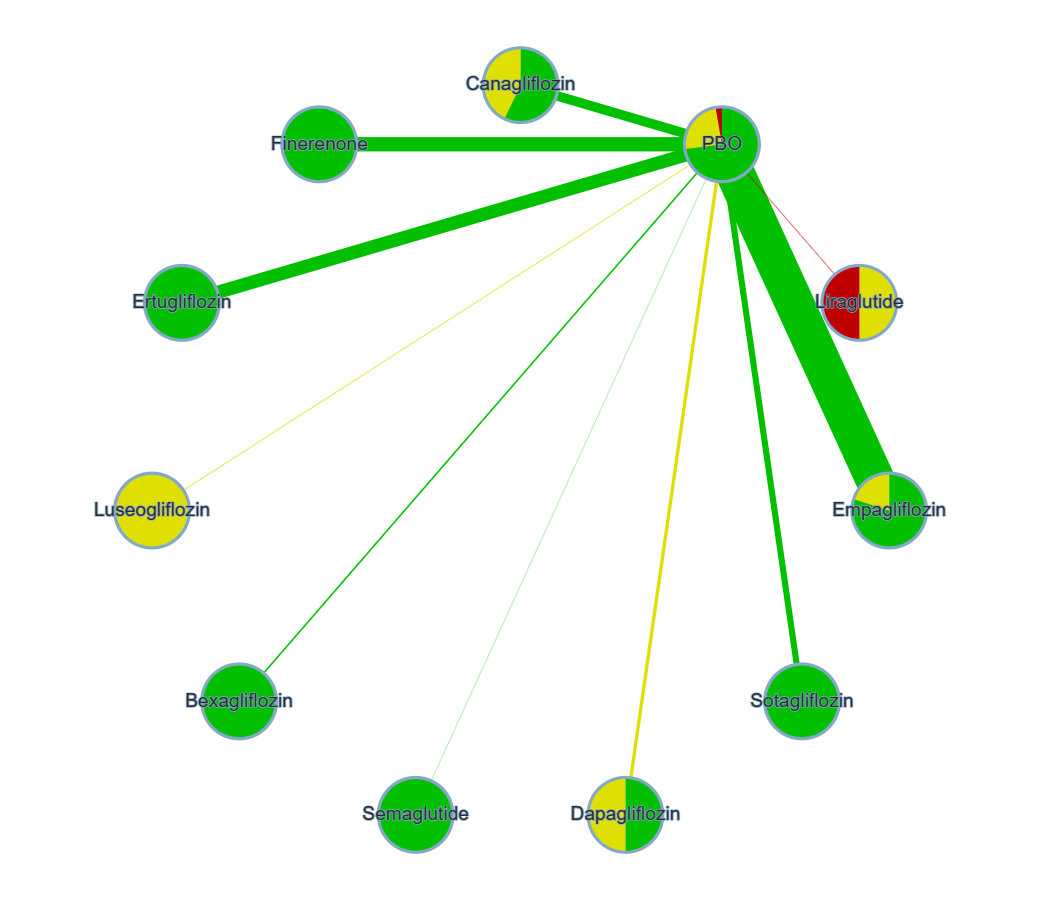


Network plot of study limitations of the included studies. Node size by equal size, node color by RoB. The colors in the circles indicate the percentage of low RoB studies (green), moderate RoB studies (yellow), high RoB studies (red) about each physical activity type. Edge width by sample size. Edge color by average RoB. The colors of the lines indicate the summative RoB assessment of each comparison. Low RoB is green, moderate RoB is yellow, high RoB is red. PBO: Placebo.

Contribution percentage of low, moderate, and high RoB comparisons to each network estimate

Low RoB is green, moderate RoB is yellow, high RoB is red.


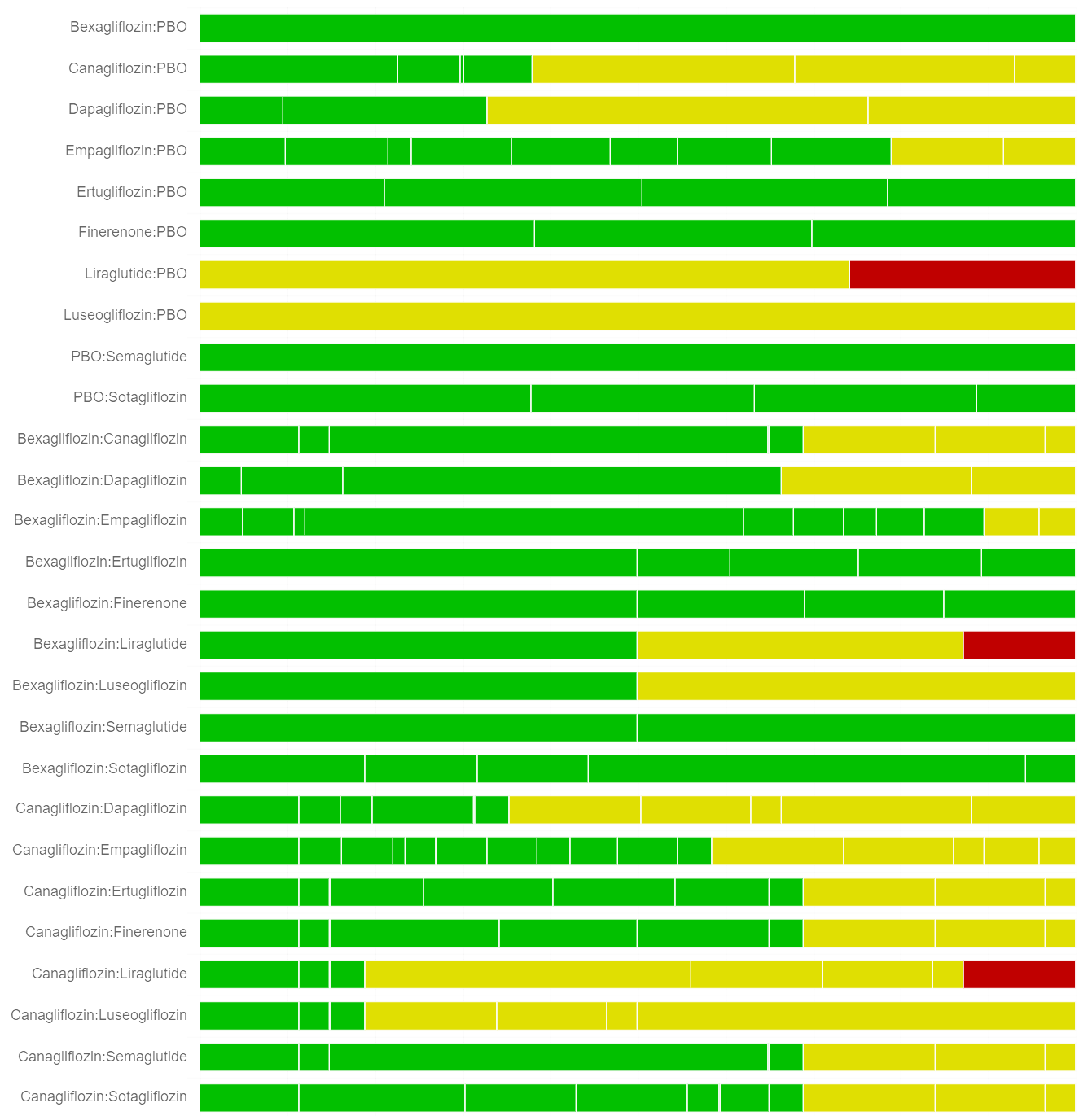


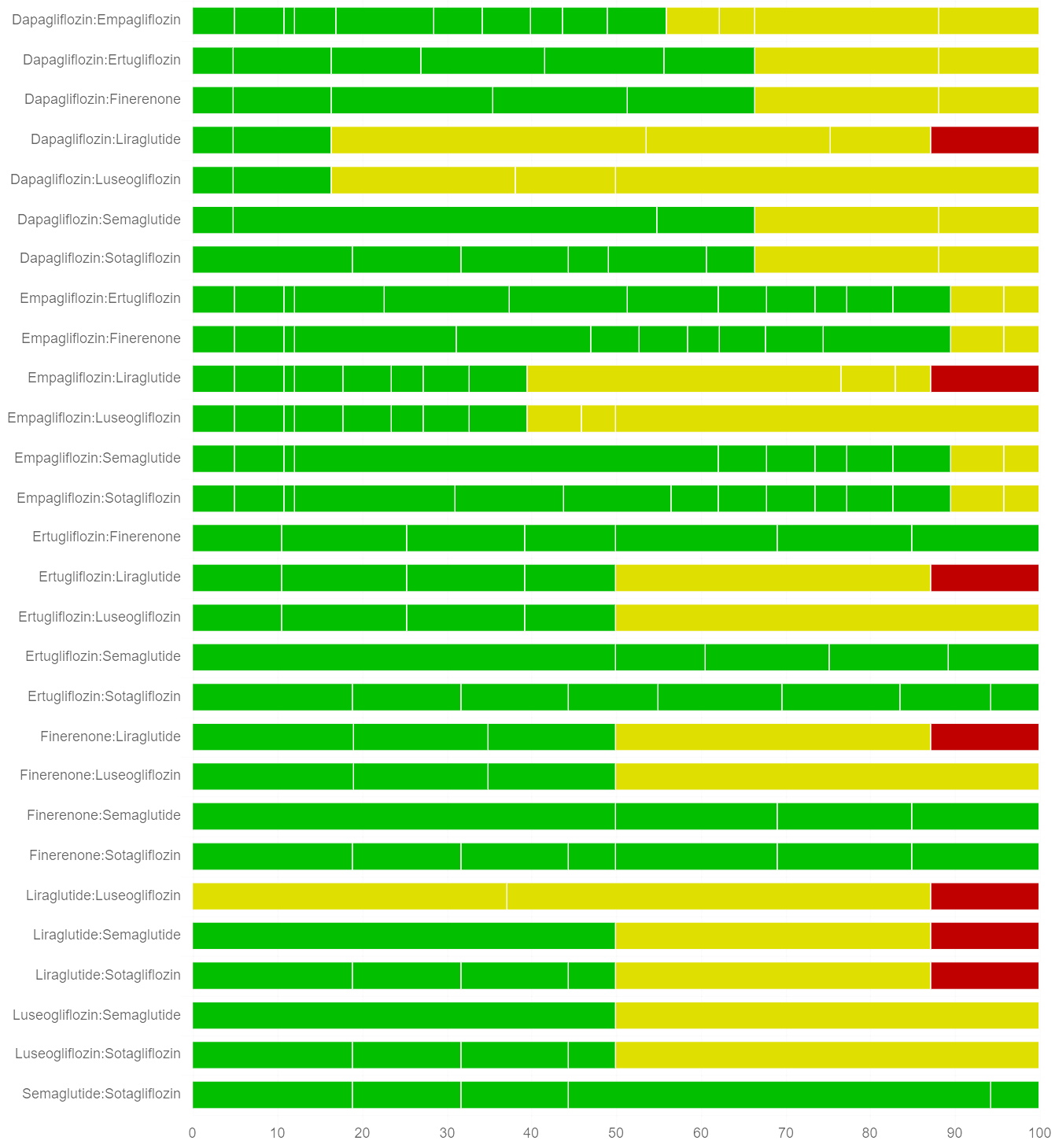


In this figure, red, yellow and green represent high, medium and low risk of bias, respectively. The proportion of red, yellow and green is based on the contribution of each study with high, medium and low risk of bias to the NMA results of the comparison group. The risk of bias in each comparison group was assessed as no concern (no downgrade), some concern (one grade downgrade), and very serious (two grades downgrade).

CINeMA for the outcome “HbA1c”

| Comparison | Number of studies | Within-study bias | Reporting bias | Indirectness | Imprecision | Heterogeneity | Incoherence | Confidence rating |
| --- | --- | --- | --- | --- | --- | --- | --- | --- |
| Bexagliflozin:PBO | 1 | No concerns | Low risk | No concerns | Major concerns | No concerns | Major concerns | Very low |
| Canagliflozin:PBO | 7 | Some concerns | Low risk | No concerns | No concerns | Major concerns | Major concerns | Very low |
| Dapagliflozin:PBO | 4 | Some concerns | Low risk | No concerns | Major concerns | No concerns | Major concerns | Very low |
| Empagliflozin:PBO | 10 | No concerns | Low risk | No concerns | No concerns | No concerns | Major concerns | Low |
| Ertugliflozin:PBO | 4 | No concerns | Low risk | No concerns | No concerns | Major concerns | Major concerns | Very low |
| Finerenone:PBO | 3 | No concerns | Low risk | No concerns | Major concerns | No concerns | Major concerns | Very low |
| Liraglutide:PBO | 2 | Some concerns | Low risk | No concerns | Major concerns | No concerns | Major concerns | Very low |
| Luseogliflozin:PBO | 1 | Some concerns | Low risk | No concerns | Major concerns | No concerns | Major concerns | Very low |
| PBO:Semaglutide | 1 | No concerns | Low risk | No concerns | Major concerns | No concerns | Major concerns | Very low |
| PBO:Sotagliflozin | 4 | No concerns | Low risk | No concerns | Major concerns | No concerns | Major concerns | Very low |
| Bexagliflozin:Canagliflozin | 0 | No concerns | Low risk | No concerns | Major concerns | No concerns | Major concerns | Very low |
| Bexagliflozin:Dapagliflozin | 0 | No concerns | Low risk | No concerns | Major concerns | No concerns | Major concerns | Very low |
| Bexagliflozin:Empagliflozin | 0 | No concerns | Low risk | No concerns | Major concerns | No concerns | Major concerns | Very low |
| Bexagliflozin:Ertugliflozin | 0 | No concerns | Low risk | No concerns | Major concerns | No concerns | Major concerns | Very low |
| Bexagliflozin:Finerenone | 0 | No concerns | Low risk | No concerns | Major concerns | No concerns | Major concerns | Very low |
| Bexagliflozin:Liraglutide | 0 | Some concerns | Low risk | No concerns | Major concerns | No concerns | Major concerns | Very low |
| Bexagliflozin:Luseogliflozin | 0 | Some concerns | Low risk | No concerns | Major concerns | No concerns | Major concerns | Very low |
| Bexagliflozin:Semaglutide | 0 | No concerns | Low risk | No concerns | Major concerns | No concerns | Major concerns | Very low |
| Bexagliflozin:Sotagliflozin | 0 | No concerns | Low risk | No concerns | Major concerns | No concerns | Major concerns | Very low |
| Canagliflozin:Dapagliflozin | 0 | Some concerns | Low risk | No concerns | Major concerns | No concerns | Major concerns | Very low |
| Canagliflozin:Empagliflozin | 0 | No concerns | Low risk | No concerns | Major concerns | No concerns | Major concerns | Very low |
| Canagliflozin:Ertugliflozin | 0 | No concerns | Low risk | No concerns | Major concerns | No concerns | Major concerns | Very low |
| Canagliflozin:Finerenone | 0 | No concerns | Low risk | No concerns | No concerns | Major concerns | Major concerns | Very low |
| Canagliflozin:Liraglutide | 0 | Some concerns | Low risk | No concerns | Major concerns | No concerns | Major concerns | Very low |
| Canagliflozin:Luseogliflozin | 0 | Some concerns | Low risk | No concerns | Major concerns | No concerns | Major concerns | Very low |
| Canagliflozin:Semaglutide | 0 | No concerns | Low risk | No concerns | Major concerns | No concerns | Major concerns | Very low |
| Canagliflozin:Sotagliflozin | 0 | No concerns | Low risk | No concerns | Major concerns | No concerns | Major concerns | Very low |
| Dapagliflozin:Empagliflozin | 0 | No concerns | Low risk | No concerns | Major concerns | No concerns | Major concerns | Very low |
| Dapagliflozin:Ertugliflozin | 0 | No concerns | Low risk | No concerns | Major concerns | No concerns | Major concerns | Very low |
| Dapagliflozin:Finerenone | 0 | No concerns | Low risk | No concerns | Major concerns | No concerns | Major concerns | Very low |
| Dapagliflozin:Liraglutide | 0 | Some concerns | Low risk | No concerns | Major concerns | No concerns | Major concerns | Very low |
| Dapagliflozin:Luseogliflozin | 0 | Some concerns | Low risk | No concerns | Major concerns | No concerns | Major concerns | Very low |
| Dapagliflozin:Semaglutide | 0 | No concerns | Low risk | No concerns | Major concerns | No concerns | Major concerns | Very low |
| Dapagliflozin:Sotagliflozin | 0 | No concerns | Low risk | No concerns | Major concerns | No concerns | Major concerns | Very low |
| Empagliflozin:Ertugliflozin | 0 | No concerns | Low risk | No concerns | Major concerns | No concerns | Major concerns | Very low |
| Empagliflozin:Finerenone | 0 | No concerns | Low risk | No concerns | No concerns | No concerns | Major concerns | Low |
| Comparison | Number of studies | Within-study bias | Reporting bias | Indirectness | Imprecision | Heterogeneity | Incoherence | Confidence rating |
| Empagliflozin:Liraglutide | 0 | Some concerns | Low risk | No concerns | Major concerns | No concerns | Major concerns | Very low |
| Empagliflozin:Luseogliflozin | 0 | Some concerns | Low risk | No concerns | Major concerns | No concerns | Major concerns | Very low |
| Empagliflozin:Semaglutide | 0 | No concerns | Low risk | No concerns | Major concerns | No concerns | Major concerns | Very low |
| Empagliflozin:Sotagliflozin | 0 | No concerns | Low risk | No concerns | Major concerns | No concerns | Major concerns | Very low |
| Ertugliflozin:Finerenone | 0 | No concerns | Low risk | No concerns | Major concerns | No concerns | Major concerns | Very low |
| Ertugliflozin:Liraglutide | 0 | Some concerns | Low risk | No concerns | Major concerns | No concerns | Major concerns | Very low |
| Ertugliflozin:Luseogliflozin | 0 | Some concerns | Low risk | No concerns | Major concerns | No concerns | Major concerns | Very low |
| Ertugliflozin:Semaglutide | 0 | No concerns | Low risk | No concerns | Major concerns | No concerns | Major concerns | Very low |
| Ertugliflozin:Sotagliflozin | 0 | No concerns | Low risk | No concerns | Major concerns | No concerns | Major concerns | Very low |
| Finerenone:Liraglutide | 0 | Some concerns | Low risk | No concerns | Major concerns | No concerns | Major concerns | Very low |
| Finerenone:Luseogliflozin | 0 | Some concerns | Low risk | No concerns | Major concerns | No concerns | Major concerns | Very low |
| Finerenone:Semaglutide | 0 | No concerns | Low risk | No concerns | Major concerns | No concerns | Major concerns | Very low |
| Finerenone:Sotagliflozin | 0 | No concerns | Low risk | No concerns | Major concerns | No concerns | Major concerns | Very low |
| Liraglutide:Luseogliflozin | 0 | Some concerns | Low risk | No concerns | Major concerns | No concerns | Major concerns | Very low |
| Liraglutide:Semaglutide | 0 | Some concerns | Low risk | No concerns | Major concerns | No concerns | Major concerns | Very low |
| Liraglutide:Sotagliflozin | 0 | Some concerns | Low risk | No concerns | Major concerns | No concerns | Major concerns | Very low |
| Luseogliflozin:Semaglutide | 0 | Some concerns | Low risk | No concerns | Major concerns | No concerns | Major concerns | Very low |
| Luseogliflozin:Sotagliflozin | 0 | Some concerns | Low risk | No concerns | Major concerns | No concerns | Major concerns | Very low |
| Semaglutide:Sotagliflozin | 0 | No concerns | Low risk | No concerns | Major concerns | No concerns | Major concerns | Very low |

PBO: Placebo.

20.2 Summary of study limitations of the included studies (eGFR)


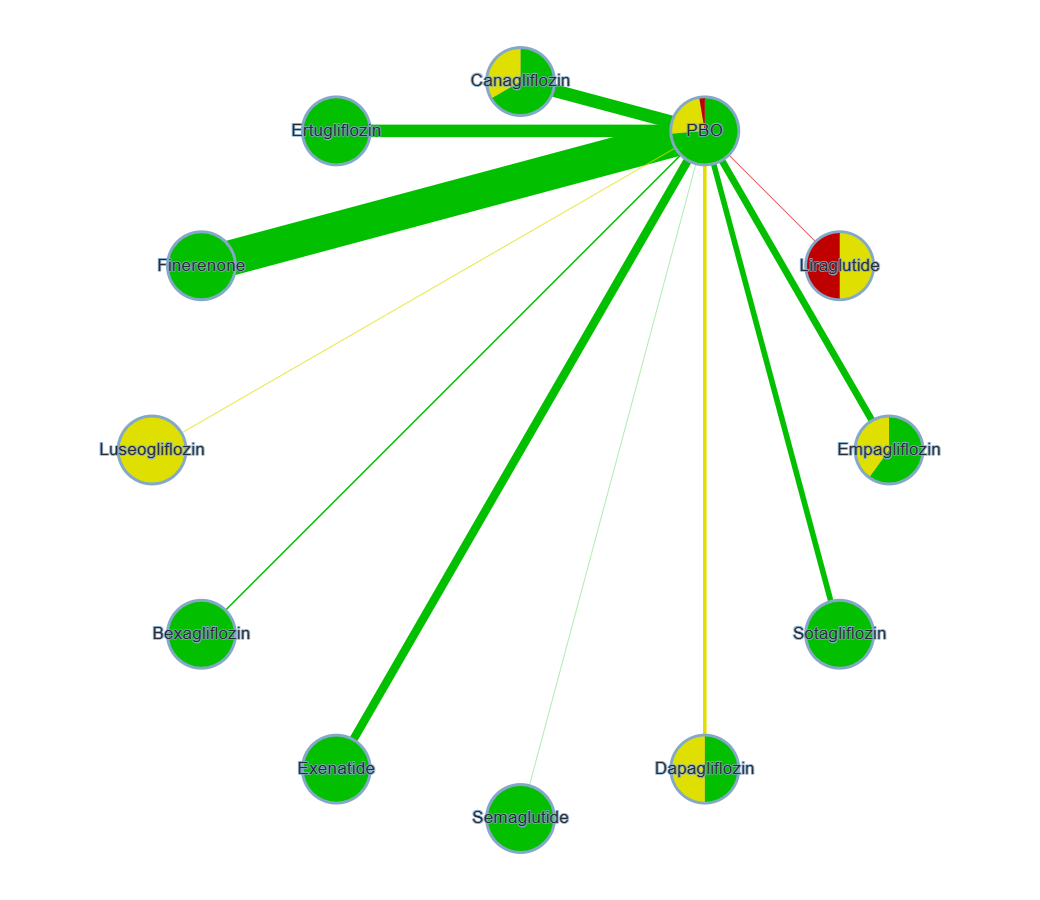


Network plot of study limitations of the included studies. Node size by equal size, node color by RoB. The colors in the circles indicate the percentage of low RoB studies (green), moderate RoB studies (yellow), high RoB studies (red) about each physical activity type. Edge width by sample size. Edge color by average RoB. The colors of the lines indicate the summative RoB assessment of each comparison. Low RoB is green, moderate RoB is yellow, high RoB is red. PBO: Placebo.

Contribution percentage of low, moderate, and high RoB comparisons to each network estimate

Low RoB is green, moderate RoB is yellow, high RoB is red.


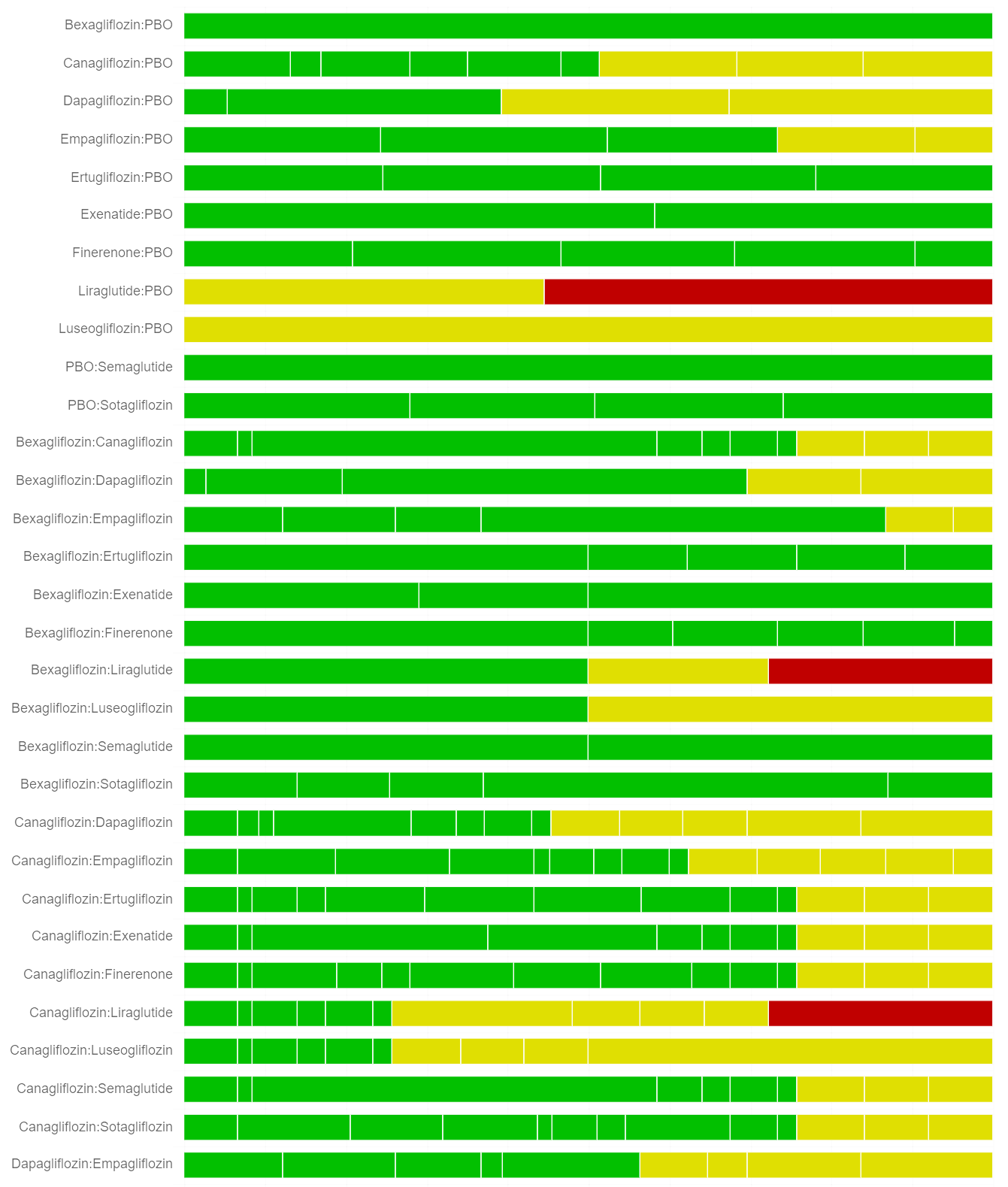


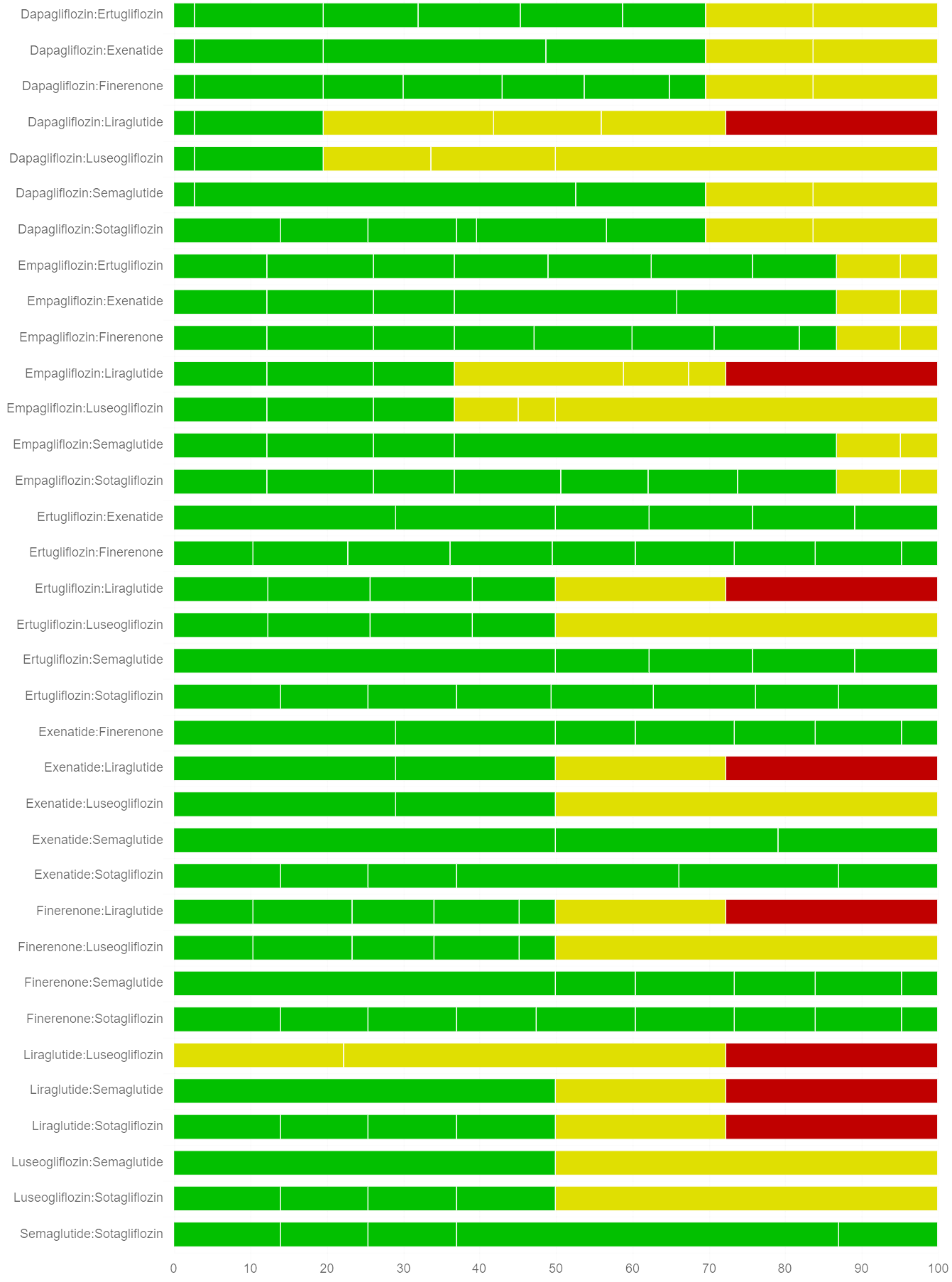


CINeMA for the outcome “eGFR”

| Comparison | Number of studies | Within-study bias | Reporting bias | Indirectness | Imprecision | Heterogeneity | Incoherence | Confidence rating |
| --- | --- | --- | --- | --- | --- | --- | --- | --- |
| Bexa:PBO | 1 | No concerns | Low risk | No concerns | Major concerns | No concerns | Major concerns | Very low |
| Canagliflozin:PBO | 9 | No concerns | Low risk | No concerns | Major concerns | No concerns | Major concerns | Very low |
| Dapagliflozin:PBO | 4 | Some concerns | Low risk | No concerns | Major concerns | No concerns | Major concerns | Very low |
| Empagliflozin:PBO | 5 | No concerns | Low risk | No concerns | Major concerns | No concerns | Major concerns | Very low |
| Ertugliflozin:PBO | 4 | No concerns | Low risk | No concerns | Major concerns | No concerns | Major concerns | Very low |
| Exenatide:PBO | 2 | No concerns | Low risk | No concerns | Major concerns | No concerns | Major concerns | Very low |
| Finerenone:PBO | 5 | No concerns | Low risk | No concerns | Major concerns | No concerns | Major concerns | Very low |
| Liraglutide:PBO | 2 | Major concerns | Low risk | No concerns | No concerns | Major concerns | Major concerns | Very low |
| Luseogliflozin:PBO | 1 | Some concerns | Low risk | No concerns | Major concerns | No concerns | Major concerns | Very low |
| PBO:Semaglutide | 1 | No concerns | Low risk | No concerns | Major concerns | No concerns | Major concerns | Very low |
| PBO:Sotagliflozin | 4 | No concerns | Low risk | No concerns | Major concerns | No concerns | Major concerns | Very low |
| Bexagliflozin:Canagliflozin | 0 | No concerns | Low risk | No concerns | Major concerns | No concerns | Major concerns | Very low |
| Bexagliflozin:Dapagliflozin | 0 | No concerns | Low risk | No concerns | Major concerns | No concerns | Major concerns | Very low |
| Bexagliflozin:Empagliflozin | 0 | No concerns | Low risk | No concerns | Major concerns | No concerns | Major concerns | Very low |
| Bexagliflozin:Ertugliflozin | 0 | No concerns | Low risk | No concerns | Major concerns | No concerns | Major concerns | Very low |
| Bexagliflozin:Exenatide | 0 | No concerns | Low risk | No concerns | Major concerns | No concerns | Major concerns | Very low |
| Bexagliflozin:Finerenone | 0 | No concerns | Low risk | No concerns | Major concerns | No concerns | Major concerns | Very low |
| Bexagliflozin:Liraglutide | 0 | Some concerns | Low risk | No concerns | No concerns | Major concerns | Major concerns | Very low |
| Bexagliflozin:Luseogliflozin | 0 | Some concerns | Low risk | No concerns | Major concerns | No concerns | Major concerns | Very low |
| Bexagliflozin:Semaglutide | 0 | No concerns | Low risk | No concerns | Major concerns | No concerns | Major concerns | Very low |
| Bexagliflozin:Sotagliflozin | 0 | No concerns | Low risk | No concerns | Major concerns | No concerns | Major concerns | Very low |
| Canagliflozin:Dapagliflozin | 0 | Some concerns | Low risk | No concerns | Major concerns | No concerns | Major concerns | Very low |
| Canagliflozin:Empagliflozin | 0 | No concerns | Low risk | No concerns | Major concerns | No concerns | Major concerns | Very low |
| Canagliflozin:Ertugliflozin | 0 | No concerns | Low risk | No concerns | Major concerns | No concerns | Major concerns | Very low |
| Canagliflozin:Exenatide | 0 | No concerns | Low risk | No concerns | Major concerns | No concerns | Major concerns | Very low |
| Canagliflozin:Finerenone | 0 | No concerns | Low risk | No concerns | Major concerns | No concerns | Major concerns | Very low |
| Canagliflozin:Liraglutide | 0 | Some concerns | Low risk | No concerns | No concerns | Major concerns | Major concerns | Very low |
| Canagliflozin:Luseogliflozin | 0 | Some concerns | Low risk | No concerns | Major concerns | No concerns | Major concerns | Very low |
| Canagliflozin:Semaglutide | 0 | No concerns | Low risk | No concerns | Major concerns | No concerns | Major concerns | Very low |
| Canagliflozin:Sotagliflozin | 0 | No concerns | Low risk | No concerns | Major concerns | No concerns | Major concerns | Very low |
| Dapagliflozin:Empagliflozin | 0 | No concerns | Low risk | No concerns | Major concerns | No concerns | Major concerns | Very low |
| Dapagliflozin:Ertugliflozin | 0 | No concerns | Low risk | No concerns | Major concerns | No concerns | Major concerns | Very low |
| Dapagliflozin:Exenatide | 0 | No concerns | Low risk | No concerns | Major concerns | No concerns | Major concerns | Very low |
| Dapagliflozin:Finerenone | 0 | No concerns | Low risk | No concerns | Major concerns | No concerns | Major concerns | Very low |
| Comparison | Number of studies | Within-study bias | Reporting bias | Indirectness | Imprecision | Heterogeneity | Incoherence | Confidence rating |
| Dapagliflozin:Liraglutide | 0 | Some concerns | Low risk | No concerns | No concerns | No concerns | Major concerns | Very low |
| Dapagliflozin:Luseogliflozin | 0 | Some concerns | Low risk | No concerns | Major concerns | No concerns | Major concerns | Very low |
| Dapagliflozin:Semaglutide | 0 | No concerns | Low risk | No concerns | Major concerns | No concerns | Major concerns | Very low |
| Dapagliflozin:Sotagliflozin | 0 | No concerns | Low risk | No concerns | Major concerns | No concerns | Major concerns | Very low |
| Empagliflozin:Ertugliflozin | 0 | No concerns | Low risk | No concerns | No concerns | Major concerns | Major concerns | Very low |
| Empagliflozin:Exenatide | 0 | No concerns | Low risk | No concerns | Major concerns | No concerns | Major concerns | Very low |
| Empagliflozin:Finerenone | 0 | No concerns | Low risk | No concerns | Major concerns | No concerns | Major concerns | Very low |
| Empagliflozin:Liraglutide | 0 | Some concerns | Low risk | No concerns | Major concerns | No concerns | Major concerns | Very low |
| Empagliflozin:Luseogliflozin | 0 | Some concerns | Low risk | No concerns | Major concerns | No concerns | Major concerns | Very low |
| Empagliflozin:Semaglutide | 0 | No concerns | Low risk | No concerns | Major concerns | No concerns | Major concerns | Very low |
| Empagliflozin:Sotagliflozin | 0 | No concerns | Low risk | No concerns | Major concerns | No concerns | Major concerns | Very low |
| Ertugliflozin:Exenatide | 0 | No concerns | Low risk | No concerns | Major concerns | No concerns | Major concerns | Very low |
| Ertugliflozin:Finerenone | 0 | No concerns | Low risk | No concerns | Major concerns | No concerns | Major concerns | Very low |
| Ertugliflozin:Liraglutide | 0 | Some concerns | Low risk | No concerns | No concerns | No concerns | Major concerns | Very low |
| Ertugliflozin:Luseogliflozin | 0 | Some concerns | Low risk | No concerns | Major concerns | No concerns | Major concerns | Very low |
| Ertugliflozin:Semaglutide | 0 | No concerns | Low risk | No concerns | Major concerns | No concerns | Major concerns | Very low |
| Ertugliflozin:Sotagliflozin | 0 | No concerns | Low risk | No concerns | Major concerns | No concerns | Major concerns | Very low |
| Exenatide:Finerenone | 0 | No concerns | Low risk | No concerns | Major concerns | No concerns | Major concerns | Very low |
| Exenatide:Liraglutide | 0 | Some concerns | Low risk | No concerns | Major concerns | No concerns | Major concerns | Very low |
| Exenatide:Luseogliflozin | 0 | Some concerns | Low risk | No concerns | Major concerns | No concerns | Major concerns | Very low |
| Exenatide:Semaglutide | 0 | No concerns | Low risk | No concerns | Major concerns | No concerns | Major concerns | Very low |
| Exenatide:Sotagliflozin | 0 | No concerns | Low risk | No concerns | Major concerns | No concerns | Major concerns | Very low |
| Finerenone:Liraglutide | 0 | Some concerns | Low risk | No concerns | No concerns | Major concerns | Major concerns | Very low |
| Finerenone:Luseogliflozin | 0 | Some concerns | Low risk | No concerns | Major concerns | No concerns | Major concerns | Very low |
| Finerenone:Semaglutide | 0 | No concerns | Low risk | No concerns | Major concerns | No concerns | Major concerns | Very low |
| Finerenone:Sotagliflozin | 0 | No concerns | Low risk | No concerns | Major concerns | No concerns | Major concerns | Very low |
| Liraglutide:Luseogliflozin | 0 | Some concerns | Low risk | No concerns | No concerns | Major concerns | Major concerns | Very low |
| Liraglutide:Semaglutide | 0 | Some concerns | Low risk | No concerns | Major concerns | No concerns | Major concerns | Very low |
| Liraglutide:Sotagliflozin | 0 | Some concerns | Low risk | No concerns | No concerns | Major concerns | Major concerns | Very low |
| Luseogliflozin:Semaglutide | 0 | Some concerns | Low risk | No concerns | Major concerns | No concerns | Major concerns | Very low |
| Luseogliflozin:Sotagliflozin | 0 | No concerns | Low risk | No concerns | Major concerns | No concerns | Major concerns | Very low |
| Semaglutide:Sotagliflozin | 0 | No concerns | Low risk | No concerns | Major concerns | No concerns | Major concerns | Very low |

PBO: Placebo.

20.3 Summary of study limitations of the included studies (LDL-C)


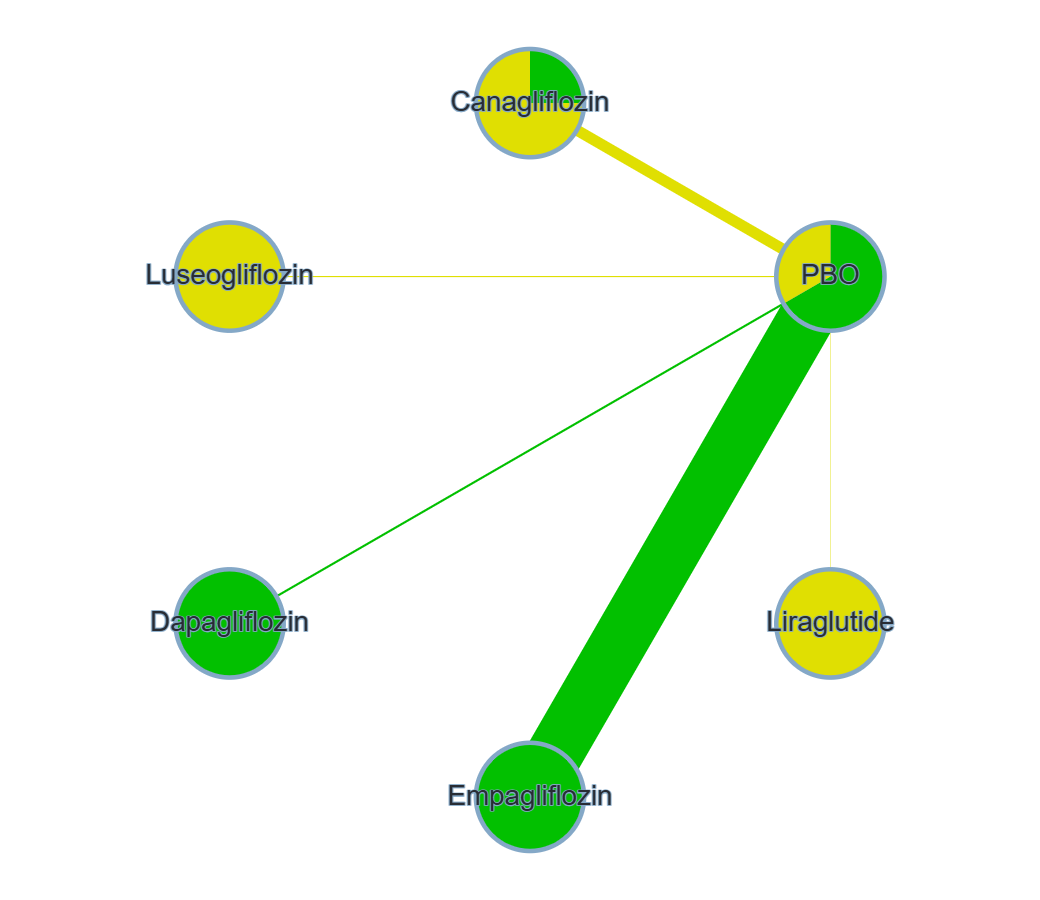


Network plot of study limitations of the included studies. Node size by equal size, node color by RoB. The colors in the circles indicate the percentage of low RoB studies (green), moderate RoB studies (yellow), high RoB studies (red) about each physical activity type. Edge width by sample size. Edge color by average RoB. The colors of the lines indicate the summative RoB assessment of each comparison. Low RoB is green, moderate RoB is yellow, high RoB is red. PBO: Placebo.

Contribution percentage of low, moderate, and high RoB comparisons to each network estimate

Low RoB is green, moderate RoB is yellow, high RoB is red.


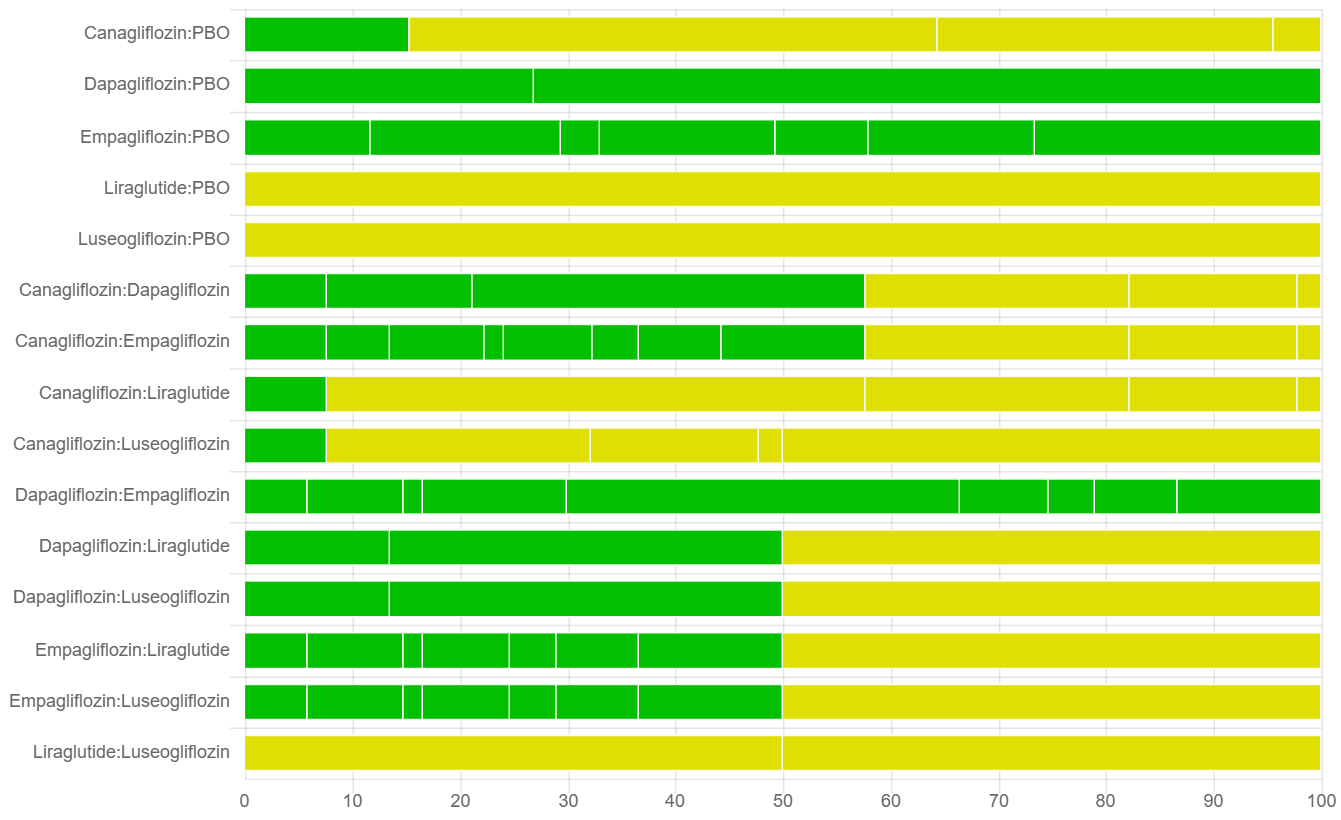


CINeMA for the outcome “LDL-C”

| Comparison | Number of studies | Within-study bias | Reporting bias | Indirectness | Imprecision | Heterogeneity | Incoherence | Confidence rating |
| --- | --- | --- | --- | --- | --- | --- | --- | --- |
| Canagliflozin:PBO | 4 | Some concerns | Low risk | No concerns | Major concerns | No concerns | Major concerns | Very low |
| Dapagliflozin:PBO | 2 | No concerns | Low risk | No concerns | Major concerns | No concerns | Major concerns | Very low |
| Empagliflozin:PBO | 7 | No concerns | Low risk | No concerns | Major concerns | No concerns | Major concerns | Very low |
| Liraglutide:PBO | 1 | Some concerns | Low risk | No concerns | No concerns | No concerns | Major concerns | Very low |
| Luseogliflozin:PBO | 1 | Some concerns | Low risk | No concerns | Major concerns | No concerns | Major concerns | Very low |
| Canagliflozin:Dapagliflozin | 0 | No concerns | Low risk | No concerns | Major concerns | No concerns | Major concerns | Very low |
| Canagliflozin:Empagliflozin | 0 | No concerns | Low risk | No concerns | Major concerns | No concerns | Major concerns | Very low |
| Canagliflozin:Liraglutide | 0 | Some concerns | Low risk | No concerns | No concerns | No concerns | Major concerns | Very low |
| Canagliflozin:Luseogliflozin | 0 | Some concerns | Low risk | No concerns | Major concerns | No concerns | Major concerns | Very low |
| Dapagliflozin:Empagliflozin | 0 | No concerns | Low risk | No concerns | Major concerns | No concerns | Major concerns | Very low |
| Dapagliflozin:Liraglutide | 0 | Some concerns | Low risk | No concerns | No concerns | No concerns | Major concerns | Very low |
| Dapagliflozin:Luseogliflozin | 0 | Some concerns | Low risk | No concerns | Major concerns | No concerns | Major concerns | Very low |
| Empagliflozin:Liraglutide | 0 | No concerns | Low risk | No concerns | No concerns | No concerns | Major concerns | Low |
| Empagliflozin:Luseogliflozin | 0 | No concerns | Low risk | No concerns | Major concerns | No concerns | Major concerns | Very low |
| Liraglutide:Luseogliflozin | 0 | Some concerns | Low risk | No concerns | No concerns | No concerns | Major concerns | Very low |

20.4 Summary of study limitations of the included studies (SBP)


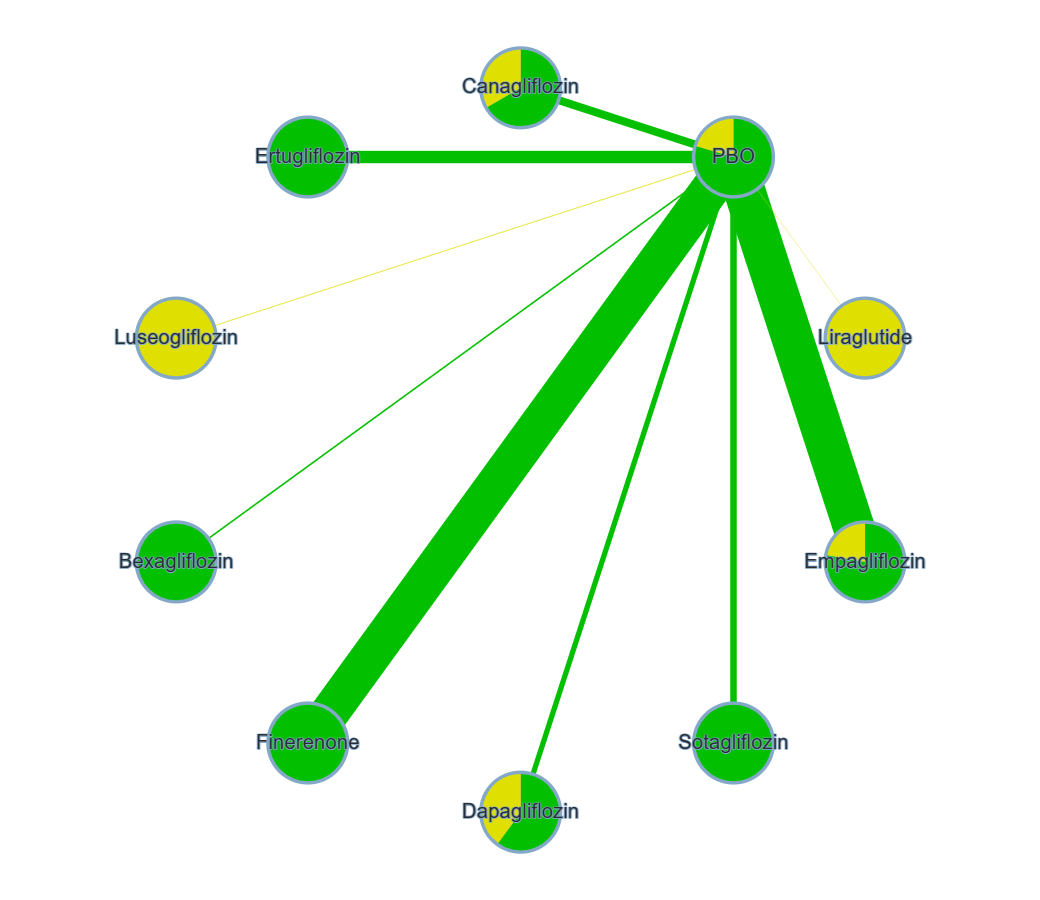


Network plot of study limitations of the included studies. Node size by equal size, node color by RoB. The colors in the circles indicate the percentage of low RoB studies (green), moderate RoB studies (yellow), high RoB studies (red) about each physical activity type. Edge width by sample size. Edge color by average RoB. The colors of the lines indicate the summative RoB assessment of each comparison. Low RoB is green, moderate RoB is yellow, high RoB is red. PBO: Placebo.

Contribution percentage of low, moderate, and high RoB comparisons to each network estimate

Low RoB is green, moderate RoB is yellow, high RoB is red.


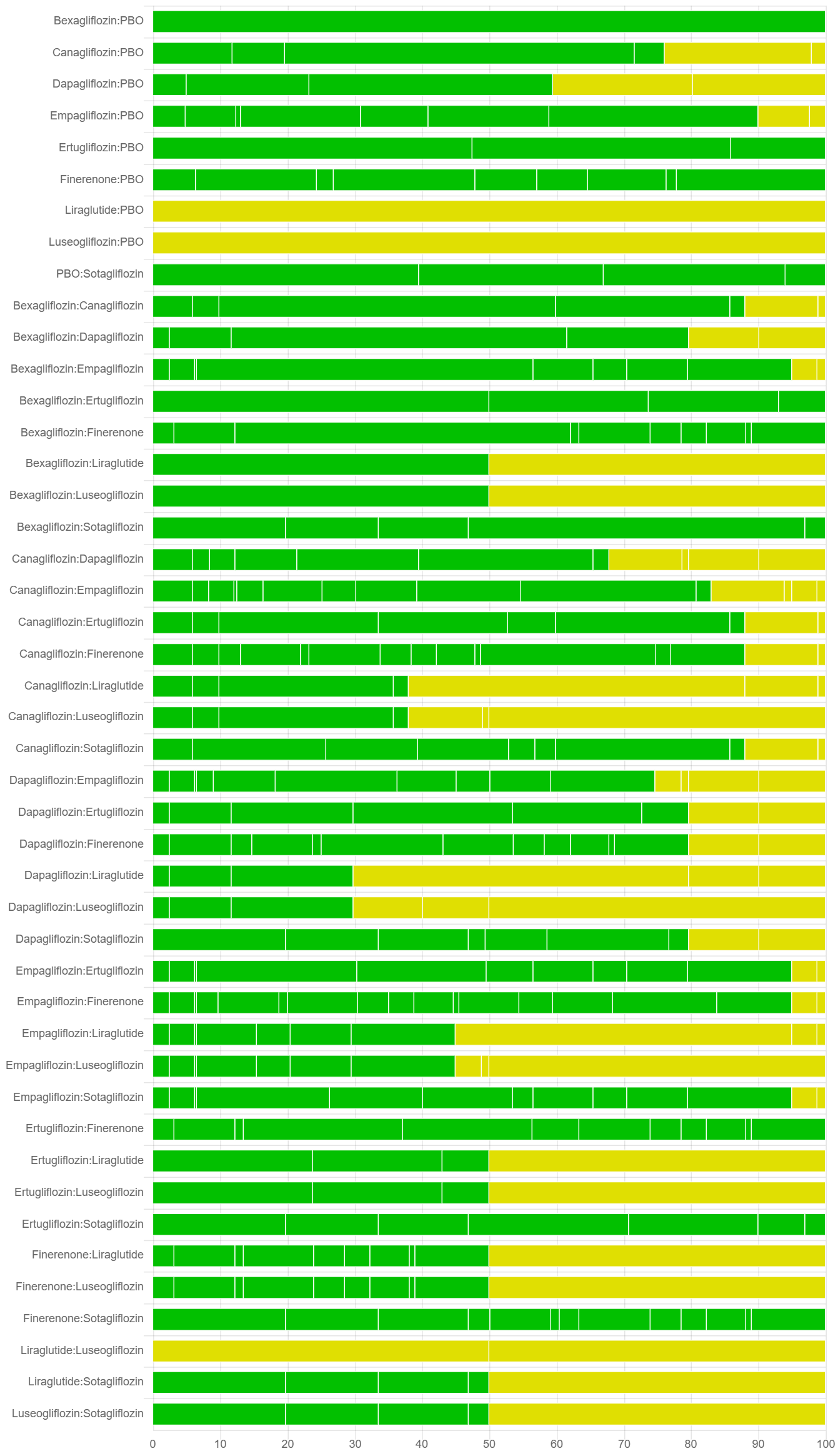


CINeMA for the outcome “SBP”

| Comparison | Number of studies | Within-study bias | Reporting bias | Indirectness | Imprecision | Heterogeneity | Incoherence | Confidence rating |
| --- | --- | --- | --- | --- | --- | --- | --- | --- |
| Bexagliflozin:  PBO | 1 | No concerns | Low risk | No concerns | No concerns | No concerns | Major concerns | Low |
| Canagliflozin:  PBO | 6 | No concerns | Low risk | No concerns | No concerns | No concerns | Major concerns | Low |
| Dapagliflozin:  PBO | 5 | No concerns | Low risk | No concerns | No concerns | No concerns | Major concerns | Low |
| Empagliflozin:  PBO | 9 | No concerns | Low risk | No concerns | No concerns | No concerns | Major concerns | Low |
| Ertugliflozin:  PBO | 3 | No concerns | Low risk | No concerns | No concerns | Major concerns | Major concerns | Very low |
| Finerenone:  PBO | 9 | No concerns | Low risk | No concerns | No concerns | No concerns | Major concerns | Low |
| Liraglutide:  PBO | 1 | Some concerns | Low risk | No concerns | Major concerns | No concerns | Major concerns | Very low |
| Luseogliflozin:PBO | 1 | Some concerns | Low risk | No concerns | Major concerns | No concerns | Major concerns | Very low |
| PBO:  Sotagliflozin | 4 | No concerns | Low risk | No concerns | Major concerns | No concerns | Major concerns | Very low |
| Bexagliflozin:  Canagliflozin | 0 | No concerns | Low risk | No concerns | Major concerns | No concerns | Major concerns | Very low |
| Bexagliflozin:  Dapagliflozin | 0 | No concerns | Low risk | No concerns | Major concerns | No concerns | Major concerns | Very low |
| Bexagliflozin:  Empagliflozin | 0 | No concerns | Low risk | No concerns | Major concerns | No concerns | Major concerns | Very low |
| Bexagliflozin:  Ertugliflozin | 0 | No concerns | Low risk | No concerns | No concerns | Major concerns | Major concerns | Very low |
| Bexagliflozin:  Finerenone | 0 | No concerns | Low risk | No concerns | No concerns | Major concerns | Major concerns | Very low |
| Bexagliflozin:  Liraglutide | 0 | Some concerns | Low risk | No concerns | Major concerns | No concerns | Major concerns | Very low |
| Bexagliflozin:  Luseogliflozin | 0 | Some concerns | Low risk | No concerns | Major concerns | No concerns | Major concerns | Very low |
| Bexagliflozin:  Sotagliflozin | 0 | No concerns | Low risk | No concerns | No concerns | No concerns | Major concerns | Low |
| Canagliflozin:  Dapagliflozin | 0 | No concerns | Low risk | No concerns | Major concerns | No concerns | Major concerns | Very low |
| Canagliflozin:  Empagliflozin | 0 | No concerns | Low risk | No concerns | Major concerns | No concerns | Major concerns | Very low |
| Canagliflozin:  Ertugliflozin | 0 | No concerns | Low risk | No concerns | Major concerns | No concerns | Major concerns | Very low |
| Canagliflozin:  Finerenone | 0 | No concerns | Low risk | No concerns | Major concerns | No concerns | Major concerns | Very low |
| Canagliflozin:  Liraglutide | 0 | Some concerns | Low risk | No concerns | Major concerns | No concerns | Major concerns | Very low |
| Canagliflozin:  Luseogliflozin | 0 | Some concerns | Low risk | No concerns | Major concerns | No concerns | Major concerns | Very low |
| Canagliflozin:  Sotagliflozin | 0 | No concerns | Low risk | No concerns | Major concerns | No concerns | Major concerns | Very low |
| Dapagliflozin:  Empagliflozin | 0 | No concerns | Low risk | No concerns | Major concerns | No concerns | Major concerns | Very low |
| Dapagliflozin:  Ertugliflozin | 0 | No concerns | Low risk | No concerns | Major concerns | No concerns | Major concerns | Very low |
| Dapagliflozin:  Finerenone | 0 | No concerns | Low risk | No concerns | Major concerns | No concerns | Major concerns | Very low |
| Dapagliflozin:  Liraglutide | 0 | Some concerns | Low risk | No concerns | Major concerns | No concerns | Major concerns | Very low |
| Dapagliflozin:  Luseogliflozin | 0 | Some concerns | Low risk | No concerns | Major concerns | No concerns | Major concerns | Very low |
| Dapagliflozin:  Sotagliflozin | 0 | No concerns | Low risk | No concerns | Major concerns | No concerns | Major concerns | Very low |
| Empagliflozin:  Ertugliflozin | 0 | No concerns | Low risk | No concerns | No concerns | No concerns | Major concerns | Low |
| Empagliflozin:  Finerenone | 0 | No concerns | Low risk | No concerns | No concerns | No concerns | Major concerns | Low |
| Empagliflozin:  Liraglutide | 0 | Some concerns | Low risk | No concerns | Major concerns | No concerns | Major concerns | Very low |
| Empagliflozin:  Luseogliflozin | 0 | Some concerns | Low risk | No concerns | Major concerns | No concerns | Major concerns | Very low |
| Empagliflozin:  Sotagliflozin | 0 | No concerns | Low risk | No concerns | No concerns | No concerns | Major concerns | Low |
| Comparison | Number of studies | Within-study bias | Reporting bias | Indirectness | Imprecision | Heterogeneity | Incoherence | Confidence rating |
|  |  |  |  |  |  |  |  |  |
| Ertugliflozin:  Finerenone | 0 | No concerns | Low risk | No concerns | Major concerns | No concerns | Major concerns | Very low |
| Ertugliflozin:  Liraglutide | 0 | Some concerns | Low risk | No concerns | Major concerns | No concerns | Major concerns | Very low |
| Ertugliflozin:  Luseogliflozin | 0 | Some concerns | Low risk | No concerns | Major concerns | No concerns | Major concerns | Very low |
| Ertugliflozin:  Sotagliflozin | 0 | No concerns | Low risk | No concerns | Major concerns | No concerns | Major concerns | Very low |
| Finerenone:  Liraglutide | 0 | Some concerns | Low risk | No concerns | Major concerns | No concerns | Major concerns | Very low |
| Finerenone:  Luseogliflozin | 0 | Some concerns | Low risk | No concerns | Major concerns | No concerns | Major concerns | Very low |
| Finerenone:  Sotagliflozin | 0 | No concerns | Low risk | No concerns | Major concerns | No concerns | Major concerns | Very low |
| Liraglutide:  Luseogliflozin | 0 | Some concerns | Low risk | No concerns | Major concerns | No concerns | Major concerns | Very low |
| Liraglutide:  Sotagliflozin | 0 | Some concerns | Low risk | No concerns | Major concerns | No concerns | Major concerns | Very low |
| Luseogliflozin:  Sotagliflozin | 0 | Some concerns | Low risk | No concerns | Major concerns | No concerns | Major concerns | Very low |

PBO: Placebo.20.5 Summary of study limitations of the included studies (DBP)


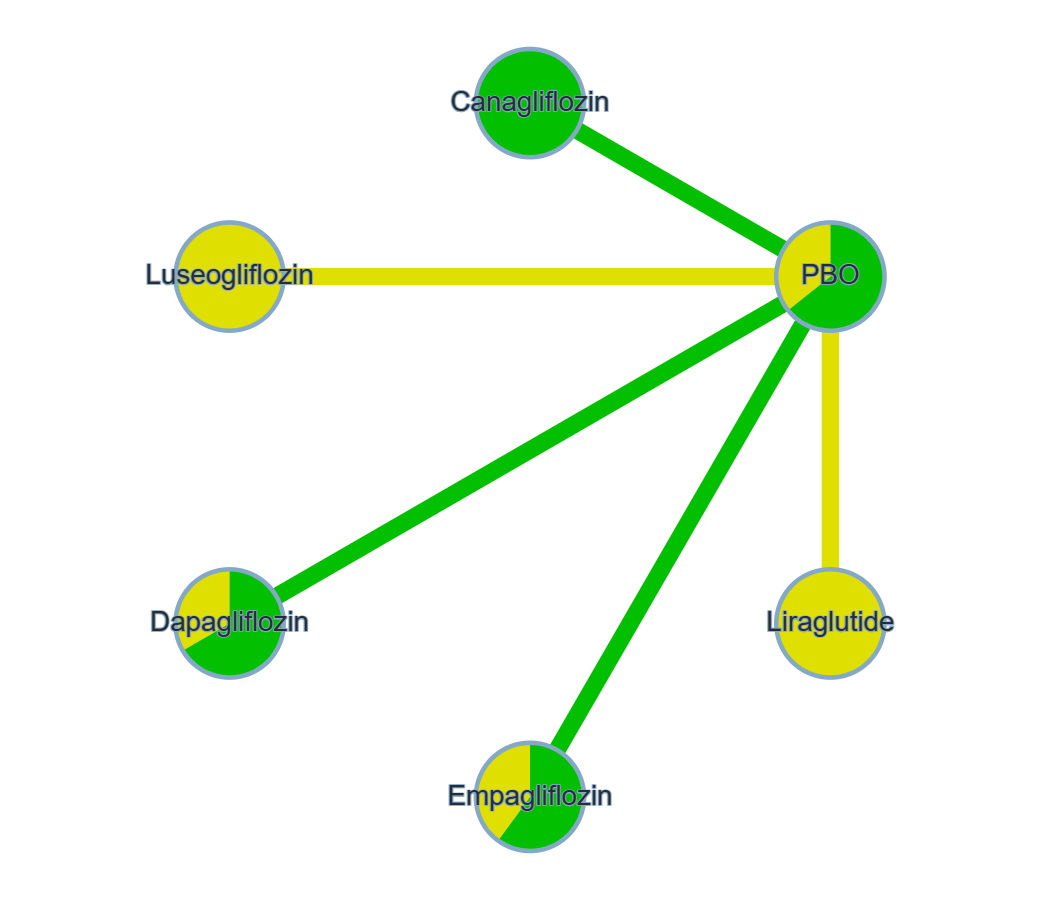


Network plot of study limitations of the included studies. Node size by equal size, node color by RoB. The colors in the circles indicate the percentage of low RoB studies (green), moderate RoB studies (yellow), high RoB studies (red) about each physical activity type. Edge width by sample size. Edge color by average RoB. The colors of the lines indicate the summative RoB assessment of each comparison. Low RoB is green, moderate RoB is yellow, high RoB is red. PBO: Placebo.

Contribution percentage of low, moderate, and high RoB comparisons to each network estimate

Low RoB is green, moderate RoB is yellow, high RoB is red.


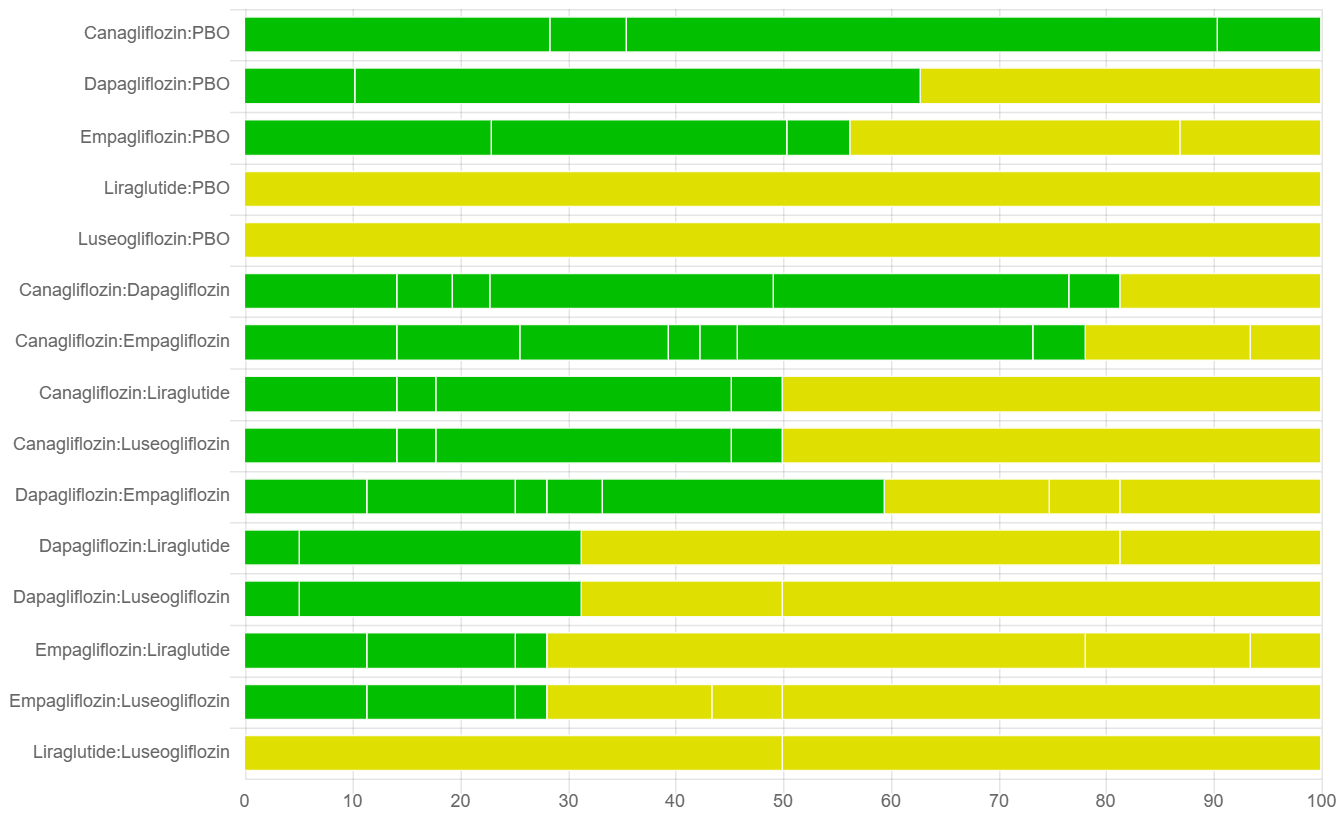


CINeMA for the outcome “DBP”

| Comparison | Number of studies | Within-study bias | Reporting bias | Indirectness | Imprecision | Heterogeneity | Incoherence | Confidence rating |
| --- | --- | --- | --- | --- | --- | --- | --- | --- |
| Canagliflozin:  PBO | 4 | No concerns | Low risk | No concerns | No concerns | Major concerns | Major concerns | Very low |
| Dapagliflozin:  PBO | 3 | No concerns | Low risk | No concerns | Major concerns | No concerns | Major concerns | Very low |
| Empagliflozin:  PBO | 5 | No concerns | Low risk | No concerns | No concerns | Major concerns | Major concerns | Very low |
| Liraglutide:  PBO | 1 | Some concerns | Low risk | No concerns | Major concerns | No concerns | Major concerns | Very low |
| Luseogliflozin:  PBO | 1 | Some concerns | Low risk | No concerns | Major concerns | No concerns | Major concerns | Very low |
| Canagliflozin:  Dapagliflozin | 0 | No concerns | Low risk | No concerns | Major concerns | No concerns | Major concerns | Very low |
| Canagliflozin:  Empagliflozin | 0 | No concerns | Low risk | No concerns | Major concerns | No concerns | Major concerns | Very low |
| Canagliflozin:  Liraglutide | 0 | Some concerns | Low risk | No concerns | Major concerns | No concerns | Major concerns | Very low |
| Canagliflozin:  Luseogliflozin | 0 | Some concerns | Low risk | No concerns | Major concerns | No concerns | Major concerns | Very low |
| Dapagliflozin:  Empagliflozin | 0 | No concerns | Low risk | No concerns | Major concerns | No concerns | Major concerns | Very low |
| Dapagliflozin:  Liraglutide | 0 | Some concerns | Low risk | No concerns | Major concerns | No concerns | Major concerns | Very low |
| Dapagliflozin:  Luseogliflozin | 0 | Some concerns | Low risk | No concerns | Major concerns | No concerns | Major concerns | Very low |
| Empagliflozin:  Liraglutide | 0 | Some concerns | Low risk | No concerns | Major concerns | No concerns | Major concerns | Very low |
| Empagliflozin:  Luseogliflozin | 0 | Some concerns | Low risk | No concerns | Major concerns | No concerns | Major concerns | Very low |
| Liraglutide:  Luseogliflozin | 0 | Some concerns | Low risk | No concerns | Major concerns | No concerns | Major concerns | Very low |

PBO: Placebo.

20.6 Summary of study limitations of the included studies (Body Weight)


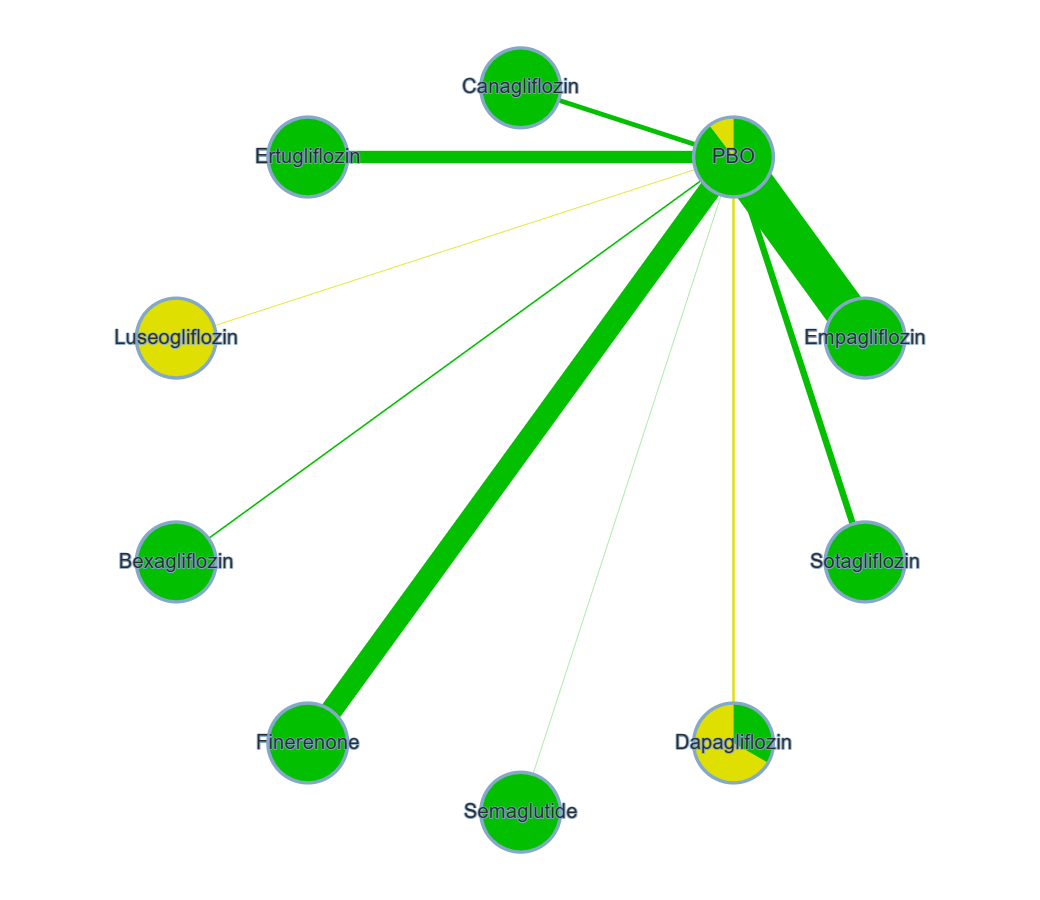


Network plot of study limitations of the included studies. Node size by equal size, node color by RoB. The colors in the circles indicate the percentage of low RoB studies (green), moderate RoB studies (yellow), high RoB studies (red) about each physical activity type. Edge width by sample size. Edge color by average RoB. The colors of the lines indicate the summative RoB assessment of each comparison. Low RoB is green, moderate RoB is yellow, high RoB is red. PBO: Placebo.

Contribution percentage of low, moderate, and high RoB comparisons to each network estimate

Low RoB is green, moderate RoB is yellow, high RoB is red.


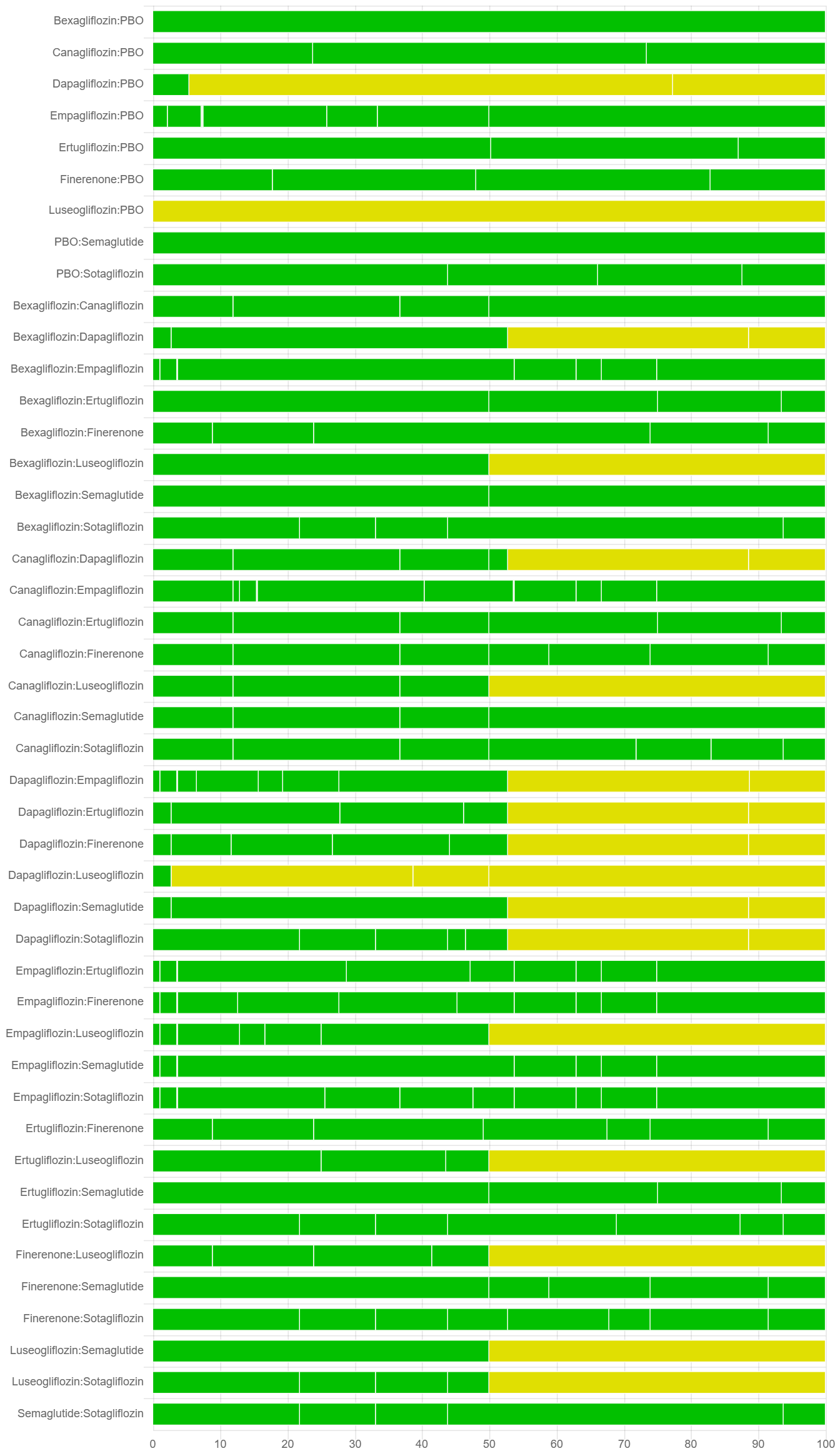


CINeMA for the outcome “Body Weight”

| Comparison | Number of studies | Within-study bias | Reporting bias | Indirectness | Imprecision | Heterogeneity | Incoherence | Confidence rating |
| --- | --- | --- | --- | --- | --- | --- | --- | --- |
| Bexagliflozin:  PBO | 1 | No concerns | Low risk | No concerns | Major concerns | No concerns | Major concerns | Very low |
| Canagliflozin:  PBO | 3 | No concerns | Low risk | No concerns | No concerns | No concerns | Major concerns | Low |
| Dapagliflozin:  PBO | 3 | Some concerns | Low risk | No concerns | Major concerns | No concerns | Major concerns | Very low |
| Empagliflozin:  PBO | 9 | No concerns | Low risk | No concerns | No concerns | No concerns | Major concerns | Low |
| Ertugliflozin:  PBO | 3 | No concerns | Low risk | No concerns | No concerns | No concerns | Major concerns | Low |
| Finerenone:  PBO | 4 | No concerns | Low risk | No concerns | Major concerns | No concerns | Major concerns | Very low |
| Luseogliflozin:PBO | 1 | Some concerns | Low risk | No concerns | Major concerns | No concerns | Major concerns | Very low |
| PBO:  Semaglutide | 1 | No concerns | Low risk | No concerns | Major concerns | No concerns | Major concerns | Very low |
| PBO:  Sotagliflozin | 4 | No concerns | Low risk | No concerns | Major concerns | No concerns | Major concerns | Very low |
| Bexagliflozin:  Canagliflozin | 0 | No concerns | Low risk | No concerns | Major concerns | No concerns | Major concerns | Very low |
| Bexagliflozin:  Dapagliflozin | 0 | No concerns | Low risk | No concerns | Major concerns | No concerns | Major concerns | Very low |
| Bexagliflozin:  Empagliflozin | 0 | No concerns | Low risk | No concerns | Major concerns | No concerns | Major concerns | Very low |
| Bexagliflozin:  Ertugliflozin | 0 | No concerns | Low risk | No concerns | Major concerns | No concerns | Major concerns | Very low |
| Bexagliflozin:  Finerenone | 0 | No concerns | Low risk | No concerns | Major concerns | No concerns | Major concerns | Very low |
| Bexagliflozin:  Luseogliflozin | 0 | Some concerns | Low risk | No concerns | Major concerns | No concerns | Major concerns | Very low |
| Bexagliflozin:  Semaglutide | 0 | No concerns | Low risk | No concerns | Major concerns | No concerns | Major concerns | Very low |
| Bexagliflozin:  Sotagliflozin | 0 | No concerns | Low risk | No concerns | Major concerns | No concerns | Major concerns | Very low |
| Canagliflozin:  Dapagliflozin | 0 | No concerns | Low risk | No concerns | No concerns | No concerns | Major concerns | Low |
| Canagliflozin:  Empagliflozin | 0 | No concerns | Low risk | No concerns | Major concerns | No concerns | Major concerns | Very low |
| Canagliflozin:  Ertugliflozin | 0 | No concerns | Low risk | No concerns | Major concerns | No concerns | Major concerns | Very low |
| Canagliflozin:  Finerenone | 0 | No concerns | Low risk | No concerns | No concerns | No concerns | Major concerns | Low |
| Canagliflozin:  Luseogliflozin | 0 | Some concerns | Low risk | No concerns | Major concerns | No concerns | Major concerns | Very low |
| Canagliflozin:  Semaglutide | 0 | No concerns | Low risk | No concerns | Major concerns | No concerns | Major concerns | Very low |
| Canagliflozin:  Sotagliflozin | 0 | No concerns | Low risk | No concerns | No concerns | No concerns | Major concerns | Low |
| Dapagliflozin:  Empagliflozin | 0 | No concerns | Low risk | No concerns | No concerns | No concerns | Major concerns | Low |
| Dapagliflozin:  Ertugliflozin | 0 | No concerns | Low risk | No concerns | No concerns | No concerns | Major concerns | Low |
| Dapagliflozin:  Finerenone | 0 | No concerns | Low risk | No concerns | Major concerns | No concerns | Major concerns | Very low |
| Dapagliflozin:  Luseogliflozin | 0 | Some concerns | Low risk | No concerns | Major concerns | No concerns | Major concerns | Very low |
| Dapagliflozin:  Semaglutide | 0 | No concerns | Low risk | No concerns | Major concerns | No concerns | Major concerns | Very low |
| Dapagliflozin:  Sotagliflozin | 0 | No concerns | Low risk | No concerns | Major concerns | No concerns | Major concerns | Very low |
| Empagliflozin:  Ertugliflozin | 0 | No concerns | Low risk | No concerns | Major concerns | No concerns | Major concerns | Very low |
| Empagliflozin:  Finerenone | 0 | No concerns | Low risk | No concerns | No concerns | No concerns | Major concerns | Low |
| Empagliflozin:  Luseogliflozin | 0 | Some concerns | Low risk | No concerns | Major concerns | No concerns | Major concerns | Very low |
| Empagliflozin:  Semaglutide | 0 | No concerns | Low risk | No concerns | Major concerns | No concerns | Major concerns | Very low |
| Empagliflozin:  Sotagliflozin | 0 | No concerns | Low risk | No concerns | Major concerns | No concerns | Major concerns | Very low |
| Comparison | Number of studies | Within-study bias | Reporting bias | Indirectness | Imprecision | Heterogeneity | Incoherence | Confidence rating |
| Ertugliflozin:  Finerenone | 0 | No concerns | Low risk | No concerns | No concerns | No concerns | Major concerns | Low |
| Ertugliflozin:  Luseogliflozin | 0 | Some concerns | Low risk | No concerns | Major concerns | No concerns | Major concerns | Very low |
| Ertugliflozin:  Semaglutide | 0 | No concerns | Low risk | No concerns | Major concerns | No concerns | Major concerns | Very low |
| Ertugliflozin:  Sotagliflozin | 0 | No concerns | Low risk | No concerns | Major concerns | No concerns | Major concerns | Very low |
| Finerenone:  Luseogliflozin | 0 | Some concerns | Low risk | No concerns | Major concerns | No concerns | Major concerns | Very low |
| Finerenone:  Semaglutide | 0 | No concerns | Low risk | No concerns | Major concerns | No concerns | Major concerns | Very low |
| Finerenone:  Sotagliflozin | 0 | No concerns | Low risk | No concerns | Major concerns | No concerns | Major concerns | Very low |
| Luseogliflozin:  Semaglutide | 0 | Some concerns | Low risk | No concerns | Major concerns | No concerns | Major concerns | Very low |
| Luseogliflozin:  Sotagliflozin | 0 | Some concerns | Low risk | No concerns | Major concerns | No concerns | Major concerns | Very low |
| Semaglutide:  Sotagliflozin | 0 | No concerns | Low risk | No concerns | Major concerns | No concerns | Major concerns | Very low |

PBO: Placebo.

20.7 Summary of study limitations of the included studies (Any AE)


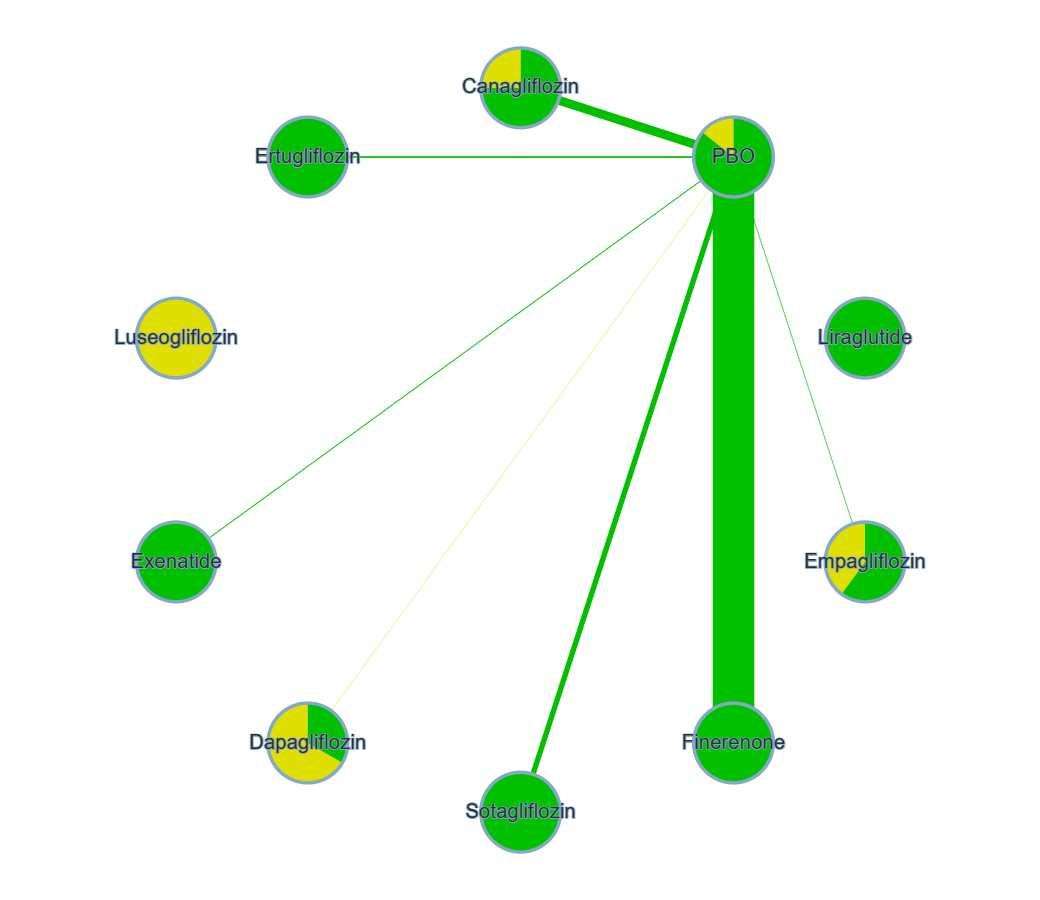


Network plot of study limitations of the included studies. Node size by equal size, node color by RoB. The colors in the circles indicate the percentage of low RoB studies (green), moderate RoB studies (yellow), high RoB studies (red) about each physical activity type. Edge width by sample size. Edge color by average RoB. The colors of the lines indicate the summative RoB assessment of each comparison. Low RoB is green, moderate RoB is yellow, high RoB is red. PBO: Placebo.

Contribution percentage of low, moderate, and high RoB comparisons to each network estimate

Low RoB is green, moderate RoB is yellow, high RoB is red.


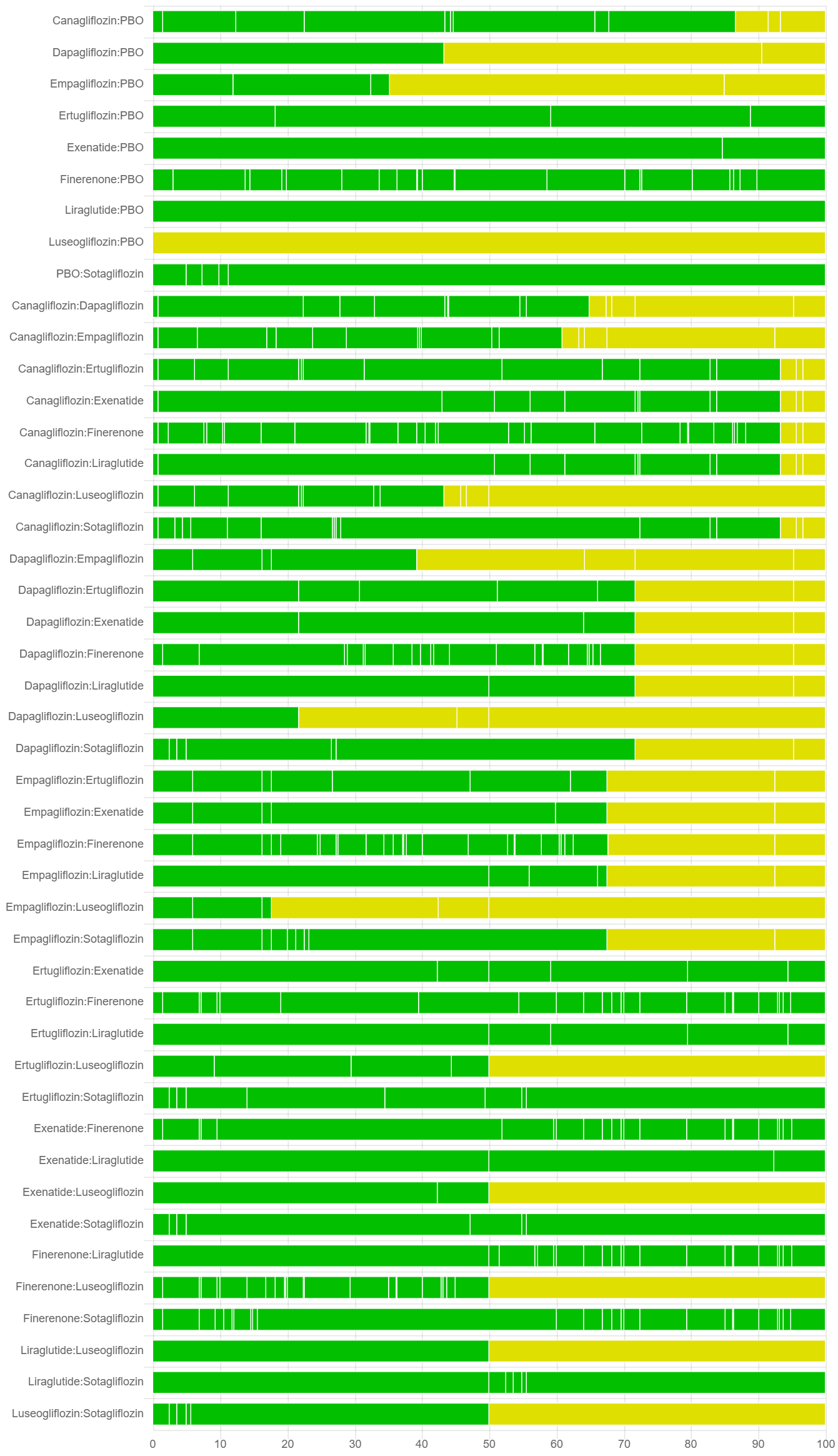


CINeMA for the outcome “Any AE”

| Comparison | Number of studies | Within-study bias | Reporting bias | Indirectness | Imprecision | Heterogeneity | Incoherence | Confidence rating |
| --- | --- | --- | --- | --- | --- | --- | --- | --- |
| Canagliflozin:  PBO | 12 | No concerns | Low risk | No concerns | No concerns | No concerns | Major concerns | Low |
| Dapagliflozin:  PBO | 3 | Some concerns | Low risk | No concerns | Major concerns | No concerns | Major concerns | Very low |
| Empagliflozin:  PBO | 5 | Some concerns | Low risk | No concerns | Major concerns | No concerns | Major concerns | Very low |
| Ertugliflozin:  PBO | 4 | No concerns | Low risk | No concerns | Major concerns | No concerns | Major concerns | Very low |
| Exenatide:PBO | 2 | No concerns | Low risk | No concerns | No concerns | No concerns | Major concerns | Low |
| Finerenone:  PBO | 23 | No concerns | Low risk | No concerns | Major concerns | No concerns | Major concerns | Very low |
| Liraglutide:  PBO | 1 | No concerns | Low risk | No concerns | Major concerns | No concerns | Major concerns | Very low |
| Luseogliflozin:PBO | 1 | Some concerns | Low risk | No concerns | Major concerns | No concerns | Major concerns | Very low |
| PBO:  Sotagliflozin | 5 | No concerns | Low risk | No concerns | Major concerns | No concerns | Major concerns | Very low |
| Canagliflozin:  Dapagliflozin | 0 | No concerns | Low risk | No concerns | Major concerns | No concerns | Major concerns | Very low |
| Canagliflozin:  Empagliflozin | 0 | No concerns | Low risk | No concerns | Major concerns | No concerns | Major concerns | Very low |
| Canagliflozin:  Ertugliflozin | 0 | No concerns | Low risk | No concerns | Major concerns | No concerns | Major concerns | Very low |
| Canagliflozin:  Exenatide | 0 | No concerns | Low risk | No concerns | No concerns | No concerns | Major concerns | Low |
| Canagliflozin:  Finerenone | 0 | No concerns | Low risk | No concerns | No concerns | No concerns | Major concerns | Low |
| Canagliflozin:  Liraglutide | 0 | No concerns | Low risk | No concerns | Major concerns | No concerns | Major concerns | Very low |
| Canagliflozin:  Luseogliflozin | 0 | Some concerns | Low risk | No concerns | Major concerns | No concerns | Major concerns | Very low |
| Canagliflozin:  Sotagliflozin | 0 | No concerns | Low risk | No concerns | No concerns | No concerns | Major concerns | Low |
| Dapagliflozin:  Empagliflozin | 0 | Some concerns | Low risk | No concerns | Major concerns | No concerns | Major concerns | Very low |
| Dapagliflozin:  Ertugliflozin | 0 | No concerns | Low risk | No concerns | Major concerns | No concerns | Major concerns | Very low |
| Dapagliflozin:  Exenatide | 0 | No concerns | Low risk | No concerns | Major concerns | No concerns | Major concerns | Very low |
| Dapagliflozin:  Finerenone | 0 | No concerns | Low risk | No concerns | Major concerns | No concerns | Major concerns | Very low |
| Dapagliflozin:  Liraglutide | 0 | No concerns | Low risk | No concerns | Major concerns | No concerns | Major concerns | Very low |
| Dapagliflozin:  Luseogliflozin | 0 | Some concerns | Low risk | No concerns | Major concerns | No concerns | Major concerns | Very low |
| Dapagliflozin:  Sotagliflozin | 0 | No concerns | Low risk | No concerns | Major concerns | No concerns | Major concerns | Very low |
| Empagliflozin:  Ertugliflozin | 0 | No concerns | Low risk | No concerns | Major concerns | No concerns | Major concerns | Very low |
| Empagliflozin:  Exenatide | 0 | No concerns | Low risk | No concerns | No concerns | No concerns | Major concerns | Low |
| Empagliflozin:  Finerenone | 0 | No concerns | Low risk | No concerns | Major concerns | No concerns | Major concerns | Very low |
| Empagliflozin:  Liraglutide | 0 | No concerns | Low risk | No concerns | Major concerns | No concerns | Major concerns | Very low |
| Empagliflozin:  Luseogliflozin | 0 | Some concerns | Low risk | No concerns | Major concerns | No concerns | Major concerns | Very low |
| Empagliflozin:  Sotagliflozin | 0 | No concerns | Low risk | No concerns | Major concerns | No concerns | Major concerns | Very low |
| Ertugliflozin:  Exenatide | 0 | No concerns | Low risk | No concerns | Major concerns | No concerns | Major concerns | Very low |
| Ertugliflozin:  Finerenone | 0 | No concerns | Low risk | No concerns | Major concerns | No concerns | Major concerns | Very low |
| Ertugliflozin:  Liraglutide | 0 | No concerns | Low risk | No concerns | Major concerns | No concerns | Major concerns | Very low |
| Ertugliflozin:  Luseogliflozin | 0 | Some concerns | Low risk | No concerns | Major concerns | No concerns | Major concerns | Very low |
| Ertugliflozin:  Sotagliflozin | 0 | No concerns | Low risk | No concerns | Major concerns | No concerns | Major concerns | Very low |
| Comparison | Number of studies | Within-study bias | Reporting bias | Indirectness | Imprecision | Heterogeneity | Incoherence | Confidence rating |
| Exenatide:  Finerenone | 0 | No concerns | Low risk | No concerns | No concerns | No concerns | Major concerns | Low |
| Exenatide:  Liraglutide | 0 | No concerns | Low risk | No concerns | Major concerns | No concerns | Major concerns | Very low |
| Exenatide:  Luseogliflozin | 0 | Some concerns | Low risk | No concerns | Major concerns | No concerns | Major concerns | Very low |
| Exenatide:  Sotagliflozin | 0 | No concerns | Low risk | No concerns | No concerns | No concerns | Major concerns | Low |
| Finerenone:  Liraglutide | 0 | No concerns | Low risk | No concerns | Major concerns | No concerns | Major concerns | Very low |
| Finerenone:  Luseogliflozin | 0 | No concerns | Low risk | No concerns | Major concerns | No concerns | Major concerns | Very low |
| Finerenone:  Sotagliflozin | 0 | No concerns | Low risk | No concerns | Major concerns | No concerns | Major concerns | Very low |
| Liraglutide:  Luseogliflozin | 0 | Some concerns | Low risk | No concerns | Major concerns | No concerns | Major concerns | Very low |
| Liraglutide:  Sotagliflozin | 0 | No concerns | Low risk | No concerns | Major concerns | No concerns | Major concerns | Very low |
| Luseogliflozin:  Sotagliflozin | 0 | No concerns | Low risk | No concerns | Major concerns | No concerns | Major concerns | Very low |

PBO: Placebo.

20.8 Summary of study limitations of the included studies (UTI)


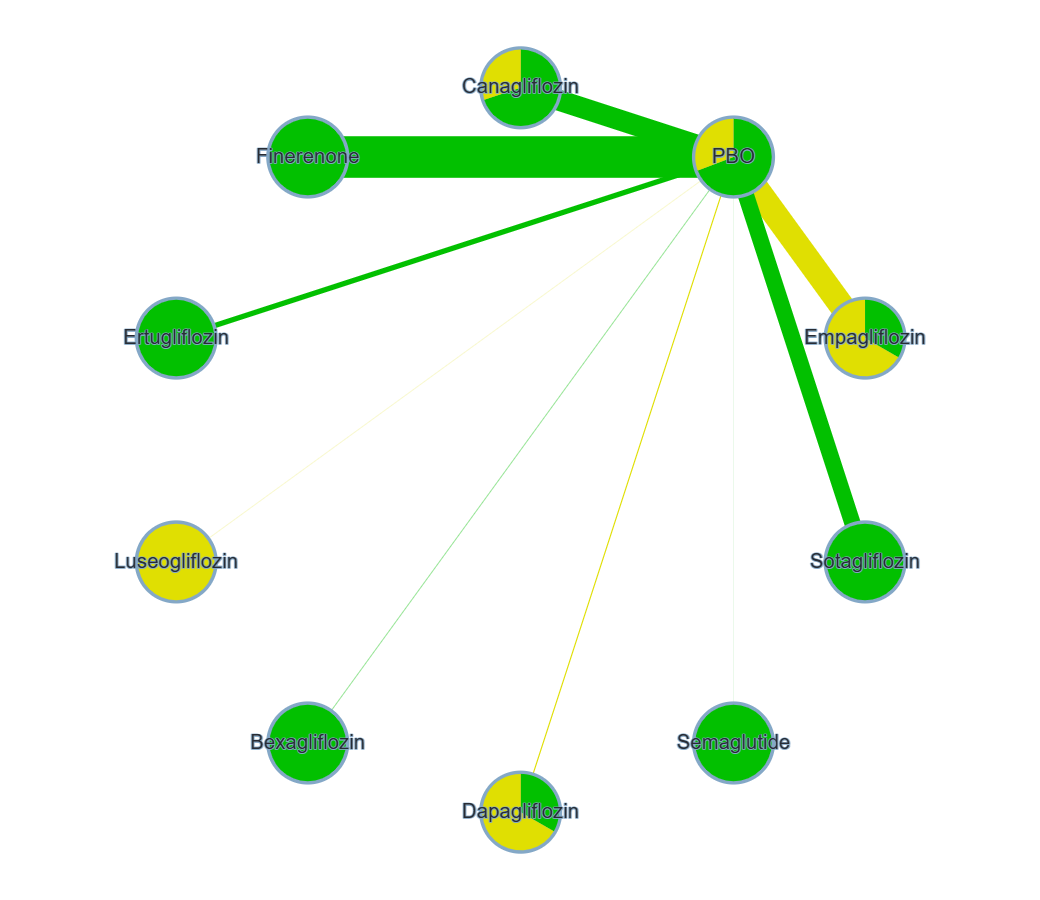


Network plot of study limitations of the included studies. Node size by equal size, node color by RoB. The colors in the circles indicate the percentage of low RoB studies (green), moderate RoB studies (yellow), high RoB studies (red) about each physical activity type. Edge width by sample size. Edge color by average RoB. The colors of the lines indicate the summative RoB assessment of each comparison. Low RoB is green, moderate RoB is yellow, high RoB is red. PBO: Placebo.

Contribution percentage of low, moderate, and high RoB comparisons to each network estimate

Low RoB is green, moderate RoB is yellow, high RoB is red.


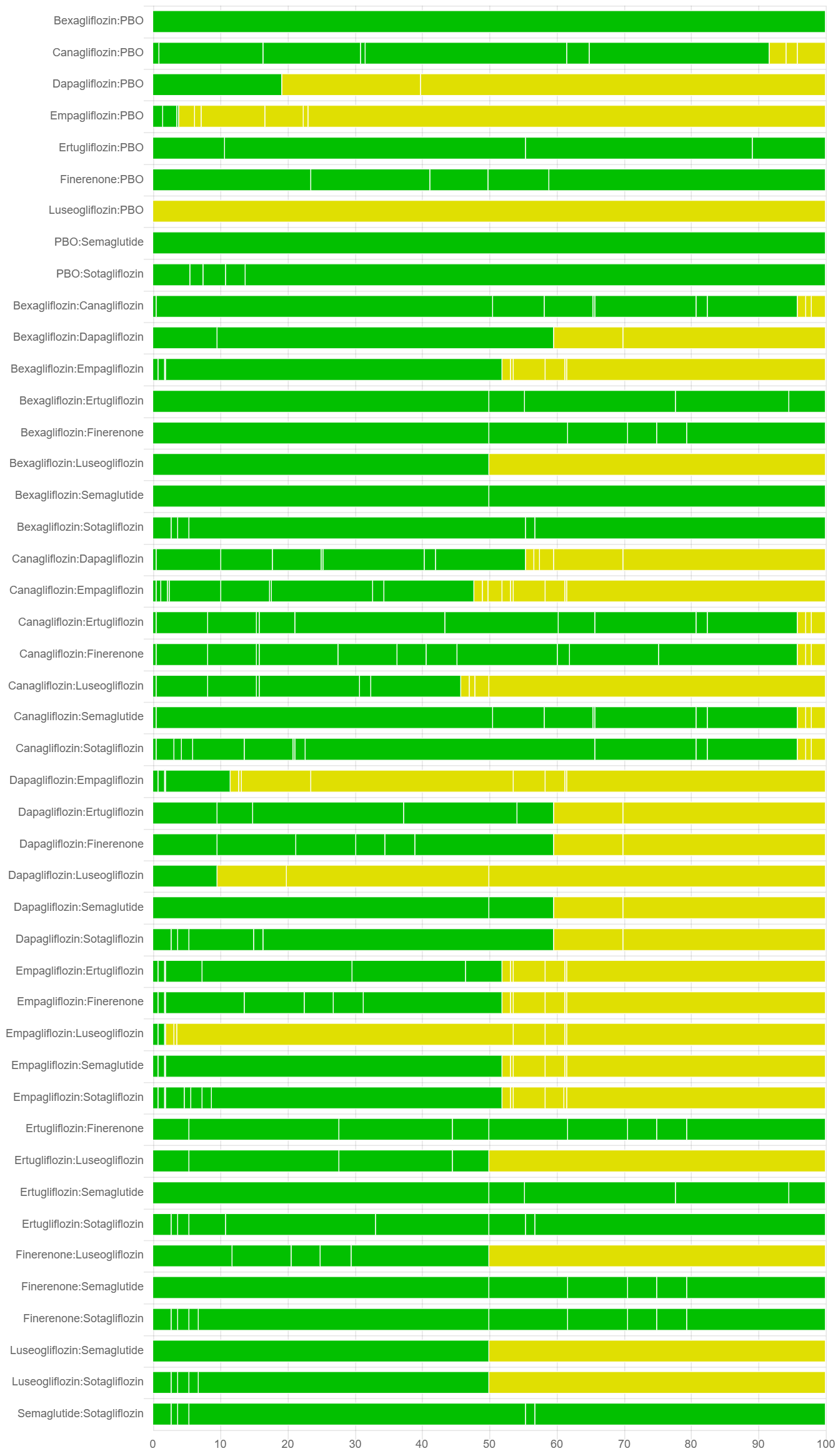


CINeMA for the outcome “UTI”

| Comparison | Number of studies | Within-study bias | Reporting bias | Indirectness | Imprecision | Heterogeneity | Incoherence | Confidence rating |
| --- | --- | --- | --- | --- | --- | --- | --- | --- |
| Bexagliflozin:  PBO | 1 | No concerns | Low risk | No concerns | Major concerns | No concerns | Major concerns | Very low |
| Canagliflozin:  PBO | 10 | No concerns | Low risk | No concerns | No concerns | No concerns | Major concerns | Low |
| Dapagliflozin:  PBO | 3 | Some concerns | Low risk | No concerns | Major concerns | No concerns | Major concerns | Very low |
| Empagliflozin:  PBO | 9 | Some concerns | Low risk | No concerns | Major concerns | No concerns | Major concerns | Very low |
| Ertugliflozin:  PBO | 4 | No concerns | Low risk | No concerns | Major concerns | No concerns | Major concerns | Very low |
| Finerenone:  PBO | 5 | No concerns | Low risk | No concerns | Major concerns | No concerns | Major concerns | Very low |
| Luseogliflozin:  PBO | 1 | Some concerns | Low risk | No concerns | Major concerns | No concerns | Major concerns | Very low |
| PBO:  Semaglutide | 1 | No concerns | Low risk | No concerns | Major concerns | No concerns | Major concerns | Very low |
| PBO:  Sotagliflozin | 5 | No concerns | Low risk | No concerns | Major concerns | No concerns | Major concerns | Very low |
| Bexagliflozin:  Canagliflozin | 0 | No concerns | Low risk | No concerns | Major concerns | No concerns | Major concerns | Very low |
| Bexagliflozin:  Dapagliflozin | 0 | No concerns | Low risk | No concerns | Major concerns | No concerns | Major concerns | Very low |
| Bexagliflozin:  Empagliflozin | 0 | No concerns | Low risk | No concerns | Major concerns | No concerns | Major concerns | Very low |
| Bexagliflozin:  Ertugliflozin | 0 | No concerns | Low risk | No concerns | Major concerns | No concerns | Major concerns | Very low |
| Bexagliflozin:  Finerenone | 0 | No concerns | Low risk | No concerns | Major concerns | No concerns | Major concerns | Very low |
| Bexagliflozin:  Luseogliflozin | 0 | Some concerns | Low risk | No concerns | Major concerns | No concerns | Major concerns | Very low |
| Bexagliflozin:  Semaglutide | 0 | No concerns | Low risk | No concerns | Major concerns | No concerns | Major concerns | Very low |
| Bexagliflozin:  Sotagliflozin | 0 | No concerns | Low risk | No concerns | Major concerns | No concerns | Major concerns | Very low |
| Canagliflozin:  Dapagliflozin | 0 | No concerns | Low risk | No concerns | Major concerns | No concerns | Major concerns | Very low |
| Canagliflozin:  Empagliflozin | 0 | Some concerns | Low risk | No concerns | Major concerns | No concerns | Major concerns | Very low |
| Canagliflozin:  Ertugliflozin | 0 | No concerns | Low risk | No concerns | Major concerns | No concerns | Major concerns | Very low |
| Canagliflozin:  Finerenone | 0 | No concerns | Low risk | No concerns | Major concerns | No concerns | Major concerns | Very low |
| Canagliflozin:  Luseogliflozin | 0 | Some concerns | Low risk | No concerns | Major concerns | No concerns | Major concerns | Very low |
| Canagliflozin:  Semaglutide | 0 | No concerns | Low risk | No concerns | Major concerns | No concerns | Major concerns | Very low |
| Canagliflozin:  Sotagliflozin | 0 | No concerns | Low risk | No concerns | Major concerns | No concerns | Major concerns | Very low |
| Dapagliflozin:  Empagliflozin | 0 | Some concerns | Low risk | No concerns | Major concerns | No concerns | Major concerns | Very low |
| Dapagliflozin:  Ertugliflozin | 0 | No concerns | Low risk | No concerns | Major concerns | No concerns | Major concerns | Very low |
| Dapagliflozin:  Finerenone | 0 | No concerns | Low risk | No concerns | Major concerns | No concerns | Major concerns | Very low |
| Dapagliflozin:  Luseogliflozin | 0 | Some concerns | Low risk | No concerns | Major concerns | No concerns | Major concerns | Very low |
| Dapagliflozin:  Semaglutide | 0 | No concerns | Low risk | No concerns | Major concerns | No concerns | Major concerns | Very low |
| Dapagliflozin:  Sotagliflozin | 0 | No concerns | Low risk | No concerns | Major concerns | No concerns | Major concerns | Very low |
| Empagliflozin:  Ertugliflozin | 0 | No concerns | Low risk | No concerns | Major concerns | No concerns | Major concerns | Very low |
| Empagliflozin:  Finerenone | 0 | No concerns | Low risk | No concerns | Major concerns | No concerns | Major concerns | Very low |
| Empagliflozin:  Luseogliflozin | 0 | Some concerns | Low risk | No concerns | Major concerns | No concerns | Major concerns | Very low |
| Empagliflozin:  Semaglutide | 0 | No concerns | Low risk | No concerns | Major concerns | No concerns | Major concerns | Very low |
| Empagliflozin:  Sotagliflozin | 0 | No concerns | Low risk | No concerns | Major concerns | No concerns | Major concerns | Very low |
| Comparison | Number of studies | Within-study bias | Reporting bias | Indirectness | Imprecision | Heterogeneity | Incoherence | Confidence rating |
| Ertugliflozin:  Finerenone | 0 | No concerns | Low risk | No concerns | Major concerns | No concerns | Major concerns | Very low |
| Ertugliflozin:  Luseogliflozin | 0 | Some concerns | Low risk | No concerns | Major concerns | No concerns | Major concerns | Very low |
| Ertugliflozin:  Semaglutide | 0 | No concerns | Low risk | No concerns | Major concerns | No concerns | Major concerns | Very low |
| Ertugliflozin:  Sotagliflozin | 0 | No concerns | Low risk | No concerns | Major concerns | No concerns | Major concerns | Very low |
| Finerenone:  Luseogliflozin | 0 | Some concerns | Low risk | No concerns | Major concerns | No concerns | Major concerns | Very low |
| Finerenone:  Semaglutide | 0 | No concerns | Low risk | No concerns | Major concerns | No concerns | Major concerns | Very low |
| Finerenone:  Sotagliflozin | 0 | No concerns | Low risk | No concerns | Major concerns | No concerns | Major concerns | Very low |
| Luseogliflozin:Semaglutide | 0 | Some concerns | Low risk | No concerns | Major concerns | No concerns | Major concerns | Very low |
| Luseogliflozin:Sotagliflozin | 0 | Some concerns | Low risk | No concerns | Major concerns | No concerns | Major concerns | Very low |
| Semaglutide:  Sotagliflozin | 0 | No concerns | Low risk | No concerns | Major concerns | No concerns | Major concerns | Very low |

PBO: Placebo.

20.9 Summary of study limitations of the included studies (Hypoglycemia)


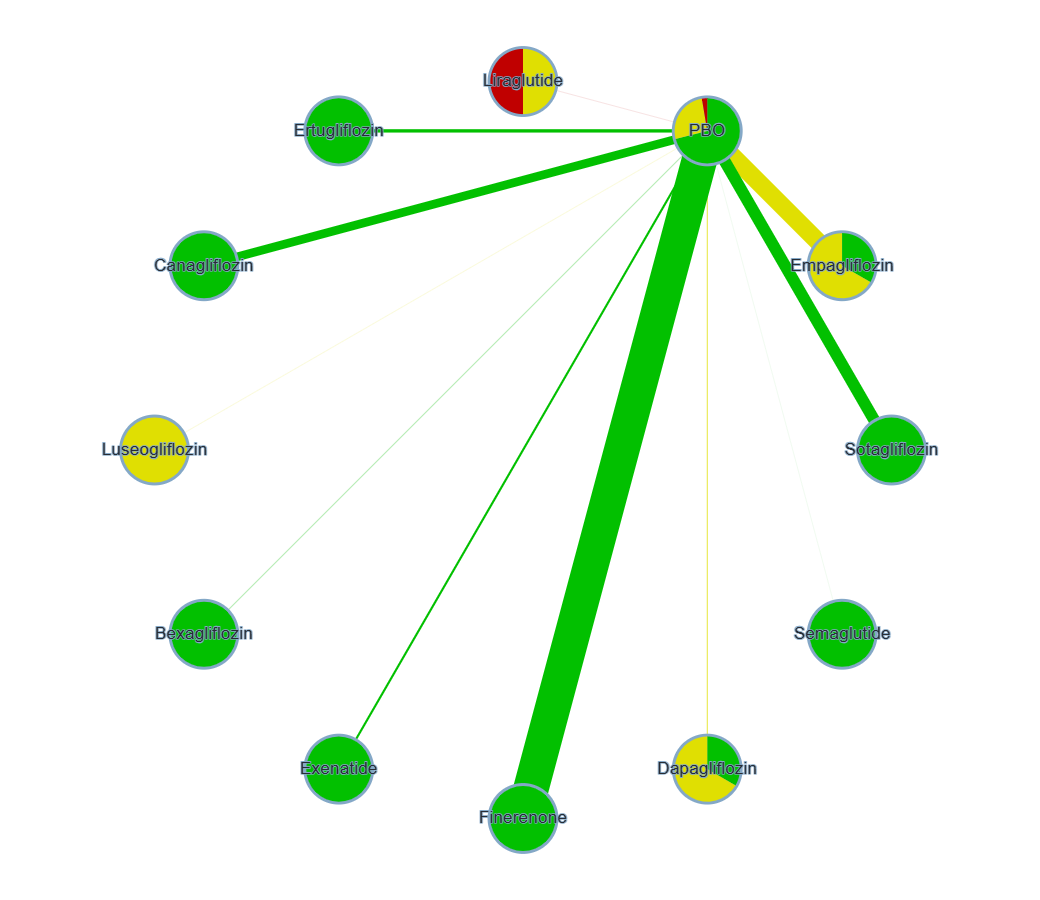


Network plot of study limitations of the included studies. Node size by equal size, node color by RoB. The colors in the circles indicate the percentage of low RoB studies (green), moderate RoB studies (yellow), high RoB studies (red) about each physical activity type. Edge width by sample size. Edge color by average RoB. The colors of the lines indicate the summative RoB assessment of each comparison. Low RoB is green, moderate RoB is yellow, high RoB is red. PBO: Placebo.

Contribution percentage of low, moderate, and high RoB comparisons to each network estimate

Low RoB is green, moderate RoB is yellow, high RoB is red.


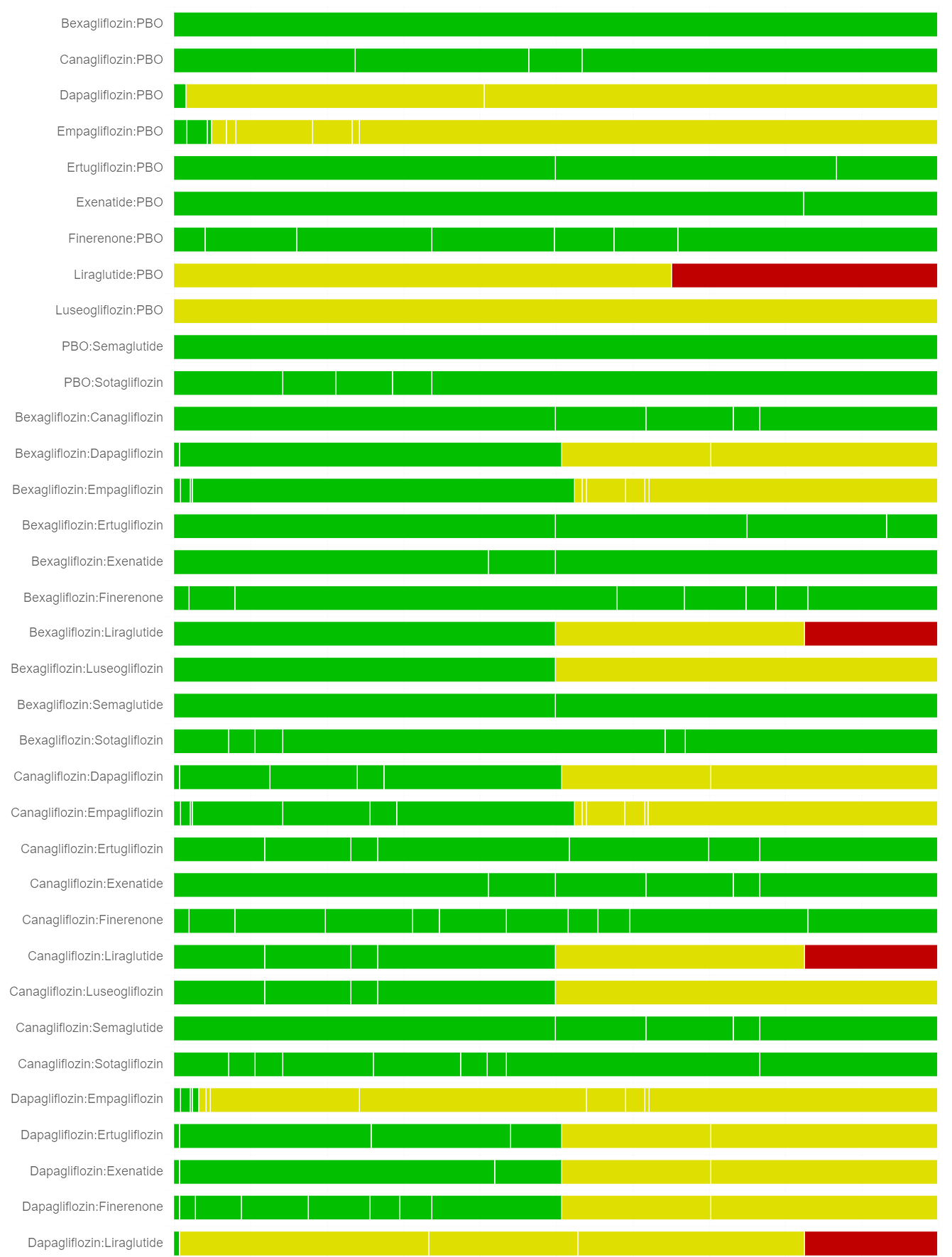


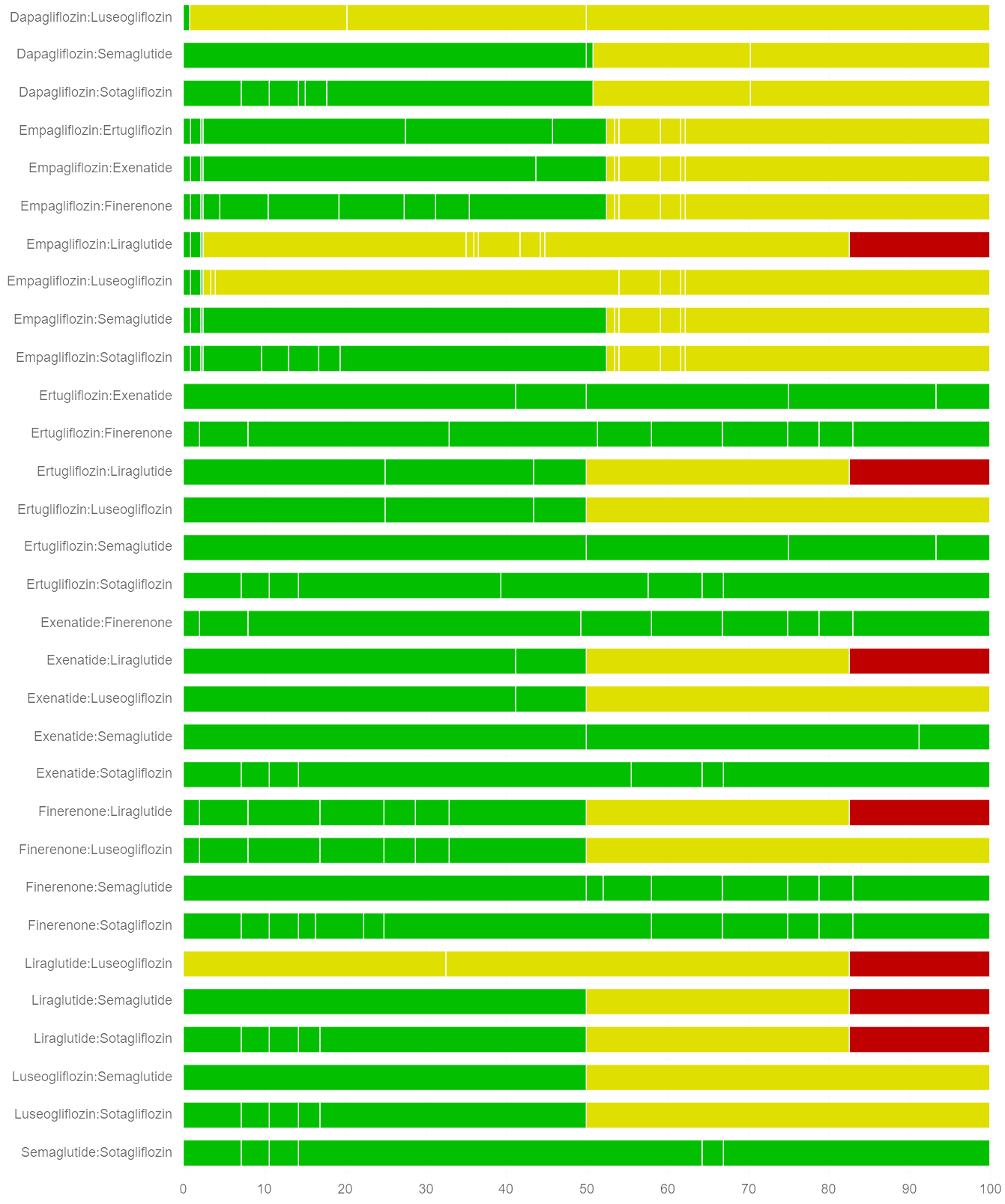


PBO: Placebo.

CINeMA for the outcome “Hypoglycemia”

| Comparison | Number of studies | Within-study bias | Reporting bias | Indirectness | Imprecision | Heterogeneity | Incoherence | Confidence rating |
| --- | --- | --- | --- | --- | --- | --- | --- | --- |
| Bexagliflozin:  PBO | 1 | No concerns | Low risk | No concerns | Major concerns | No concerns | Major concerns | Very low |
| Canagliflozin:  PBO | 4 | No concerns | Low risk | No concerns | Major concerns | No concerns | Major concerns | Very low |
| Dapagliflozin:  PBO | 3 | Some concerns | Low risk | No concerns | Major concerns | No concerns | Major concerns | Very low |
| Empagliflozin:  PBO | 9 | Some concerns | Low risk | No concerns | No concerns | No concerns | Major concerns | Very low |
| Ertugliflozin:  PBO | 3 | No concerns | Low risk | No concerns | Major concerns | No concerns | Major concerns | Very low |
| Exenatide:PBO | 2 | No concerns | Low risk | No concerns | Major concerns | No concerns | Major concerns | Very low |
| Finerenone:  PBO | 7 | No concerns | Low risk | No concerns | No concerns | No concerns | Major concerns | Low |
| Liraglutide:  PBO | 2 | Some concerns | Low risk | No concerns | Major concerns | No concerns | Major concerns | Very low |
| Luseogliflozin:PBO | 1 | Some concerns | Low risk | No concerns | Major concerns | No concerns | Major concerns | Very low |
| PBO:  Semaglutide | 1 | No concerns | Low risk | No concerns | Major concerns | No concerns | Major concerns | Very low |
| PBO:  Sotagliflozin | 5 | No concerns | Low risk | No concerns | Major concerns | No concerns | Major concerns | Very low |
| Bexagliflozin:  Canagliflozin | 0 | No concerns | Low risk | No concerns | Major concerns | No concerns | Major concerns | Very low |
| Bexagliflozin:  Dapagliflozin | 0 | No concerns | Low risk | No concerns | Major concerns | No concerns | Major concerns | Very low |
| Bexagliflozin:  Empagliflozin | 0 | No concerns | Low risk | No concerns | Major concerns | No concerns | Major concerns | Very low |
| Bexagliflozin:  Ertugliflozin | 0 | No concerns | Low risk | No concerns | Major concerns | No concerns | Major concerns | Very low |
| Bexagliflozin:  Exenatide | 0 | No concerns | Low risk | No concerns | Major concerns | No concerns | Major concerns | Very low |
| Bexagliflozin:  Finerenone | 0 | No concerns | Low risk | No concerns | Major concerns | No concerns | Major concerns | Very low |
| Bexagliflozin:  Liraglutide | 0 | Some concerns | Low risk | No concerns | Major concerns | No concerns | Major concerns | Very low |
| Bexagliflozin:  Luseogliflozin | 0 | Some concerns | Low risk | No concerns | Major concerns | No concerns | Major concerns | Very low |
| Bexagliflozin:  Semaglutide | 0 | No concerns | Low risk | No concerns | Major concerns | No concerns | Major concerns | Very low |
| Bexagliflozin:  Sotagliflozin | 0 | No concerns | Low risk | No concerns | Major concerns | No concerns | Major concerns | Very low |
| Canagliflozin:  Dapagliflozin | 0 | No concerns | Low risk | No concerns | Major concerns | No concerns | Major concerns | Very low |
| Canagliflozin:  Empagliflozin | 0 | No concerns | Low risk | No concerns | Major concerns | No concerns | Major concerns | Very low |
| Canagliflozin:  Ertugliflozin | 0 | No concerns | Low risk | No concerns | Major concerns | No concerns | Major concerns | Very low |
| Canagliflozin:  Exenatide | 0 | No concerns | Low risk | No concerns | Major concerns | No concerns | Major concerns | Very low |
| Canagliflozin:  Finerenone | 0 | No concerns | Low risk | No concerns | Major concerns | No concerns | Major concerns | Very low |
| Canagliflozin:  Liraglutide | 0 | Some concerns | Low risk | No concerns | Major concerns | No concerns | Major concerns | Very low |
| Canagliflozin:  Luseogliflozin | 0 | Some concerns | Low risk | No concerns | Major concerns | No concerns | Major concerns | Very low |
| Canagliflozin:  Semaglutide | 0 | No concerns | Low risk | No concerns | Major concerns | No concerns | Major concerns | Very low |
| Canagliflozin:  Sotagliflozin | 0 | No concerns | Low risk | No concerns | Major concerns | No concerns | Major concerns | Very low |
| Dapagliflozin:  Empagliflozin | 0 | Some concerns | Low risk | No concerns | Major concerns | No concerns | Major concerns | Very low |
| Dapagliflozin:  Ertugliflozin | 0 | No concerns | Low risk | No concerns | Major concerns | No concerns | Major concerns | Very low |
| Dapagliflozin:  Exenatide | 0 | No concerns | Low risk | No concerns | Major concerns | No concerns | Major concerns | Very low |
| Dapagliflozin:  Finerenone | 0 | No concerns | Low risk | No concerns | Major concerns | No concerns | Major concerns | Very low |
| Comparison | Number of studies | Within-study bias | Reporting bias | Indirectness | Imprecision | Heterogeneity | Incoherence | Confidence rating |
| Dapagliflozin:  Liraglutide | 0 | Some concerns | Low risk | No concerns | Major concerns | No concerns | Major concerns | Very low |
| Dapagliflozin:  Luseogliflozin | 0 | Some concerns | Low risk | No concerns | Major concerns | No concerns | Major concerns | Very low |
| Dapagliflozin:  Semaglutide | 0 | No concerns | Low risk | No concerns | Major concerns | No concerns | Major concerns | Very low |
| Dapagliflozin:  Sotagliflozin | 0 | No concerns | Low risk | No concerns | Major concerns | No concerns | Major concerns | Very low |
| Empagliflozin:  Ertugliflozin | 0 | No concerns | Low risk | No concerns | Major concerns | No concerns | Major concerns | Very low |
| Empagliflozin:  Exenatide | 0 | No concerns | Low risk | No concerns | Major concerns | No concerns | Major concerns | Very low |
| Empagliflozin:  Finerenone | 0 | No concerns | Low risk | No concerns | Major concerns | No concerns | Major concerns | Very low |
| Empagliflozin:  Liraglutide | 0 | Some concerns | Low risk | No concerns | Major concerns | No concerns | Major concerns | Very low |
| Empagliflozin:  Luseogliflozin | 0 | Some concerns | Low risk | No concerns | Major concerns | No concerns | Major concerns | Very low |
| Empagliflozin:  Semaglutide | 0 | No concerns | Low risk | No concerns | Major concerns | No concerns | Major concerns | Very low |
| Empagliflozin:  Sotagliflozin | 0 | No concerns | Low risk | No concerns | Major concerns | No concerns | Major concerns | Very low |
| Ertugliflozin:  Exenatide | 0 | No concerns | Low risk | No concerns | Major concerns | No concerns | Major concerns | Very low |
| Ertugliflozin:  Finerenone | 0 | No concerns | Low risk | No concerns | Major concerns | No concerns | Major concerns | Very low |
| Ertugliflozin:  Liraglutide | 0 | Some concerns | Low risk | No concerns | Major concerns | No concerns | Major concerns | Very low |
| Ertugliflozin:  Luseogliflozin | 0 | Some concerns | Low risk | No concerns | Major concerns | No concerns | Major concerns | Very low |
| Ertugliflozin:  Semaglutide | 0 | No concerns | Low risk | No concerns | Major concerns | No concerns | Major concerns | Very low |
| Ertugliflozin:  Sotagliflozin | 0 | No concerns | Low risk | No concerns | Major concerns | No concerns | Major concerns | Very low |
| Exenatide:  Finerenone | 0 | No concerns | Low risk | No concerns | Major concerns | No concerns | Major concerns | Very low |
| Exenatide:  Liraglutide | 0 | Some concerns | Low risk | No concerns | Major concerns | No concerns | Major concerns | Very low |
| Exenatide:  Luseogliflozin | 0 | Some concerns | Low risk | No concerns | Major concerns | No concerns | Major concerns | Very low |
| Exenatide:  Semaglutide | 0 | No concerns | Low risk | No concerns | Major concerns | No concerns | Major concerns | Very low |
| Exenatide:  Sotagliflozin | 0 | No concerns | Low risk | No concerns | Major concerns | No concerns | Major concerns | Very low |
| Finerenone:  Liraglutide | 0 | Some concerns | Low risk | No concerns | Major concerns | No concerns | Major concerns | Very low |
| Finerenone:  Luseogliflozin | 0 | Some concerns | Low risk | No concerns | Major concerns | No concerns | Major concerns | Very low |
| Finerenone:  Semaglutide | 0 | No concerns | Low risk | No concerns | Major concerns | No concerns | Major concerns | Very low |
| Finerenone:  Sotagliflozin | 0 | No concerns | Low risk | No concerns | Major concerns | No concerns | Major concerns | Very low |
| Liraglutide:  Luseogliflozin | 0 | Some concerns | Low risk | No concerns | Major concerns | No concerns | Major concerns | Very low |
| Liraglutide:  Semaglutide | 0 | Some concerns | Low risk | No concerns | Major concerns | No concerns | Major concerns | Very low |
| Liraglutide:  Sotagliflozin | 0 | Some concerns | Low risk | No concerns | Major concerns | No concerns | Major concerns | Very low |
| Luseogliflozin:  Semaglutide | 0 | Some concerns | Low risk | No concerns | Major concerns | No concerns | Major concerns | Very low |
| Luseogliflozin:  Sotagliflozin | 0 | Some concerns | Low risk | No concerns | Major concerns | No concerns | Major concerns | Very low |
| Semaglutide:  Sotagliflozin | 0 | No concerns | Low risk | No concerns | Major concerns | No concerns | Major concerns | Very low |

PBO: Placebo.

20.10 Summary of study limitations of the included studies (AKI)


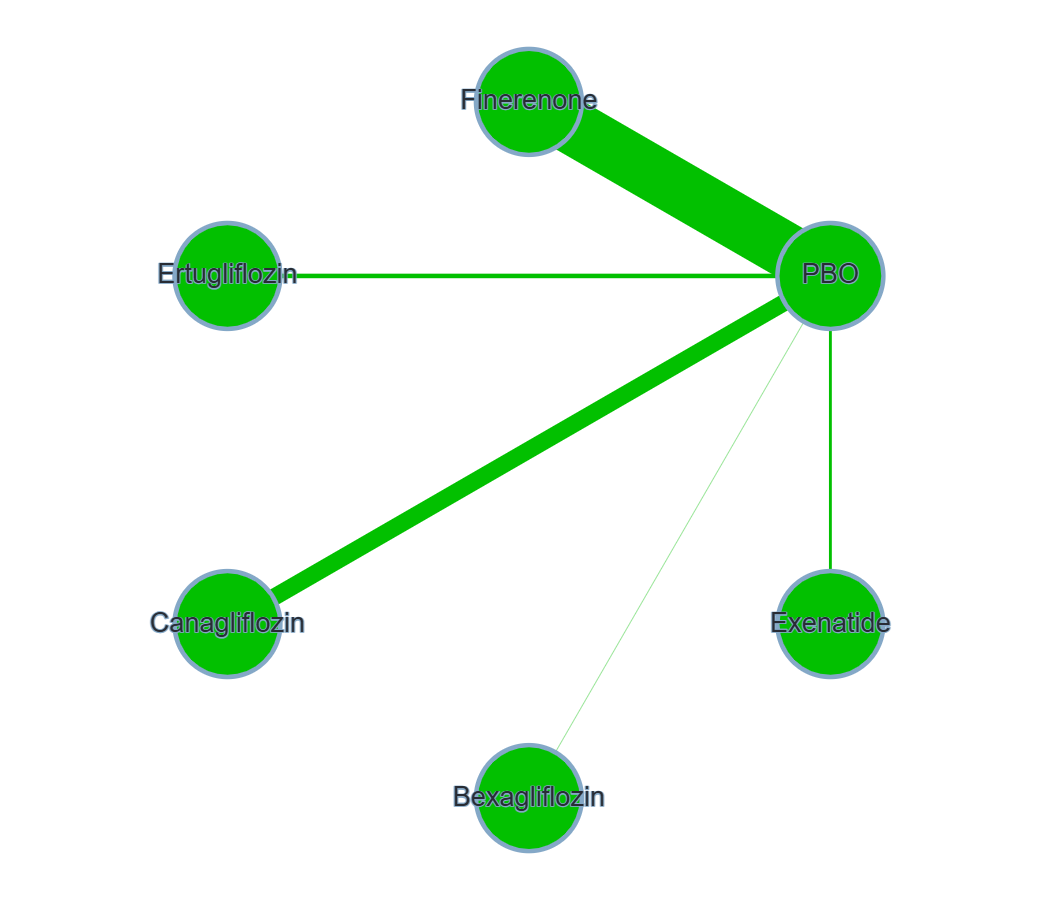


Network plot of study limitations of the included studies. Node size by equal size, node color by RoB. The colors in the circles indicate the percentage of low RoB studies (green), moderate RoB studies (yellow), high RoB studies (red) about each physical activity type. Edge width by sample size. Edge color by average RoB. The colors of the lines indicate the summative RoB assessment of each comparison. Low RoB is green, moderate RoB is yellow, high RoB is red. PBO: Placebo.

Contribution percentage of low, moderate, and high RoB comparisons to each network estimate

Low RoB is green, moderate RoB is yellow, high RoB is red.


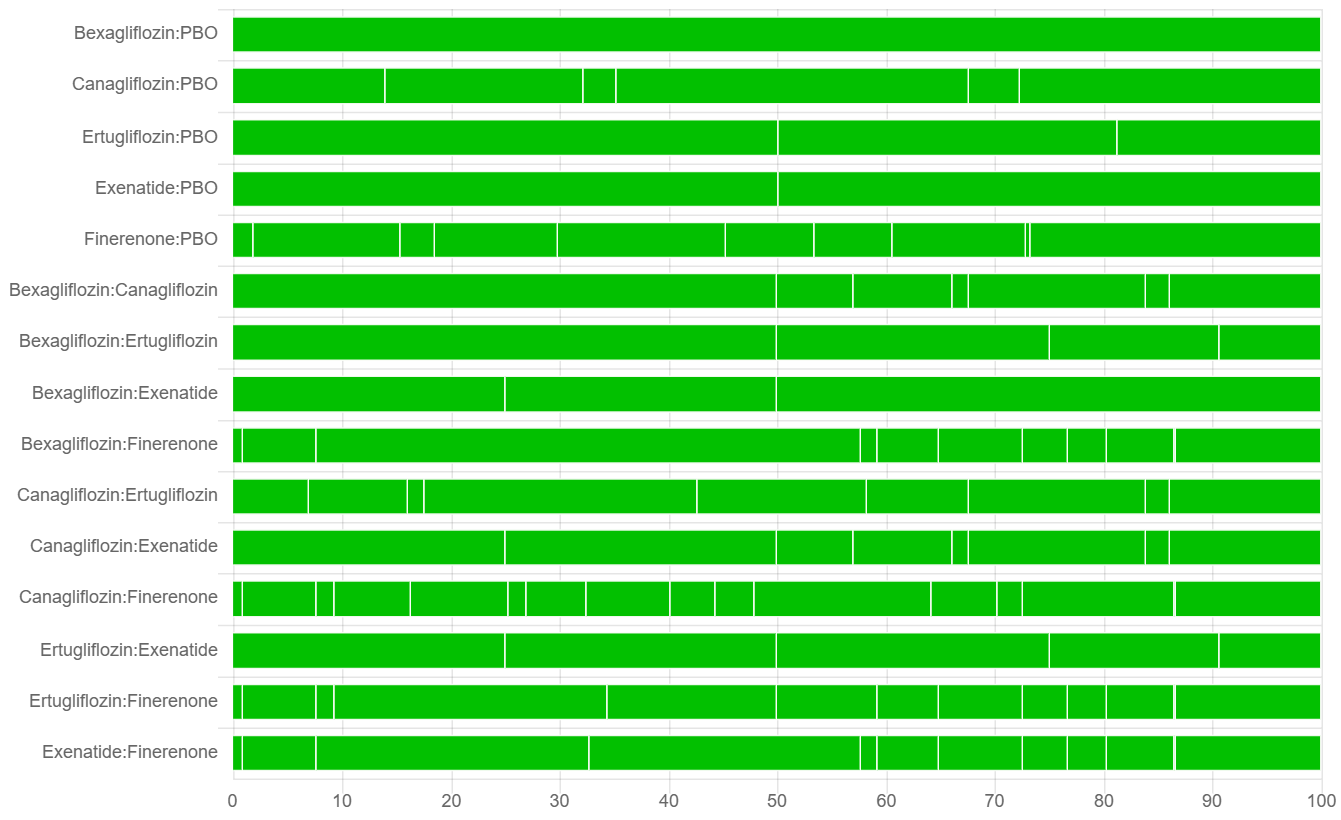


CINeMA for the outcome “AKI”

| Comparison | Number of studies | Within-study bias | Reporting bias | Indirectness | Imprecision | Heterogeneity | Incoherence | Confidence rating |
| --- | --- | --- | --- | --- | --- | --- | --- | --- |
| Bexagliflozin:  PBO | 1 | No concerns | Low risk | No concerns | Major concerns | No concerns | Major concerns | Very low |
| Canagliflozin:  PBO | 6 | No concerns | Low risk | No concerns | Major concerns | No concerns | Major concerns | Very low |
| Ertugliflozin:  PBO | 3 | No concerns | Low risk | No concerns | Major concerns | No concerns | Major concerns | Very low |
| Exenatide:PBO | 2 | No concerns | Low risk | No concerns | Major concerns | No concerns | Major concerns | Very low |
| Finerenone:  PBO | 10 | No concerns | Low risk | No concerns | Major concerns | No concerns | Major concerns | Very low |
| Bexagliflozin:  Canagliflozin | 0 | No concerns | Low risk | No concerns | Major concerns | No concerns | Major concerns | Very low |
| Bexagliflozin:  Ertugliflozin | 0 | No concerns | Low risk | No concerns | Major concerns | No concerns | Major concerns | Very low |
| Bexagliflozin:  Exenatide | 0 | No concerns | Low risk | No concerns | Major concerns | No concerns | Major concerns | Very low |
| Bexagliflozin:  Finerenone | 0 | No concerns | Low risk | No concerns | Major concerns | No concerns | Major concerns | Very low |
| Canagliflozin:  Ertugliflozin | 0 | No concerns | Low risk | No concerns | Major concerns | No concerns | Major concerns | Very low |
| Canagliflozin:  Exenatide | 0 | No concerns | Low risk | No concerns | Major concerns | No concerns | Major concerns | Very low |
| Canagliflozin:  Finerenone | 0 | No concerns | Low risk | No concerns | Major concerns | No concerns | Major concerns | Very low |
| Ertugliflozin:  Exenatide | 0 | No concerns | Low risk | No concerns | Major concerns | No concerns | Major concerns | Very low |
| Ertugliflozin:  Finerenone | 0 | No concerns | Low risk | No concerns | Major concerns | No concerns | Major concerns | Very low |
| Exenatide:  Finerenone | 0 | No concerns | Low risk | No concerns | Major concerns | No concerns | Major concerns | Very low |

PBO: Placebo.

Appendix 21: Subgroup Analysis

The funnel plot indicated potential biases in eGFR, HbA1c, and body weight. Consequently, we conducted subgroup analyses of these three outcome measures to investigate the sources of heterogeneity.

21.1 Forest plot of eGFR


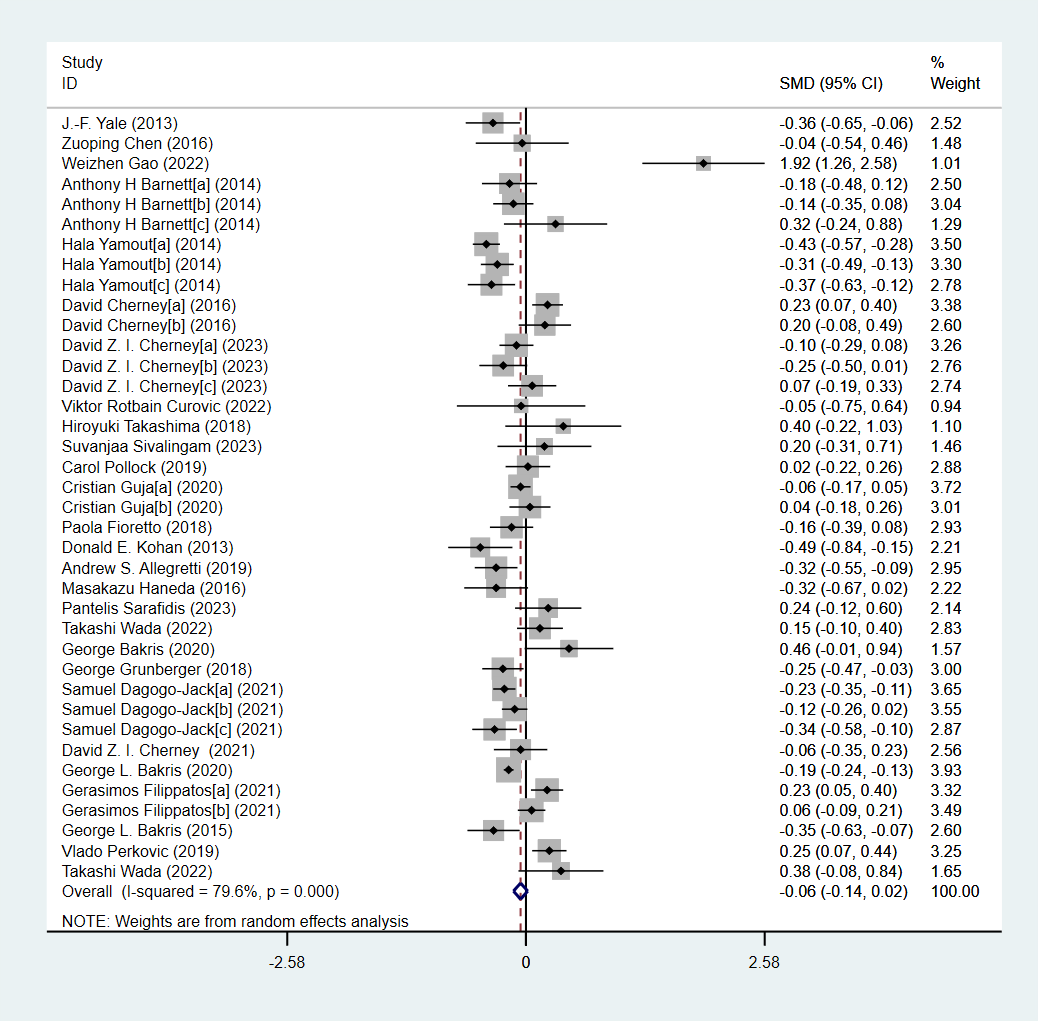


As illustrated in this figure, I^2^ = 79.6%, which exceeds 75%, indicating high statistical heterogeneity. Consequently, a subgroup analysis was conducted.

Subgroup analysis with country as variable


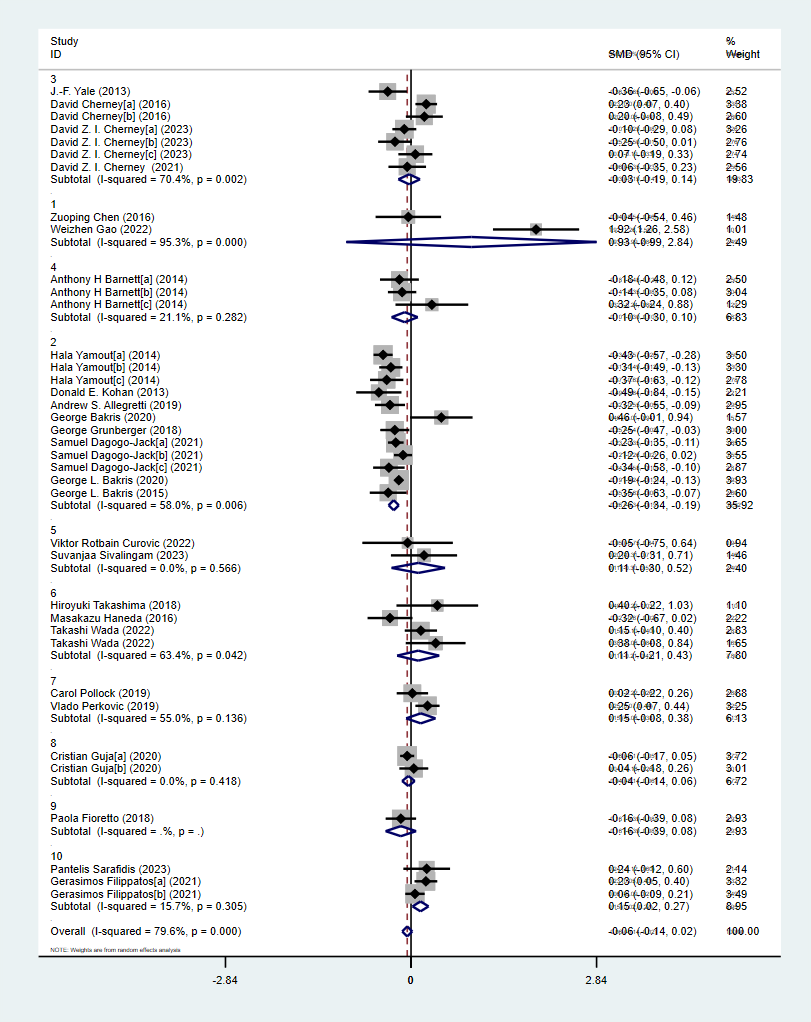


1: China; 2: America; 3: Canada; 4: The United Kingdom, UK; 5: Denmark; 6: Japan; 7: Australia; 8: Romania; 9: Italy; 10: Greece.

Subgroup analysis of eGFR based on country as a variable indicated that the country of origin may serve as a potential source of heterogeneity. Heterogeneity was observed to decrease when the countries of origin were limited to the United Kingdom, Denmark, Australia, Romania, and Greece.

Subgroup analysis with duration of medication as variable


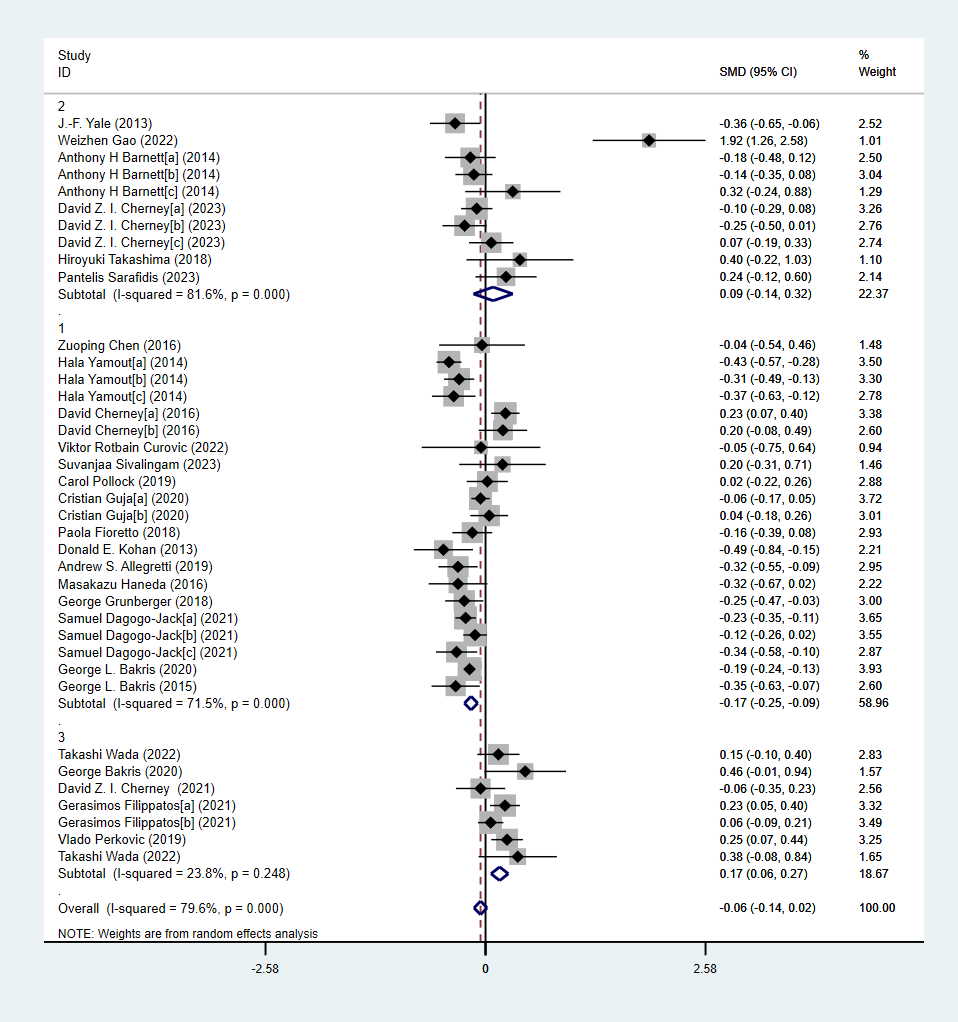


1: less than 26weeks (including 26weeks); 2: from 26 to 52 weeks (inclusive 52 weeks); 3: More than 52 weeks.

Subgroup analysis of eGFR, with treatment duration as a variable, indicated that treatment durations of less than 26 weeks, 26 to 52 weeks, and greater than 52 weeks might contribute to potential heterogeneity. A significant reduction in heterogeneity was observed when the duration of medication exceeded 52 weeks.


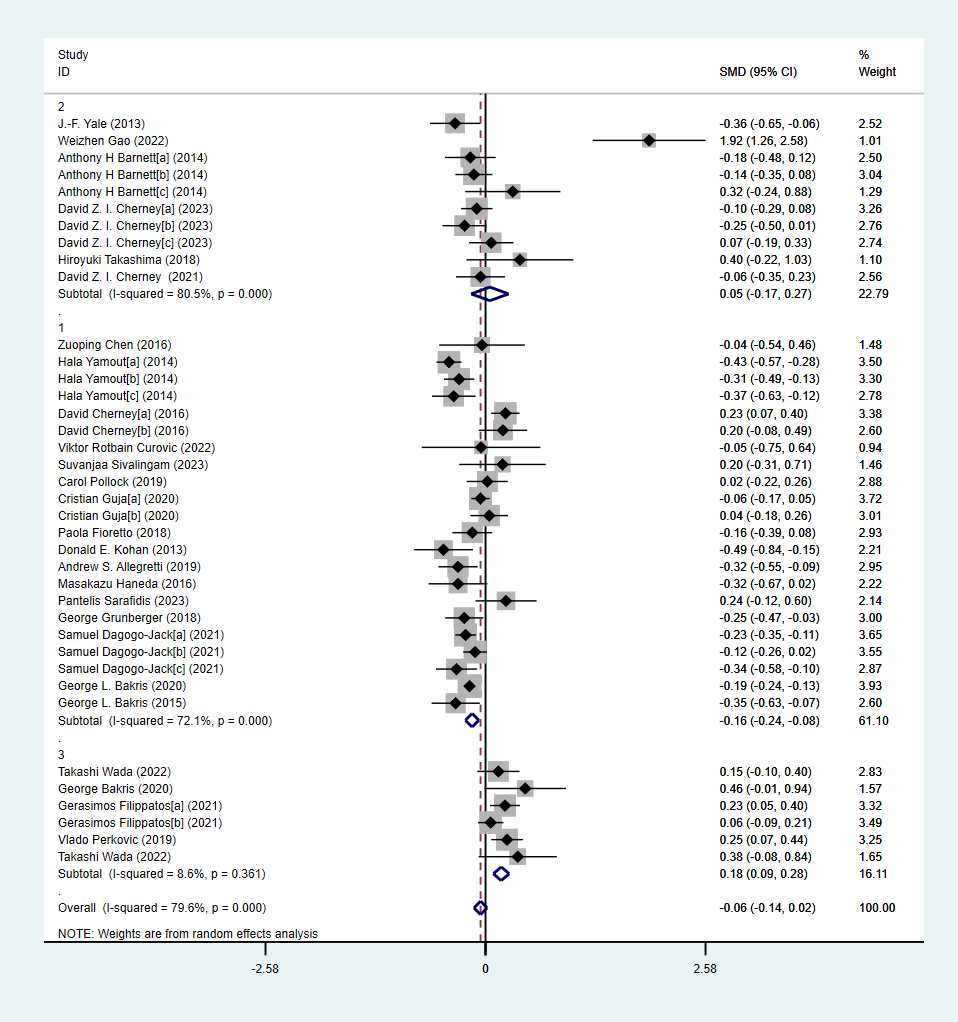


1: less than 52 weeks (inclusive 52 weeks); 2: from 52 weeks to 104 weeks (inclusive 104 weeks); 3: more than 104 weeks.

Subgroup analysis of eGFR, with treatment duration as a variable, indicated that treatment durations of less than 52 weeks, 52 to 104 weeks, and greater than 104 weeks might contribute to potential heterogeneity. A significant reduction in heterogeneity was observed when the duration of medication exceeded 104 weeks.

Subgroup analysis with type of medicationas variable


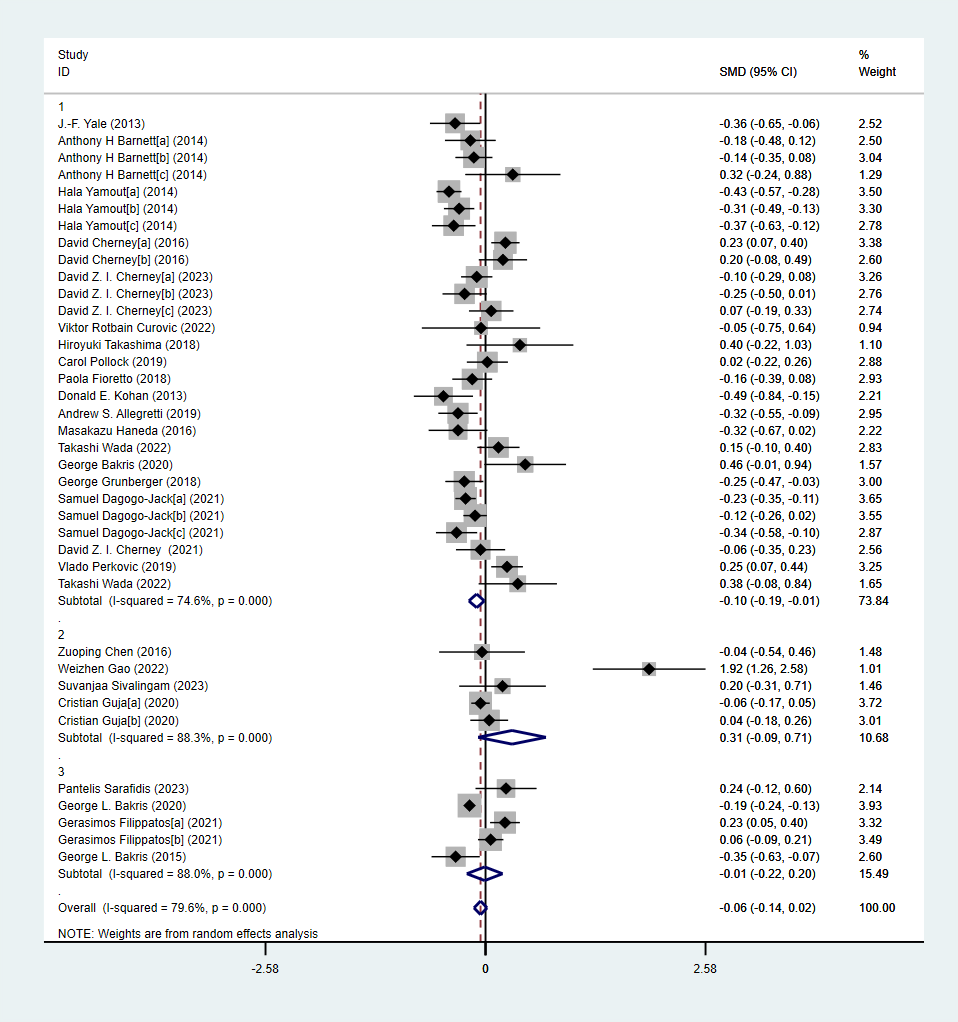


1: SGLT-2 inhibitors; 2: GLP-1 receptor agonists; 3: Finerenone.

Subgroup analysis of eGFR, with drug type as a stratification variable, revealed that the type of drug treatment did not contribute to heterogeneity.

Subgroup analysis with number of participants variable


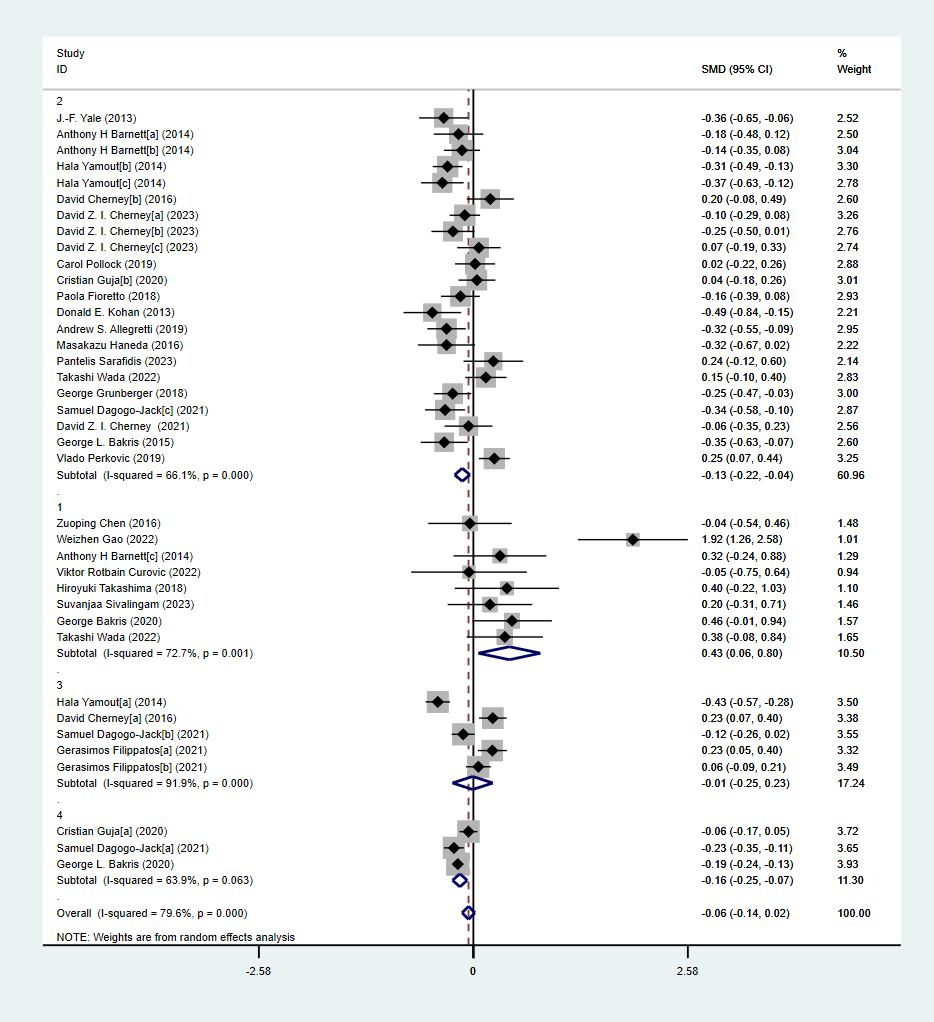


1: Number of people less than 100; 2: Number of people from 100 to 500; 3: Number of people from 500 to 1000; 4: Number of people more than 1000.

As shown in the figure, insignificant heterogeneity was observed only in the group of "number of participants more than 1000".

21.2 Forest plot of HbA1c


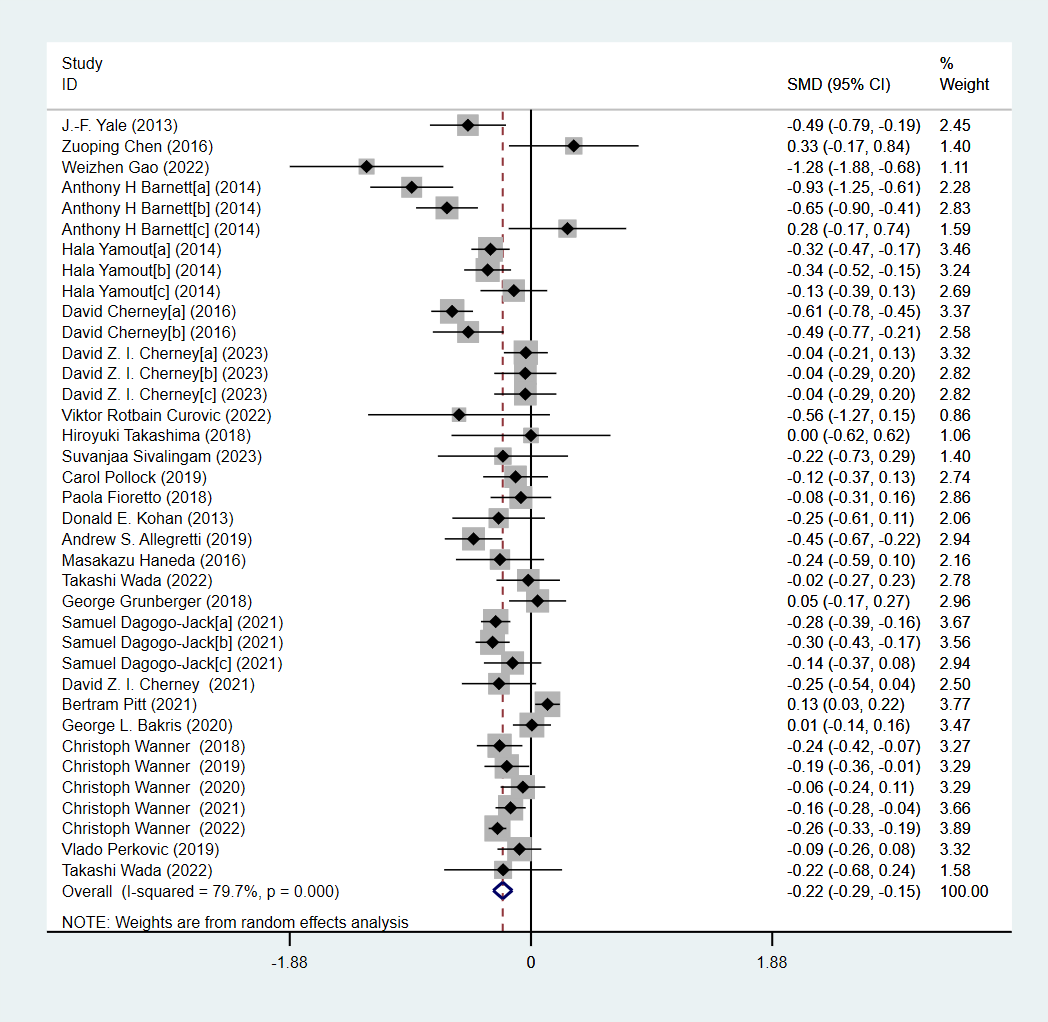


As illustrated in this figure, I^2^ = 79.7%, which exceeds 75%, indicating high statistical heterogeneity. Consequently, a subgroup analysis was conducted.

Subgroup analysis with country as variable


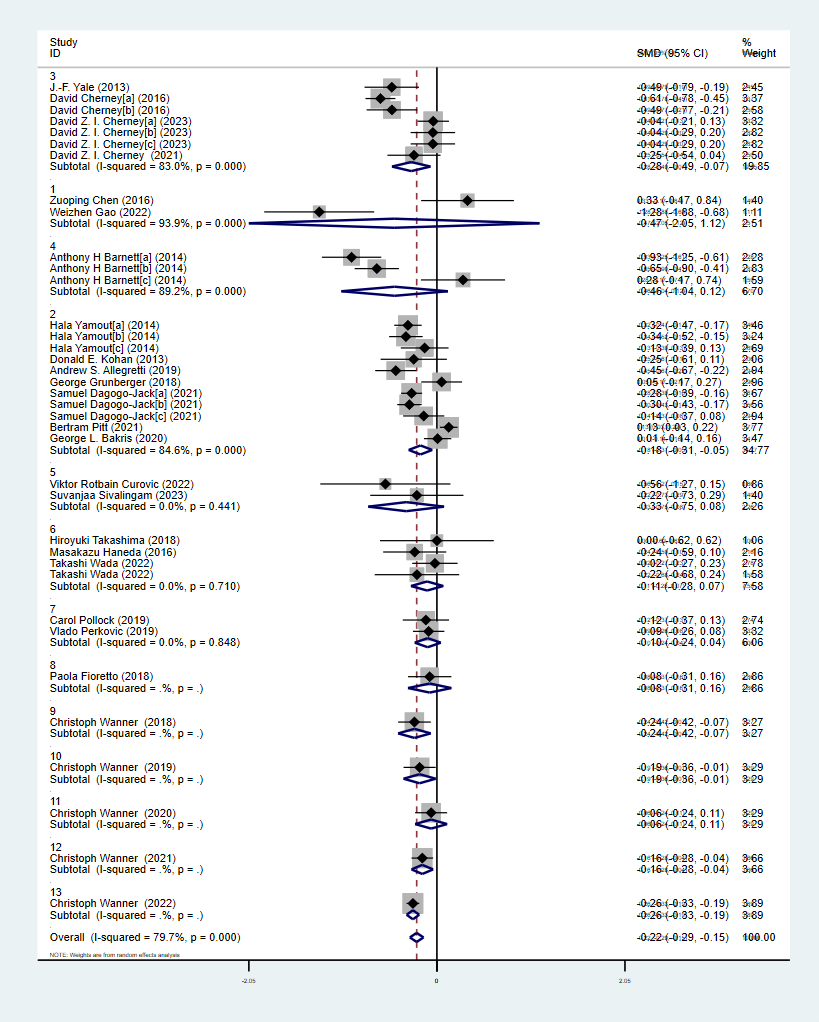


1: China; 2: America; 3: Canada; 4: The United Kingdom, UK; 5: Denmark; 6: Japan; 7: Australia; 8: Italy; 9: Germany

Subgroup analysis of HbA1c based on country as a variable indicated that the country of origin may serve as a potential source of heterogeneity. Lower heterogeneity was observed in studies originating from Denmark, Japan, and Australia.

Subgroup analysis with duration of medication as variable


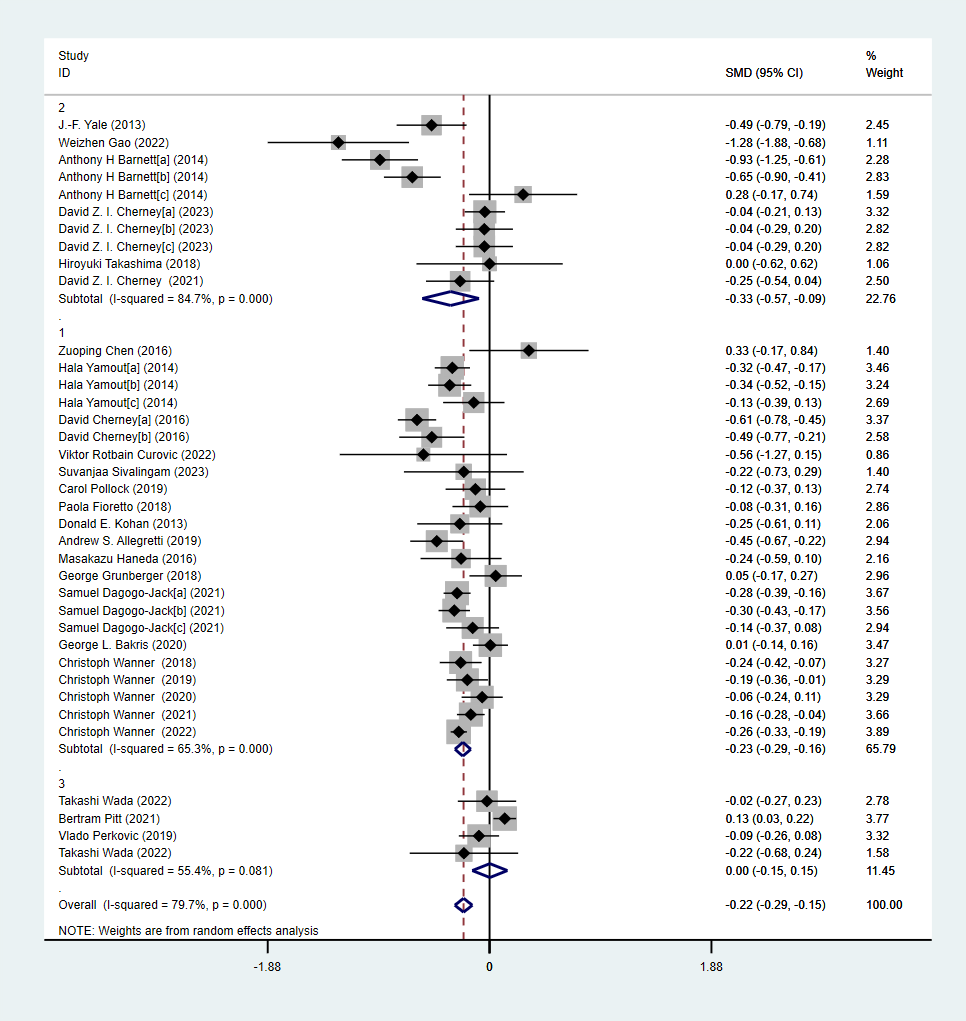


1: less than 26weeks (including 26weeks); 2: from 26 to 52 weeks (including 52 weeks); 3: More than 52 weeks.

Subgroup analysis of eGFR, with treatment duration as a variable, indicated that treatment durations of less than 26 weeks, 26 to 52 weeks, and greater than 52 weeks might contribute to potential heterogeneity. Heterogeneity was not evident when the duration of medication was longer than 52 weeks.

Subgroup analysis with type of medicationas variable


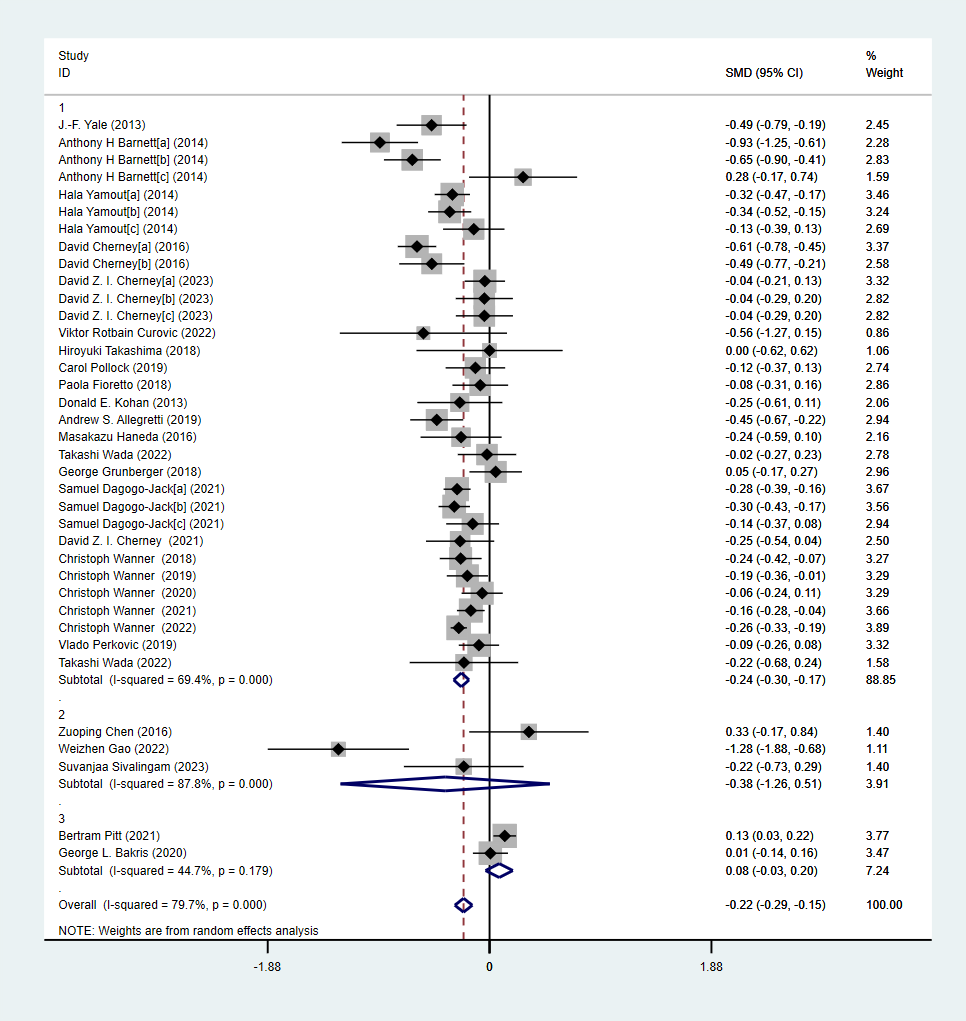


1: SGLT-2 inhibitors; 2: GLP-1 receptor agonists; 3: Finerenone.

Subgroup analysis of HbA1c based on type of medicationas as a variable indicated that the type of medicationas may serve as a potential source of heterogeneity. We observed reduced heterogeneity in the experimental group treated with Finerenone.

Subgroup analysis with number of participants variable


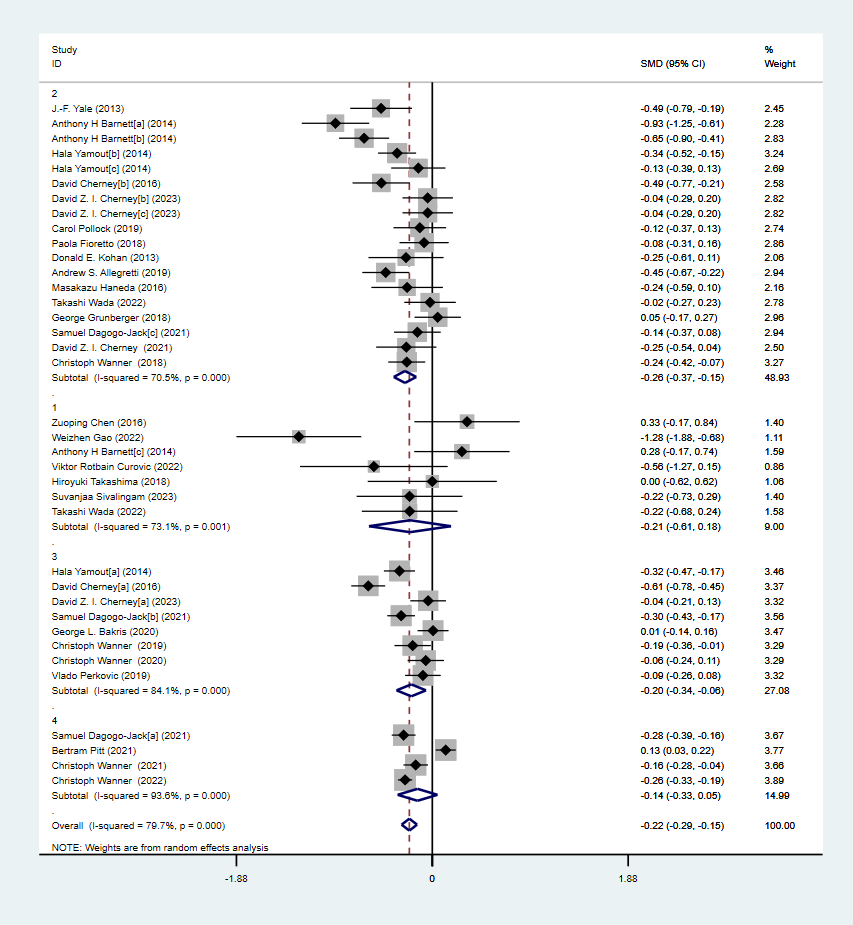


1: Number of people less than 100; 2: Number of people from 100 to 500; 3: Number of people from 500 to 1000; 4: Number of people more than 1000.

As depicted in the figure, the number of participants did not significantly contribute to the high statistical heterogeneity observed in HbA1c.

21.3 Forest plot of Body Weight


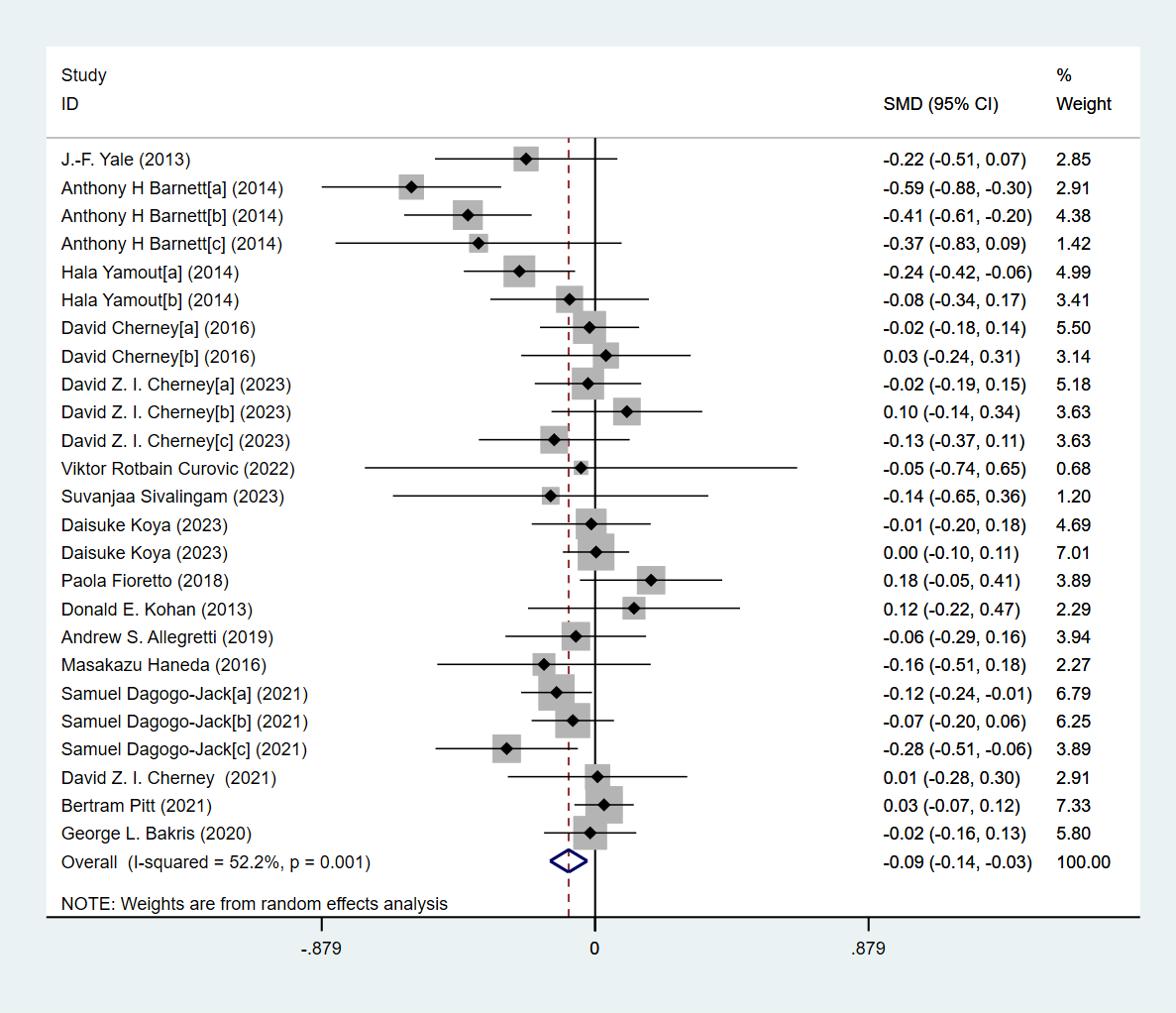


As illustrated in this figure, I^2^ = 52.2%, which exceeds 50%, indicating moderate statistical heterogeneity. Consequently, a subgroup analysis was conducted.

Subgroup analysis with country as variable


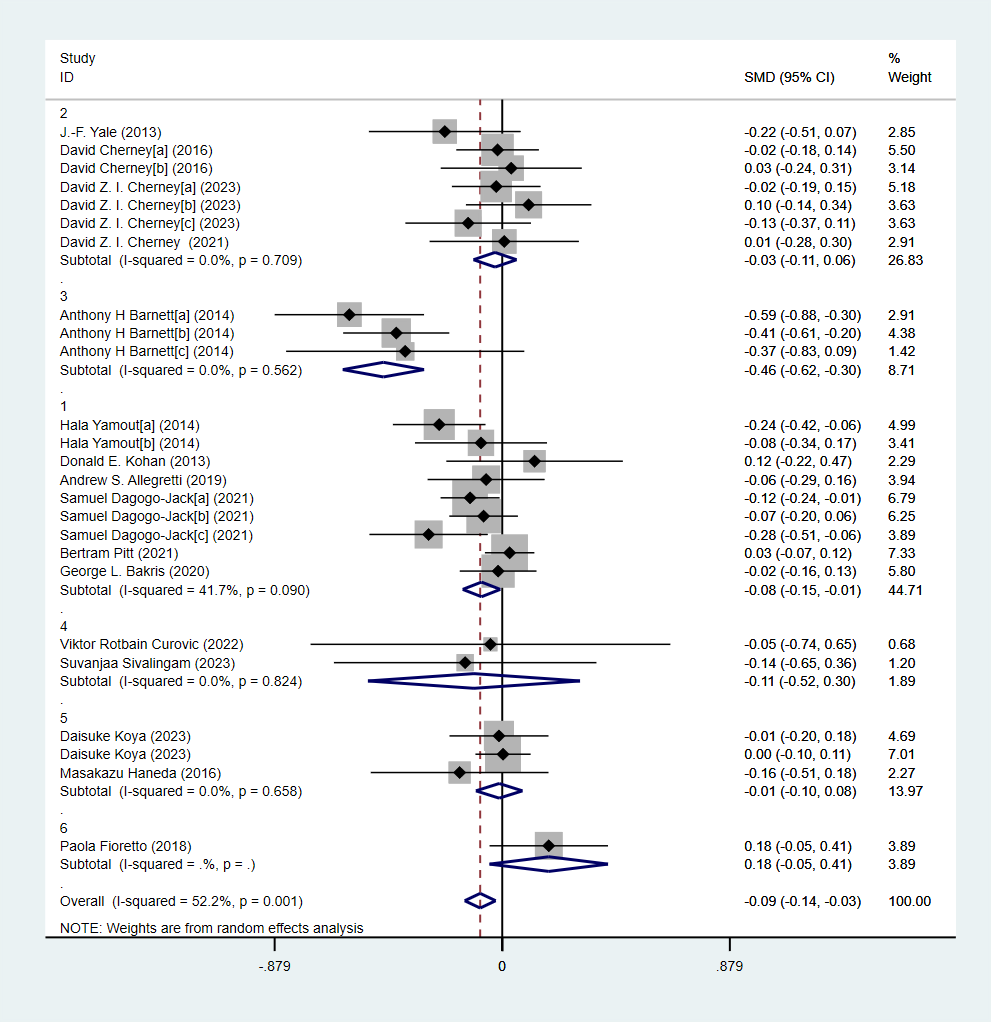


1: America; 2: Canada; 3: The United Kingdom, UK; 4: Denmark; 5: Japan; 6: Italy.

As illustrated in the figure, the original source of the article may contribute to the moderate heterogeneity observed in body weight.

Subgroup analysis with duration of medication as variable


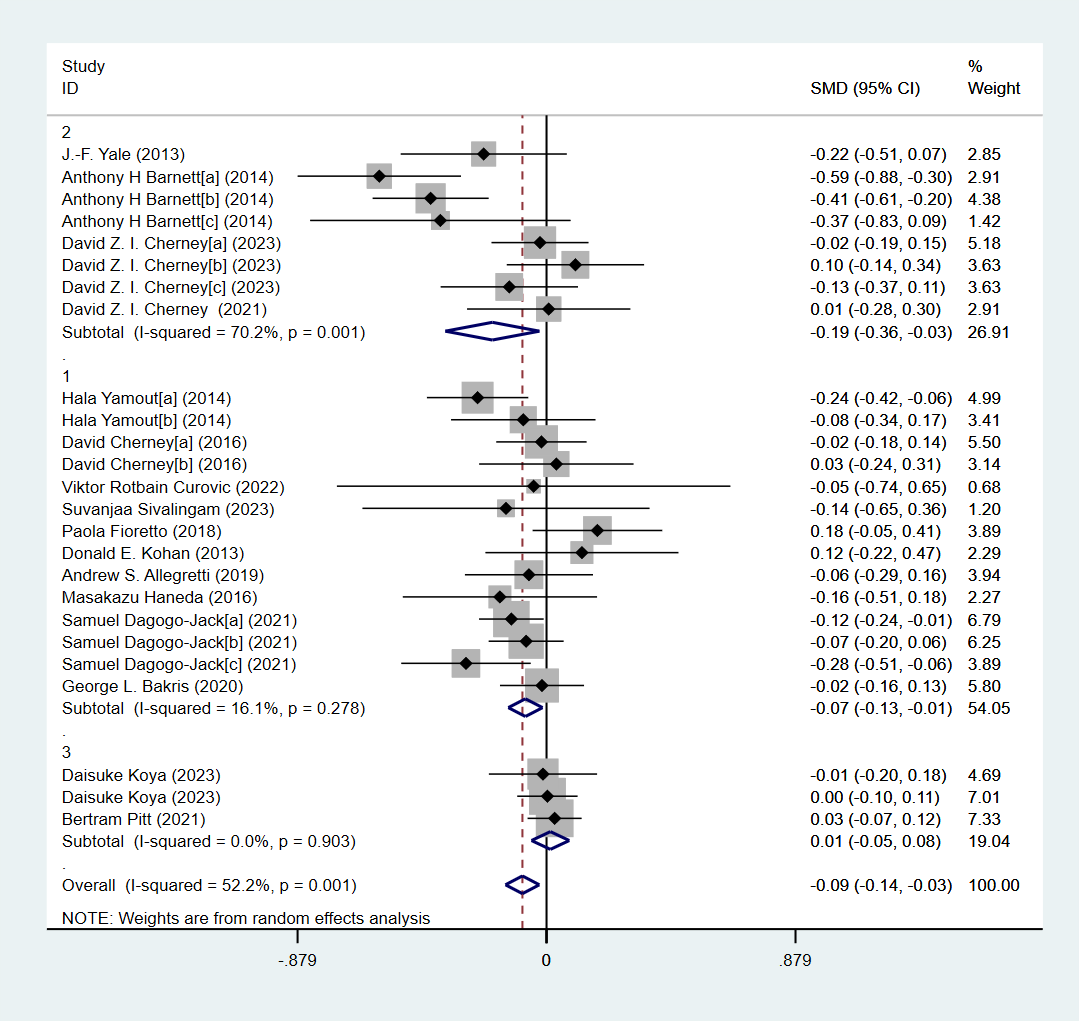


1: less than 26weeks (including 26weeks); 2: from 26 to 52 weeks (including 52 weeks); 3: More than 52 weeks.

As demonstrated, the duration of medication use may serve as a potential source of heterogeneity. Notably, statistical heterogeneity was significantly reduced for treatment durations of 26 weeks or less, and for durations exceeding 52 weeks.

Subgroup analysis with type of medicationas variable


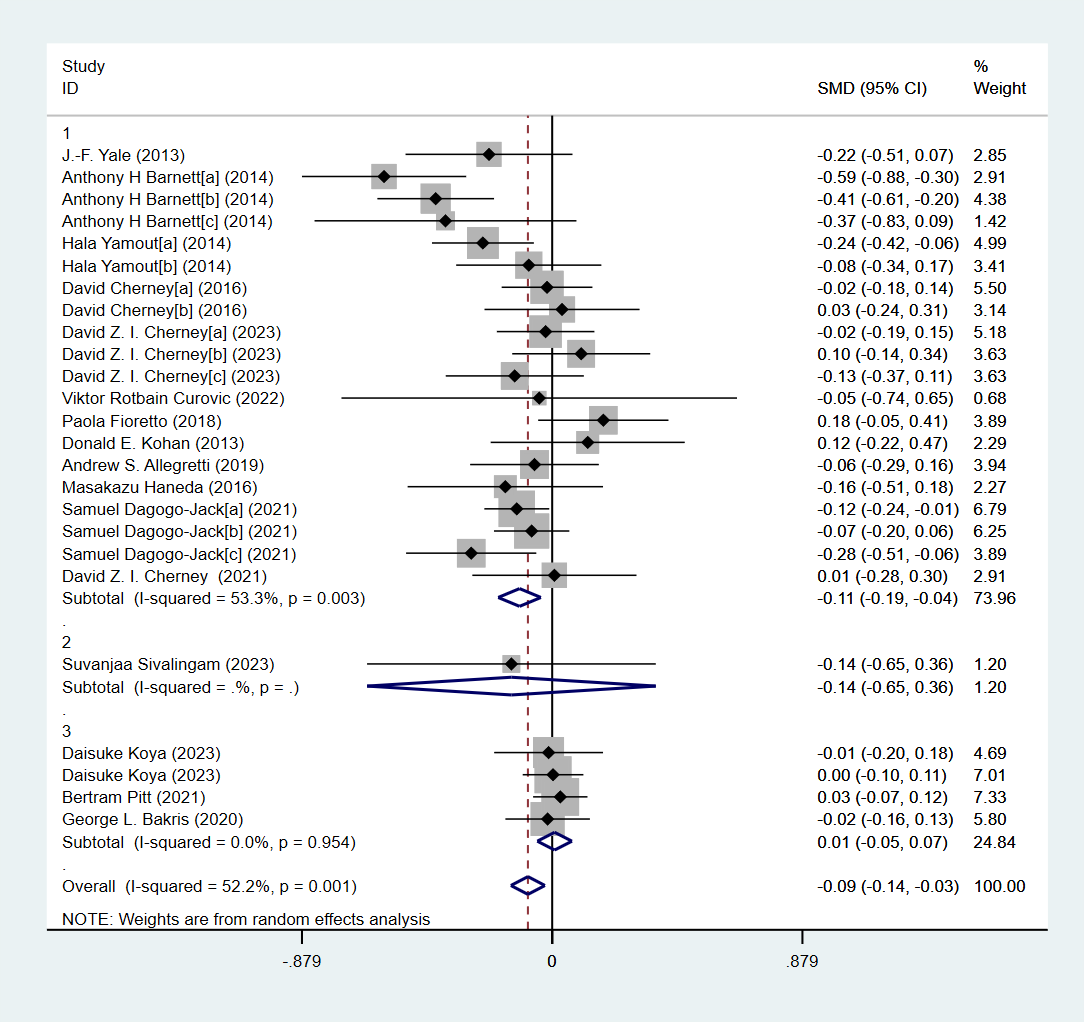


1: SGLT-2 inhibitors; 2: GLP-1 receptor agonists; 3: Finerenone.

Subgroup analysis of body weight based on type of medicationas as a variable indicated that the type of medicationas may serve as a potential source of heterogeneity. We observed reduced heterogeneity in the experimental group treated with Finerenone.

Subgroup analysis with number of participants variable


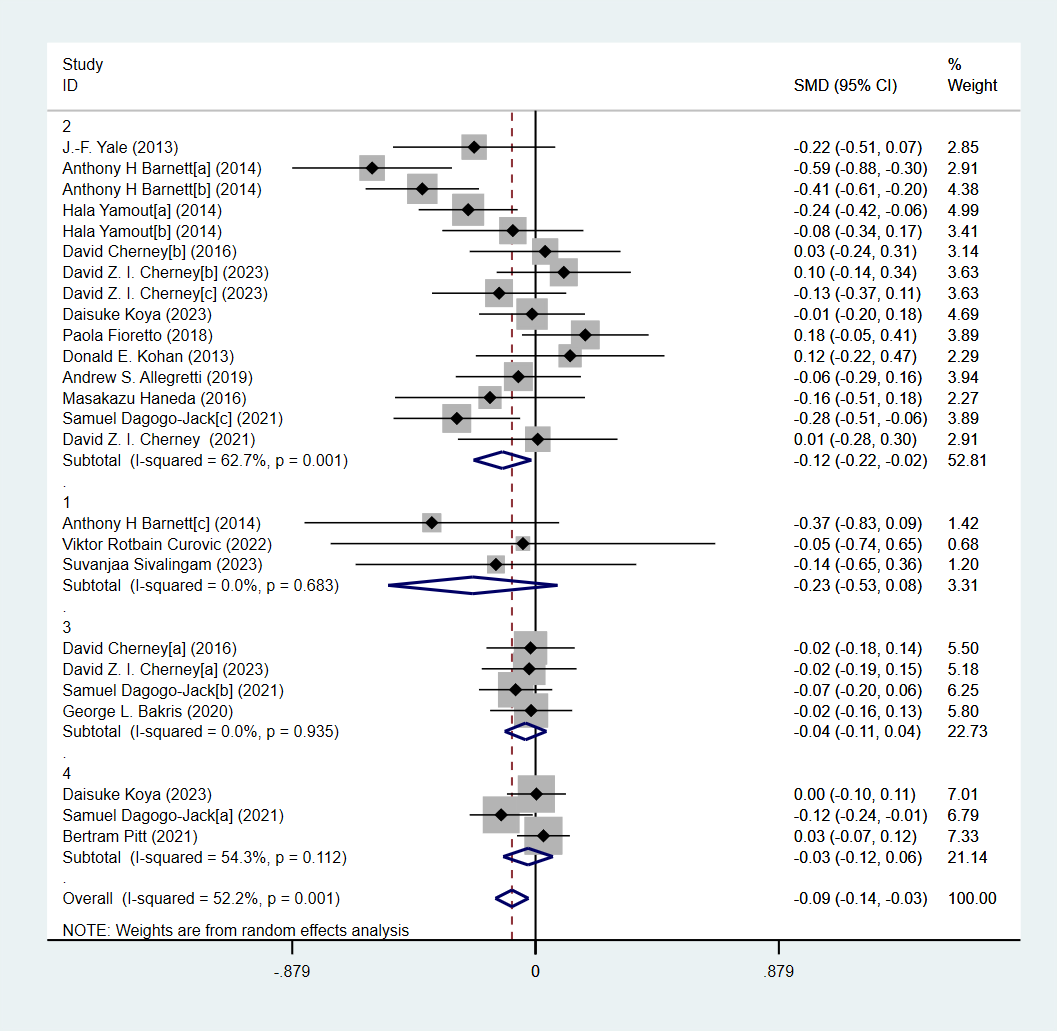


1: Number of people less than 100; 2: Number of people from 100 to 500; 3: Number of people from 500 to 1000; 4: Number of people more than 1000.

As illustrated in the figure, a reduction in heterogeneity was observed across subgroups comprising fewer than 100 participants, those with 500 to 1000 participants, and those exceeding 1000 participants.
